# Small class sizes for improving student achievement in primary and secondary schools: a systematic review

**DOI:** 10.4073/csr.2018.10

**Published:** 2018-10-11

**Authors:** Trine Filges, Christoffer Scavenius Sonne‐Schmidt, Bjørn Christian Viinholt Nielsen

## Abstract

This Campbell systematic review examines the impact of class size on academic achievement. The review summarises findings from 148 reports from 41 countries. Ten studies were included in the meta‐analysis.

Included studies concerned children in grades kindergarten to 12 (or the equivalent in European countries) in general education. The primary focus was on measures of academic achievement. All study designs that used a well‐defined control group were eligible for inclusion.

A total of 127 studies, consisting of 148 papers, met the inclusion criteria. These 127 studies analysed 55 different populations from 41 different countries. A large number of studies (45) analysed data from the Student Teacher Achievement Ratio (STAR) experiment which was for class size reduction in grade K‐3 in the US in the eighties. However only ten studies, including four of the STAR programme, could be included in the meta‐analysis.

Overall, the evidence suggests at best a small effect on reading achievement. There is a negative, but statistically insignificant, effect on mathematics.

For the non‐STAR studies the primary study effect sizes for reading were close to zero but the weighted average was positive and statistically significant. There was some inconsistency in the direction of the primary study effect sizes for mathematics and the weighted average effect was negative and statistically non‐significant.

The STAR results are more positive, but do not change the overall finding. All reported results from the studies analysing STAR data indicated a positive effect of smaller class sizes for both reading and maths, but the average effects are small

**Plain language summary:**

**Small class size has at best a small effect on academic achievement**

Reducing class size is seen as a way of improving student performance. But larger class sizes help control education budgets. The evidence suggests at best a small effect on reading achievement. There is a negative, but statistically insignificant, effect on mathematics, so it cannot be ruled out that some children may be adversely affected.

**What is this review about?**

Increasing class size is one of the key variables that policy makers can use to control spending on education.

But the consensus among many in education research is that smaller classes are effective in improving student achievement which has led to a policy of class size reductions in a number of US states, the UK, and the Netherlands. This policy is disputed by those who argue that the effects of class size reduction are only modest and that there are other more cost‐effective strategies for improving educational standards.

Despite the important policy and practice implications of the topic, the research literature on the educational effects of class‐size differences has not been clear.

This review systematically reports findings from relevant studies that measure the effects of class size on academic achievement.

**What is the aim of this review?**

This Campbell systematic review examines the impact of class size on academic achievement. The review summarises findings from 148 reports from 41 countries. Ten studies were included in the meta‐analysis.

**What are the main findings of this review?**

**What studies are included?**

Included studies concerned children in grades kindergarten to 12 (or the equivalent in European countries) in general education. The primary focus was on measures of academic achievement. All study designs that used a well‐defined control group were eligible for inclusion.

A total of 127 studies, consisting of 148 papers, met the inclusion criteria. These 127 studies analysed 55 different populations from 41 different countries. A large number of studies (45) analysed data from the Student Teacher Achievement Ratio (STAR) experiment which was for class size reduction in grade K‐3 in the US in the eighties. However only ten studies, including four of the STAR programme, could be included in the meta‐analysis.

**What are the main results?**

Overall, the evidence suggests at best a small effect on reading achievement. There is a negative, but statistically insignificant, effect on mathematics.

For the non‐STAR studies the primary study effect sizes for reading were close to zero but the weighted average was positive and statistically significant. There was some inconsistency in the direction of the primary study effect sizes for mathematics and the weighted average effect was negative and statistically non‐significant.

The STAR results are more positive, but do not change the overall finding. All reported results from the studies analysing STAR data indicated a positive effect of smaller class sizes for both reading and maths, but the average effects are small.

**What do the findings of this review mean?**

There is some evidence to suggest that there is an effect of reducing class size on reading achievement, although the effect is very small. There is no significant effect on mathematics achievement, though the average is negative meaning a possible adverse impact on some students cannot be ruled out.

The overall reading effect corresponds to a 53 per cent chance that a randomly selected score of a student from the treated population of small classes is greater than the score of a randomly selected student from the comparison population of larger classes. This is a very small effect.

Class size reduction is costly. The available evidence points to no or only very small effect sizes of small classes in comparison to larger classes. Moreover, we cannot rule out the possibility that small classes may be counterproductive for some students. It is therefore crucial to know more about the relationship between class size and achievement in order to determine where money is best allocated.

**How up‐to‐date is this review?**

The review authors searched for studies published up to February 2017. This Campbell systematic review was published in 2018.

**Executive Summary/Abstract:**

**BACKGROUND**

Increasing class size is one of the key variables that policy makers can use to control spending on education. Reducing class size to increase student achievement is an approach that has been tried, debated, and analysed for several decades. Despite the important policy and practice implications of the topic, the research literature on the educational effects of class‐size differences has not been clear.

The consensus among many in education research, that smaller classes are effective in improving student achievement has led to a policy of class size reductions in a number of U.S. states, the United Kingdom, and the Netherlands. This policy is disputed by those who argue that the effects of class size reduction are only modest and that there are other more cost‐effective strategies for improving educational standards.

**OBJECTIVES**

The purpose of this review is to systematically uncover relevant studies in the literature that measure the effects of class size on academic achievement. We will synthesize the effects in a transparent manner and, where possible, we will investigate the extent to which the effects differ among different groups of students such as high/low performers, high/low income families, or members of minority/non‐minority groups, and whether timing, intensity, and duration have an impact on the magnitude of the effect.

**SEARCH METHODS**

Relevant studies were identified through electronic searches of bibliographic databases, internet search engines and hand searching of core journals. Searches were carried out to February 2017. We searched to identify both published and unpublished literature. The searches were international in scope. Reference lists of included studies and relevant reviews were also searched.

**SELECTION CRITERIA**

The intervention of interest was a reduction in class size. We included children in grades kindergarten to 12 (or the equivalent in European countries) in general education. The primary focus was on measures of academic achievement. All study designs that used a well‐defined control group were eligible for inclusion. Studies that utilized qualitative approaches were not included.

**DATA COLLECTION AND ANALYSIS**

The total number of potential relevant studies constituted 8,128 hits. A total of 127 studies, consisting of 148 papers, met the inclusion criteria and were critically appraised by the review authors. The 127 studies analysed 55 different populations from 41 different countries.

A large number of studies (45) analysed data from the STAR experiment (class size reduction in grade K‐3) and its follow up data.

Of the 82 studies not analysing data from the STAR experiment, only six could be used in the data synthesis. Fifty eight studies could not be used in the data synthesis as they were judged to have too high risk of bias either due to confounding (51), other sources of bias (4) or selective reporting of results (3). Eighteen studies did not provide enough information enabling us to calculate an effects size and standard error or did not provide results in a form enabling us to use it in the data synthesis.

Meta‐analysis was used to examine the effects of class size on student achievement in reading and mathematics. Random effects models were used to pool data across the studies not analysing STAR data. Pooled estimates were weighted using inverse variance methods, and 95% confidence intervals were estimated. Effect sizes were measured as standardised mean differences (SMD). It was only possible to perform a meta‐analysis by the end of the treatment year (end of the school year).

Four of the studies analysing STAR data provided effect estimates that could be used in the data synthesis. The four studies differed in terms of both the chosen comparison condition and decision rules in selecting a sample for analysis. Which of these four studies' effect estimates should be included in the data synthesis was not obvious as the decision rule (concerning studies using the same data set) as described in the protocol could not be used. Contrary to usual practice we therefore report the results of all four studies and do not pool the results with the studies not analysing STAR data except in the sensitivity analysis. We took into consideration the ICC in the results reported for the STAR experiment and corrected the effect sizes and standard errors using ρ = 0.22. No adjustment due to clustering was necessary for the studies not analysing STAR data.

Sensitivity analysis was used to evaluate whether the pooled effect sizes were robust across components of methodological quality, in relation to inclusion of a primary study result with an unclear sign, inclusion of effect sizes from the STAR experiment and to using a one‐student reduction in class size in studies using class size as a continuous variable.

**RESULTS**

All studies, not analysing STAR data, reported outcomes by the end of the treatment (end of the school year) only. The STAR experiment was a four year longitudinal study with outcomes reported by the end of each school year. The experiment was conducted to assess the effectiveness of small classes compared with regular‐sized classes and of teachers' aides in regular‐sized classes on improving cognitive achievement in kindergarten and in the first, second, and third grades. The goal of the STAR experiment was to have approximately 100 small classes with 13‐17 students (S), 100 regular classes with 22‐25 students (R), and 100 regular with aide classes with 22‐25 students (RA).

Of the six studies not analysing STAR, only five were used in the meta‐analysis as the direction of the effect size in one study was unclear. The studies were from USA, the Netherlands and France, one was a RCT and five were NRS. The grades investigated spanned kindergarten to 3. Grade and one study investigated grade 10. The sample sizes varied; the smallest study investigated 104 students and the largest study investigated 11,567 students. The class size reductions varied from a minimum of one student in four studies, a minimum of seven students in another study to a minimum of 8 students in the last study.

All outcomes were scaled such that a positive effect size favours the students in small classes, i.e. when an effect size is *positive* a class size reduction improves the students' achievement.

Primary study effect sizes for reading lied in the range ‐0.08 to 0.14. Three of the study‐level effects were statistically non‐significant. The weighted average was positive and statistically significant. The random effects weighted standardised mean difference was 0.11 (95% CI 0.05 to 0.16) which may be characterised as small. There is some inconsistency in the direction of the effect sizes between the primary studies. Primary study effect sizes for mathematics lies in the range ‐0.41 to 0.11. Two of the study‐level effects were statistically non‐significant. The weighted average was negative and statistically non‐significant. The random effects weighted standardised mean difference was ‐0.03 (95% CI ‐0.22 to 0.16). There is some inconsistency in the direction as well as the magnitude of the effect sizes between the primary studies.

All reported results from the four studies analysing STAR data indicated a positive effect favouring the treated; all of the study‐level effects were statistically significant. The study‐level effect sizes for reading varied between 0.17 and 0.34 and the study‐level effect sizes for mathematics varied between 0.15 and 0.33.

There were no appreciable changes in the results when we included the extremes of the range of effect sizes from the STAR experiment. The reading outcome lost statistical significance when the effect size from the primary study reporting a result with an unclear direction was included with a negative sign and when the results from the studies using class size as a continuous variable were included with a one student reduction in class size instead of a standard deviation reduction in class size.

Otherwise, there were no appreciable changes in the results.

**AUTHORS’ CONCLUSIONS**

There is some evidence to suggest that there is an effect of reducing class size on reading achievement, although the effect is very small. We found a statistically significant positive effect of reducing the class size on reading. The effect on mathematics achievement was not statistically significant, thus it is uncertain if there may be a negative effect.

The overall reading effect corresponds to a 53 per cent chance that a randomly selected score of a student from the treated population of small classes is greater than the score of a randomly selected student from the comparison population of larger classes. The overall effect on mathematics achievement corresponds to a 49 per cent chance that a randomly selected score of a student from the treated population of small classes is greater than the score of a randomly selected student from the comparison population of larger classes.

Class size reduction is costly and the available evidence points to no or only very small effect sizes of small classes in comparison to larger classes. Taking the individual variation in effects into consideration, we cannot rule out the possibility that small classes may be counterproductive for some students. It is therefore crucial to know more about the relationship between class size and achievement and how it influences what teachers and students do in the classroom in order to determine where money is best allocated.

## 1 Background

### 1.1 THE PROBLEM, CONDITION OR ISSUE

Increasing class size is one of the key variables that policy makers can use to control spending on education. The average class size at the lower secondary level is 23 students in OECD countries, but there are significant differences, ranging from over 32 in Japan and Korea to 19 or below in Estonia, Iceland, Luxembourg, Slovenia and the United Kingdom (OECD, 2012). On the other hand, reducing class size to increase student achievement is an approach that has been tried, debated, and analysed for several decades. Between 2000 and 2009, many countries invested additional resources to decrease class size (OECD, 2012).

Despite the important policy and practice implications of the topic, the research literature on the educational effects of class‐size differences has not been clear. A large part of the research on the effects of class size has found that smaller class sizes improve student achievement (for example Finn & Achilles, 1999; Konstantopoulos, 2009; Molnar et al., 1999; Schanzenbach, 2007). The consensus among many in education research that smaller classes are effective in improving student achievement has led to a policy of class size reductions in a number of U.S. states, the United Kingdom, and the Netherlands. This policy is disputed by those who argue that the effects of class size reduction are only modest and that there are other more cost‐effective strategies for improving educational standards (Hattie, 2005; Hedges, Laine, & Greenwald, 1994; Rivkin, Hanushek, & Kain, 2005). There is no consensus in the literature as to whether class size reduction can pass a cost‐benefit test (Dustmann, Rajah & van Soest, 2003; Dynarski, Hyman & Schanzenbach, 2011; Finn, Gerber & Boyd‐Zaharias, 2005; Muenning & Woolf, 2007).

As it is costly to reduce class size, it is important to consider the types of students who might benefit most from smaller class sizes and to consider the timing, intensity, and duration of class size reduction as well. Low socioeconomic status is strongly associated with low school performance. Results from the Programme for International Student Assessment (PISA) point to the fact that most of the students who perform poorly in PISA are from socio‐economically disadvantaged backgrounds (OECD, 2010). Across OECD countries, a student from a more socio‐economically advantaged background outperforms a student from an average background by about one year's worth of education in reading, and by even more in comparison to students with low socio‐economic background. Results from PISA also show that some students with low socioeconomic status excel in PISA, demonstrating that overcoming socio‐economic barriers to academic achievement is indeed possible (OECD, 2010).

Smaller class size has been shown to be more beneficial for students from socioeconomically disadvantaged backgrounds (Biddle & Berliner, 2002). Evidence from the Tennessee STAR randomised controlled trial showed that minority students, students living in poverty, and students who were educationally disadvantaged benefitted the most from reduced class size (Finn, 2002; Word et al. (1994). Further, evidence from the controlled, though not randomised, trial, the Wisconsin's Student Achievement Guarantee in Education (SAGE) program, showed that students from minority and low‐income families benefitted the most from reduced class size (Molnar et al., 1999). Thus, rather than implementing costly universal class size reduction policies, it may be more economically efficient to target schools with high concentrations of socioeconomic disadvantaged students for class size reductions.

In the case of the timing of class size reduction, the question is: when does class size reduction have the largest effect? Ehrenberg, Brewer, Gamoran and Willms (2001) hypothesized that students educated in small classes during the early grades may be more likely to develop working habits and learning strategies that enable them to better take advantage of learning opportunities in later grades. According to Bascia and Fredua‐Kwarteng (2008), researchers agree that class size reduction is most effective in the primary grades. That empirical research shows class size to be most effective in the early grades is also concluded by Biddle and Berliner (2002) and the evidence from both STAR and SAGE back this conclusion up (Finn, Gerber, Achilles, & Boyd‐Zaharias, 2001; Smith, Molnar, & Zahorik, 2003). Of course, there is still the possibility that smaller classes may also be advantageous at later strategic points of transition, for example, in the first year of secondary education. Research evidence on this possibility is, however, needed.

For intensity, the question is: how small does a class have to be in order to optimize the advantage? For example, large gains are attainable when class size is below 20 students (Biddle & Berliner, 2002; Finn, 2002) but gains are also attainable if class size is not below 20 students (Angrist & Lavy, 2000; Borland, Howsen & Trawick, 2005; Fredrikson, Öckert & Oosterbeek, 2013; Schanzenbach, 2007). It has been argued that the impact of class size reduction of different sizes and from different baseline class sizes is reasonably stable and more or less linear when measured per student (Angrist & Pischke, 2009, see page 267; Schanzenbach, 2007). Other researchers argue that the effect of class size is not only non‐linear but also non‐monotonic, implying that an optimal class size exists (Borland, Howsen & Trawick, 2005). Thus, the question of whether the size of reduction and initial class size matters for the magnitude of gain from small classes is still an open question.

Finally, researchers agree that the length of the intervention (number of years spent in small classes) is linked with the sustainability of benefits (Biddle & Berliner, 2002; Finn, 2002; Grissmer, 1999; Nye, Hedges & Konstantopoulos, 1999) whereas the evidence on whether more years spent in small classes leads to larger gains in academic achievement is mixed (Biddle & Berliner, 2002; Egelson, Harman, Hood & Achilles, 2002; Finn 2002; Kruger, 1999). How long a student should remain in a small class before eventually returning to a class of regular size is an unanswered question.

### 1.2 THE INTERVENTION

The intervention in this systematic review is a reduction in class size. What constitutes a reduced class size? This seemingly simple issue has confounded the understanding of outcomes of the research and it is one of the reasons there is disagreement about whether class size reduction works (Graue, Hatch, Rao & Oen, 2007).

Two terms are used to describe the intervention, class size and student‐teacher ratio, and it is important to distinguish between these two terms. The first, class size, focuses on reducing group size and, hence, is operationalized as the number of students a teacher instructs in a classroom at a point in time. For this definition, a reduced number of students are assigned to a class in the belief that teachers will then develop an in‐depth understanding of student learning needs through more focused interactions, better assessment, and fewer disciplinary problems. These mechanisms are based on the dynamics of a smaller group (Ehrenberg et al., 2001). The second term is student‐teacher ratio and is often used as a proxy for class size, defined as a school's total student enrollment divided by the number of its full time teachers.

From this perspective, lowering the ratio of students to teachers provides enhanced opportunities for learning. The concept of using student‐teacher ratios as a proxy for class size is based on a view of teachers as units of expertise and is less focused on the student‐teacher relationship. Increasing the relative units of expertise available to students increases learning, but does not rely on particular teacher‐student interactions (Graue et al., 2007).

Although class size and student‐teacher ratio are related, they involve different assumptions about how a reduction changes the opportunities for students and teachers. In addition, the discrepancy between the two can vary depending on teachers' roles and the amount of time teachers spend in the classroom during the school day.

In this review, the intervention is class size reduction. Studies only considering average class size measured as student‐teacher ratio at school level (or higher levels) will not be eligible. Neither will studies where the intervention is the assignment of an extra teacher (or teaching assistants or other adults) to a class be eligible. The assignment of additional teachers (or teaching assistants or other adults) to a classroom is not the same as reducing the size of the class, and this review focuses exclusively on the effects of class size in the sense of number of students in a classroom.

### 1.3 HOW THE INTERVENTION MIGHT WORK

*Smaller classes allow teachers to adapt their instruction to the needs of individual students. For example, teachers' instruction can be more easily adapted to the development of the individual students. The concept of adaptive education refers to instruction that is adapted to meet the individual needs and abilities of students (Houtveen, Booij, de Jong & van de Grift, 1999). With adaptive education, some students receive more time, instruction, or help from the teacher than other students.*

Research has shown that in smaller classes, teachers have more time and opportunity to give individual students the attention they need (Betts & Shkolnik, 1999; Blatchford & Mortimore, 1994; Bourke, 1986; Molnar et al., 1999; Molnar et al., 2000; Smith & Glass, 1980). Additional, less pressure may be placed upon the physical space and resources within the classroom. Both of these factors may be connected to less pupil misbehaviour and disciplinary problems detected in larger classes (Wilson, 2002).

In smaller classes, it is possible for students with low levels of ability to receive more attention from the teacher, with the result that not necessarily all students profit equally. More generally, teachers are able to devote more of their time to educational content (the tasks students must complete) and less to classroom management (for example, maintaining order) in smaller classes. An increased amount of time spend on task, contributes to enhanced academic achievement.

*It has often been pointed out, however, that teachers do not necessarily change the way they teach when faced with smaller classes and therefore do not take advantage of all of the benefits offered by a smaller class size. Research suggests that such situations do indeed exist in practice (e.g.**Blatchford & Mortimore, 1994*; *Shapson, Wright, Eason & Fitzgerald, 1980**)*.

**Figure 1:**
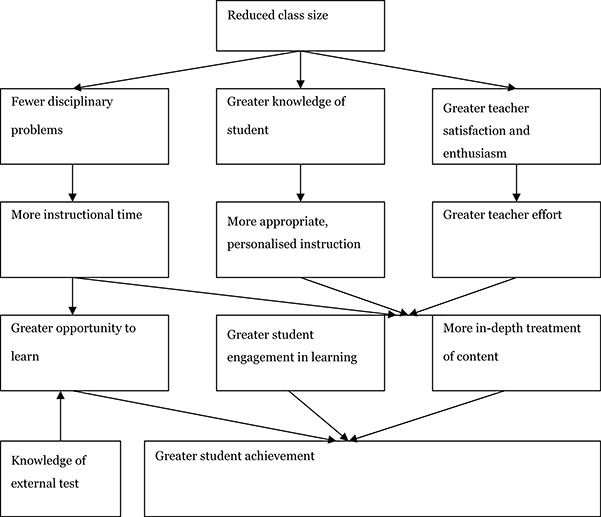
An explanation of the impact of class size on student achievement (Anderson, 2000).

Anderson (2000) addressed the question of why reductions in class size should be expected to enhance student achievement and part of his theory was tested in Annevelink, Bosker and Doolaard (2004). To explain the relationship between class size and achievement, Anderson developed a causal model, which starts with reduced class size and ends with student achievement. Anderson noted that small classes would not, in and of themselves, solve all educational problems. The number of students in a classroom can have only an indirect effect on student achievement. As Zahorik (1999) states: “Class size, of course, cannot influence academic achievement directly. It must first influence what teachers and students do in the classroom before it can possibly affect student learning” (p. 50). In other words, what teachers do matter. Anderson's causal model of the effect of reduced class size on student achievement is depicted in Figure 1.

Anderson's model predicts that a reduced class size will have direct positive effects on the following three variables: 1) Disciplinary problems, 2) Knowledge of student, and 3) Teacher satisfaction and enthusiasm. Each of these variables, in turn, begins a separate path. Fewer disciplinary problems are expected to lead to more instructional time, which in combination with teacher knowledge of the external test, produces greater opportunity to learn. In combination with more appropriate, personalised instruction and greater teacher effort, more instructional time potentially produces greater student engagement in learning as well as more in‐depth treatment of content.

Greater knowledge of students is expected to provide more appropriate personalised instruction, and in combination with more instructional time and greater teacher effort, potentially produces greater student engagement in learning and more in‐depth treatment of content.

Greater teacher satisfaction and enthusiasm are expected to result in greater teacher effort, which in combination with more instructional time and more appropriate, personalised instruction produces greater student engagement in learning and more in‐depth treatment of content.

Finally greater student achievement is the expected result of a combination of the three variables: Greater opportunity to learn, greater student engagement in learning, and more in‐depth treatment of content.

The path from greater knowledge of students through appropriate, personalised instruction and student engagement in learning to student achievement is tested in Annevelink et al. (2004) on students in Grade 1 in 46 Dutch schools in the school year 1999‐2000. Personalised instruction is operationalised as the number of specific types of interactions. Teachers seeking to provide more personalised instruction are expected to provide fewer interactions directed at the organization and personal interactions, and more interactions directed at the task and praising interactions. These changes in interactions are expected to result in a situation where the student spends more time on task.

The level of student engagement is operationalised as the amount of time a student spends on task. Students who spend more time on task are expected to achieve higher learning results.

Smaller classes were related to more interactions of all kinds and more task‐directed and praising interactions resulted in more time spent on task which in turn was related to higher student achievement as expected. Notice that more organizational or personal interactions in smaller classes were contrary to expectations whereas more task‐directed interactions or praising interactions was consistent with expectations (Annevelink et al., 2004).

### 1.4 WHY IT IS IMPORTANT TO DO THE REVIEW

Class size is one of the most researched educational interventions in social science, yet there is no clear consensus on the effectiveness of small class sizes for improving student achievement. While one strand of class size research points to small and insignificant effects of smaller classes, another points to positive and significant effects on student achievement of smaller classes.

The early meta‐analysis by Glass and Smith (1979) analysed the outcomes of 77 studies including 725 comparisons between smaller and larger class sizes on student achievement. They concluded that a class size reduction had a positive effect on student achievement. Hedges and Stock (1983) reanalysed Glass and Smith's data using different statistical methods, but found very little difference in the average effect sizes across the two analysis methods.

However, the updated literature reviews by Hanushek (Hanushek, 1989; 1999; 2003) cast doubt on these findings. His reviews looked at 276 estimates of pupil‐teacher ratios as a proxy for class size, and most of these estimates pointed to insignificant effects. Based on a vote counting method, Hanushek concluded that “there is no strong or consistent relationship between school resources and student performance” (Hanushek, 1989, p. 47). Krueger (2003), however, points out that Hanushek relies too much on a few studies, which reported many estimates from even smaller subsamples of the same dataset. Many of the 276 estimates were from the same dataset but estimated on several smaller subsamples, and these many small sample estimates are more likely to be insignificant. The vote counting method used in Hanushek's original literature review (Hanushek, 1989) is also criticised by Hedges et al. (1994), who offer a reanalysis of the data from Hanushek's reviews using more sophisticated synthesis methods. Hedges et al. (1994) used a combined significance test.^1^ They tested two null hypotheses: 1) no positive relation between the resource and output and 2) no negative relation between the resource and output. The tests determine if the data are consistent with the null hypothesis in all studies or false in at least some of the studies. Further, Hedges et al. (1994) reported the median standardized regression coefficient.^2^ The conclusion is that “it shows systematic positive relations between resource inputs and school outcomes” (Hedges et al., 1994, p. 5). Hence, dependent upon which synthesis method^3^ is considered appropriate; conclusions based on the same evidence are quite different.

The divergent conclusions of the above‐mentioned reviews are further based on non‐experimental evidence, combining measurements from primary studies that have different specifications and assumptions. According to Grissmer (1999), the different specifications and assumptions, as well as the appropriateness of the specifications and assumptions, account for the inconsistency of the results of the primary studies.

The Tennessee STAR experiment provides rare evidence of the effect of class size from a randomized controlled trial (RCT). The STAR experiment was implemented in Tennessee in the 1980s, assigning kindergarten children to either normal sized classes (around 22 students) or small classes (around 15 students). The study ran for four years, until the assigned children reached third grade, but not even based on this kind of evidence do researchers agree about the conclusion.

According to Finn and Achilles (1990), Nye et al. (1999) and Krueger (1999), STAR results show that class size reduction increased student achievement. However, Hanushek (1999; 2003) questions these results because of attrition from the project, crossover between treatments, and selective test taking, which may have violated the initial randomization.

While the class size debate on what can be concluded based on the same evidence is acceptable and meaningful in the research community, it is probably of less help in guiding decision‐makers and practitioners. If research is to inform practice, there must be an attempt to reach some agreement about what the research does and does not tell us about the effectiveness of interventions as well as what conclusions can be reasonably drawn from research. The researchers must reach a better understanding of questions such as: for who does class size reduction have an effect? When does class size reduction have an effect on student achievement? How small does a class have to be in order to be advantageous?

The purpose of this review is to systematically uncover relevant studies in the literature that measure the effects of class size on academic achievement and synthesize the effects in a transparent manner.

## 2 Objectives

The purpose of this review is to systematically uncover relevant studies in the literature that measure the effects of class size on academic achievement. We will synthesize the effects in a transparent manner and, where possible, we will investigate the extent to which the effects differ among different groups of students such as high/low performers, high/low income families, or members of minority/non‐minority groups, and whether timing, intensity, and duration have an impact on the magnitude of the effect.

## 3 Methods

### 3.1 TITLE REGISTRATION AND REVIEW PROTOCOL

The title for this systematic review was approved in The Campbell Collaboration on 9. October 2012. The systematic review protocol was published on March 3, 2015. Both the title registration and the protocol are available in the Campbell Library at:

https://www.campbellcollaboration.org/library/small‐class‐sizes‐student‐achievement‐primary‐and‐secondary‐schools.html

### 3.2 CRITERIA FOR CONSIDERING STUDIES FOR THIS REVIEW

#### 3.2.1 Types of studies

The study designs eligible for inclusion were:

- Controlled trials:
  ○ RCTs ‐ randomized controlled trials
  ○ QRCTs ‐ quasi‐randomized controlled trials where participants are allocated by, for example, alternate allocation, participant's birth date, date, case number or alphabetically
  ○ NRCTs ‐ non‐randomized controlled trials where participants are allocated by other actions controlled by the researcher
- Non‐randomized studies (NRS) where allocation is not controlled by the researcher and two or more groups of participants are compared. Participants are allocated by, for example, time differences, location differences, decision makers, policy rules or participant preferences.

We included study designs that used a well‐defined control group; i.e. the control or comparison condition was students in classes with more students than in the treatment classes.

Non‐randomised studies, where the reduction of class size has occurred in the course of usual decisions outside the researcher's control, must demonstrate pre‐treatment group equivalence via matching, statistical controls, or evidence of equivalence on key risk variables and participant characteristics. These factors are outlined in section 3.4.3 under the subheading of *Confounding*, and the methodological appropriateness of the included studies was assessed according to the risk of bias model outlined in section 3.4.3.

Different studies used different types of data. Some used test score data on individual students and actual class‐size data for each student. Others used individual student data but average class‐size data for students in that grade in each school. Still others used average scores for students in a grade level within a school and average class size for students in that school. We only included studies that used measures of class size and measures of outcome data at the individual or class level. We excluded studies that relied on measures of class size as and measures of outcomes aggregated to a level higher than the class (e.g., school or school district).

Some studies did not have actual class size data and used the average student‐teacher ratio within the school (or at higher levels, e.g. school districts). Studies only considering average class size measured as student‐teacher ratio within a school (or at higher levels) were not eligible.

#### 3.2.2 Types of participants

We included children in grades kindergarten to 12 (or the equivalent in European countries) in general education. Studies that met inclusion criteria were accepted from all countries. We excluded children in home‐school, in pre‐school programs, and in special education.

#### 3.2.3 Types of interventions

The intervention in this review is a reduction in class size, i.e. a comparison of classes with larger and small numbers. The more precise class size is measured the more reliable the findings of a study will be.

Studies only considering the average class size measured as student‐teacher ratio within a school (or at higher levels) were not eligible. Neither were studies where the intervention was the assignment of an extra teacher (or teaching assistants or other adults) to a class eligible. The assignment of additional teachers (or teaching assistants or other adults) to a classroom is not the same as reducing the size of the class, and this review focused exclusively on the effects of reducing class size. We acknowledge that class size can change per subject or eventually vary during the day. The precision of the class size measure was recorded.

#### 3.2.4 Types of outcome measures

##### Primary outcomes

The primary focus was on measures of academic achievement. Academic achievement outcomes included reading and mathematics. Outcome measures had to be standardised measures of academic achievement. The primary outcome variables used in the identified studies were standardised reading and mathematics tests (Stanford Achievement Test (SAT), Item Response Theory‐scaled scores, State wide End‐of‐Grade test (EOG) and NovLex (a lexical database for French elementary‐school readers)).

Studies were only included if they considered one or more of the primary outcomes.

##### Secondary outcomes

We planned to code the following effect sizes as secondary outcomes when available: standardised test in other academic subjects at primary school level (e.g. in science or second language) and measures of global academic performance (e.g. Woodcock‐Johnson III Tests of Achievement, Stanford Achievement Test (SAT), Grade Point Average). None of these secondary outcomes were reported in studies that could be used in the data synthesis.

#### 3.2.5 Duration of follow‐up

All follow‐up durations reported in the primary studies were recorded.

Time points for measures we planned to consider were:

- 0 to 1 year follow up
- 1 to 2 year follow up
- More than 2 year follow up

All studies that could be used in the data synthesis reported outcomes in the short run only; by the end of the school year in which treatment were given.

#### 3.2.6 Types of settings

The location of the intervention was classes, grades kindergarten to 12 (or the equivalent in European countries) in regular private, public or boarding schools were eligible. Home‐schools would have been excluded.

### 3.3 SEARCH METHODS FOR IDENTIFICATION OF STUDIES

#### 3.3.1 Bibliographical database searches

The original electronic searches for this review were performed in 2015. Those searches covered content from 1980‐2015. In February 2017 the searches were updated to cover content from 2015‐2017. The 2017 update had a minor change in the searched electronic resources. These changes are described below. Following electronic databases were searched:

ERIC (EBSCO‐host) ‐ searched from 1980‐2017

SocIndex (EBSCO‐host) ‐ searched from 1980‐2017

EconLit (EBSCO‐host) ‐ searched from 1980‐2017

PsycInfo (EBSCO‐host) ‐ searched from 1980‐2017

Academic Search Premier (EBSCO‐host) ‐ searched from 2015‐2017

Teacher Reference Center (EBSCO‐host) ‐ searched from 2015‐2017

Education Research Complete (EBSCO‐host) ‐ searched from 1980‐2015

International Bibliography of the Social Sciences (ProQuest‐host) ‐ searched from 1980‐2015

ProQuest Dissertations & Theses A&I (ProQuest‐host) ‐ searched from 1980‐2015

Social Science Citation Index (ISI Web of Science) ‐ searched from 1980‐2017

Science Citation Index (ISI Web of Science) ‐ searched from 1980‐2017

#### 3.3.2 Searching other resources

We also searched in other electronic resources for relevant publications:

Campbell Collaboration Library ‐ searched from 1980‐2017

Centre for Reviews and Dissemination Databases ‐ searched from 1980‐2017

EPPI‐Centre Systematic Reviews ‐ Database of Education Research ‐ searched from 1980‐2017

Social Care Online ‐ searched from 1980‐2017

Bibliotek.dk (Danish National Library portal) ‐ searched from 1980‐2015

Bibsys.no (Norwegian National Library portal) ‐ searched from 1980‐2015

Libris.kb.se (Swedish National Library portal) ‐ searched from 1980‐2015

#### 3.3.3 Grey literature search

We searched specific electronic repositories for additional grey literature:

What Works Clearinghouse – U.S. Department of Education ‐ searched from 1980‐2017

EDU.au.dk – Danish Clearinghouse for Education ‐ searched from 1980‐2017

European Educational Research Association ‐ searched from 1980‐2017

American Education Research Association ‐ searched from 1980‐2017

Social Science Research Network ‐ searched from 1980‐2017

Google Scholar ‐ searched from 2015‐2017

#### 3.3.4 Hand search

We hand‐searched following journals for additional references:

*Middle School Journal* – (2014‐2015)

*Elementary School Journal* – (2014‐2015)

*American Educational Research Journal* – (2014‐2015)

*Learning Environments Research* – (2014‐2015)

#### 3.3.5 Search documentation

Selected search strings from the recent search update as well as the resources searched in the 2015 original 2015 search can be found in the Appendix 11.1.

### 3.4 DATA COLLECTION AND ANALYSIS

#### 3.4.1 Selection of studies

Under the supervision of review authors, two review team assistants first independently screened titles and abstracts to exclude studies that were clearly irrelevant. Studies considered eligible by at least one assistant or studies where there was not enough information in the title and abstract to judge eligibility, were retrieved in full text. The full texts were then screened independently by two review team assistants under the supervision of the review authors. Any disagreements of eligibility were resolved by the review authors. Exclusion reasons for studies that otherwise might be expected to be eligible were documented and presented in the appendix.

The study inclusion criteria were piloted by the review authors (see Appendix 11.3). The overall search and screening process was illustrated in a flow‐diagram. None of the review authors were blind to the authors, institutions, or the journals responsible for the publication of the articles.

#### 3.4.2 Data extraction and management

Two review authors independently coded and extracted data from included studies. A coding sheet was piloted on several studies and no revision was necessary (see Appendix 11.4). Disagreements were minor and were resolved by discussion. Data and information was extracted on: Available characteristics of participants, intervention characteristics and control conditions, research design, sample size, risk of bias and potential confounding factors, outcomes, and results. Extracted data was stored electronically. Analysis was conducted in RevMan5 and Stata.

#### 3.4.3 Assessment of risk of bias in included studies

We assessed the methodological quality of studies using a risk of bias model developed by Prof. Barnaby Reeves in association with the Cochrane Non‐Randomised Studies Methods Group.^4^ This model is an extension of the Cochrane Collaboration's risk of bias tool and covers risk of bias in non‐randomised studies that have a well‐defined control group.

The extended model is organised and follows the same steps as the risk of bias model according to the 2008‐version of the Cochrane Hand book, chapter 8 (Higgins & Green, 2008). The extension to the model is explained in the three following points:

1) The extended model specifically incorporates a formalised and structured approach for the assessment of selection bias in non‐randomised studies by adding an explicit item about confounding. This is based on a list of confounders considered to be important and defined in the protocol for the review. The assessment of confounding is made using a worksheet where, for each confounder, it is marked whether the confounder was considered by the researchers, the precision with which it was measured, the imbalance between groups, and the care with which adjustment was carried out (see Appendix 11.5). This assessment will inform the final risk of bias score for confounding.
2) Another feature of non‐randomised studies that make them at high risk of bias is that they need not have a protocol in advance of starting the recruitment process. The item concerning selective reporting therefore also requires assessment of the extent to which analyses (and potentially, other choices) could have been manipulated to bias the findings reported, e.g., choice of method of model fitting, potential confounders considered / included. In addition, the model includes two separate yes/no items asking reviewers whether they think the researchers had a pre‐specified protocol and analysis plan.
3) Finally, the risk of bias assessment is refined, making it possible to discriminate between studies with varying degrees of risk. This refinement is achieved with the addition of a 5‐point scale for certain items (see the following section, *Risk of bias judgement items* for details).

The refined assessment is pertinent when thinking of data synthesis as it operationalizes the identification of studies (especially in relation to non‐randomised studies) with a very high risk of bias. The refinement increases transparency in assessment judgements and provides justification for not including a study with a very high risk of bias in the meta‐analysis.

##### Risk of bias judgement items

The risk of bias model used in this review is based on nine items (see Appendix 11.5). The nine items refer to:

- **sequence generation** (Judged on a low/high risk/unclear scale)
- **allocation concealment** (Judged on a low/high risk/unclear scale)
- **confounders** (Judged on a 5 point scale/unclear)
- **blinding** (Judged on a 5 point scale/unclear)
- **incomplete outcome data** (Judged on a 5 point scale/unclear)
- **selective outcome reporting** (Judged on a 5 point scale/unclear)
- **other potential threats to validity** (Judged on a 5 point scale/unclear)
- **a priori protocol** (Judged on a yes/no/unclear scale)
- **a priori analysis plan** (Judged on a yes/no/unclear scale)

In the 5‐point scale, 1 corresponds to Low risk of bias and 5 corresponds to High risk of bias. A score of 5 on any of the items assessed on the 5‐point scale translates to a risk of bias so high that the findings will not be considered in the data synthesis (because they are more likely to mislead than inform).

##### Confounding

An important part of the risk of bias assessment of non‐randomised studies is how the studies deal with confounding factors (see Appendix 11.5). Selection bias is understood as systematic baseline differences between groups and can therefore compromise comparability between groups. Baseline differences can be observable (e.g. age and gender) and unobservable (to the researcher; e.g. motivation). There is no single non‐randomised study design that always deals adequately with the selection problem: Different designs represent different approaches to dealing with selection problems under different assumptions and require different types of data. There can be particularly great variations in how different designs deal with selection on unobservables. The “adequate” method depends on the model generating participation, i.e. assumptions about the nature of the process by which participants are selected into a program. A major difficulty in estimating causal effects of class size on student outcomes is the potential endogeneity of class size, stemming from the processes that match students with teachers, and schools. Not only do families choose neighbourhoods and schools, but principals and other administrators assign students to classrooms. Because these decision makers utilize information on students, teachers and schools, information that is often not available to researchers, the estimators are quite susceptible to biases from a number of sources.

The primary studies must at least demonstrate pre‐treatment group equivalence via matching, statistical controls, or evidence of equivalence on key risk variables and participant characteristics. For this review, we identified the following observable confounding factors to be most relevant: age and grade level, performance at baseline, gender, socioeconomic background and local education spending. In each study, we assessed whether these confounding factors had been considered, and in addition we assessed other confounding factors considered in the individual studies. Furthermore, we assessed how each study dealt with unobservables.

##### Importance of pre‐specified confounding factors

The motivation for focusing on age and grade level, performance at baseline, gender, socioeconomic background and local education spending is given below.

Generally development of cognitive functions relating to school performance and learning are age dependent, and furthermore systematic differences in performance level often refer to systematic differences in preconditions for further development and learning of both cognitive and social character (Piaget, 2001; Vygotsky, 1978).

Therefore, to be sure that an effect estimate is a result from a comparison of groups with no systematic baseline differences it is important to control for the students' grade level (or age) and their performance at baseline (e.g. reading level, mathematics level).

With respect to gender it is well‐known that there exist gender differences in school performance (Holmlund & Sund, 2005). Girls outperform boys with respect to reading and boys outperform boys with respect to mathematics (Stoet & Geary, 2013). Although part of the literature finds that these gender differences have vanished over time (Hyde, Fennema, & Lamon, 1990; Hyde & Linn, 1988), we find it important to include this potential confounder.

Students from more advantaged socioeconomic backgrounds on average begin school better prepared to learn and receive greater support from their parents during their schooling years (Ehrenberg et al., 2001). Further, there is evidence that class size may be negatively correlated with the student's socioeconomic backgrounds. For example, in a study of over 1,000 primary schools in Latin America, Willms and Somers (2001) found that the correlation between the pupil/teacher ratio in the school and the socioeconomic level of students in the school was about –.15. Moreover, Willms and Somers (2001) found that schools enrolling students from higher socioeconomic backgrounds tended to have better infrastructures, more instructional materials, and better libraries. The correlations of these variables with school‐level socioeconomic status varied between .26 and .36.

Finally, as outlined in the background section, students with socio‐economically disadvantaged backgrounds perform poorly in school tests (OECD, 2010).

Therefore, the accuracy of the estimated effects of class size will depend crucially on how well socioeconomic background is controlled for. Socioeconomic background factors are, e.g. parents' educational level, family income, minority background, etc.

#### 3.4.4 Measures of treatment effect

For continuous outcomes, effects sizes with 95% confidence intervals were calculated using means and standard deviations where available, or alternatively from mean differences, standard errors and 95% confidence intervals (whichever were available), using the methods suggested by Lipsey & Wilson (2001). Hedges' *g* was used for estimating standardised mean differences (SMD).

Software for storing data and statistical analyses were Excel and RevMan 5.0.

#### 3.4.5 Unit of analysis issues

To account for possible statistical dependencies, we examined a number of issues: we assessed whether suitable cluster analysis was used (e.g. cluster summary statistics, robust standard errors, the use of the design effect to adjust standard errors, multilevel models and mixture models), if assignment of units to treatment was clustered, whether individuals had undergone multiple interventions, whether there were multiple treatment groups, and whether several studies were based on the same data source.

##### Cluster assignment to treatment

We checked for consistency in the unit of allocation and the unit of analysis, as statistical analysis errors can occur when they are different. In cases where study investigators had not applied appropriate analysis methods that control for clustering effects, we estimated the intra‐cluster correlation (Donner, Piaggio, & Villar, 2001) and corrected the effect size and standard error. Based on the analysis in Stockford (2009), we used an intra‐cluster correlation () of 0.22. We report the corrected results and the non‐corrected results. We used the following formulas (see Hedges, 2007, page 349):

$$\begin{array}{c} d=\left(\frac{MD}{SD}\right)\sqrt{1-\frac{2(n-1)\rho }{N-2}}\\ SE=\sqrt{\left(\frac{N^{T}+N^{C}}{N^{T}N^{C}}\right)(1+(n-1)\rho )+d^{2}\left(\frac{\left(N-2){\left(1-\rho \right)}^{2}+n(N-2n)\rho^{2}+2(N-2n)\rho (1-\rho \right)}{2(N-2)[(N-2)-2(n-1)\rho ]}\right)} \end{array}$$

where *n* is cluster size and *N^T^*, *N^C^* are treatment and control group sample sizes and *N* is total sample size.

##### Multiple Interventions per Individual

There were no studies with multiple interventions per individual.

##### Multiple Studies using the Same Sample of Data

Five studies analysed the same population, using data from the Third International Mathematics and Science Study (TIMSS) data set from 1995. Three studies used TIMMS data from 2011. Data from the National Educational Longitudinal Study (NELS data from USA) was used in five studies.

Two studies analysed the same US population using the Early Childhood Longitudinal Study‐Kindergarten Class of 1998‐1999 data set. Five studies analysed data from Indiana's Prime Time Project (1984‐1988). Five studies analysed the Student Achievement Guarantee in Education Program (SAGE) implemented in Wisconsin in 1996‐2001. Three studies analysed the same sample of students from Israel. Four studies analysed the same population using the PRIMA survey which contains information on Dutch pupils who were enrolled in grades 2, 4, 6 and 8 in the school‐year 1994/95. Two studies used the same sample of Swedish students from 1998 to 1999. Finally, four studies analysed the British Class Size Study (1996‐1999). We reviewed all studies, but in the meta‐analysis we only included one estimate of the effect from each sample of data in order to avoid dependencies between the “observations” (i.e. the estimates of the effect) in the meta‐analysis. The choice of which estimates to include was based on our risk of bias assessment of the studies. We chose the estimate from each sample of data from the study that we judged to have the least risk of bias due to confounding.

One RCT (the STAR experiment conducted in Tennessee in 1985–1989) was reported in several studies (45 studies reported in 51 papers). We reviewed all studies but it was unclear which study should be judged to have the least risk of bias. We reported all relevant results from the studies analysing STAR but none of the studies were included in the meta‐analysis of non‐STAR studies.

##### Multiple Time Points

All studies that could be used in the data synthesis reported outcomes in the short run only.

#### 3.4.6 Dealing with missing data

Where studies had missing summary data, such as missing standard deviations, we calculated SMDs from mean differences, standard errors and 95% confidence intervals (whichever were available), using the methods suggested by Lipsey & Wilson (2001). We requested information from the principal investigators (if current contact information could be located) if not enough information was provided to calculate an effect size and standard error.

#### 3.4.7 Assessment of heterogeneity

Heterogeneity among primary outcome studies was assessed with the Chi‐squared (Q) test, and the I‐squared, and τ‐squared statistics (Higgins, Thompson, Deeks, & Altman, 2003). Any interpretation of the Chi‐squared test was made cautiously on account of its low statistical power.

#### 3.4.8 Data synthesis

All studies that could be used in the data synthesis reported outcomes in the short run only; by the end of the school year in which treatment were given. We carried out our meta‐analyses using the standardised mean differences (SMD). All analyses were inverse variance weighted using random effects statistical models that incorporate both the sampling variance and between study variance components into the study level weights. Random effects weighted mean effect sizes were calculated using 95% confidence intervals.

#### 3.4.9 Sensitivity analysis

Sensitivity analysis was used to evaluate whether the pooled effect sizes were robust across components of methodological quality.

For methodological quality, we performed sensitivity analysis for study design and the confounding item of the risk of bias checklists, respectively. Sensitivity analysis was further used to examine the robustness of conclusions in relation to inclusion of a result with an unclear sign, inclusion of effect sizes from the STAR experiment and to multiplying the reported effect with a standard deviation reduction in class size in the studies using class size as a continuous variable.

## 4 Results

### 4.1 DESCRIPTION OF STUDIES

#### 4.1.1 Results of the search

The search was performed between 2015 and February 2017.

The results are summarised in Figure 1 in section 11.2. The total number of potential relevant records was 8,128 after excluding duplicates (database: 7,434, grey, hand search, snowballing and other resources: 694). All 8,128 records were screened based on title and abstract; 7754 were excluded for not fulfilling the first level screening criteria and 374 records were ordered for retrieval and screened in full text. Of these, 226 did not fulfil the second level screening criteria and were excluded. Eighteen records were unobtainable despite efforts to locate them through libraries and searches on the internet. The references are listed in section 8.3.

A total of 127 unique studies, reported in 148 papers were included in the review. Further details of the included and excluded studies are provided in section 10.

#### 4.1.2 Included studies

The search resulted in a final selection of 127 studies, reported in 148 papers, which met the inclusion criteria for this review. The 127 studies analysed 55 different populations. A large number of studies (45) analysed data from the STAR experiment (class size reduction in grade K‐3) and its follow up data.

Of the 82 studies not analysing data from the STAR experiment, only six could be used in the data synthesis. Fifty eight studies could not be used in the data synthesis as they were judged to have too high risk of bias on either the confounding item (51), for the other bias item (4) or for the selective reporting item (3). Eighteen studies did not provide enough information enabling us to calculate an effects size and standard error or did not provide results in a form enabling us to use it in the data synthesis.

##### 4.1.2.1 STAR studies

A large number of studies analysed data from the STAR experiment (class size reduction in grade K‐3) and its follow up data^5^, 45 studies reported in 51 papers.

The four‐year STAR experiment was conducted in Tennessee in 1985–1989, to assess the effectiveness of small classes compared with regular‐sized classes and of teachers' aides in regular‐sized classes on improving cognitive achievement in kindergarten and in the first, second, and third grades. According to the Technical report (Word, 1994) and Word et al. (1990), ^6^ the goal of the STAR experiment was to have approximately 100 small classes with 13‐17 students (S), 100 regular classes with 22‐25 students (R), and 100 regular with aide classes with 22‐25 students (RA). In Word et al. (1994) it is reported that in the 1985‐86 year (the first year of the experiment), the STAR project had 128 small classes (approximately 1,900 students), 101 regular classes, (approximately 2,300 students), and 99 regular classes with teacher aides (approximately 2,200 students). Both students and teachers were randomised and randomisation was done within schools so at least one of each class type (S, R and RA) was present at each school. Every class was to remain the same type for four years and a new teacher was randomly assigned to each class in each subsequent grade.

Four studies provided results for grade K‐3, that could be used in the data synthesis. The first study, by Folger and Breda (1989), provided effect sizes comparing small classes to regular classes for each grade level (K‐3). The results of the analysis conducted by Folger and reported in Folger and Breda (1989) was also reported in Word et al. (1990) and Word et al. (1994). Both reports by Word et al. provide a summary of original results from the primary analyses of the STAR experiment. The primary analyses were analysis‐of‐variance models conducted by Professor Finn. However, only a summary of the analyses showing significance levels (.05, .01, .001, all levels are only reported as < = and not the exact level of significance) are reported (which cannot be used in the data synthesis). The second study, by Finn, Gerber, Achilles and Boyd‐Zaharias (2001), provided effect sizes comparing small classes to regular classes for each grade level (K‐3) but used different decision rules in selecting a sample for analysis than in Folger and Breda (1989). In addition Finn et al. (2001) included covariates in the analysis. The third study, by Nye, Achilles, Boyd‐Zaharias, Fulton & Wallenhorst (1992/1994) (Nye, Achilles, Boyd‐Zaharias, Fulton & Wallenhorst (1994) is a published and shorter version of the 1992 paper), provided effect sizes comparing small classes to the average of regular and regular with aide classes and other than the different comparison they also used different decision rules in selecting a sample for analysis than in Folger & Breda (1989). The effect sizes from the analysis in Nye et al. (1992/1994) are also reported in Finn (1998), Finn & Achilles (1999) and Nye, Achilles, Boyd‐Zaharias & Fulton (1993). Finally, effect sizes comparing small classes to the average of regular and regular with aide classes were also provided in the study by Hanushek (1999).

Which of these four studies's effect estimates should be included in the data synthesis is not obvious as the decision rule as described in the protocol cannot be used (all studies analysing the same RCT).

The four studies differed in terms of both the chosen comparison condition and decision rules in selecting a sample for analysis (see table 4.1) and which one should be judged to have the least risk of bias is not obvious. Below we describe the different posibilities of chosing a comparison and selecting a sample for analysis.

**Table 4.1:** Characteristics of studies analysing STAR data used in the data synthesis

	**Folger, 1989**	**Nye, 1992/994**	**Finn, 2001**	**Hanushek, 1999**
Comparison	R	R + RA	R	R + RA
Size of R and RA classes used	21‐28	22‐26	22‐26	22‐25
Out‐of range classes	Excluded	Included	Not reported	Not reported
STAR trained teachers	Included	Excluded	Not reported	Not reported
Regression with covariate adjustment	No	No	Yes	No
Intention to treat/treatment as received	Not reported	Not reported	Not reported	Not reported

The numbers of S, R and RA classes and students, as reported in the Technical report (Word, 1994) and Word et al. (1990), are probably the number of students and classes that initially were randomised to any of the three conditions (S, R and RA). However, a considerably proportion of classes did not fall into the range they were intended to. According to the STAR Database User's Guide (Finn et al.,2007, using a table of the distribution of classes by grade and designation reported in Achilles, 1999) between 18 and 32 per cent of classes each year was ‘out of range’; falling in the range of either 18‐21 students or 26‐30 students (see section 10.3 for details). In addition a total of 14 regular and regular with aide classes fell in the range of small classes throughout one of the four years but were not considered out of range according to Finn et al. 2007. The four studies providing effect estimates of the STAR experiment either excluded, included or did not report how they handled the out of range classes. In addition the range of regular sized classes used in the four studies differed, only one study used the range 22‐25 (see table 4.1).

In 2. Grade a number of schools and teachers were randomly chosen to receive special STAR training. A second choice of selection of analysis sample concerns whether to include or exclude the classes whose teachers received STAR training and in addition it is unclear how many actually received training. According to Word et al. (1990) and Folger and Breda (1989), 57 teachers in grade 2 from 13 randomly chosen schools and another 57 teachers in grade 3 received Project STAR training. According to Word et al. (1994) p. 73, 67 teachers received training in grade 2 and on page 117 it is stated that all teachers (57 teachers and 57 classes) from 13 schools received training in 2. Grade and all teachers from the same 13 schools (57 classes) received training in 3. Grade. According to Finn et al. (2007) the training was given to 54 second grade teachers from 15 STAR schools. The four studies either excluded, included or did not report how they handled these classes (see table 4.1).

The four studies also differed in the comparison condition they chose. They either compared small classes to regular classes only or to the average of regular and regular with aide classes. Which comparison is most appropriate for this review is however not obvious. At the beginning of 1. Grade approximately half of the students in regular and regular with aide classes interchanged classes (see section 10.3 for details). At the beginning of 2. Grade (3. Grade) 6 (5) per cent of the students in regular and regular with aide classes interchanged classes. Which choice of comparison is appropriate concerning the analysis for grades 1‐3 is thus unclear.

In addition to the regular and regular with aide class interchanging; each year students from small classes moved to regular or regular‐with‐aide classes and students from regular and regular with aide classes moved to small classes (6, 4 and 4 per cent at the beginning of 1. 2. and 3. Grade). In total 25 per cent of all students moved class type at some point. Whether all of these students actually moved classes or a part of the reported movement of students between classes were due to reclassification of class type (small or regular sized) is unclear. The reported number of students moving to and from classes with aide cannot be due to reclassification between small and regular sized classes. At least some reclassification must have occurred though as the following two pieces of evidence show: First, according to the numbers reported in the Technical report (Word et al., 1994), the distribution of class type was not constant in the 13 schools randomly chosen to receive STAR training. It is reported there are 21 small classes, 19 regular classes and 17 regular with aide classes in these schools in 2. Grade. In 3. Grade it is reported there are 25 small, 15 regular and 17 regular with aide classes in the same 13 schools. Thus four classes are apparently reclassified from regular sized to small even though classes were to remain the same type for four years. Second, according to the Technical report (Word et al., 1994) two schools in 3. Grade had incomplete test data and were removed. Compared to the total number of classes in 2. Grade however, only the number of regular classes is reduced from second to third grade (with 11). Some classes must have been reclassified as randomisation was done within schools so each school had at least one class of each type (S, R and RA). None of the four studies providing effect estimates, were explicit about how they handled this moving around of students (and classes).

The four studies are characterised concerning comparison, selection of sample for analysis and method of estimation in table 4.1. Only the study by Hanushek (1999) used the range 22‐25 for regular sized classes. The study compares small classes to the average of regular and regular with aide classes and otherwise nothing is reported concerning sample selection (out of range classes and STAR trained teachers) nor how the treatment was defined (as received or intended). The study by Folger and Breda (1989) compares small classes to regular classes only but uses a range of 21‐28 students for regular classes. It is reported that STAR trained teachers and their classes are included and out of range classes are excluded but it is not reported how out of range classes are defined (for example are the 14 regular and regular with aide classes that fell in the range of small classes excluded and is the definition of out of range classes different than that reported in Finn et al., 2007, considering the different range og regular classes?). The study by Nye et al. (1992/1994) compares small classes to the average of regular and regular with aide classes and uses a range of 22‐26 students for regular sized classes. It is reported that STAR trained teachers and their classes are excluded and out of range classes are included but it is not reported how out of range classes are categorised (for example are the 14 regular and regular with aide classes that fell in the range of small classes considered small and are the classes in the range 18‐21 categorised as small or regular?). The study by Finn et al. (2001) compares small classes to regular classes only and uses a range of 22‐26 students for regular classes. Otherwise nothing is reported concerning sample selection (out of range classes and STAR trained teachers) or how the treatment was defined (as received or intended). The study includes covariates in the analysis.

We find it very difficult to decide which study or effect estimate is the ‘right’ one to include in the data synthesis. Contrary to usual practice we will therefore not chose one study to include in the data synthesis but will report the results of all four studies in section 4.3 and further examine the robustness of our conclusions when including the extremes (smallest and largest) of the range of effect sizes from the STAR experiment in section 4.3.5.

Concerning the follow up study of the STAR experiment (known as the Lasting benefits study, LBS) a technical report providing effect estimates concerning grade 4, 5, 6, 7 and 8 was published each year. However, only one of the technical reports could be located (Nye et al., 1992, reporting results for grade 5). The remaining technical reports (Nye et al., 1991, 1993, 1994 and 1995) for grade 4, 6, 7 and 8 were unobtainable. The results for grade 4 are however reported in Finn & Achilles (1989). In addition the effect sizes from the technical reports for grade 4 and 5 are also reported in Nye, Achilles, Zaharias & Fulton (1993), Achilles, Nye, Zaharias & Fulton (1993) and Finn & Achilles (1999). Finn & Achilles (1999) also report the effect sizes from the technical reports for grade 6 and 7. The effect sizes from the technical report for grade 8 could not be located. Finn, Gerber, Achilles & Boyd‐Zaharias (2001) report effect sizes for grade 8 (and grade 4 and 6) in a reanalysis of the follow up data. None of these studies reporting results using follow up data from the STAR experiment could however be used in the data synthesis due to too high risk of bias (see section 4.2).

Several other studies reported results from a variety of re‐analyses of the STAR experiment (and follow up data) but none of them could be used in the data synthesis. An overview of the reasons for exclusions from the data synthesis is given in section 10.2.

##### 4.1.2.2 Non‐STAR studies

Of the 82 studies (reported in 97 papers) not analysing data from the STAR experiment (or follow up data), only six could be used in the data synthesis.

Five studies (West & Wößmann, 2006; Wößmann & West, 2006; Wößmann, 2003; Wößmann, 2005b and Pong & Pallas, 2001) analysed the same population, using data from the Third International Mathematics and Science Study (TIMSS) data set from 1995. None of these studies were used in the data synthesis as all five studies were judged to have a score of 5 on the risk of bias scale for the confounding item. Three studies used TIMMS data from 2011 (Konstantopoulos & Li, 2016; Li & Konstantopoulos, 2017 and Li, 2015). All three studies were judged 5 on the confounding item and were not included in the analysis. Data from the National Educational Longitudinal Study (NELS data from USA) was used in five studies (Akerhielm, 1995; Boozer and Rouse, 2001; Dee & West, 2011; Hudson, 2011 and Maasoumi, Millimet & Rangaprasad, 2005). The studies by Boozer and Rouse (2001) and Akerhielm (1995) were judged to have a score of 5 on the risk of bias scale for the confounding item and were excluded from the data synthesis. The studies by Dee and West (2011) and Maasoumi, Millimet and Rangaprasad (2005) did not provide results we could use in the data synthesis (results were reported as differences between subjects and first or second order stochastic dominance tests respectively). The study by Hudson (2011) was used in the data synthesis.

Two studies (Milesi & Gamoran, 2006 and Wenfan & Qiuyun, 2005) analysed the same US population using the Early Childhood Longitudinal Study‐Kindergarten Class of 1998‐1999 data set. The study by Wenfan and Qiuyun (2005) was judged to have a too high risk of bias (scored 5 on the confounding item) and was excluded from the data synthesis. The study by Milesi and Gamoran (2006) was used in the data synthesis. Five studies analysed data from Indiana's Prime Time Project (1984‐1988) (Gilman, 1988; Gilman, Swan & Stone, 1988; McGiverin, 1989; Sanogo & Gilman, 1994 and Tillitsky et al., 1988). The four studies by Gilman (1988), Gilman et al. (1988), McGiverin (1989) and Tillitsky et al., (1988) were all rated 5 on the risk of bias scale and the study by Sanogo and Gilman (1994) did not provide results we could use in the data synthesis (do not report what type of classes are included). Five studies analysed the Student Achievement Guarantee in Education Program (SAGE) implemented in Wisconsin in 1996‐2001 (Maier et al., 1997; Molnar, Smith & Zahorik, 1997; Molnar, Smith & Zahorik, 1998; Molnar et al., 1999 and Molnar et al., 2001). None of the studies provided results that could be used in the data synthesis (for details see section 10.1). Three studies analysed the same sample of students from Israel (Angrist & Lavy, 1999; Lavy, 2001 and Otsu, Xu & Matsushita, 2015). The two studies by Angrist and Lavy (1999) and Lavy (2001) were both judged to have a too high risk of bias (scored 5 on the confounding item) and in the study by Otsu et al. (2015) relevant results were presented graphically and no effect sizes or standard errors could be extracted.

Four studies (Dobbelsteen, Levin & Oosterbeek, 2002; Levin, 2001; Ma & Koenker, 2006 and Gerritsen, Plug & Webbink, 2017) analysed the same population using the PRIMA survey which contains information on Dutch pupils who were enrolled in grades 2, 4, 6 and 8 in the school‐year 1994/95. Three studies (Dobbelsteen et al., 2002; Levin, 2001 and Ma & Koenker, 2006) were however judged to have a too high risk of bias (scored 5 on the confounding item) and were excluded from the data synthesis. The study by Gerritsen et al. (2017) was used in the data synthesis. Another two studies (Krueger & Lindahl, 2002 and Lindahl, 2005) used the same sample of Swedish students from 1998 to 1999. Both were judged to have a too high risk of bias (scored 5 on the confounding item).

Finally, four studies analysed the British Class Size Study (1996‐1999) (Blatchford & Basset, 2003; Blatchford, Bassett, Goldstein & Martin, 2003; Blatchford, Goldstein, Martin & Browne, 2002 and Carpenter, Goldstein & Rasbash, 2003). Blatchford et al., 2002 and Carpenter et al., 2003 were both judged to have a too high risk of bias (scored 5 on the selective reporting item) and were excluded from the data synthesis. Neither the study by Blatchford and Basset (2003) nor the study by Blatchford et al. (2003) provided information that enabled us to calculate an effect size and standard error (see section 10.1 for details).

In Table 4.2 we show the total number of studies, not analysing the STAR experiment that met the inclusion criteria for this review. The first column shows the total number of studies grouped by country of origin. The second column shows the number of these studies that did not provide enough data to calculate an effect estimate. The third column gives the number of studies that were coded with very high risk of bias. The fourth column gives the number of studies that were excluded from the data synthesis due to overlapping samples. The last column gives the total number of studies used in the data synthesis.

**Table 4.2:** Number of Included Studies, Not Using STAR Data

			**Reduction due to**		
**Country**	**Total**	**Missing data**	**Too high risk of bias**	**Used same data sets**	**Used in data synthesis**
**Australia**	1	‐	1	‐	0
**Bolivia**	1	‐	1	‐	0
**Canada**	1	1	‐	‐	0
**Columbia**	1	‐	1	‐	0
**Cypres**	1	‐	1	‐	0
**Denmark**	1	‐	1	‐	0
**France**	3	1	‐	‐	2
**Germany**	1	1	‐	‐	0
**Greece**	1	‐	1	‐	0
**Hong Kong**	1	‐	1	‐	0
**Israel**	3	1	2	‐	0
**Italy**	1	‐	1	‐	0
**Japan**	3	‐	3	‐	0
**Lesotho**	1	‐	1	‐	0
**Multiple** ^1^	8	‐	8	‐	0
**New Zealand**	1	‐	1	‐	0
**NL**	5	‐	4	‐	1
**Norway**	2	‐	2	‐	0
**Poland**	1	‐	1	‐	0
**Sri Lanka**	1	‐	1	‐	0
**Sweden**	2	‐	2	‐	0
**UK**	5	2	3	‐	0
**USA**	37	12	22	‐	3
**Total**	82	18	58	0	6

*Note: The reduction due to too high risk of bias preceded the reduction due to using same data set.*

1 *The countries included in these eight studies are: Australia, Belgium, Canada, Croatia, Czech Republic, Denmark, England, France, Germany, Greece, Hong Kong, Hungary, Iceland, Ireland, Italy, Japan, Korea, Lithuania, Malta, Norway, Portugal, Romania, Scotland, Singapore, Slovak Republic, Slovenia, Spain, Sweden, the Netherlands, USA, Chinese Taipei, Scotland and Switzerland.*

Fifty‐eight studies were judged to have a score of 5 on the risk of bias scale for either the confounding item (51), for the other bias item (4) or for the selective reporting item (3) (see a supplementary document for the detailed risk of bias assessments). In accordance with the protocol, we excluded these studies from the data synthesis on the basis that they would be more likely to mislead than inform. Eighteen studies did not provide enough information enabling us to calculate an effects size and standard error or did not provide results in a form enabling us to use it in the data synthesis. All studies (those not analysing STAR data) are listed in table 10.1 in section 10.1 along with the reason if the study is not used in the data synthesis.

The main characteristics of the six studies (not analysing STAR) used in the data synthesis are shown in table 4.3.

**Table 4.3:** Characteristics of Studies Used in the Data Synthesis

Study	Bressoux, 2009	Ecalle, 2006	Gerritsen, 2017
Country	France	France	Netherlands
Time period	1991‐1992	2002‐2003	1994‐1995
Grade	3	1	2
Study design	NRS	RCT	NRS
Class size	Mean (SD): 22.9 (4.3)	S: 10‐12, R: 20‐25	Mean (SD): 24.07 (4.5)
Number of students	Total 1,680	S: 570; R: 622	Total 470
Number of classes	Total 100	S: 100; R: 100	NR
**Study**	**Hudson, 2011**	**Milesi, 2006**	**Munoz, 2001**
**Country**	USA	USA	USA
**Time period**	1990	1998‐1999	1999‐2000
**Grade**	10	KG	3
**Study design**	NRS	NRS	NRS
**Class size**	Mean (SD): Reading: 22.61 (6.3); Mathematics: 23.37 (7.1)	S: less than 18, R: 18‐23, L: more than 23	S: less than 19, L: more than 18 (‘usual’ size is 24)
**Number of students**	NR	Total 11,567	S: 47; L: 57
**Number of classes**	NR	Total 2,437	NR

*Note: KG: Kindergarten, S: Small classes, R: Regular classes, L: Large classes, NR: Not reported, NRS: Nonrandomised study, RCT Randomised controlled trial*

The studies used in the data synthesis were from USA, the Netherlands and France, one was a RCT and five were NRS. None of the studies were conducted recently, the oldest, used data from 1990 and the earliest was conducted in the beginning of 2000. The grades investigated spanned kindergarten to 3. Grade and one study investigated grade 10. The sample sizes varied; the smallest study investigated 104 students and the largest study investigated 11,567 students. The class size reductions analysed varied from a minimum of one student in four studies, a minimum of seven students in another study (small classes less than 18 students and large classes more than 23 students) to a minimum of 8 students in the last study (small classes 10‐12,students and large 20‐25 students).

#### 4.1.3 Excluded studies

In addition to the 127 studies that met the inclusion criteria for this review, 38 studies (reported in 50 papers) at first sight appeared relevant but did not meet our criteria for inclusion. The studies and reasons for exclusion are given in a supplementary document.

### 4.2 RISK OF BIAS IN INCLUDED STUDIES

The risk of bias coding for each of the 127 studies is shown in a supplementary document.

#### 4.2.1 STAR studies

Forty‐five studies analysed data from the STAR experiment and its follow up data. Both children and teachers were randomly allocated within schools to the three types of classes but the method is not described. All studies analysing the STAR experiment were judged Unclear on the sequence generation item and Low risk of bias on the allocation concealment item (as the allocation was non‐sequential) with the exception of one study (Harvey, 1994) which analysed a subgroup (the subgroup is retainees, i.e. selected on a potential outcome variable).

Only four studies provided results for grade K‐3, that can be used in the data synthesis. In addition three other studies provided results that can be used in the data synthesis but analyse only one grade (K or 1). Seven studies reported results from one or more of the five studies that can be used in the data synthesis. Seventeen studies provided no results that can be used in the data synthesis. Eleven studies analysed STAR follow up data (known as the Lasting Benefits Study LBS) and were all given a score of 5 on the Other risk of bias item corresponding to a risk of bias so high that the findings should not be considered in the data synthesis. Another three studies (analysing STAR data, not the follow up) were given a score of 5 on the Incomplete outcome data item (one study) and the Other risk of bias item (two studies).

**Table 4.4:** Risk of Bias ‐ Distribution of the 45 Studies Analysing STAR Data

**Relevant results reported are from other included studies**	7
**STAR follow up data (LBS)**	11
**Provide no results that can be used in the data synthesis**	17
**Used in data synthesis**	4
**Provide results for only one grade (K or 1) that can be used**	3
**Too high risk of bias**	3
**Total**	45

#### 4.2.2 Non‐STAR studies

Concerning studies that did not analyse STAR (or follow up) data, all studies, except two, used non‐randomised designs, they were all judged to have a high risk of bias on the sequence generation item and the allocation concealment item. The two studies using randomised designs did not report the method of randomisation and were judged unclear on the sequence generation and allocation concealment items. All studies were judged 4 on the blinding item. None of the studies had an *a priori* protocol or an *a priori* analysis plan.

A summary of the risk of bias associated with confounding, incomplete data, other bias and selective reporting for the 64 studies from which it was possible to extract an effect estimate is shown in Table 4.5. Fifty one studies were given a score of 5 on the confounding item, corresponding to a risk of bias so high that the findings should not be considered in the data synthesis. For these 51 studies, we did not find it relevant to judge on the remaining items because of their already high risk of bias. Of the remaining 13 studies, four were given a score of 5 on the Other risk of bias item and three were given a score of 5 on the Selective reporting item, corresponding to a risk of bias so high that the findings should not be considered in the data synthesis. For these seven studies, we did not find it relevant to judge on the remaining items because of their already high risk of bias. None of the other studies were given a score of 5 on the incomplete data.

**Table 4.5:** Risk of Bias ‐ Distribution of the Studies Not Analysing STAR Data

**Risk of bias item**	**Judgement**								**Total number of studies**
	High	Low	Unclear	1	2	3	4	5	
**Sequence generation**	80	0	2	‐	‐	‐	‐	‐	82
**Allocation concealment**	80	0	2	‐	‐	‐	‐	‐	82
**Blinding** ^*^	‐	‐	0	0	0	0	82	0	82
**Incomplete data**^[Link]^, ^1:^	‐	‐	2	2	1	1	0	0	6
**Selective reporting**^[Link]^, ^2:^	‐	‐	0	5	0	1	0	3	9
**Other bias**^[Link]^, ^3:^	‐	‐	0	4	1	1	0	4	10
**Confounding**^[Link]^, ^4:^	‐	‐	0	2	0	1	2	51	56

Notes: *: The judgement is based on a 5‐point scale where 1 indicates low risk of bias and 5 indicates high risk of bias. Studies scoring 5 on any item of the risk of bias tool were not included in the data synthesis and therefore, it was not relevant to judge on the remaining items for these studies.

1: *Not judged for the eighteen studies that did not provide enough data to calculate an effect estimate, for the* 51 *studies scoring 5 on the confounding item, the four studies scoring 5 on the other bias item and the three studies scoring 5 on the selective reporting item.*

2: *Not judged for the eighteen studies that did not provide enough data to calculate an effect estimate, for the* 51 *studies scoring 5 on the confounding item and the four studies scoring 5 on the other bias item..*

3: *Not judged for the eighteen studies that did not provide enough data to calculate an effect estimate, for the* 51 *studies scoring 5 on the confounding item and the three studies scoring 5 on the selective reporting item.*

4: *Not judged for the eighteen studies that did not provide enough data to calculate an effect estimate, for the one study using a randomised design and neither for the seven studies that scored 5 on the selective reporting and other bias items.*

### 4.3 SYNTHESIS OF RESULTS

In order to carry out a meta‐analysis, every study must have a comparable type of effect size. All studies reported standardised mean differences (SMD) and variances or data that enabled calculation of standardised mean differences and variances. All studies, not analysing STAR data, reported outcomes by the end of the treatment (end of the school year) only. The STAR experiment was a four year longitudinal study with outcomes reported by the end of each school year.

All outcomes are scaled such that a positive effect size favours the students in small classes, i.e. when an effect size is *positive* a class size reduction improves the students' achievement.

#### 4.3.1 STAR studies

Four studies provided effect estimates that could be used in the data synthesis.

The four studies differed in terms of both the chosen comparison condition and decision rules in selecting a sample for analysis. Contrary to usual practice we report the results of all four studies and do not pool the results with the studies not analysing STAR data. We took into consideration the ICC in the results reported for the STAR experiment and corrected the effect sizes and standard errors using ρ = 0.22. Only the standard errors changed (increased) due to the correction implying wider confidence intervals than reported in the studies. The uncorrected results are shown in section 10.4.

All reported results indicated a positive effect favouring the treated; all of the study‐level effects were statistically significant. The study‐level effect sizes for reading varied between 0.17 and 0.34 and the study‐level effect sizes for mathematics varied between 0.15 and 0.33, see table 4.6. The effect sizes for reading reported in Hanushek (1999) were generally smaller than the other effect sizes for reading for each grade. Otherwise no clear patterns could be found.

**Table 4.6:** Effect Sizes from the STAR Experiment

	**Folger, 1989**	**Nye, 1992/994**	**Finn, 2001**	**Hanushek, 1999**
Read SMD [95% CI]				
Kindergarten	0.21 [0.07, 0.35]	0.18 [0.06, 0.30]	0.21 [0.07, 0.35]	0.17 [0.05, 0.29]
1. Grade	0.34 [0.20, 0.48]	0.24 [0.12, 0.36]	0.30 [0.16, 0.44]	0.23 [0.11, 0.35]
2. Grade	0.26 [0.12, 0.40]	0.23 [0.11, 0.35]	0.26 [0.12, 0.40]	0.20 [0.08, 0.32]
3. Grade	0.24 [0.10, 0.38]	0.26 [0.14, 0.38]	0.22 [0.10, 0.34]	0.22 [0.10, 0.34]
Mathematics SMD [95% CI]				
Kindergarten	0.17 [0.03, 0.31]	0.15 [0.03, 0.27]	0.19 [0.05, 0.33]	0.17 [0.03, 0.31]
1. Grade	0.33 [0.19, 0.47]	0.27 [0.15, 0.39]	0.31 [0.17, 0.45]	0.26 [0.14, 0.38]
2. Grade	0.23 [0.09, 0.37]	0.20 [0.08, 0.32]	0.25 [0.11, 0.39]	0.19 [0.07, 0.31]
3. Grade	0.21 [0.07, 0.35]	0.23 [0.11, 0.35]	0.15 [0.01, 0.29]	0.18 [0.06, 0.30]

#### 4.3.2 Non‐STAR studies

Six studies provided standardised mean differences and variances or data that enabled calculation of standardised mean differences and variances effect estimates that could be used in the data synthesis. No adjustment were necessary for clustering; as the studies either did not analyse whole classes (only one or a few students in a class), included class random effects or used a two level model (student and class).

Three studies compared the achievement of students in small classes to the achievement of students in larger classes (defined as reported in table 4.3). The class size reductions in these studies varied from a minimum of one student (the intended reduction was six students) in Munoz (2001), a minimum of seven students in Milesi (2006) to a minimum of 8 students in Ecalle (2006). Three studies (Bressoux, 2009; Gerritsen, 2017 and Hudson, 2011) included class size as a continuous variable in their models. Thus, the reported coefficients reflect the effect of a one student increase in class size on achievement. All three studies reported mean class size as well as the standard deviation of class size. We will use the effect of a standard deviation reduction in class size (as reported in the studies) in the data synthesis and investigate the robustness of results in the sensitivity analysis. Thus the results of the study by Bressoux (2009) will reflect a class size reduction of four students and the study by Gerritsen (2017) will reflect a class size reduction of five students. Concerning the study by Hudson (2011) it is, however, unclear what the correct sign of the effect is. The coefficient labels in the table of results (Table 3 page 17) are ‘Class size’ and the coefficient values reported are positive. Nevertheless, the interpretation in the text is that there is a positive effect of a class size reduction on achievement in reading as well as mathematics. Nowhere in the paper is it reported that the variable ‘class size’ is somehow rescaled to a variable reflecting decreasing class sizes. Thus, either the signs of the class size coefficients are incorrect or the interpretations in the text are incorrect. The results of this study will not be pooled with the other five studies but reported separately and included in the sensitivity analysis.

#### 4.3.3 Reading

Three of the reported results indicated a positive effect favouring the treated and two indicated a negative effect favouring the comparison; three of the study‐level effects were statistically non‐significant.

**Figure 4.1:**
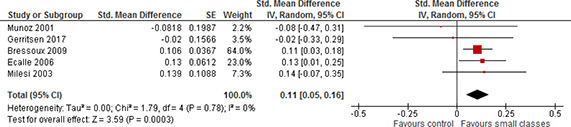
Reading

The weighted average was positive and statistically significant. The random effects weighted standardised mean difference was 0.11 (95% CI 0.05 to 0.16, p = 0.0003). Although the p‐value of the Q‐statistic is notoriously underpowered to detect heterogeneity in small meta‐analyses, the estimated τ^2^ is 0.00 and I^2^ is 0%, implying that heterogeneity among these five studies is not present. The forest plot is displayed in Figure 4.1.

The reported result in Hudson (2011) was a SMD of 0.03 [95% CI 0.01 to 0.04].

#### 4.3.4 Mathematics

**Figure 4.2:**
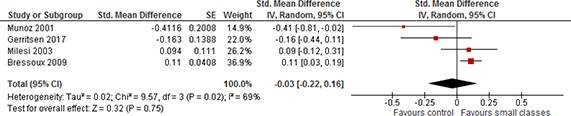
Mathematics

The study by Ecalle (2006) did not report results for mathematics. Two of the reported results indicated a positive effect favouring the treated and two indicated a negative effect favouring the comparison; two of the study‐level effects were statistically non‐significant. The weighted average was negative and statistically non‐significant. The random effects weighted standardised mean difference was ‐0.03 (95% CI ‐0.22 to 0.16, p = 0.75). The estimated τ^2^ is 0.02 and I^2^ is 69%, implying that there is some heterogeneity among these four studies. The forest plot is displayed in Figure 4.2.

The reported result in Hudson (2011) was a SMD of 0.02 [95% CI 0.01 to 0.04].

#### 4.3.5 Sensitivity analysis

Sensitivity analyses were planned to evaluate whether the pooled effect sizes were robust across study design and components of methodological quality. We found one randomised controlled trial, and evaluated the impact of study design. For methodological quality, we further carried out sensitivity analyses for the confounding risk of bias component of the risk of bias checklists. We examined the robustness of our conclusions when we excluded the study reporting results from a randomised controlled trial and when we excluded the study with risk of bias score of 4 on the confounding item. The analyses are performed separate by outcome, essentially replicating the meta‐analyses conducted in 4.3.3 and 4.3.4. We further examined the robustness of our conclusions when we did not multiply the reported effects with a standard deviation reduction in class size in the studies using class size as a continuous variable and when we included the reported result from the study with an unclear sign of the effect; including the effect both as a positive effect as well as negative effect. Last, we examined the robustness of our conclusions when including the extremes (smallest and largest) of the range of effect sizes from the STAR experiment.

The results of excluding the RCT study and the study with a score of 4 on the confounding risk of bias item are provided in table 4.7 and displayed in forest plots in section 12.

**Table 4.7:** Sensitivity Analysis. Exclusion of the RCT Study and the Study with a Score of 4 on the Confounding Risk of Bias Items. Separately by Outcome. Standardised Mean Difference (SMD) with 95% Confidence Interval (CI).

				**95% CI**	
**Outcome**	**Studies excluded**	**Number of studies k**	**Mean SMD**	**Lower**	**Upper**
		5	0.11	0.05	0.16
Reading	RCT	4	0.10	0.03	0.16
	Confounding score of 4	4	0.11	0.05	0.17
		4	‐0.03	‐0.22	0.16
Mathematics	Confounding score of 4	3	0.06	‐0.08	0.19

There were no appreciable changes in the results following removal of any of the studies.

In summary, the conclusions of the main syntheses do not change.

The results when not multiplying the reported effects with a standard deviation reduction in class size in the studies using class size as a continuous variable, of including the study with an unclear sign of the effect (Hudson, 2011) and include the extremes of the range of effect sizes from the STAR experiment are provided in table 4.8 and displayed in forest plots in section 12.

**Table 4.8:** Sensitivity Analysis. One Student Class Size Reduction, Inclusion of Standardised Mean Difference (SMD) with Unclear Sign and Extremes of the Range of STAR SMDs. Separately by Outcome. SMD with 95% Confidence Interval (CI).

				**95% CI**	
**Outcome**	**Change to analysis**	**Number of studies k**	**Mean SMD**	**Lower**	**Upper**
Reading		5	0.11	0.05	0.16
	One student reduction in class size in Bressoux, 2009 and Gerritsen, 2017	5	0.03	‐0.01	0.07
	Include Hudson (2011) with positive SMD	6	0.07	0.01	0.12
	Include Hudson (2011) with negative SMD	6	0.06	‐0.03	0.15
	Include Hanushek (1999) KG	6	0.12	0.07	0.17
	Include Folger (1989) 1G	6	0.14	0.05	0.24
Mathematics		4	‐0.03	‐0.22	0.16
	One student reduction in class size in Bressoux, 2009 and Gerritsen, 2017	4	‐0.00	‐0.07	0.07
	Include Hudson (2011) with positive SMD	5	0.02	‐0.07	0.11
	Include Hudson (2011) with negative SMD	5	‐0.00	‐0.11	0.1
	Include Finn (2001) 3G	5	0.03	‐0.10	0.17
	Include Folger (1989) 1G	5	0.05	‐0.13	0.23

The reading outcome lost statistically significance when Hudson (2011) was included with a negative SMD and when Bressoux, 2009 and Gerritsen, 2017 were included with a one student reduction in class size. Otherwise, there were no appreciable changes in the results.

In summary, the conclusion of the main synthesis concerning reading changes except when Hudson (2011) was included with a positive SMD and the conclusion concerning mathematics do not change.

## 5 Discussion

### 5.1 SUMMARY OF MAIN RESULTS

This review focused on the effect of reducing the class size on students' achievement. The available evidence does suggest that there is an effect on reading achievement, although the effect is small. We found a statistically significant positive effect of reducing the class size on reading. The effect on mathematics achievement was negative and not statistically significant. The effects were measured by standardised mean differences. The weighted average reading effect was 0.11 and the weighted average mathematics effect was ‐0.03. Measured as the probability‐of‐benefit (POB) statistic, defined as the probability that a randomly selected score from the treated population (small classes) would be greater than a randomly selected score from the comparison population, the reading POB was 0.531. A standardised mean difference of 0.11 in reading therefore corresponds to a 53 per cent chance that a randomly selected score of a student from the treated population of small classes is greater than the score of a randomly selected student from the comparison population. The lower and upper 95% confidence interval corresponds to 51 respectively 55 per cent chance of a randomly selected score of the treated being higher than a score from the comparison population.

A standardised mean difference of ‐0.03 in mathematics corresponds to a 49 per cent chance that a randomly selected score of a student from the treated population of small classes is greater than the score of a randomly selected student from the comparison population. The lower and upper 95% confidence interval corresponds to 44 respectively 55 per cent chance of a randomly selected score of the treated being higher than a score from the comparison population.

None of the studies that could be used in the meta‐analysis provided secondary outcomes.

### 5.2 OVERALL COMPLETENESS AND APPLICABILITY OF EVIDENCE

In this review we included in total ten studies in the data synthesis and of these only five studies were used in the meta‐analysis. This number is very low compared to the large number of studies (127) meeting the inclusion criteria. The reduction was caused by three different factors. A total of 45 studies analysed data from the STAR experiment. Only four of these studies, could be used in the data synthesis and none of them were included in the meta‐analysis as the decision rule as described in the protocol could not be used.

Of the remaining 82 studies not analysing STAR data, 18 studies did not report effect estimates or provide data that would allow the calculation of an effect size. Fifty eight studies were judged to have a very high risk of bias (5 on the scale) and, in accordance with the protocol, we excluded these from the data synthesis on the basis that they would be more likely to mislead than inform.

If all the 82 studies had provided an effect estimate with lower risk of bias, the final list of useable studies in the data synthesis would have been larger^7^ which again would have provided a more robust literature on which to base conclusions.

The five studies used in the meta‐analysis covered France, the Netherlands and USA, whereas 41 countries were represented by the 82 studies. The geographical coverage thus became narrower as studies from Australia, Belgium, Bolivia, Canada, Chinese Taipei, Columbia, Croatia, Cyprus, Czech Republic, Denmark, England, Germany, Greece, Hong Kong, Hungary, Iceland, Ireland, Israel, Italy, Japan, Korea, Lesotho, Lithuania, Malta, New Zealand, Norway, Poland, Portugal, Romania, Scotland, Singapore, Slovak Republic, Slovenia, Spain, Sri Lanka, Sweden, Switzerland and UK could not be used in the data synthesis. This is a clear limitation of the review.

All the studies used in the meta‐analysis were restricted to grade levels kindergarten to 3. Grade. This is also a clear limitation of the review.

It was not possible to examine the impact of the moderators.

None of the studies were eligible for analysis of any of the secondary outcomes.

### 5.3 QUALITY OF THE EVIDENCE

The majority of studies used non‐randomised designs. Overall the risk of bias in the included studies was high.

Among the 82 studies not analysing STAR data, fifty eight studies were judged to be at very high risk of bias. Among the 45 studies analysing STAR data, 14 studies were judged to be at very high risk of bias.

The risk of bias was examined using a tool for assessing risk of bias incorporating non‐randomised studies. We attempted to enhance the quality of the evidence in this review by excluding studies judged to be at very high risk of bias using this tool. We believe this process excluded those studies that are more likely to mislead than inform.

Furthermore, we performed a number of sensitivity analyses for each outcome to check whether the obtained results are robust across study design and methodological quality, to inclusion of a result with an unclear sign, inclusion of effect sizes from the STAR experiment and to multiplying the reported effect with a standard deviation reduction in class size in the studies using class size as a continuous variable.

To check the robustness across study design and methodological quality, we removed the study reporting results from a randomised controlled trial and we removed the study with risk of bias score of 4 on the confounding item. The overall conclusions did not change.

The reading outcome, however, lost statistically significance when the study with an unclear sign was included with a negative SMD and when the two studies using class size as a continuous variable were included with a one student reduction in class size instead of a class size standard deviation (as reported in the studies) reduction in class size. Otherwise the conclusions did not change.

There was overall inconsistency in the direction of effects on both the reading outcome and the mathematics outcome. Some effects favoured small classes and some effects favoured regular classes.

### 5.4 LIMITATIONS AND POTENTIAL BIASES IN THE REVIEW PROCESS

We believe that all the publicly available studies on the effect of a reduction in class size on student achievement up to the censor date were identified during the review process. However, eighteen references were not obtained in full text.

We believe that there are no other potential biases in the review process as two members of the review team^8^ independently coded the included studies. Any disagreements were resolved by discussion. Further, decisions about inclusion of studies and assessment of study quality were made by two review authors independently and minor disagreements resolved by discussion. Numeric data extraction was made by one review author and was checked by a second review author.

### 5.5 AGREEMENTS AND DISAGREEMENTS WITH OTHER STUDIES OR REVIEWS

To our knowledge this is the first systematic review of the literature on the effects on student achievement of reducing the class size, no directly comparable literature exists.

Early related contributions are the meta‐analysis by Glass and Smith (1979) and the updated literature reviews by Hanushek (Hanushek, 1989; 1999; 2003). Both samples of studies, however, included a number of studies analysing pupil‐teacher ratios and not the actual class size and both contributions included several estimates from the same datasets. Glass & Smith (1979) analysed 725 comparisons from their 77 included studies and based on a meta‐regression model, Glass and Smith (1979) conclude: ‘There is little doubt that, other things equal, more is learned in smaller classes' (p.15). The overall effect size (SMD) is 0.088 and they find no differential effects of subject taught.

Hanushek's quantitative summary of the literature is based on 277 estimates drawn from 59 studies. Based on a vote counting method, Hanushek concluded that “there is no strong or consistent relationship between school resources and student performance” (Hanushek, 1989, p. 47).

A more recent review is found in Shin & Chung (2009), which, however, also include several estimates from the same data set but only include studies analysing actual class size. Further, only studies conducted in the US and published in the period from 1989 to 2008 were included. Ultimately, 17 studies were included for analysis of which 8 are studies analysing STAR data. They computed a total of 120 effect sizes from the 17 studies. Based on a random effects model they find that combining all 120 effect estimates (of which 78 are from STAR) without considering dependence between them the pooled standardised mean difference (SMD) is 0.20. When dependence is taken into consideration, by using state as the unit of analysis (they use the average SMD per state implying the effect size used for Tennessee is a simple average of the 78 SMD based on STAR data), the pooled SMD decreases to 0.08.

Most recently, Chingos (2013) offers a review, though not a systematic review, and like the two earlier reviews also includes actual class size and pupil‐teacher ratio without any distinguishing between them. No data synthesis is performed, but a narrative synthesis is given (although effect sizes from each included study are shown where possible) and the overall conclusion is: ‘The evidence on the efficacy of class size is clearly mixed, with one high‐quality study finding quite large effects, another finding no effects, and a handful finding effects in between’ (p. 430).

The conclusions of these earlier reviews are, with the exception of Hanushek's reviews^9^, that the evidence is either mixed or favours small classes. However, none of the reviews properly take into consideration the dependence between effect estimates used in the analyses and with the exception of Shin & Chung (2009) they do not distinguish between actual class size and pupil/teacher ratio. Therefore the results are not directly comparable to the results of our review. The available evidence analysed in our systematic review does suggest that there is an effect of reducing class size on student achievement, although only in reading and the size of the effect is small. As such, the conclusions are not inconsistent, though, even if the reviews are based on different inclusion criteria concerning the intervention and substantially different approaches and statistical methods compared to ours.

## 6 Authors' conclusions

### 6.1 IMPLICATIONS FOR PRACTICE AND POLICY

The effectiveness of small class sizes for improving student achievement has been one of the most debated issues in educational research. One strand of class size research points to small and insignificant effects, another points to positive and significant effects. In this review, the intervention has been class size reduction. Studies only considering average class size measured as student‐teacher ratio at school level (or higher levels) were not included.

We have found evidence that there is an effect on reading achievement, although the effect is very small. We found a statistically significant positive effect of reducing the class size on reading. The effect on mathematics achievement was negative and not statistically significant.

Measured as the probability‐of‐benefit (POB) statistic, defined as the probability that a randomly selected score from the treated population (small classes) would be greater than a randomly selected score from the comparison population, the overall reading effect corresponds to a 53 per cent chance that a randomly selected score of a student from the treated population of small classes is greater than the score of a randomly selected student from the comparison population. The overall effect on mathematics achievement corresponds to a 49 per cent chance that a randomly selected score of a student from the treated population of small classes is greater than the score of a randomly selected student from the comparison population.

Class size reduction is costly and the available evidence points to no or only very small effect sizes of small classes in comparison to larger classes. Taking the individual variation in effects into consideration, we cannot rule out the possibility that small classes may be counterproductive for some students. It is therefore crucial to know more about the relationship between class size and achievement and how it influences what teachers and students do in the classroom in order to determine where money is best allocated.

### 6.2 IMPLICATIONS FOR RESEARCH

In this review we found evidence that reducing the class size results in an increased reading score, although the impact is very small. We found no evidence of an impact on the mathematics score.

By excluding from the data synthesis studies judged to be at very high risk of bias this review aimed at enhancing the quality of the evidence on the effects of reducing class size. We believe this process excluded those studies that are more likely to mislead than inform on the true effect sizes. Overall the risk of bias in the studies included in the review was high. Many of the available studies were judged to be at very high risk of bias. Fifty‐one of the studies not analysing STAR data were given a score of 5 on the confounding item, corresponding to a risk of bias so high that the findings should not be considered in the data synthesis. Of the remaining 13 studies, four were given a score of 5 on the Other risk of bias item and three were given a score of 5 on the Selective reporting item, corresponding to a risk of bias so high that the findings should not be considered in the data synthesis, leaving only six studies to be meta analysed.

Some of the studies judged to be at very high risk of bias, based the analysis on an instrument variable (IV) design relying on an average of class size (grade or regional) as instrument or a rule of maximum class size (and some studies in addition restricted the analysis to intervals around the discontinuities in class size induced by maximum class‐size rules). These studies, however, failed to deliver convincing arguments that the identification strategies were not subject to too high risk of selection. In general, the studies relying on an average class size as instrument did not explain or discuss the assumption that the instrument does not affect outcomes other than through their effect on class size and in some cases even the (first stage) effect on class size was very week. In general, there was a lack of country specific information given in the studies using rules of maximum class size (does the rule apply to all schools and to which extent is it binding). In addition, in some studies the IV class size was based on enrolment by the end of the school year and not the beginning which made it potentially endogenous.

A further concern is the practical use of effect sizes from studies using rules of maximum class size as instrument is that between the discontinuities triggered by the rules, predicted class size varies with actual enrolment, which is a function of the covariates. Therefore, predicted class size is not a valid instrument except when the rule triggers a change in the number of classes. Further, identification arises only when the rule binds, so if one uses a rule that binds only in some schools, one learns about the effects of class size only for those schools.

In general, studies using IV for causal inference only provides an estimate for a specific group namely, people whose behaviour change due to changes in the particular instrument used^10^. It is not informative about effects on never‐takers and always‐takers because the instrument does not affect their treatment status. The estimated effect is thus applicable only to the subpopulation whose treatment status is affected by the instrument. As a consequence, the effects differ for different IVs and care has to be taken as to whether they provide useful information. The effect is interesting when the instrument it is based on is interesting in the sense that it corresponds to a policy instrument of interest. Further, if those that are affected by the instrument are not affected in the same way the IV estimate is an average of the impacts of changing treatment status in both directions, and cannot be interpreted as a treatment effect. To turn the IV estimate into a local average treatment effect (LATE) requires a monotonicity assumption. The movements induced by the instrument go in one direction only, from no treatment to treatment. The IV estimate, interpreted as a LATE, is only applicable to the complier population, those that are affected by the instrument in the ‘right way’. It is not possible to characterise the complier population as an observation's subpopulation cannot be determined and defiers do not exist by assumption.

In the binary‐treatment– binary‐instrument context, the IV estimate can, given monotonicity, be interpreted as a LATE; i.e. the average treatment effect for the subpopulation of compliers. If treatment or instruments are not binary, interpretation becomes more complicated. In the binary‐treatment– multivalued‐instrument (ordered to take values from 0 to *J*) context, the IV estimate, given monotonicity, is a weighted average of pairwise LATE parameters (comparing subgroup *j* with subgroup *j*−1). The IV estimate can thus be interpreted as the weighted average of average treatment effects in each of the *J* subgroups of compliers. In the multivalued‐treatment (ordered to take values from 0 to *T*) – multivalued‐instrument (ordered to take values from 0 to *J*) context, the IV estimate for *each pair of instrument values*, given monotonicity, is a weighted average of the effects from going from *t*‐1 to *t* for persons induced by the change in the value of the instrument to move from any level below *t* to the level *t* or any level above. Persons can be counted multiple times in forming the weights.

As the effect of class size belongs to the multivalued‐treatment – multivalued‐instrument category, the results of the studies using IV for causal inference would have been very difficult, not to say impossible, to interpret and use for any practical purposes even if they had delivered convincing arguments that the instruments used were not subject to high risk of selection.

As studies from a variety of countries (38 countries) could not be used in the data synthesis the geographical coverage of the evidence of the effects of reducing the class size became rather narrow, covering only three countries, two European and the US.

The planned examination of potential moderators of the effect, such as gender, age, intensity and duration, was not possible due to low number of studies included in the data synthesis. If effect sizes from all the countries represented in the review had been useable in the data synthesis, additional valuable information about the heterogeneous effects of reducing the class size may have resulted.

These considerations point to the need for future studies that more thoroughly discuss the identifying assumptions and justify their choice of method by considering and reporting all relevant data and tests. Further, future studies should rely on identification strategies where the resulting effect sizes are manageable to interpret and use for practical and political purposes.

It would be natural to consider conducting a large randomised controlled trial (or a series of large RCTs) with specific allocation to small or standard size classes. Specific attention would also have to be paid to stringency in terms of conducting a well‐designed RCT with low risk of bias as well as ensuring that the sample sizes are large enough to enable sufficient power. The trial or trials should be designed, conducted and reported according to methodological criteria for rigour in respect of internal and external validity in order to achieve robust results regarding both the short‐term and the longer‐term effects.

## 7 Methods Not Implemented

### 7.1.1 Assessment of reporting bias

We were unable to comment on the possibility of publication bias because there were insufficient studies for the construction of funnel plots.

### 7.1.2 Moderator analysis and investigation of heterogeneity

We planned to investigate the following factors with the aim of explaining observed heterogeneity: Study‐level summaries of participant characteristics (studies considering a specific age (or grade level) group or socioeconomic status group, or studies where separate effects for high/low socioeconomic status or age (grade level) divided are available), intensity (size of reduction and initial class size) and duration (number of years in a small class).

There were, however, insufficient studies for moderator analysis to be performed.

## 8 References

### 8.1 REFERENCES TO INCLUDED STUDIES

References denoted with ‐ is a working paper attached to the primary reference listed just above.

#### 8.1.1 STAR studies

Achilles, C. M. (1993). The Lasting Benefits Study (LBS) in Grades 4 and 5 (1990‐1991): A Legacy from Tennessee's Four‐Year (K‐3) Class‐Size Study (1985‐1989), Project STAR. Paper# 7. Working Paper, Tennessee State Univ., Nashville. Center of Excellence: Basic Skills.Achilles, C. M. (1993). The Teacher Aide Puzzle: Student Achievement Issues. An Exploratory Study. Paper presented at the Annual Meeting of the Mid‐South Educational Research Association (New Orleans, LA, November 1993)Balestra, S., & Backes‐Gellner, U. (2014). Revisiting Class‐Size Effects: Where They Come From and How Long They Last (No. 0102). University of Zurich, Institute for Strategy and Business Economics (ISU).Bingham, C. S. (1994). Class Size as an Early Intervention Strategy in White‐Minority Achievement Gap Reduction. Paper presented at the Annual Meeting of the American Association of School Administrators (San Francisco, CA, February 1994).Chetty, R., Friedman, J. N., Hilger, N., Saez, E., Schanzenbach, D. W., & Yagan, D. (2011). How does your kindergarten classroom affect your earnings? Evidence from Project STAR. The Quarterly Journal of Economics, 126(4), 1593–1660.2225634210.1093/qje/qjr041Ding, W., & Lehrer, S. (2005). Class size and student achievement: Experimental estimates of who benefits and who loses from reductions. Queen's University Department of Economics Working Papers, (1046).Ding, W., & Lehrer, S. F. (2010). Estimating treatment effects from contaminated multiperiod education experiments: the dynamic impacts of class size reductions. The Review of Economics and Statistics, 92(1), 31–42.Ding, W., & Lehrer, S. F. (2009). Estimating Treatment Effects from Contaminated Multi‐Period Education Experiments: The Dynamic Impacts of Class Size Reductions. NBER Working Paper (No. w15200).Ding, W., & Lehrer, S. F. (2007). Estimating Treatment Effects from Contaminated Multi‐Period Education Experiments: The Dynamic Impacts of Class Size Reductions. CLSRN Working Paper, No. 35
Ding, W., & Lehrer, S. F. (2011). Experimental estimates of the impacts of class size on test scores: robustness and heterogeneity. Education Economics, 19(3), 229–252.Finn, J. D. (1998). Class Size and Students at Risk. What Is Known? What Is Next? A Commissioned Paper. Technical Report, National Inst. on the Education of At‐Risk Students.Finn, J. D., & Achilles, C. M. (1990). Answers and questions about class size: A statewide experiment. American Educational Research Journal, 27(3), 557–577.Finn, J. D., & Achilles, C. M. (1999). Tennessee's class size study: Findings, implications, misconceptions. Educational evaluation and policy analysis, 21(2), 97–109.Finn, J. D., Achilles, C. M., Bain, H. P., Folger, J., Johnston, J. M., Lintz, M. N., & Word, E. R. (1990). Three years in a small class. Teaching and Teacher Education, 6(2), 127–136.Finn, J. D., Fulton, D., Zaharias, J., & Nye, B. A. (1989). Carry‐over effects of small classes. Peabody Journal of Education, 67(1), 75–84.Finn, J. D., Gerber, S. B., & Boyd‐Zaharias, J. (2005). Small classes in the early grades, academic achievement, and graduating from high school. Journal of Educational Psychology, 97(2), 214.Finn, J. D., Gerber, S. B., Achilles, C. M., & Boyd‐Zaharias, J. (2001). The enduring effects of small classes. Teachers College Record, 103(2), 145–183.Folger, J., & Breda, C. (1989). Evidence from Project STAR about class size and student achievement. Peabody Journal of Education, 67(1), 17–33.Hanushek, E. A. (1999). Some findings from an independent investigation of the Tennessee STAR experiment and from other investigations of class size effects. Educational Evaluation and Policy Analysis, 21(2), 143–163.Harvey, B. H. (1994). The Effect of Class Size on Achievement and Retention in the Primary Grades: Implications for Policy Makers. Unpublished Report.Jackson, E., & Page, M. E. (2013). Estimating the distributional effects of education reforms: A look at Project STAR. Economics of Education Review, 32, 92–103.Jacobs, R. (1987). The Effect of Class Sizes of 1: 15, 1: 25, and 1: 25 Plus Full‐Time Aide on Kindergarten Reading Readiness Achievement. (Doctoral Dissertation, Tennessee State University)Konstantopoulos, S. (2008). Do small classes reduce the achievement gap between low and high achievers? Evidence from Project STAR. The Elementary School Journal, 108(4), 275–291.Konstantopoulos, S. (2011). How consistent are class size effects?
Evaluation Review, 35(1), 71–92.2136264110.1177/0193841X11399847Konstantopoulos, S. (2009). How Consistent Are Class Size Effects?
IZA Discussion Paper 4566.10.1177/0193841X1139984721362641Konstantopoulos, S., & Chung, V. (2009). What are the long‐term effects of small classes on the achievement gap? Evidence from the lasting benefits study. American Journal of Education, 116(1), 125–154.Krueger, A. B. (1999). Experimental estimates of education production functions. The Quarterly Journal of Economics, 114(2), 497–532.Krueger, A. B. (1997). Experimental Estimates of Education Production Functions. NBER Working Paper No. 6051.Krueger, A. B., & Whitmore, D. M. (2001). The effect of attending a small class in the early grades on college‐test taking and middle school test results: Evidence from Project STAR. The Economic Journal, 111(468), 1–28.Krueger, A. B., & Whitmore, D. M. (2001). The effect of attending a small class in the early grades on college‐test taking and middle school test results: Evidence from Project STAR. NBER Working Paper No. 7656.Krueger, A. B., & Whitmore, D. M. (2001). Would smaller classes help close the black‐white achievement gap?
Working Paper (Vol. 451). Industrial Relations Section, Princeton University.Lehrer, S., & Ding, W. (2004, August). Estimating Dynamic Treatment Effects from Project STAR. In Econometric Society 2004 North American Summer Meetings. Working Paper (No. 252) Econometric Society.McKee, G., Sims, K. R., & Rivkin, S. G. (2015). Disruption, learning, and the heterogeneous benefits of smaller classes. Empirical Economics, 48(3), 1267–1286.McKee, G. J., Rivkin, S. G., & Sims, K. R. (2010). Disruption, achievement and the heterogeneous benefits of smaller classes. NBER Working Paper No. 15812
Mitchell, D. E., Beach, S. A., & Badarak, G. (1989). Modeling the relationship between achievement and class size: A re‐analysis of the Tennessee project star data. Peabody Journal of Education, 67(1), 34–74.Mosteller, F. (1995). The Tennessee study of class size in the early school grades. The Future of Children, 5(2), 113–127
8528684Nye, B. A. (1992). The Lasting Benefits Study: A Continuing Analysis of the Effect of Small Class Size in Kindergarten through Third Grade on Student Achievement Test Scores in Subsequent Grade Levels: Fifth Grade. Technical Report, Tennessee State Univ., Nashville. Center of Excellence: Basic Skills.Nye, B. A. (1993). Class‐Size Research from Experiment to Field Study to Policy Application. Paper presented at the Annual Meeting of the American Educational Research Association (74th; Atlanta, GA, April 12‐16, 1993).Nye, B. A., Achilles, C. M., Boyd‐Zaharias, J., Fulton, B. D., & Wallenhorst, M. P. (1994). Small Is Far Better. Research in the Schools, 1(1), 9–20.Nye, B. A., Achilles, C. M., Zaharias, J. B., Fulton, B. D., & Wallenhorst, M. P. (1992). Small is far better: A report on three class‐size initiatives. Report, Mid‐South Educational Research Association, Knoxville, Tennessee.Nye, B. A., Hedges, L. V., & Konstantopoulos, S. (2000). Do the disadvantaged benefit more from small classes? Evidence from the Tennessee class size experiment. American journal of education, 109(1), 1–26.Nye, B., Hedges, L. V., & Konstantopoulos, S. (2000). The effects of small classes on academic achievement: The results of the Tennessee class size experiment. American Educational Research Journal, 37(1), 123–151.Nye, B., Hedges, L. V., & Konstantopoulos, S. (2001). Are effects of small classes cumulative? Evidence from a Tennessee experiment. The Journal of Educational Research, 94(6), 336–345.Nye, B., Hedges, L. V., & Konstantopoulos, S. (2001). The long‐term effects of small classes in early grades: Lasting benefits in mathematics achievement at grade 9. The Journal of Experimental Education, 69(3), 245–257.Nye, B., Hedges, L. V., & Konstantopoulos, S. (2002). Do low‐achieving students benefit more from small classes? Evidence from the Tennessee class size experiment. Educational Evaluation and Policy Analysis, 24(3), 201–217.Prais, S. J. (1996). Class‐size and Learning: the Tennessee experiment—what follows?
Oxford Review of Education, 22(4), 399–414.Schanzenbach, D. W. (2007). What Have Researchers Learned from Project STAR?
Brookings Papers on Education Policy, 205(228), 2006–2007.Shin, Y. (2012). Do black children benefit more from small classes? Multivariate instrumental variable estimators with ignorable missing data. Journal of Educational and Behavioral Statistics, 37(4), 543–574.Shin, Y., & Raudenbush, S. W. (2011). The causal effect of class size on academic achievement: Multivariate instrumental variable estimators with data missing at random. Journal of Educational and Behavioral Statistics, 36(2), 154–185.Sohn, K. (2015). Nonrobustness of the Carryover Effects of Small Classes in Project STAR. Teachers College Record, 117(3).Word, E. (1990). Student/Teacher Achievement Ratio (STAR) Tennessee's K‐3 Class Size Study. Final Summary Report 1985‐1990.

#### 8.1.2 Non‐STAR studies

Word, E. (1990). Student/Teacher Achievement Ratio (STAR) Tennessee's K‐3 Class Size Study. Final Summary Report 1985‐1990. Technical Report.Achilles, C. M., Harman, P., & Egelson, P. (1995). Using Research Results on Class Size To Improve Pupil Achievement Outcomes. Research in the Schools, 2(2), 23–30.Akerhielm, K. (1995). Does class size matter?
Economics of education Review, 14(3), 229–241.Allhusen, V., Belsky, J., Booth‐LaForce, C. L., Bradley, R., Brownwell, C. A., Burchinal, M., & Hirsh‐Pasek, K. (2004). Does class size in first grade relate to children's academic and social performance or observed classroom processes?
Developmental psychology, 40(5), 651.1535515610.1037/0012-1649.40.5.651Angrist, J. D., & Lavy, V. (1999). Using Maimonides' rule to estimate the effect of class size on scholastic achievement. The Quarterly Journal of Economics, 114(2), 533–575.Angrist, J. D., & Lavy, V. (1997). Using Maimonides' Rule to Estimate the Effect of Class Size on Student Achievement. NBER Working Paper No. 5888
Angrist, J. D., Battistin, E., & Vuri, D. (2014). In a small moment: class size and moral hazard in the Mezzogiorno. NBER Working Paper No. 20173.Annevelink, E., Bosker, R., & Doolaard, S. (2004). Additional staffing, classroom processes, and achievement. Paper Onderwijs Research Dagen (9‐11 juni 2004). Utrecht.Blatchford, P. (2003). The class size debate: Is small better?. McGraw‐Hill Education (UK).Blatchford, P., Bassett, P., Goldstein, H., & Martin, C. (2003). Are class size differences related to pupils' educational progress and classroom processes? findings from the institute of education class size study of children aged 5‐7 years. British Educational Research Journal, 29(5), 709–730.Blatchford, P., Goldstein, H., Martin, C., & Browne, W. (2002). A study of class size effects in English school reception year classes. British Educational Research Journal, 28(2), 169–185.Bonesrønning, H. (2003). Class size effects on student achievement in Norway: Patterns and explanations. Southern Economic Journal, 69(4), 952–965.Boozer, M. A., & Maloney, T. (2001). The Effects of Class Size on the Long‐Run Growth in Reading Abilities and Early Adult Outcomes in the Christchurch Health and Development Study. Working Paper (No. 01/14) New Zealand Treasury.Boozer, M., & Rouse, C. (2001). Intraschool variation in class size: Patterns and implications. Journal of Urban Economics, 50(1), 163–189.Boozer, M., & Rouse, C. (1995). Intraschool Variation in Class Size: Patterns and Implications. NBER Working Paper No. 5144
Borland, M. V., Howsen, R. M., & Trawick, M. W. (2005). An investigation of the effect of class size on student academic achievement. Education Economics, 13(1), 73–83.Bosworth, R. (2014). Class size, class composition, and the distribution of student achievement. Education Economics, 22(2), 141–165.Bressoux, P., Kramarz, F., & Prost, C. (2009). Teachers' training, class size and students' outcomes: Learning from administrative forecasting mistakes. The Economic Journal, 119(536), 540–561.Breton, T. R. (2014). Evidence that class size matters in 4th grade mathematics: An analysis of TIMSS 2007 data for Colombia. International Journal of Educational Development, 34, 51–57
Breton, T. R. (2012). Evidence that Class Size Matters in 4th Grade Mathematics: An Analysis of TIMSS 2007 Data for Colombia. Working Paper, SSRN: http://ssrn.com/abstract=2049154
Burde, R. H. (1990). A study of the relationship of class size and student achievement on the Michigan Educational Assessment Program fourth‐grade test. (Dissertation, Western Michigan University).Carpenter, J. R., Goldstein, H., & Rasbash, J. (2003). A novel bootstrap procedure for assessing the relationship between class size and achievement. Journal of the Royal Statistical Society: Series C (Applied Statistics), 52(4), 431–443.Chargois, T. (2008). Student achievement: Identification of impact variables. (Dissertation, Lamar University‐Beaumont).Clanet, J. (2010). The relationship between teaching practices and student achievement in first year classes. European Journal of Psychology of Education, 25(2), 192–206.Costello, P. A. (1992). The Effectiveness of Class Size on Reading Achievement. Unpublished report.Dee, T. S., & West, M. R. (2011). The non‐cognitive returns to class size. Educational Evaluation and Policy Analysis, 33(1), 23–46.Dee, T., Finn, W. T. J., Hollister, R., Tyler, J., & Rubinstein, Y. (2008). The Non‐Cognitive Returns to Class Size. NBER Working Papers (NO. 13994).Dennis, B. D. (1986). Effects of small class size (1: 15) on the teaching/learning process in grade two. (Dissertation, Tennessee State University).Department of Education. Planning, Research, and Evaluation Branch
(1995). The Nevada class size reduction evaluation study 1995. Report, Nevada Dept. of Education.Dharmadasa, I. (1995). Class Size and Student Achievement in Sri Lanka. Paper presented at the Annual Conference of the Mid‐South Educational Research Association (Biloxi, MS, November 8‐10, 1995).Dieterle, S. (2013). Development Class‐size Reduction Policies and the Quality of Entering Teachers. Edinburgh School of Economics, ESE Discussion Papers NO. 224.Dobbelsteen, S., Levin, J., & Oosterbeek, H. (2002). The causal effect of class size on scholastic achievement: distinguishing the pure class size effect from the effect of changes in class composition. Oxford Bulletin of Economics and statistics, 64(1), 17–38.Ecalle, J., Magnan, A., & Gibert, F. (2006). Class size effects on literacy skills and literacy interest in first grade: A large‐scale investigation. Journal of school psychology, 44(3), 191–209.Galton, M., & Pell, T. (2012). Longitudinal effects of class size reductions on attainment: Results from Hong Kong primary classrooms. International Journal of Educational Research, 53, 360–369.Gerritsen, S., Plug, E., & Webbink, D. (2017). Teacher quality and student achievement: Evidence from a sample of Dutch twins. Journal of Applied Econometrics, 32(3), 643–660.Gilman, D. A. (1988). Prime Time in the First Grade at the North Gibson School Corporation: The First Four Years. A Longitudinal Evaluation of Indiana's State‐Supported Reduced Class Size Program. Technical Report.Gilman, D. A., Swan, E., & Stone, W. (1988). The educational effects of a state supported reduced class size program: A Comprehensive Evaluation of Indiana's Project PRIME TIME at the North Gibson School Corporation. Contemporary Education, 59(2), 112.Haenn, J. F. (2002). Class Size and Student Success: Comparing the Results of Five Elementary Schools Using Small Class Sizes. Working paper presented at the Annual Meetings Of the American Educational Research's Association.Hallinan, M. T., & Sørensen, A. B. (1985). Class size, ability group size, and student achievement. American Journal of Education, 94(1), 71–89.Hirschfeld, H. (2016). Class Size in Relation to Student Achievement and Behavioral Issues. (Master Thesis, Goucher College, Baltimore).Hojo, M. (2011). Education Production Function and Class‐Size Effects in Japanese Public Schools. Technical Report, Institute of Economic Research, Hitotsubashi University.Hojo, M. (2013). Class‐size effects in Japanese schools: A spline regression approach. Economics Letters, 120(3), 583–587.Hudson, Z. (2011). An Argument for a Nationalized Education Effort: Confronting the Complications of the Tradeoffs between Teacher Quality and Class Size. Working Paper, SSRN: https://papers.ssrn.com/sol3/papers.cfm?abstract_id=1747297
Iacovou, M. (2002). Class size in the early years: Is smaller really better?
Education Economics, 10(3), 261–290.Iacovou, M. (2001). Class size in the early years: is smaller really better? ISER Working Paper Series (No. 2001‐10).Jakubowski, M., & Sakowski, P. (2006). Quasi‐experimental estimates of class size effect in primary schools in Poland. International Journal of Educational Research, 45(3), 202–215.Jakubowski, M., & Sakowski, P. (2006). Quasi‐Experimental Estimates of Class Size Effect in Primary Schools in Poland. MPRA Working Paper NO. 4958.Konstantopoulos, S., & Shen, T. (2016). Class size effects on mathematics achievement in Cyprus: evidence from TIMSS. Educational Research and Evaluation, 22(1‐2), 86–109.Konstantopoulos, S., & Traynor, A. (2014). Class Size Effects on Reading Achievement Using PIRLS Data: Evidence from Greece. Teachers College Record, 116(2), n2.Krueger, A., & Lindahl, M. (2002). The School's Need for Resources‐A Report on the Importance of Small Classes. Report, The Expert Group on Public Finance (ESO), Stockholm.Lavy, V. (2001). Estimating the Effect of School Time of Instruction on Student Achievements. Hebrew University Economics Working Paper No. 01‐4
Levin, J. (2001). For whom the reductions count: A quantile regression analysis of class size and peer effects on scholastic achievement. Empirical Economics, 26(1), 221–246.Li, W. (2015). Two essays on educational research:(1) Using maximum class size rules to evaluate the causal effects of class size on mathematics achievement: Evidence from TIMSS 2011;(2) Power considerations for models of change. (Dissertation. Michigan State University).Li, W., & Konstantopoulos, S. (2016). Class Size Effects on Fourth‐Grade Mathematics Achievement: Evidence From TIMSS 2011. Journal of Research on Educational Effectiveness, 9(4), 503–530.Li, W., & Konstantopoulos, S. (2017). Does class‐size reduction close the achievement gap? Evidence from TIMSS 2011. School Effectiveness and School Improvement, 28(2), 292–313.Lindahl, M. (2005). Home versus school learning: A new approach to estimating the effect of class size on achievement. The Scandinavian Journal of Economics, 107(2), 375–394.Lindahl, M. (2001). Home versus School Learning: A New Approach to Estimating the Effect of Class Size on Achievement. IZA Discussion Papers 261.Ma, L., & Koenker, R. (2006). Quantile regression methods for recursive structural equation models. Journal of Econometrics, 134(2), 471–506.Maier, P., Molnar, A., Percy, S., Smith, P., & Zahorik, J. (1997). First Year Results of the Student Achievement Guarantee in Education Program. Report, Wisconsin Univ., Madison. Center for Urban Iniatives and Research.Maples, J. B. (2009). An analysis of the effects of class size on student achievement in selected middle schools in the Sandhills Region of North Carolina. (Dissertation. Fayetteville State University).McGiverin, J., Gilman, D., & Tillitski, C. (1989). A meta‐analysis of the relation between class size and achievement. The Elementary School Journal, 90(1), 47–56.Merritt, E. G., Rimm‐Kaufman, S. E., Berry, R. Q., Walkowiak, T. A., & Larsen, R. A. (2011). The Contribution of Mathematics Instructional Quality and Class Size to Student Achievement for Third Grade Students from Low Income Families. SREE Fall 2011 Conference Abstract.Milesi, C., & Gamoran, A. (2006). Effects of class size and instruction on kindergarten achievement. Educational Evaluation and Policy Analysis, 28(4), 287–313.Milesi, C., & Gamoran, A. (2003). Effects of Class Size and Instruction on Kindergarten Achievement. Conference Papers ‐ American Sociological Association.Molnar, A., Smith, P., & Zahorik, J. (1998). Evaluation Results of the Student Achievement Guarantee in Education (SAGE) Program, 1997‐98. Report, Wisconsin Univ., Madison. School of Education.Molnar, A., Smith, P., & Zahorik, J. (1999). Evaluation Results of the Student Achievement Guarantee in Education (SAGE) Program, 1998‐99. Report, Wisconsin Univ., Madison. School of Education.Molnar, A., Smith, P., Zahorik, J., Halbach, A., Ehrle, K., & Hoffman, L. M. (2001). 2000‐2001 evaluation results of the Student Achievement Guarantee in Education (SAGE) Program. Report, Center for Education, Research, Analysis and Innovation
Molnar, A., Smith, P., Zahorik, J., Palmer, A., Halbach, A., & Ehrle, K. (1999). Evaluating the SAGE program: A pilot program in targeted pupil‐teacher reduction in Wisconsin. Educational Evaluation and Policy Analysis, 21(2), 165–177.Moshoeshoe, R. (2015). Average and Heterogeneous Effects of Class Size on Educational Achievement in Lesotho. Working Paper (No. 496)
Economic Research Southern Africa.Munoz, M. A. (2001). Class Size Reduction in a Large Urban School District: A Mixed Methodology Evaluation Research Study. Unpublished Report, Jefferson County Public Schools, Louisville, KY.Murdoch, J. (1986). The effects of age, gender, class size, and school on academic achievement and social adjustment: in grades one through five (Doctoral dissertation, Brigham Young University. Department of Curriculum and Instructional Science).Maasoumi, E., Millimet, D. L., & Rangaprasad, V. (2005). Class size and educational policy: who benefits from smaller classes?
Econometric Reviews, 24(4), 333–368.Nandrup, A. B. (2016). Do class size effects differ across grades?
Education Economics, 24(1), 83–95.Nandrup, A. B. (2015). Do class size effects differ across grades?. Economic Working Papers 2015‐07
Aarhus University.Otsu, T., Xu, K. L., & Matsushita, Y. (2015). Empirical likelihood for regression discontinuity design. Journal of Econometrics, 186(1), 94–112.Pong, S. L., & Pallas, A. (2001). Class size and eighth‐grade math achievement in the United States and abroad. Educational evaluation and policy analysis, 23(3), 251–273.Sanogo, Y., & Gilman, D. (1994). Class Size and Student Achievement: Tennessee's Star and Indiana's Prime Time Projects. Unpublished Report.Shapson, S. M., Wright, E. N., Eason, G., & Fitzgerald, J. (1980). An experimental study of the effects of class size. American Educational Research Journal, 17(2), 141–152.Tienken, C. H., & Achilles, C. M. (2009). Relationship Between Class Size and Students' Opportunity to Learn Writing in Middle School. Research in the Schools, 16(1).Tillitski, C., Gilman, D., Mohr, A., & Stone, W. (1998). Class size reduction in North Givson School corporation: A three‐year cohort study. ERS Spectrum, 6(4), 37–40.Uhrain, C. (2016). Effect of Class Size on Student Achievement in Secondary School (Doctoral dissertation, Walden University).Urquiola, M. (2006). Identifying class size effects in developing countries: Evidence from rural Bolivia. The Review of Economics and Statistics, 88(1), 171–177.Urquiola, M. (2000). Identifying class size effects in developing countries: Evidence from rural schools in Bolivia Working Paper, (Vol. 2711) World Bank, Development Research Group, Public Services for Human Development Team.Urquiola, M. (2001). Identifying class size effects in developing countries: Evidence from rural schools in Bolivia. Policy Research Working Paper Series.Vaag Iversen, J. M., & Bonesrønning, H. (2013). Disadvantaged students in the early grades: will smaller classes help them?
Education Economics, 21(4), 305–324.Watson, K., Watson, K., Handal, B., Handal, B., Maher, M., & Maher, M. (2016). The influence of class size upon numeracy and literacy performance. Quality Assurance in Education, 24(4), 507–527.West, M. R., & Woessmann, L. (2003). Which School Systems Sort Weaker Students into Smaller Classes? International Evidence. IZA Discussion Paper No. 744
West, M. R., & Wöβmann, L. (2006). Which school systems sort weaker students into smaller classes? International evidence. European Journal of Political Economy, 22(4), 944–968.West, M. R., & Wöβmann, L. (2002). Class‐Size Effects in School Systems Around the World: Evidence from Between‐Grade Variation in TIMSS. IZA Discussion Paper No. 485.West, M. R., & Wöβmann, L. (2002). Class‐Size Effects in School Systems Around the World: Evidence from Between‐Grade Variation in TIMSS. Working Paper (No.1099)
Kiel Institute for the World Economy (IfW).Wiermann, C. (2005). Class Size, Instruction Time and Central Exit Examinations: disentangling the Relative Contributions to Scholastic Achievement. Discussion Paper (No. 05‐04)
Research Group Heterogeneous Labor, University of Konstanz/ZEW Mannheim.Wöβmann, L. (2005). Educational production in East Asia: The impact of family background and schooling policies on student performance. German Economic Review, 6(3), 331–353.Wöβmann, L. (2003). Educational Production in East Asia: The Impact of Family Background and Schooling Policies on Student Performance. IZA Discussion Paper No. 745.Wöβmann, L. (2005). Educational production in Europe. Economic policy, 20(43), 446–504.Wöβmann, L. (2003). European education production functions: what makes a difference for student achievement in Europe?
Working Paper (No. 190). Directorate General Economic and Financial Affairs (DG ECFIN), European Commission.Woessmann, L., & West, M. (2006). Class‐size effects in school systems around the world: Evidence from between‐grade variation in TIMSS. European Economic Review, 50(3), 695–736.Wossmann, L. (2003). Educational production in East Asia: the impact of family background and schooling policies on student performance. Working Paper, Kiel Institute for the World Economy (IfW).Yan, W., & Lin, Q. (2005). Effects of class size and length of day on kindergartners' academic achievement: Findings from early childhood longitudinal study. Early Education and Development, 16(1), 49–68.

### 8.2 REFERENCES TO EXCLUDED STUDIES

Akabayashi, H., & Nakamura, R. (2014). Can small class policy close the gap? An empirical analysis of class size effects in Japan. The Japanese Economic Review, 65(3), 253–281.Altinok, N., & Kingdon, G. (2012). New evidence on class size effects: A pupil fixed effects approach. Oxford Bulletin of Economics and Statistics, 74(2), 203–234.Altinok, N., & Kingdon, G. (2009). New Evidence on Class Size Effects: A Pupil Fixed Effects Approach. Working Paper No 2009‐16, CSAE Working Paper Series from Centre for the Study of African Economies, University of Oxford
Babcock, P., & Betts, J. R. (2009). Reduced‐Class Distinctions: Effort, Ability, and the Education Production Function. NBER Working Paper No. 14777.Baroody, A. E. (2017). Exploring the contribution of classroom formats on teaching effectiveness and achievement in upper elementary classrooms. School Effectiveness and School Improvement, 28(2), 314–335.Bernal, P., Mittag, N., & Qureshi, J. A. (2016). Estimating effects of school quality using multiple proxies. Labour Economics, 39, 1–10.Browning, M., & Heinesen, E. (2007). Class size, teacher hours and educational attainment. The Scandinavian Journal of Economics, 109(2), 415–438.Cho, H., Glewwe, P., & Whitler, M. (2012). Do reductions in class size raise students' test scores? Evidence from population variation in Minnesota's elementary schools. Economics of Education Review, 31(3), 77–95.Choi, E. J., Moon, H. R., & Ridder, G. (2017). Within‐District School Lotteries, District Selection, and The Average Partial Effects of School Inputs. USC Dornsife Institute for New Economic Thinking Working Paper No. 17 ‐ 11
Chowa, G. A., Masa, R. D., Ramos, Y., & Ansong, D. (2015). How do student and school characteristics influence youth academic achievement in Ghana? A hierarchical linear modeling of Ghana YouthSave baseline data. International Journal of Educational Development, 45, 129–140.Corak, M., & Lauzon, D. (2009). Differences in the distribution of high school achievement: The role of class‐size and time‐in‐term. Economics of Education Review, 28(2), 189–198.Corak, M., & Lauzon, D. (2010). Differences in the Distribution of High School Achievement: The Role of Class Size and Time‐in‐Term. Analytical Studies Branch Research Paper Series, 270.Corak, M., & Lauzon, D. (2010). Differences in the Distribution of High School Achievement: The Role of Class Size and Time‐in‐Term. IZA Discussion Paper No. 4824
Cordero Ferrera, J. O. S. É., Crespo Cebada, E., PedrajaChaparro, F., & Santín González, D. (2011). Exploring educational efficiency divergences across Spanish regions in PISA 2006. Revista de economíaaplicada, 19(57).Coupé, T., Olefir, A., & Alonso, J. D. (2016). Class size, school size and the size of the school network. Education Economics, 24(3), 329–351.Coupé, T., Olefir, A., & Alonso, J. D. (2011). Is optimization an opportunity? an assessment of the impact of class size and school size on the performance of Ukrainian secondary schools. Policy Research Working Papers November 2011.Denny, K., & Oppedisano, V. (2013). The surprising effect of larger class sizes: Evidence using two identification strategies. Labour Economics, 23, 57–65.Denny, K., & Oppedisano, V. (2010). Class size effects: evidence using a new estimation technique. Working Paper (No. 10/39), UCD Centre for Economic Research
Dieterle, S. G. (2015). Class‐size reduction policies and the quality of entering teachers. Labour Economics, 36, 35–47.Fabunmi, M., Brai‐Abu, P., & Adeniji, I. A. (2007). Class factors as determinants of secondary school student's academic performance in Oyo State, Nigeria. Journal of Social Science, 14(3), 243–247.Fredriksson, P., Öckert, B., & Oosterbeek, H. (2013). Long‐term effects of class size. The Quarterly Journal of Economics, 128(1), 249–285.Fredriksson, P., Öckert, B., & Oosterbeek, H. (2011). Long‐term effects of class size. IZA Discussion Papers No. 5879.Fredriksson, P., Öckert, B., & Oosterbeek, H. (2012). Långsiktiga effekter av mindre klasser. IFAU Rapport 2012:5.Fredriksson, P., Öckert, B., & Oosterbeek, H. (2014). Inside the Black Box of Class Size: Mechanisms, Behavioral Responses, and Social Background. IZA Discussion Papers No. 8019.Funkhouser, E. (2009). The effect of kindergarten classroom size reduction on second grade student achievement: Evidence from California. Economics of Education Review, 28(3), 403–414.Gary‐Bobo, R. J., & Mahjoub, M. B. (2013). Estimation of Class‐Size Effects, Using” Maimonides' Rule” and Other Instruments: the Case of French Junior High Schools. Annals of Economics and Statistics, 193–225.Gary‐Bobo, R. J., & Mahjoub, M. B. (2013). Estimation of Class‐Size Effects, Using” Maimonides' Rule” and Other Instruments: the Case of French Junior High Schools. CEPR Discussion Papers No 5754.Grantham, M. K. (2000). Impact of Small Class Size on Achievement. Unpublished Report.Heinesen, E. (2010). Estimating Class‐size Effects using Within‐school Variation in Subject‐specific Classes. The Economic Journal, 120(545), 737–760.Heinesen, E. (2007). Estimating class‐size effects using variation in subject‐specific classes. AKF (Amternes og Kommunernes Forskningsinstitut).Hoxby, C. M. (2000). The effects of class size on student achievement: New evidence from population variation. The Quarterly Journal of Economics, 115(4), 1239–1285.Hoxby, C. M. (1998). The Effects of Class Size and Composition on Student Achievement: New Evidence from Natural Population Variation. NBER Working Paper No. 6869.Jaciw, A. P. (2016). Applications of a Within‐Study Comparison Approach for Evaluating Bias in Generalized Causal Inferences From Comparison Groups Studies. Evaluation review, 40(3), 241–276.2773365410.1177/0193841X16664457Jepsen, C., & Rivkin, S. (2009). Class size reduction and student achievement the potential tradeoff between teacher quality and class size. Journal of human resources, 44(1), 223–250.Jepsen, C., & Rivkin, S. G. (2002). Class size reduction, teacher quality, and academic achievement in California public elementary schools
San Francisco: Public Policy Institute. of CA.Jones, S. (2016). How does classroom composition affect learning outcomes in Ugandan primary schools?
International Journal of Educational Development, 48, 66–78.Krassel, K. F., & Heinesen, E. (2014). Class‐size effects in secondary school. Education Economics, 22(4), 412–426.Leuven, E., & Løkken, S. A. (2017). Long Term Impacts of Class Size in Compulsory School. IZA Discussion Papers No. 10594.Leuven, E., Oosterbeek, H., & Rønning, M. (2008). Quasi‐experimental estimates of the effect of class size on achievement in Norway. The Scandinavian Journal of Economics, 110(4), 663–693.Lubienski, S. T., Lubienski, C., & Crane, C. C. (2008). Achievement differences and school type: The role of school climate, teacher certification, and instruction. American Journal of Education, 115(1), 97–138.Masci, C., Ieva, F., Agasisti, T., & Paganoni, A. M. (2016). Does class matter more than school? Evidence from a multilevel statistical analysis on Italian junior secondary school students. Socio‐Economic Planning Sciences, 54, 47–57.Murdoch, B., & Guy, P. W. (2002). Active learning in small and large classes. Accounting Education, 11(3), 271–282.Musau, L. M., & Migosi, J. A. (2013). Effect of class size on girls' academic performance in Science, Mathematics and Technology subjects. International Journal of Education Economics and Development, 4(3), 278–288.Rønning, M., Leuven, E., & Oosterbeek, H. (2008). Quasi‐experimental estimates of the effect of class size on achievement in Norway. REPEC Working Paper Series No. 9308.Sims, D. P. (2009). Crowding Peter to educate Paul: Lessons from a class size reduction externality. Economics of Education Review, 28(4), 465–473.Speas, C. (2003). Class size reduction program evaluation, 2000‐01. Report, Wake County Public School System, Raleigh, NC. Dept. of Evaluation and Research.Speas, C. (2003). Class‐Size Reduction Program Evaluation, 2001‐02. Report, Wake County Public School System, Raleigh, NC. Dept. of Evaluation and Research.Stecher, B. M., & Bohrnstedt, G. W. (2000). Class Size Reduction in California: Summary of the 1998‐99 Evaluation Findings. Unpublished Report, CSR Research Consortium.Tsai, L. T., & Yang, C. C. (2015). Hierarchical effects of school‐, classroom‐, and student‐level factors on the science performance of eighth‐grade Taiwanese students. International Journal of Science Education, 37(8), 1166–1181.

### 8.3 REFERENCES TO UNOBTAINABLE STUDIES

AchillesC. M. (1987). Some Analyses of Kindergarten Results in a Statewide Study of Class Size: Project STAR, Tennessee, 1985‐86. (Draft). UnpublishedBainH. P. et al. (1986). Small Class Size Once Again: An Experiment in Grade One, Metro‐Nashville Public Schools. UnpublishedBainH. P. & Jacobs, R. (1990) Project STAR Research Synopsis: The Effect of Reduced Class Size on Kindergarten Reading Readiness. UnpublishedBarberH. E. (1989). The effect of grade level on the relationship between class size and academic achievement. Dissertation Abstracts International
BudgeD. (1998). Cuts help children learn. TES: Times Educational Supplement
ButlerJ.M. & HandleyH.M. (1989). Differences in Achievement for First and Second Graders Associated with Reduction in Class Size. UnpublishedGilmanD. et al. (1987). PRIME TIME at North Gibson School Corporation: A Three Year Study. A Comprehensive Evaluation of Indiana's Program of State Supported Class Size Reduction. UnpublishedHuffmanW. (1999). Reducing Class Size Misses Mark. Staff General Research Papers
Indiana State Dept. of Public Instruction and Indianapolis Div of Curriculum
; (1983) Project Primetime: 1982‐83 Report. UnpublishedMinnesota State Dept. of Education; St Paul
. (1987). Class Size in Kindergarten through Grade Three: A Report to the Minnesota Legislature. UnpublishedNye, B. A. (1995) The Lasting Benefits Study: A continuing analysis of the effect of small class size in Kindergarten through Third Grade on Student Achievement Test Scores in Subsequent Grade Levels: 8th Grade Technical Report. Nashville: Tennessee State University, Center of Excellence for Research in Basic Skills.Nye, B.A. (1991). The Lasting Benefits Study: A Continuing Analysis of the Effect of Small Class Size in Kindergarten through Third Grade on Student Achievement Test Scores in Subsequent Grade Levels: Fourth Grade. Technical Report
Nashville: Tennessee State University, Center of Excellence for Research in Basic Skills.Nye, B.A. (1993) The Lasting Benefits Study: A Continuing Analysis of the Effect of Small Class Size in Kindergarten through Third Grade on Student Achievement Test Scores in Subsequent Grade Levels: Sixth Grade. Technical Report. Nashville: Tennessee State University, Center of Excellence for Research in Basic Skills.Nye, B.A. (1994) The Lasting Benefits Study: A continuing analysis of the effect of small class size in Kindergarten through Third Grade on Student Achievement Test Scores in Subsequent Grade Levels: Seventh Grade Technical Report. Nashville: Tennessee State University, Center of Excellence for Research in Basic Skills.SassiR.B. (2011). Got a raise? Thank your kindergarten teacher. Journal of the American Academy of Child & Adolescent Psychiatry
10.1016/j.jaac.2010.10.00321156263SwanE. et al. (1985) The Educational Effects of a State Supported Reduced Class Size Program. A Comprehensive Evaluation of Indiana's Project PRIME TIME at the North Gibson School Corporation. UnpublishedUnknown authors
. (2001). Class‐Size Reduction Helps Narrow Gaps, Study Finds. Education USA (Aspen Publishers Inc.)
ViaderoD. (2005), British Study Tracks Effect of Class Size. Education Week


### 8.4 ADDITIONAL REFERENCES

Anderson, L.W. (2000). Why should reduced class size lead to increased student achievement? In M. C.Wang & J. D.Finn (Eds.) How small classes help teachers do their best (pp. 3–24). Philadelphia, PA: Temple University Center for Research in Human Development and Education.Angrist, J.D. & V.Lavy (1999): “Using Maimonides' Rule to Estimate the Effect of Class Size on Scholastic Achievement”, The Quarterly Journal of Economics, Vol. 114, No. 2 (May, 1999), pp. 533–575.Angrist, J.D., & Pischke, J.S. (2009). Mostly Harmless Econometrics: An Empiricist's Companion
Princeton, NJ: Princeton University Press.Annevelink, E., Bosker, R. & Doolaard, S. (2004). Additional staffing, classroom processes and achievement. Retrieved April 28. 2014 from http://edu.fss.uu.nl/ord/fullpapers/Annevelink%20FP.doc
Bascia, N., & Fredua‐Kwarteng, E. (2008). Class size reduction: What the literature suggests about what works
Toronto: Canadian Education Association.Betts, J. R., & Shkolnik, J. L. (1999). The behavioral effect of variations in class size: the case of math teachers. Educational Evaluation and Policy Analysis, 21(2), 193–213.Biddle, B. J., & Berliner, D.C. (2002). Small class size and its effects. Educational Leadership, 59(5), 12–23.Blatchford, P., & Mortimore, P. (1994). The issue of class size for young children in schools: what can we learn from research?
Oxford Review of Education, 20(4), 411–428.Bourke, S. (1986). How smaller is better: some relationships between class size, teaching practices and student achievement. American Educational Research Journal, 23, 558–571.Donner, A., Piaggio, G. & Villar, J. (2001). Statistical methods for the meta‐analysis of cluster randomized trials. Statistical Methods in Medical Research 2001, 10(5), 325–38.10.1177/09622802010100050211697225Dustmann, C., Rajah, N. & van Soest, A. (2003). Class size, education and wages. The Economic Journal, 11(3), F99–F120.Dynarski, S., Hyman, J.M. & Schanzenbach, D.W. (2011). Experimental evidence on the effect of childhood investment on postsecondary attainment and degree completion. NBER Working Paper 17533.Egelson, P., Harman, P., Hood, A. & Achilles, C.M. (2002). How class size makes a difference
Greensboro, N.C.: Southeast Regional Vision for Education (SERVE).Ehrenberg, R. G., Brewer, D. J., Gamoran, A., & Willms, J. D. (2001). Class size and student achievement. Psychological Science and the Public Interest, 2(1), 1–30.10.1111/1529-1006.00326151146Finn, J. D. (2002). Small classes in American schools: Research, practice and politics. Phi Delta Kappan, 83(7), 551–560.Finn, J. D., & Achilles, C. M. (1990). Answers and questions about class size: A statewide experiment. American Educational Research Journal, 27(3), 557–577.Finn, J. D., & Achilles, C. M. (1999). Tennessee's class size study: Findings, implications, misconceptions. Educational Evaluation and Policy Analysis, 21(2), 97–109.Finn, J. D., Gerber, S. B., Achilles, C. M., & Boyd‐Zaharias, J. (2001a). The Enduring Effects of Small Classes. Teachers College Record, 103(2), 145–183.Finn, J.D., Gerber, S.B. & Boyd‐Zaharias, J. (2005). Small classes in the early grades, academic achievement, and graduating from high school. Journal of Educational Psychology, 97(2), p. 214–223.Fredriksson, P., Öckert, B., & Oosterbeek, H. (2013). Long‐term effects of class size. The Quarterly Journal of Economics, 128(1), 249–285;Glass, G. & Smith, M. L. (1979). Meta‐analysis of research on class size and achievement. Educational Evaluation and Policy Analysis, 1, 2–16.Graue, E., Hatch, K., Rao, K., & Oen, D. (2007). The wisdom of class size reduction. American Educational Research Journal, 44(3), 670–700.Grissmer, D. (1999). Conclusion: Class size effects: Assessing the evidence, its policy implications and future research agenda. Educational Evaluation and Policy Analysis, 21(2), 231–248.Hanushek, E. (1989). The impact of differential expenditures on school performance. Educational Researcher, 18(4), 45–62.Hanushek, E. (1999). Some findings from an independent investigation of the Tennessee STAR experiment and from other investigations of class size effects. Educational Evaluation and Policy Analysis, 21(2), 143–165.Hanushek, E. A. (2003). The failure of input‐based schooling policies. Economic Journal
113(1), F64–F98.Hattie, J. (2005). The paradox of reducing class size and improving learning outcomes. International Journal of Educational Research, 43, 387–425.Heckman, J.J. & Urzúa, S. (2010). Comparing IV with structural models: What simple IV can and cannot identify. Journal of Econometrics, 156, 27–37.2044037510.1016/j.jeconom.2009.09.006PMC2861784Hedges, L.V. (2007). Effect sizes in cluster‐randomized designs. Journal of Educational and Behavioral Statistics, 32(4), 341–370.Hedges, L. V. (2007). Meta‐analysis. In: Rao, C.R. (ed.). The Handbook of Statistics, 919–53. Amsterdam: Elsevier.Hedges, L. V., Laine, R. D., & Greenwald, R. (1994). Does money matter? A meta‐analysis of studies of the effects of differential school inputs on student outcomes. Educational Researcher, 23(3), 5–14.Hedges, L. V. & Olkin, I. (1985) Statistical methods for meta‐analysis. New York: Academic Press.Hedges, L. V. & Stock, W. (1983). The effects of class size: An examination of rival hypotheses. American Educational Research Journal, 20(1), 63–85.Higgins, J.P.T., & Green, S. (eds.) (2008). Cochrane Handbook for Systematic Reviews of Interventions. Wiley‐Blackwell.Higgins, J.P.T. & Green, S. (eds) (2011). Cochrane Handbook for Systematic Reviews of Interventions. Version 5.1.0 [updated March 2011]. Wiley‐Blackwell The Cochrane Collaboration. Available from www.cochrane‐handbook.org.Higgins, J.P., Thompson, S.G., Deeks, J.J., & Altman, D.G. (2003). Measuring inconsistency in meta‐analyses. British Medical Journal, 327 (7414), 557–60.10.1136/bmj.327.7414.557PMC19285912958120Holmlund, H., & Sund, K. (2005). Is the gender gap in school performance affected by the sex of the teacher?
Swedish Institute for Social Research (SOFI), Stockholm University, working paper 5/2005.Houtveen, A.A.M., Booij, N., de Jong, R. & van de Grift, W.J.C.M. (1999). Adaptive instruction and pupil achievement. School Effectiveness and School Improvement, 10(2), 172–192.Hyde, J. S., Fennema, E., & Lamon, S. J. (1990). Gender differences in mathematics performance: A meta‐analysis. Psychological Bulletin, 107(2), 139–155.213879410.1037/0033-2909.107.2.139Hyde, J. S., & Linn, M. C. (1988). Gender differences in verbal ability: A meta‐analysis. Psychological Bulletin, 104(1), 53–69.Konstantopoulos, S. (2009). Effects of teachers on minority and disadvantaged students' achievement in the early grades. Elementary School Journal, 110 (1), 92–113.Krueger, A. B. (1999). Experimental estimates of education production functions. Quarterly Journal of Economics, 114(2), 497–532.Krueger, A. B. (2003). Economic considerations and class size. The Economic Journal, 113 (February), F34–F63.Lipsey, M. W., & Wilson, D. B. (2001). Practical meta‐analysis. Applied Social Research Methods Series, v. 49.Molnar, A., Smith, P., Zahorik, J., Palmer, A., Halbach, A., et al. (1999). Evaluating the SAGE Program: A pilot program in targeted pupil‐teacher reduction in Wisconsin. Educational Evaluation and Policy Analysis, 21, 165–178.Molnar, A., Smith, P., Zahorik, J., Palmer, A., Halbach, A. & Ehrle, K. (2000). Wisconsin's Student Achievement Guarantee in Education (SAGE) Class Size Reduction Program: Achievement Effects, Teaching, and Classroom implications. In M. C.Wang & J. D.Finn, How small classes help teachers do their best. (pp. 227–277). Philadelphia: Temple University Center for Research in Human Development and Education.Muenning, P. & Woolf, S.H. (2007). Health and economic benefits of reducing the number of students per classroom in US primary schools. American Journal of Public Health.
97, p. 2020–2027.1790143010.2105/AJPH.2006.105478PMC2040354Nye, B., Hedges, L. V., & Konstantopoulos, S. (1999). The long‐term effects of small classes: A five‐year follow‐up of the Tennessee class size experiment. Educational Evaluation and Policy Analysis, 21(2), 127–142.OECD
(2010). PISA 2009 results: Overcoming social background ‐ equity in learning opportunities and outcomes (Volume II). Retrieved from 10.1787/9789264091504-en
OECD
(2012). Education indicators in focus. OECD 2012/09 (November)Piaget, J. (2001) The psychology of intelligence. New York, NY: Routledge.Rivkin, S. G., Hanushek, E.A., & Kain, J. F. (2005). Teachers, schools, and achievement. Econometrica, Vol. 73, No. 2 (March, 2005), 417–458
Schanzenbach, D.W. (2007). What have researchers learned from Project STAR? Brookings Papers on Education Policy
Washington, DC: Brookings Institution.Shapson, S. M., Wright, E. N., Eason, G., & Fitzgerald, J. (1980). An experimental study of the effects of class size. American Educational Research journal, 17(2), 141–152.Smith, M.L., & Glass, G. V. (1980). Meta‐analysis of research on class size and its relationship to attitudes and instruction. American Educational Journal, 17(4), 419–433.Smith, P., Molnar, A., & Zahorik, J. (2003). Class‐size reduction: A fresh look at the data, Educational Leadership, (61), 72–74.Stockford, S.M. (2009). Meta‐analysis of intraclass correlation coefficients from multilevel models of educational achievement. Ph.D. Thesis, Arizona State University, page 1–126.Stoet, G., & Geary, D. C. (2013). Sex differences in mathematics and reading achievement are inversely related: Within‐ and across‐nation assessment of 10 years of PISA data. PLoS ONE, 8(3).10.1371/journal.pone.0057988PMC359632723516422Vygotsky, L. S. (1978). Mind in society: The development of higher psychological processes
Cambridge, MA: Harvard university press.Willms, J.D. & Somers, M. (2001). Family, Classroom, and School Effects on Childrens Educational Outcomes in Latin America. School Effectiveness and School Improvement: An International Journal of Research, Policy and Practice, 12 (4), 409–445.Wilson, V. (2002). Does small really make a difference? A review of the literature on the effects of class size on teaching practice and pupils' behaviour and attainment. The Scottish Council for Research in Education (SCRE) Research Report No 107.WordE, JohnsonJ, BainHP, FultonDB, ZahariasJB, LintzMN, AchillesCM, FolgerJ, BredaC. (1990) Student/Teacher Achievement Ratio (STAR): Tennessee's K—3 class‐size study (Tennessee State Department of Education, Nashville).

## 9. Information about this review

### 9.1. REVIEW AUTHORS

**Table:** 

**Lead review author:**	
Name:	Trine Filges
Title:	Senior Researcher
Affiliation:	SFI‐Campbell
Address:	Herluf Trollesgade 11
City, State, Province or County:	Copenhagen
Postal Code:	1052
Country:	Denmark
Phone:	45 33480926
Email:	tif@sfi.dk
**Co‐authors:**	
Name:	Christoffer Scavenius Sonne‐Schmidt
Title:	Researcher
Affiliation:	SFI‐Campbell
Address:	Herluf Trollesgade 11
City, State, Province or County:	Copenhagen
Postal Code:	1052
Country:	Denmark
Phone:	45 33480971
Email:	css@sfi.dk
Name:	Anne Marie Klint Jørgensen
Title:	Librarian/Information Specialist
Affiliation:	SFI‐Campbell
Address:	Herluf Trollesgade 11
City, State, Province or County:	Copenhagen
Postal Code:	1052
Country:	Denmark
Phone:	45 33480868
Email:	amk@sfi.dk

### 9.2 ROLES AND RESPONSIBILITIES

Below is listed who is responsible for the following areas:

- Content: Christoffer Scavenius Sonne‐Schmidt
- Systematic review methods: Trine Filges
- Statistical analysis: Trine Filges Christoffer Scavenius Sonne‐Schmidt
- Information retrieval: Anne Marie Klint Jørgensen

### 9.3 SOURCES OF SUPPORT

SFI Campbell.

### 9.4 DECLARATIONS OF INTEREST

None.

### 9.5 PLANS FOR UPDATING THE REVIEW

We plan to update the review with a frequency of two years. Trine Filges will be responsible.

### 9.6 AUTHOR DECLARATION

**Authors' responsibilities**

By completing this form, you accept responsibility for maintaining the review in light of new evidence, comments and criticisms, and other developments, and updating the review at least once every five years, or, if requested, transferring responsibility for maintaining the review to others as agreed with the Coordinating Group. If an update is not submitted according to agreed plans, or if we are unable to contact you for an extended period, the relevant Coordinating Group has the right to propose the update to alternative authors.

**Publication in the Campbell Library**

The Campbell Collaboration places no restrictions on publication of the findings of a Campbell systematic review in a more abbreviated form as a journal article either before or after the publication of the monograph version in *Campbell Systematic Reviews*. Some journals, however, have restrictions that preclude publication of findings that have been, or will be, reported elsewhere, and authors considering publication in such a journal should be aware of possible conflict with publication of the monograph version in *Campbell Systematic Reviews*. Publication in a journal after publication or in press status in *Campbell Systematic Reviews* should acknowledge the Campbell version and include a citation to it. Note that systematic reviews published in *Campbell Systematic Reviews* and co‐registered with the Cochrane Collaboration may have additional requirements or restrictions for co‐publication. Review authors accept responsibility for meeting any co‐publication requirements.

**I understand the commitment required to update a Campbell review, and agree to publish in the Campbell Library. Signed on behalf of the authors**:

**Form completed by: Trine Filges Date: 10 October 2018**

## 10 Characteristics of included studies

### 10.1 NON‐STAR STUDIES

**Table:** 

**Study**	**Used/reason not used in data synthesis**	**Treatment year (s)**	**Country**
Achilles, 1995	Too high risk of bias on the confounding item	1991‐1994	USA
Akerhielm, 1995	Too high risk of bias on the confounding item	1988	USA
Angrist, 1999	Too high risk of bias on the confounding item	1991	Israel
Angrist, 2014	Too high risk of bias on the confounding item	2009‐2011	Italy
Annevelink, 2004	Too high risk of bias on the confounding item	2000‐2001	NL
Blatchford, 2002	Too high risk of bias on the selective reporting item	1996/97	UK
Blatchford, 2003a	Collection of results from British Class Size Study. Cannot assess RoB as not enough information is provided. Only one effect size reported (but not number of observations used, so cannot calculate standard errors), the rest reported as NS or a narrative description such as ‘there was found to be an effect’.	1996/97 and maybe 1997/98	UK
Blatchford, 2003b	No results reported other than graphs without CI.	1996‐1999	UK
Bonesrønning, 2003	Too high risk of bias on the confounding item	1998‐2000	Norway
Boozer, 1995	Too high risk of bias on the confounding item	1988	USA
Boozer, 2001a	Too high risk of bias on the confounding item	1985‐1990	New Zealand
Boozer, 2001b	Too high risk of bias on the confounding item	1988	USA
Borland, 2005	Too high risk of bias on the confounding item	1990	USA
Bosworth, 2014	Not enough information provided to calculate standard errors	2001‐2002	USA
Bressoux, 2009	Used in data synthesis	1991‐1992	France
Breton, 2012	Too high risk of bias on the confounding item	1997	Columbia
Burde, 1990	Too high risk of bias on the confounding item	1988	USA
Carpenter, 2003	Too high risk of bias on the selective reporting item	1996/1997	UK
Chargois, 2008	Too high risk of bias on the confounding item	2007	USA
Clanet, 2010	Only report the significance level and only sign of the effects that are significant	2001‐2002	France
Costello, 1992	Too high risk of bias on the confounding item	1995	USA
Dee, 2011	Subject specific test score, may be mathematics, reading, science or history but not specified. First difference between subjects is outcome	1988	USA
Dennis, 1986	Too high risk of bias on the confounding item	1985‐1986	USA
Dharmadasa, 1995	Too high risk of bias on the confounding item	1989	Sri Lanka
Dieterle, 2013	Only have data at required level for two of three grades and do not provide useable separate results	2003‐2004	USA
Dobbelsteen, 2002	Too high risk of bias on the confounding item	1994/1995	NL
Ecalle, 2006	Used in data synthesis	2002‐2003	France
Galton, 2012	Too high risk of bias on the confounding item	2004‐2008	Hong Kong
Gerritsen, 2017	Used in data synthesis	1994‐2005	NL
Gilman, 1988a	Too high risk of bias on the confounding item	1984‐1988	USA
Gilman, 1988b	Too high risk of bias on the confounding item	1985	USA
Haenn, 2002	Too high risk of bias on the confounding item	1994/1995 to probably 2001	USA
Hallinan, 1985	Too high risk of bias on the confounding item	Not reported	USA
Hirschfeld,2016	Too high risk of bias on the confounding item	2016	USA
Hojo, 2011	Too high risk of bias on the confounding item	2007	Japan
Hojo, 2013	Too high risk of bias on the other bias item	2003	Japan
Hudson, 2011	Used in data synthesis	1990	USA
Iacovou, 2002	Too high risk of bias on the confounding item	1965, 1969 and 1974	UK
Iversen, 2013	Too high risk of bias on the confounding item	2003‐2004	Norway
Jakubowski, 2006	Too high risk of bias on the confounding item	2002‐2004	Poland
Konstantopoulos, 2014	Too high risk of bias on the confounding item	2001	Greece
Konstantopoulos, 2016	Too high risk of bias on the confounding item	2003 and 2007	Cyprus
Konstantopoulos, 2016	Too high risk of bias on the confounding item	2011	Multiple^1^
Krueger, 2002	Too high risk of bias on the confounding item	1998‐1999	Sweden
Lavy, 2001	Too high risk of bias on the confounding item	1991	Israel
Levin, 2001	Too high risk of bias on the confounding item	1994/1995	NL
Li, 2015	Too high risk of bias on the confounding item	2011	Multiple^2^
Li, 2017	Too high risk of bias on the confounding item	2011	Multiple^1^
Lindahl, 2005	Too high risk of bias on the confounding item	1998	Sweden
Ma, 2006	Too high risk of bias on the confounding item	1994/1995	NL
Maier, 1997	A Regular classroom refers to a classroom with one teacher. Most regular classrooms have 15 or fewer students, but a few exceed 15. A 2‐Teacher Team classroom is a class where two teachers work collaboratively to teach as many as 30 students. A Shared‐Space classroom is a classroom that has been fitted with a temporary wall that creates two teaching spaces, each with one teacher and about 15 students. A Floating Teacher classroom is a room consisting of one teacher and about 30 students, except during reading, language arts, and mathematics instruction when another teacher joins the class to reduce the ratio to 15:1. Only analyse effect of type of classroom within SAGE schools.	1995‐1996	USA
Maples, 2009	Too high risk of bias on the confounding item	2006‐2007	USA
McGiverin, 1989	Too high risk of bias on the confounding item	1984‐85	USA
Merritt, 2011	Too high risk of bias on the other bias item	2010	USA
Milesi, 2006	Used in data synthesis	1998‐1999	USA
Molnar, 1998	See Maier, 1997	1997‐1998	USA
Molnar, 1999a	See Maier, 1997	1998‐1999	USA
Molnar, 1999b	See Maier, 1997	1996‐1998	USA
Molnar, 2001	See Maier, 1997	2000‐2001	USA
Moshoeshoe, 2015	Too high risk of bias on the confounding item	2000	Lesotho
Munoz, 2001	Used in data synthesis	1999‐2000	USA
Murdoch, 1986	Only report p values from a multivariate model (8 outcomes) with CS, age, gender and school, separated by grade	1984‐1985	USA
Maasoumi, 2005	No method/results we can use (first or second order stochastic dominance tests)	1988	USA
Nandrup, 2016	Too high risk of bias on the confounding item	2009/2010‐2011/2012	Denmark
NICHD, 2004	Not enough information provided to calculate standard errors	1990‐1991	USA
Otsu, 2015	Relevant results are presented graphically and no ES and SE can be extracted. (Uses selected data of Angrist and Lavy (1999); schools with either one or two classes in grade 4)	1991	Israel
Pollard, 1995	Too high risk of bias on the confounding item	1990‐1992 and 1996‐1997	USA
Pong, 2001	Too high risk of bias on the confounding item	1994‐1995	Multiple^3^
Sanogo, 1994	Reproduction of STAR and Indiana PRIME Time results (Word et al. 1990 and Tillitsky, Gilman, Mohr, and Stone, 1988). Do not report what type of classes are included in the PRIME Time results	1985‐1989 and 1984‐1987	USA
Shapson, 1980	They do not report outcomes for all groups for all years, so we cannot determine the effect of being randomized to one of the four arms.	1977‐1979	Canada
Tienken, 2009	Too high risk of bias on the confounding item	2001‐2006	USA
Tillitsky, 1988	Too high risk of bias on the confounding item	1984‐1987	USA
Uhrain, 2016	Too high risk of bias on the confounding item	2012‐2013	USA
Urquiola, 2006	Too high risk of bias on the other bias item	1993	Bolivia
Watson, 2016	Too high risk of bias on the confounding item	2008‐2012	Australia
Wenfan, 2005	Too high risk of bias on the confounding item	1998‐1999	USA
West, 2006	Too high risk of bias on the confounding item	1994‐1995	Multiple^4^
Wiermann, 2005	Difference between mathematics and physics test scores (the chemistry/biology and the reading/biology differences scores 5)	2000	Germany
Wößmann, 2006	Too high risk of bias on the confounding item	1994‐1995	Multiple^5^
Wößmann, 2003	Too high risk of bias on the confounding item	1994‐1995	Multiple^6^
Wößmann, 2005a	Too high risk of bias on the confounding item	1995	Japan and Singapore
Wößmann, 2005b	Too high risk of bias on the confounding item	1995	Multiple^5^

1: Austria, Lithuania, Croatia, Malta, Czech Republic, Portugal, Denmark, Romania, Germany, Slovak Republic, Hungary, Slovenia, Italy and Spain

2: Austria, Lithuania, Croatia, Malta, Czech Republic, Portugal, Denmark, Romania, Germany, Slovak Republic, Hungary, Slovenia, Italy, Spain, Hong Kong, Singapore, Japan and Chinese Taipei

3: USA, Canada, Australia, France, Germany, Iceland, Singapore, Korea and Hong Kong.

4: Belgium Fr., Canada, Czech Rep., Franc, Greece, Iceland, Portugal, Romania, Singapore, Slovenia and Spain.

5: USA, Australia, Belgium (Fl), Belgium (Fr), Canada, Czech Rep., France, Greece, Hong Kong, Iceland, Japan, Korea, Portugal, Romania, Scotland, Singapore, Slovenia and Spain.

6: USA, Austria, Belgium, Denmark, England and Scotland, France, Germany, Greece, Iceland, Ireland, the Netherlands, Norway, Portugal, Spain, Sweden, and Switzerland.

### 10.2 STAR STUDIES

**Table:** 

**Study**	**Used/not used in data synthesis**	**Notes**
Achilles, 1993a	Not used in data synthesis	STAR. Reproduction of the results in Word et al. 1990 (significance levels from analysis‐of‐variance models) and further results on various subgroups (for example entering STAR in grade 1 or results on retained/not retained etc.)
Achilles, 1993b	Provide effect sizes from other studies.	Grade 4 results reproduced from Finn 1989 and Grade 5 results reproduced from Nye, 1992 and judged 5 in the other risk of bias data item. Separate results for S vs R and R vs RA
Balestra, 2014	Provide no results that can be used in data synthesis	STAR (quantile regression) only reported for kindergarten and 1. grade and Lasting Benefit Study reanalysis of graduation from high school (not an outcome of this review)
Bingham, 1994	Provide no results that can be used in data synthesis	STAR reanalysis. No useful data provided (only means)
Chetty, 2011	Provide no results that can be used in data synthesis	STAR no useful outcomes provided. Test score as the average mathematics and reading percentile rank score attained in the student's year of entry into the experiment is only relevant outcome reported for this review.
Ding, 2005	Provide no results that can be used in data synthesis	STAR reanalysis. None of the analyses can be used for this review. Analyses the effect of each class size in the range 12‐28 relative to 22. Further report results from regressions where class size is interacted with several covariates.
Ding, 2010	Not used in data synthesis	STAR reanalysis. Structural equation model. Effects of number of years (and sequence) treated
Ding, 2011	Provide no results that can be used in data synthesis	STAR reanalysis. Uses KG data only. Do not separate R and RA. Regression with small class interacted with covariates
Doulgas, 1989	Provide no results that can be used in data synthesis	Report percent of variance accounted for by factors (among others class size) affecting mean class achievement
Finn, 1989	Provide effect sizes for grade 4. Too high risk of bias (other bias item)	Report means, SD's and effect sizes for grade 4
Finn, 1990a	Provide results and data that can be used in data synthesis (although only for grade 1)	Report effect sizes, comparing small classes to the mean of regular and regular with aide. Report means for each of the three conditions and report standard deviations based on students in regular classes. Report total number of students and number of classes in the three conditions. Results divided on location (inner‐city, rural etc.) also provided. A growth analysis of students participating in the same classroom arrangement for both years and who had complete data (35%) performed but is given 5 on incomplete data
Finn, 1990b	Too high RoB	STAR reanalysis for those in same class arrangement for 3 years (K‐2. grade) Judged 5 in RoB (incomplete outcome data)
Finn, 1998	Provide effect sizes from other studies.	Reporting of effect sizes (KG‐3) from Nye, 1993 and Nye, 1992/1994.
Finn, 1999	Provide results from the LBS technical reports grade 4‐7. Could use results for grade 6 and 7 as the technical reports for these grades are not available (scores 5 on the other risk of bias item though). Otherwise no results are provided that can be used in data synthesis.	Reporting of effect sizes (KG‐3) from Finn, 1998 (who reports effect sizes from other studies). Reporting of effect sizes for grades 4, 5, 6 and 7 from Finn et al. 1989; the LBS Technical Reports: Nye et al., 1992; Nye et al., 1993 (study not available) and Nye et al., 1994 (study not available). The result for 6. Grade is to a large extent different from the result reported in Finn, 2001. Calculate Grade Equivalence effect sizes (not an outcome of this review) and behaviour effect sizes
Finn, 2001	Provide effect sizes for grade KG‐3 and grade 4, 6 and 8. Grade 4, 6 and 8 judged 5 on the other risk of bias item.	Reanalysis of STAR and LBS. Report effect sizes, comparing small classes to regular classes. Do not report whether classes of trained teachers or out‐of‐range classes are excluded or not. Report the total number of students used, though not per grade for KG‐3. Results are slightly different than the results reported in Folger 1989 for KG‐3 grade and in Finn 1989 for 4. Grade and to a large extent different from the result reported in Finn 1999 for grade 6. LBS results judged 5 in RoB (other bias)
Finn, 2005	Too high RoB	Analysis of high school graduation. Judged 5 in RoB (other bias)
Folger, 1989	Provide effect sizes for grade KG‐3. Used in data synthesis	It is most likely small classes compared to regular classes. Includes the teachers receiving STAR training although it is unclear how many teachers were trained. According to Word (1990) and and this study, 57 teachers in grade 2 from 13 randomly chosen schools and another 57 teachers in grade 3 received Project STAR training. According to Word et al. (1994) p. 73, 67 teachers received training in grade 2 and on page 117 it is stated that all teachers (57 teachers and 57 classes) from 13 schools received training in 2. Grade and all teachers from the same 13 schools (57 classes) received training in 3. Grade. The distribution of class type is not constant in these 13 schools; in 2. Grade it is reported there are 21 S, 19 R and 17 RA and in 3. Grade there are 25 S, 15 R and 17 RA. According to Finn et al. (2007): Second, during the summer between grade 1 and grade 2 (summer 1987), a three‐day training course was given to 54 second‐grade teachers (out of 340) from 15 STAR schools. The training was the same for all 54 teachers, since the assignment to class types had not yet been made. Excludes out‐of‐range classes although unclear how they are defined. Uses a range of 21‐28 students for regular classes (original the range was 22‐25. Analysis of STAR includes the 67 teachers receiving STAR training (although reports that it is 57 teachers in grade 2 from 13 randomly chosen schools and another 57 teachers in grade 3) and excludes out‐of‐range classes, results also shown in Word (1990 and 1994).
Hanushek, 1999	Provide effect sizes for grade KG‐3. Used in data synthesis	Compares small classes to the mean of regular and regular with aide. Do not explicitly report the numbers used for analysis but probably include the classes of trained teachers and out‐of‐range classes. Report the numbers with achievement data.
Harvey, 1994	Too high RoB	STAR data, only retainees used (reanalysis). Judged 5 in RoB (other bias)
Jackson, 2013	Provide no results that can be used in data synthesis	Reanalysis uses only kindergarten and 1. Grade and a composite z‐score (average of mathematics, reading and word scores).
Jacobs, 1987	Provide no results that can be used in data synthesis and too high RoB	Is judged 5 in RoB (incomplete outcome data) Results in table 3, 4 and 5 (for three different outcomes) have main effect for class type (not small separated out). Cross tabulation of the 3 outcomes in table 6, 7 and 8 but only raw totals and percent scoring low/middle/high and other tables subdivided on several covariates. Scores for small class size are given in fig. 20 and 38, but no standard deviation
Konstantopoulos, 2008	Provide no results that can be used in data synthesis	STAR reanalysis. Quantile regression with covariates (gender, ethnicity and SES). Whether achievement distribution used is taken over Treated/Control or Treated+Control is not reported
Konstantopoulos, 2009	Provide no results that can be used in data synthesis and too high RoB	Reanalysis of STAR and Lasting Benefits Study data. ITT and IV analyses (same quantile regression effect of 3. grade treatment in 4‐8 grade separately), also available, and a dose analysis (judged 5 in RoB, other bias). Unclear what their achievement distribution is.
Konstantopoulos, 2011	Not used in data synthesis. Too high RoB	Reanalysis of STAR data. ITT analysis. Each school treated as an individual RCT ‐ effect size from linear regression (with small class and regular with aide compared to regular classes in the same model, cannot separate teacher effect from treatment effect in schools with only one small class and/or only one regular class (approximately 43% of schools had only one small class and 81% had only one small and/or one regular class)) ‐ overall mean calculated by inverse variance weighted random effects model. Judged 5 in RoB (other bias)
Krueger, 1999	Provide no results that can be used in data synthesis	STAR reanalysis. Average percentile scores in mathematics, reading and word (not shown separately) used for analysis.
Krueger, 2001a	Too high RoB and provide no results that can be used in data synthesis	Same analyses as Krueger & Whitmore, 2001, with updated data (in addition they only report weighted averages of percentages and do not report the numbers used for analysis, so results cannot be used).
Krueger, 2001b	Too high RoB and provide no results that can be used in data synthesis	STAR follow up. Analysis of scores on two high school entrance exams is judged 5 in RoB (other bias). Analysis of entrance exam taken or not is also available (not an outcome of this review)
Mckee, 2010	Not used in data synthesis	STAR reanalysis. Only KG and merge R and RA. OLS w/wo school FE controlling for teachers with fewer than three years of experience and teachers with an advanced degree, and for the student's race‐ethnicity, gender, age, special education status, whether or not they are repeating kindergarten, attendance record, and subsidized lunch eligibility. Specifications that do not include school fixed effects also include indicators for community type (suburban, rural, urban, and inner‐city). Transform test scores to have zero mean and SD of one
McKee, 2015	Not used in data synthesis	STAR reanalysis. Use only KG and pool R and RA classes and transform test scores to have zero mean and SD of one and include covariates
Mosteller, 1995	Provides results from other articles only	Provides results from other articles: Finn, J.D., and Achilles, C.M. Answers and questions about class size: A state‐wide experiment. American Educational Research Journal (1990) 27, 3:557–77, Table 5. And Word, E., Johnston, J., Bain, H.P., et al. Student/Teacher Achievement Ratio (STAR): Tennessee's K‐3 class size study, Nashville: Tennessee Department of Education, Figures 1 and 2.
Nye, 1992	Too high RoB. Not used in the data synthesis	Technical report for fifth grade of the Lasting Benefits Study. Scores 5 on the incomplete outcome data item (and other risk of bias)
Nye, 1993	Results for KG‐3 grade used in the data synthesis. Results for grade 4 and 5 are reproduced from Finn, 1989, Nye et al., 1991 (not available) and Nye, 1992.	Results for grade KG‐3 are obtained comparing small classes to the mean of regular and regular with aide, also divided on white/minority (same analysis and results as in Nye, 1992/1994). Excludes the 67 teachers receiving STAR training (it is 67 teachers according to the technical report (Word 1994) page 73 (text and table IV‐12 providing the numbers used for analysis) but on page 117 and 192 and according to Word (1990) and Folger & Breda (1989) it was 57 teachers in grade 2 from 13 randomly chosen schools and another 57 teachers in grade 3) and includes out‐of‐range classes. Numbers used for KG and 1 grade are 5734 and 5905. Do not report the numbers used for 2. and 3. Grade analyses. Report effect sizes for grade 4 and 5 comparing small to regular. Grade 4 results reproduced from Finn, 1989 and Nye et al., 1991 (not available) and Grade 5 results reproduced from Nye, 1992.
Nye, 1992/1994	Results for KG‐3 grade used in the data synthesis. Results for grade 4 and 5 are reproduced from Finn, 1989, Nye et al., 1991 (not available) and Nye, 1992.	Compares small classes to the mean of regular and regular with aide, also divided on white/minority (same analysis and results as in Nye, 1993). Excludes the 67 teachers receiving STAR training (it is 67 teachers according to the technical report (Word, 1994) page 73 (text and table IV‐12 providing the numbers used for analysis) but on page 117 and 192 and according to Word (1990) and Folger & Breda (1989) it was 57 teachers in grade 2 from 13 randomly chosen schools and another 57 teachers in grade 3) and includes out‐of‐range classes. Numbers used for KG and 1 grade are 5734 and 5905. Do not report the numbers used for 2. and 3. Grade analyses. Report effect sizes for grade 4 and 5 comparing small to regular. Grade 4 results reproduced from Finn, 1989 and Nye et al., 1991 (not available) and Grade 5 results reproduced from Nye, 1992.
Nye, 2000a	Provide no results that can be used in data synthesis	Hierarchical linear regression model separate for each grade and reading and mathematics including gender, SES and minority status, interaction of small class and gender, SES and minority respectively and (three way) interaction of small class, gender and minority and a similar analysis with three way interaction: small class, gender and SES. Coefficient estimates with stars. Cannot be used. Also available are effect sizes (d's) separated by white/minority and high/low SES and ES's by gender within race (white/minority) and SES (high/low) (but do not report number of observations used so we cannot calculate standard errors).
Nye, 2000b	Provide no results that can be used in data synthesis	Three analyses (two separate models for treatment as received (a two level and a three level model) and a three level model for treatment as assigned) each comparing regular to small and (for the two level model only) regular with aide (in the three level model regular and regular with aide are assumed to be the same).. Analysis separate for each grade and reading and mathematics including gender and SES, interaction of small class and gender (although coefficients shown report they are for gender and minority interaction?), geographic location of school, teacher experience, school SES and school minority. Effect size estimates with stars (indicating significance level).
Nye, 2001a	Too high RoB	STAR follow up (9. Grade) Two analyses: 1) Students who participated at least 1 year and was part of the trial in 3. Grade; 2) students participating all 4 years. Judged 5 in RoB (incomplete outcome data)
Nye, 2001b	Provide no results that can be used in data synthesis and too high RoB	STAR reanalysis, grade 1‐3, special sample: it is unclear whether some of the students in the control group they use have spent some years in a small class (the control group is characterised by: small class in some or no grades, see table 1). In the analysis for each grade they include only treated who were in small class for that grade and all previous grades. Unclear whether the control group is required to have been in the experiment for all previous grades but probably not, the total sample size increases from grade 1 to 3 whereas the treated group considerably decreases. Grade 2 and 3 judged 5 in RoB (incomplete outcome data) and it is not possible to calculate standard errors (so results for grade 1 cannot be used either)
Nye, 2002	Provide no results that can be used in data synthesis	Analysis separate for each grade and reading and mathematics including gender, SES, minority status, low achiever (below median within classes at end of kindergarten) and interaction of small class and low achiever. Coefficient estimates with stars (indicating significance level). Cannot be used. Table 1 provides effect sizes (d's) separated by low/high achievers (relative within class at end of kindergarten) (but do not report number of observations used so we cannot calculate standard errors).
Prais, 1996	Provides results from other articles and otherwise provide no results that can be used in data synthesis	STAR reanalysis. ‘Reproduction of the Technical reports (Word, 1994) (mathematics/reading average scores) table p. 47/47 and figure p. 54/53, figure p.65/64, figure p.78/77 and figure p. 92/93 and (own) calculation of yearly value added and 3 years average of value added.
Schanzenbach, 2007	Not used in data synthesis	ITT reanalysis using composite mathematics and reading. Also provide results for composite test score for 4, 5, 6, 7 and 8 grade.
Shin, 2012	Provide no results that can be used in data synthesis	STAR reanalysis using new comers each year only and separate by race. Several analyses: 1) ITT (by IV, random assignment as IV for actual class size, i.e. multiple CS reduction levels and include new students each year also) separated by race and controlling for race and the race difference in same equation; 2) same as 1) but in a structural simultaneous model. They investigate whether there is school‐level confounding, by comparing a model with school‐level fixed‐effects to a model without fixed‐effects (comparison of 3L ITT and 2L ITT in table 2 and 3)
Shin, 2011	Provide no results that can be used in data synthesis	Same analyses as Shin, 2012, but not separated by race. They investigate whether there is school‐level confounding, by comparing a model with school‐level fixed‐effects to a model without fixed‐effects (comparison of 3L ITT and 2L ITT in table 4 and 5)
Sohn, 2015	Too high RoB	LBS reanalysis (CTBS data) 4., 6. and 8. grade. Analyse number of years in small class and divide on ‘effective’ (i.e. significant difference) and ineffective schools (also show total). Results cannot be used
Word, 1990 and 1994	Final report for grade KG‐3. Only report significance levels reported (can not be used). Summary of relevant results (effect sizes) from Folger, 1989 can be used.	Summary of original results. Only report significance levels reported (analysis‐of‐variance model results can not be used as they are only reported as a summary of the analyses showing significance levels (.05, .01, .001, all levels are <=). Provide effect sizes for KG‐3 grade from an analysis conducted by Folger (also provided in Folger & Breda, 1989).

### 10.3 STAR STUDENTS AND CLASSES

**Table 10.3.1:** Number of students and transfers in percent, Kindergarten to 1. Grade

**1. Grade**							
**Kindergarten**		Total number	Drop out	Small	Regular	Regular/aide	
	Small	1900	26	68	3	3	100
	Regular	2194	30	6	34	30	100
	Regular/aide	2231	29	5	34	32	100
	Total	6325	29	24	25	22	100
	Transfer to 1 G	4515					

**Table 10.3.2:** Number of students and transfers in percent, 1. Grade to 2. Grade

**2. Grade**							
**1. Grade**		Total number	Drop out	Small	Regular	Regular/aide	
	Small	925	23	75	1	1	100
	Regular	2584	28	6	58	8	100
	Regular/aide	2320	26	2	5	67	100
	Total	6829	26	24	24	26	100
	Newcomers in 1. Grade	2314					
	Transfer to 2. Grade	5049					

**Table 10.3.3:** Table Number of students and transfers in percent, 2. Grade to 3. Grade

**3. Grade**							
**2. Grade**		Total number	Drop out	Small	Regular	Regular/aide	
	Small	2016	19	78	2	2	100
	Regular	2329	23	7	64	7	100
	Regular/aide	2495	21	2	3	74	100
	Total	6840	21	26	23	30	100
	Newcomers in 2. Grade	1791					
	Transfer to 3. Grade	5413					

*Source: Nye, Hedges & Konstantopoulos (2000b) and own calculations*

**Table 10.3.4:** Total transfers

	**Number**	**Per cent**
Total drop out	5017	43
Total movers	2843	25
Total stayers	3740	32
Total STAR students	11600	100

**Table 10.3.5:** Distribution of STAR classes by grade (Kindergarden‐3) by designation S (Small), R (Regular), and RA (Regular with Aide)

	**Class size**	**K (number of classes)**			**1 (number of classes)**			**2 (number of classes)**			**3 (number of classes)**		
		S	R	RA	S	R	RA	S	R	RA	S	R	RA
B	11										2		
	12	8			2			3			2		
A	13	19			14			16			15		
	14	22			18			27			17		
	15	23		1	31			32			31		
	16	31	4		16	1		29	1		31		1
	17	24	4	1	33	1		19			27		
B	18		1	2	6	2		6			10	1	
	19		7	6	3	4	3	1	3	3	5		4
	20		6	6	1	10	6		2	1		9	13
	21		14	12		18	18		7	11		11	12
C	22		20	20		27	15		23	21		13	16
	23		16	21		19	20		20	21		10	14
	24		19	14		16	11		22	25		15	14
	25		6	6		7	9		9	15		16	15
B	26		4	3		5	9		6	7		5	12
	27		1	6		2	4		4	1		5	8
	28			1		1	2		1			2	6
	29					1	2		2	2		2	2
	30					1	1						
Total as reported		127	99	99	124	115	100	133	100	107	140	90	107
Total as calculated		127	102	99	124	115	100	133	100	107	140	89	117
Too small^1^		8	36	28	2	36	27	3	13	15	4	21	30
Too large^2^		0	5	10	10	10	18	7	13	10	15	14	28
Total		8	41	38	12	46	45	10	26	25	19	35	58
Out‐of range as reported (sum of B's)		8	33	36	12	44	45	10	25	25	19	35	57
Per cent too large for S and too small for R and RA^3^		0	35	28	8	31	27	5	13	14	11	24	26

A = range for (S); B = “out of range”; C = range for both (R) and (RA) classes. SOURCE: Achilles (1999) as reported in Finn et al. (2007). 1: <13 for S and <22 for R and RA. 2: >17 for S and >25 for R and RA. 3: >17 for S and <22 for R and RA.

**Table 10.3.6:** Number of STAR classes by grade (Kindergarten‐3) and designation S (small), R (Regular) and RA (Regular with Aide)

**Class size**							
		11‐12	13‐17	18‐21	22‐25	26‐30	Total
**Kindergarten**	S	8	119	0	0	0	127
	R	0	8	28	61	5	102
	RA	0	2	26	61	10	99
**1. Grade**	S	2	112	10	0	0	124
	R	0	2	34	69	10	115
	RA	0	0	27	55	18	100
**2. Grade**	S	3	123	7	0	0	133
	R	0	1	12	74	13	100
	RA	0	0	15	82	10	107
**3. Grade**	S	4	121	15	0	0	140
	R	0	0	21	54	14	89
	RA	0	1	29	59	28	117

SOURCE: Achilles (1999) as reported in Finn et al. (2007)

### 10.4 STAR UNCORRECTED EFFECT SIZES

**Table:** 

	**Folger, 1989**	**Nye, 1992/994**	**Finn, 2001**	**Hanushek, 1999**
Read SMD [95% CI]				
Kindergarten	0.21 [0.15, 0.27]	0.18 [0.12, 0.24]	0.21 [0.15, 0.27]	0.17 [0.11, 0.23]
1. Grade	0.34 [0.28, 0.40]	0.24 [0.18, 0.30]	0.30 [0.24, 0.36]	0.23 [0.17, 0.29]
2. Grade	0.26 [0.20, 0.32]	0.23 [0.17, 0.29]	0.26 [0.20, 0.32]	0.20 [0.14, 0.26]
3. Grade	0.24 [0.18, 0.30]	0.26 [0.20, 0.32]	0.22 [0.16, 0.28]	0.22 [0.16, 0.28]
Mathematics SMD [95% CI]				
Kindergarten	0.17 [0.11, 0.23]	0.15 [0.09, 0.21]	0.19 [0.13, 0.25]	0.17 [0.11, 0.23]
1. Grade	0.33 [0.27, 0.39]	0.27 [0.21, 0.33]	0.31 [0.25, 0.37]	0.26 [0.20, 0.32]
2. Grade	0.23 [0.17, 0.29]	0.20 [0.14, 0.26]	0.25 [0.19, 0.31]	0.19 [0.13, 0.25]
3. Grade	0.21 [0.15, 0.27]	0.23 [0.17, 0.29]	0.15 [0.09, 0.21]	0.18 [0.12, 0.24]

*Technical report:*

*Word, E.R., Johnston, J., Bain, H.P., Fulton, B.D., Zaharias, J.B., Achilles, C.M., Lintz, M.N., Folger, J. & Breda, C. (1994). The state of Tennessee's Student/Teacher Achievement Ratio (STAR) Project: Technical report 1985–1990. Nashville: Tennessee State Department of Education, 1994.*

*Finn et al., 2007*

*Finn, J.D., Boyd‐Zaharias, J., Fish, R.M. & Gerber, S.B. (2007). Project STAR and Beyond: Database User's Guide. HEROS, Incorporated.*

## 11 Appendices

### 11.1 SEARCH DOCUMENTATION

Examples of search strings used to search different host services: EBSCO, ProQuest, ISI Web of Science.

**ERIC (EBSCO)**

Latest search 14/2/2017. Search string from 2017 update. Search is limited from 20150101‐20171231. Search performed in full text.

**Table:** 

**Search**	**Terms**	**Results**
S16	S13 AND S15 – limited to 20150101‐20171231	235
S15	S5 AND S14	30,732
S14	student* OR pupil*	745,639
S13	S10 AND S11 AND S12	250
S12	S3 OR S4	12,413
S11	S1 OR S2	805
S10	S5 OR S6 OR S7 OR S8 OR S9	46,459
S9	Intellect* N2 develop*	197
S8	DE “Intellectual Development”	56
S7	School* N1 (performan* OR achiev*)	492
S6	Academic* N2 (performance* or achiev* or abilit* or outcome*)	4,251
S5	learn* OR develop* OR perform* OR achiev* OR abilit* OR outcome* OR improve*	46,459
S4	DE “Middle Schools” OR DE “Elementary Schools” OR DE “Secondary Schools* OR DE “Junior High Schools”	4,407
S3	(primary N1 School*) OR (elementary N1 school*) OR (secondary N1 school*) OR (middle N1 school*) OR (junior N1 high*)	12,413
S2	DE “Class Size” OR DE “Classroom Environment” OR DE “Crowding” OR DE “Flexible Scheduling” OR DE “Small Classes” OR DE “Teacher Student Ratio”	753
S1	class N2 size*	193

**International Bibliography of the Social Sciences (ProQuest)**

Latest searched January 2015. Search limited to 1980‐2015. Search performed in full text.

**Table:** 

**Search**	**Terms**	**Results**
S1	(“class size”) OR class size* OR class near/2 size*	1690
S2	((“class size”) OR class size* OR class near/2 size*) OR (((“classroom environment”) OR classroom environment* OR classroom near/1 environment*) OR ((“crowding”) OR crowding*)) OR ((flexible NEAR/1 scheduling* OR (“flexible scheduling”)) OR (“small classes*” OR small NEAR/1 classes*))	3423
S3	((“primary schools”) OR primary school* OR primary NEAR/1 school*) OR (((“elementary school students” OR “elementary schools”) OR elementary school* OR elementary NEAR/1 school*) OR ((“secondary schools”) OR secondary school* OR secondary near/1 school*)) OR (((“middle schools”) OR middle school* OR middle near/1 school*) OR ((“junior high schools” OR “junior high school students”) OR junior high* OR junior near/1 high))	13820
S4	((“learning”) OR learn*) OR (((“development”) OR develop* OR child development*) OR ((“performance”) OR perform*)) OR (((“achievement”) OR achieve*) OR ((“intellectual ability” OR “ability”) OR intelle* near/2 abili*)) OR (((“outcomes”) OR outcome*) OR ((“improvement”) OR improve*))	639914
S5	(school NEAR/1 (performan* OR achiev*)) OR (((“intellectual development”) OR intellectual near/1 development*) OR (intelle* near/2 develop*))	2516
S6	((school NEAR/1 (performan* OR achiev*)) OR (((“intellectual development”) OR intellectual near/1 development*) OR (intelle* near/2 develop*))) OR (((“learning”) OR learn*) OR (((“development”) OR develop* OR child development*) OR ((“performance”) OR perform*)) OR (((“achievement”) OR achieve*) OR ((“intellectual ability” OR “ability”) OR intelle* near/2 abili*)) OR (((“outcomes”) OR outcome*) OR ((“improvement”) OR improve*)))	639924
S7	(((school NEAR/1 (performan* OR achiev*)) OR (((“intellectual development”) OR intellectual near/1 development*) OR (intelle* near/2 develop*))) OR (((“learning”) OR learn*) OR (((“development”) OR develop* OR child development*) OR ((“performance”) OR perform*)) OR (((“achievement”) OR achieve*) OR ((“intellectual ability” OR “ability”) OR intelle* near/2 abili*)) OR (((“outcomes”) OR outcome*) OR ((“improvement”) OR improve*)))) AND (((“primary schools”) OR primary school* OR primary NEAR/1 school*) OR (((“elementary school students” OR “elementary schools”) OR elementary school* OR elementary NEAR/1 school*) OR ((“secondary schools”) OR secondary school* OR secondary near/1 school*)) OR (((“middle schools”) OR middle school* OR middle near/1 school*) OR ((“junior high schools” OR “junior high school students”) OR junior high* OR junior near/1 high))) AND (((“class size”) OR class size* OR class NEAR/2 size*) OR (((“classroom environment”) OR classroom environment* OR classroom near/1 environment*) OR ((“crowding”) OR crowding*)) OR ((flexible NEAR/1 scheduling* OR (“flexible scheduling”)) OR (“small classes*” OR small NEAR/1 classes*)))	189

**Science Citation Index & Social Science Citation Index (ISI Web of Science)**

Latest search 14/2/2017. Search string from 2017 update. Search is limited from 20150101‐20171231.

**Table:** 

**Search**	**Results**	**Search Terms**
# 17	503	#16 OR #14
		*Indexes = SCI‐EXPANDED, SSCI Timespan = 2015‐2017*
# 16	8	#15 AND #13 AND #12
		*Indexes = SCI‐EXPANDED, SSCI Timespan = 2015‐2017*
# 15	29	(TI = (“class size*”))
		*Indexes = SCI‐EXPANDED, SSCI Timespan = 2015‐2017*
# 14	503	#13 AND #12 AND #11
		*Indexes = SCI‐EXPANDED, SSCI Timespan = 2015‐2017*
# 13	2,073,907	#9 OR #8 OR #7 OR #6 OR #5 OR #4
		*Indexes = SCI‐EXPANDED, SSCI Timespan = 2015‐2017*
# 12	75,716	#10 OR #3
		*Indexes = SCI‐EXPANDED, SSCI Timespan = 2015‐2017*
# 11	10,953	#2 OR #1
		*Indexes = SCI‐EXPANDED, SSCI Timespan = 2015‐2017*
# 10	67,485	(TS = (student* OR pupil*))
		*Indexes = SCI‐EXPANDED, SSCI Timespan = 2015‐2017*
# 9	826,667	(TS = (intellect* OR develop*))
		*Indexes = SCI‐EXPANDED, SSCI Timespan = 2015‐2017*
# 8	151	(TS = (“intellectual Development”))
		*Indexes = SCI‐EXPANDED, SSCI Timespan = 2015‐2017*
# 7	1,030,786	(TS = (school OR perform* OR achiev*))
		*Indexes = SCI‐EXPANDED, SSCI Timespan = 2015‐2017*
# 6	1,658,747	(TS = ((learn* OR develop* OR perfrom* OR achiev* OR abilit* OR outcome* OR improve*)))
		*Indexes = SCI‐EXPANDED, SSCI Timespan = 2015‐2017*
# 5	1,335,501	(TS = ((academic* OR performance* OR achiev* OR abilit* OR outcome* OR improve*)))
		*Indexes = SCI‐EXPANDED, SSCI Timespan = 2015‐2017*
# 4	1,863,667	(TS = ((learn* or develop* or perform* or achiev* or abilit* or outcome*)))
		*Indexes = SCI‐EXPANDED, SSCI Timespan = 2015‐2017*
# 3	17,143	(TS = ((primary school*) OR (elementary school*) OR (secondary school*) OR (middle school*) OR (junior high*) OR (“middle schools”) OR (“elementary schools”) OR (“secondary schools”) OR (“junior high schools”)))
		*Indexes = SCI‐EXPANDED, SSCI Timespan = 2015‐2017*
# 2	2,439	(TS = (“class size” OR“classroom environment” OR“crowding” OR“flexible scheduling” OR“small classes” OR“teacher student ratio”))
		*Indexes = SCI‐EXPANDED, SSCI Timespan = 2015‐2017*
# 1	8,715	(TS = (class size*))
		*Indexes = SCI‐EXPANDED, SSCI Timespan = 2015‐2017*

**Centre for Reviews and Dissemination Databases**

Latest searched January 2017. Search limited to 2015‐2017. Search performed in full text.

This search string was also utilised on Campbell Collaboration Library, EPPI‐Centre Systematic Reviews ‐ Database of Education, Social Care Online with minor modifications.

**Table:** 

**Search**	**Terms**	**Hits**
**1**	class size*	**0**
**2**	“Class Size” OR “Classroom Environment” OR DE “Crowding” OR “Flexible Scheduling” OR “Small Classes” OR “Teacher Student Ratio”	**3**
**3**	(Primary School*) or (Elementary school*) or (secondary school*) or (middle school*) or (Junior high) or (“Middle Schools”) OR (“Elementary Schools”) OR (“Secondary Schools”) OR (“Junior High Schools”)	**29**
**4**	learn* or develop* or perform* or achiev* or abilit* or outcome*	**39712**
**5**	learn* or develop* or perform* or achiev* or abilit* or outcome* or improve*	**41059**
**6**	Intellectual Development*	**7**
**7**	“Class Size” OR “Classroom Environment” OR DE “Crowding” OR “Flexible Scheduling” OR “Small Classes” OR “Teacher Student Ratio” AND (Primary School*) or (Elementary school*) or (secondary school*) or (middle school*) or (Junior high) or (“Middle Schools”) OR (“Elementary Schools”) OR (“Secondary Schools”) OR (“Junior High Schools”)	**3**
**8**	“Class Size” OR “Classroom Environment” OR DE “Crowding” OR “Flexible Scheduling” OR “Small Classes” OR “Teacher Student Ratio” AND Learn* or develop* or perform* or achiev* or abilit* or outcome* or improve* or Intellectual Development* AND Primary School* or Elementary school* or secondary school* or middle school* or Junior high or “Middle Schools” OR “Elementary Schools” OR “Secondary Schools” OR “Junior High Schools”	**3**

**Searches on National Library Portals**

Searches on these portals were performed in both English and Danish, Swedish and Norwegian. Searches where performed latest in 2015. Searches were limited to 1980‐2015.

**Bibliotek.dk**

**Table:** 

**Search**	**Terms**	**Hits**
1	EM: class size	10
2	TI: class size*	30
3	(TI: class size* OR EM: class size) – Limiters: 1980‐2015, bøger + artikler + tidsskrifter + e‐bøger, engelsk, dansk, norsk, svensk	40

**Table:** 

**Search**	**Terms**	**Hits**
1	EM: “klassestørrelse*” ‐ EMNE, Bøger, tidsskrifter, artikler, 1980‐, dansk, svensk, norsk, engelsk	36

**Libris**

**Table:** 

**Search**	**Term**	**Hits**
1	class size* ‐ Limiters: Keywords	175
2	class size* ‐ Limiters: Title	32
3	class size* ‐ Limiters: Subject	24
4	“class size” OR subject:(class size) OR title:(class size*)	145

**Table:** 

**Search**	**Terms**	**Hits**
1	klasstorlek* ‐ Fritekst	34
2	tit:klasstorlek* ‐ Titel	5
3	zamn:“^Klasstorlek^” ‐ Emne	15
4	S1 OR S2 OR S3	35

**BIBSYS**

**Table:** 

**Search**	**Terms**	**Hits**
**1**	Class Size* OR “Classroom Environment” OR “Crowding” OR “Flexible Scheduling” OR “Small Classes” OR “Teacher Student Ratio”	11.669
**2**	Class Size* OR “Classroom Environment” OR “Crowding” OR “Flexible Scheduling” OR “Small Classes” OR “Teacher Student Ratio” AND Primary School* OR Elementary school* OR secondary school* OR middle school* OR Junior high OR “Middle Schools”	228
**3**	((“Class Size*” OR “Classroom Environment” OR “Crowding” OR “Flexible Scheduling” OR “Small Classes” OR “Teacher Student Ratio”) AND (Primary School* OR Elementary school* OR secondary school* OR middle school* OR Junior high OR “Middle Schools”) AND (learn* OR develop* OR perform* OR achiev* OR abilit* OR outcome* OR improve*))	188

**Table:** 

**Search**	**Terms**	**Hits**
1	(“klassestørrelse*”)	21

**Grey literature sources**

Latest searches performed in 2017.

**Table:** 

**Web‐source**	**Search**	**Terms**	**Limiters**	**Hits**
*What Works Clearinghouse ‐ U.S. Department of Education*	1	class size*	Reviewed Studies	2
	2	small class*	Reviewed Studies	3
	3	classroom environment*	Reviewed Studies	12
*edu.au.dk ‐ clearinghouse*	1	class size*	Publikationer	85
*European Educational Research Association*	1	class size*		26
*American Educational Research Association (AERA)*	1	class size*		205
*Social Science Research Network (SSRN)*	1	class size*	Title, Abstract, Abstract ID & Keywords	762
	2	“class size”	Title	60
	3	“class size”	Title, Abstract, Abstract ID & Keywords	154

**Google Scholar**

**Table:** 

**Search Documentation Template**	**Insert terms/detalis below**
• Authors	
• Publication	
• Journal ISSN	
• All of the words	class size
• Any of the words	effect RCT random review intervention trial teach learn achievement student
• None of the words	
• The phrase	
• Year of publication between	2015‐2017
• Data Source	Google Scholar
• Title words only	X
• Results	55
• Search Date	02/02‐2017.

### 11.2 FLOW CHART FOR LITERATURE SEARCH

**Figure 11.1:**
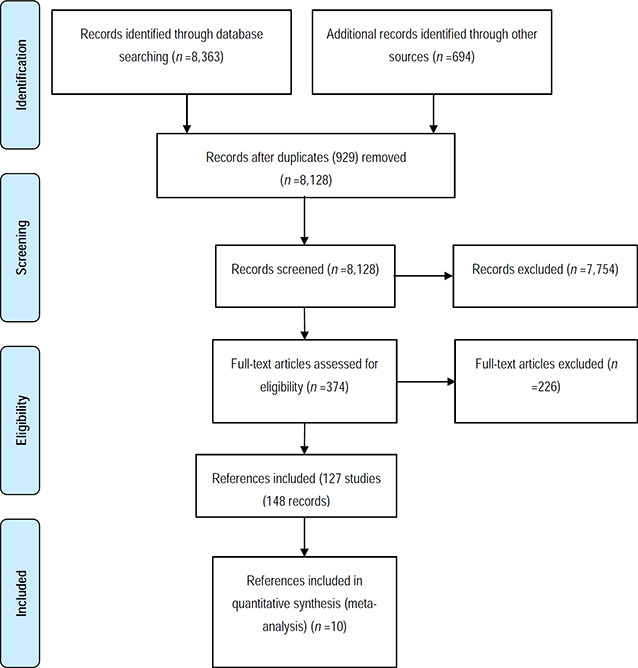


### 11.3 FIRST AND SECOND LEVEL SCREENING

First level screening is on the basis of titles and abstracts. Second level is on the basis of full text

Reference id. No.:
Study id. No.:
Reviewers initials:
Source:
Year of publication:
Duration of study:
Country/countries of origin
Author

The study will be excluded if one or more of the answers to question 1‐3 are ‘No’. If the answers to question 1 to 3 are ‘Yes’ or ‘Uncertain’, then the full text of the study will be retrieved for second level eligibility. All unanswered questions need to be posed again on the basis of the full text. If not enough information is available, or if the study is unclear, the author of the study will be contacted if possible.

**First level screening questions are based on titles and abstracts**

1. Does the study focus on class size?
  Yes ‐ include
  No – if no then stop here and exclude
  Uncertain ‐ include

Question 1 guidance:

The intervention in this review is a reduction in class size. Studies only considering student‐teacher ratio will not be eligible. Neither will studies where the intervention is the assignment of an extra teacher (or teaching assistants or other adults) to a class be eligible.

2. Are the participants children in grades kindergarten to 12 (or the equivalent in European countries) in general education?
  Yes ‐ include
  No – if no then stop here and exclude
  Uncertain ‐ include

Question 2 guidance:

Regular private, public or boarding schools are eligible. We exclude children in home‐school, in pre‐school programs, and in special education.

3. Is the report/article a quantitative evaluation study with a comparison condition?
  Yes ‐ include
  No – if no then stop here and exclude
  Uncertain ‐ include

Question 3 guidance:

We are only interested in primary quantitative studies with a comparison group, where the authors have analysed the data. We are not interested in theoretical papers on the topic or surveys/reviews of studies of the topic. (This question may be difficult to answer on the base of titles and abstracts alone.)

**Second level screening questions based on full text**

4. Are outcomes measured at the individual or class level ?
  Yes ‐ include
  No – if no then stop here and exclude
  Uncertain ‐ include

Question 4 guidance

Some use test score data on individual students and actual class‐size data for each student. Others use individual student data but average class‐size data for students in that grade in each school. Still others use average scores for students in a grade level within a school and average class size for students in that school. We will only include studies that use data on the individual or class level. We will exclude studies that rely on data aggregated to a level higher than the class.

### 11.4 CODING FORM

**Table:** 

**Names of author(s)**
**Title**
**Language**
**Journal**
**Year**
**Country**
**Participant characteristic (age, grade level, gender, socioeconomic status, ethnicity)**
**Duration of class size reduction (years)**
**Class size (divide into treated/comparison)**
**Type of data used in study (administrative, questionnaire, other (specify))**
**Level of aggregation (individual or class)**
**Time period covered by analysis (divide into intervention and follow up)**
**Sample size (divide into treated/comparison)**

**Outcome measures**

Instructions: Please enter outcome measures in the order in which they are described in the report. Note that a single outcome measure can be completed by multiple sources and at multiple points in time (data from specific sources and time‐points will be entered later).

**Table:** 

#	Outcome & measure	Reliability & Validity	Format	Direction	Pg# & notes
1		Info from: Other samples This sample Unclear	Dichotomy Continuous	High score or event is Positive Negative Can't tell	

* Repeat as needed

**OUTCOME DATA**

**DICHOTOMOUS OUTCOME DATA**

**Table:** 

OUTCOME	TIME POINT (s) (record exact time from participation, there may be more than one, record them all)	SOURCE	VALID Ns	CASES	NON‐CASES	STATISTICS	Pg. # & NOTES
		Questionnaire Admin data Other (specify) Unclear	Participation	Participation	Participation	RR (risk ratio) OR (odds ratio) SE (standard error) 95% CI DF P‐ value (enter exact p value if available) Chi2 Other	
			Comparison	Comparison	Comparison		

**Repeat as needed**

**CONTINUOUS OUTCOME DATA**

**Table:** 

OUTCOME	TIME POINT (s) (record exact time from participation, there may be more than one, record them all)	SOURCE (specify)	VALID Ns	Means	SDs	STATISTICS	Pg. # & NOTES
		Questionnaire Admin data Other (specify) Unclear	Participation	Participation	Participation	P t F Df ES Other	
			Comparison	Comparison	Comparison		

*Repeat as needed

### 11.5 ASSESSMENT OF RISK OF BIAS IN INCLUDED STUDIES

**Risk of bias table**

**Table:** 

**Item**	**Judgement** ^a^	**Description** (quote from paper, or describe key information)
1. Sequence generation		
2. Allocation concealment		
3. Confounding^b^, ^c^		
4. Blinding?^b^		
5. Incomplete outcome data addressed?^b^		
6. Free of selective reporting?^b^		
7. Free of other bias?		
*8. A priori* protocol?^d^		
*9. A priori* analysis plan?^e^		

a Some items on low/high risk/unclear scale (double‐line border), some on 5 point scale/unclear (single line border), some on yes/no/unclear scale (dashed border). For all items, record “unclear” if inadequate reporting prevents a judgement being made.

b For each outcome in the study.

c This item is only used for NRCTs and NRSs. It is based on list of confounders considered important at the outset and defined in the protocol for the review (*assessment against worksheet*).

d Did the researchers write a protocol defining the study population, intervention and comparator, primary and other outcomes, data collection methods, etc. in advance of starting the study?

e Did the researchers have an analysis plan defining the primary and other outcomes, statistical methods, subgroup analyses, etc. in advance of starting the study?

**Risk of bias tool**

**Studies for which RoB tool is intended**

The risk of bias model was developed by Prof. Barnaby Reeves in association with the Cochrane Non‐Randomised Studies Methods Group.^11^ This model, an extension of the Cochrane Collaboration's risk of bias tool, covers risk of bias in both randomised controlled trials (RCTs and QRCTs) and in non‐randomised studies (NRCTs and NRSs).

The point of departure for the risk of bias model is the Cochrane Handbook for Systematic Reviews of interventions (Higgins & Green, 2008). The existing Cochrane risk of bias tool needs elaboration when assessing non‐randomised studies because, for non‐randomised studies, particular attention should be paid to selection bias / risk of confounding. Additional item on confounding is used only for non‐randomised studies (NRCTs and NRSs) and is not used for randomised controlled trials (RCTs and QRCTs).

**Assessment of risk of bias**

Issues when using modified RoB tool to assess included non‐randomised studies:

- Use existing principle: score judgment and provide information (preferably direct quote) to support judgment
- Additional item on confounding used only for non‐randomised studies (NRCTs and NRSs).
- 5‐point scale for some items (distinguish “unclear” from intermediate risk of bias).
- Keep in mind the general philosophy – assessment is not about whether researchers could have done better but about risk of bias; the assessment tool must be used in a standard way whatever the difficulty / circumstances of investigating the research question of interest and whatever the study design used.
- Anchors: “1/No/low risk” of bias should correspond to a high quality RCT. “5/high risk” of bias should correspond to a risk of bias that means the findings should not be considered (too risky, too much bias, more likely to mislead than inform)

1. Sequence generation
  - Low/high/unclear RoB item
  - Always high RoB (not random) for a non‐randomised study
  - Might argue that this item redundant for NRS since always high – but important to include in RoB table (‘level playing field’ argument)
2. Allocation concealment
  - Low/high/unclear RoB item
  - Potentially low RoB for a non‐randomised study, e.g. quasi‐randomised (so high RoB to sequence generation) but concealed (reviewer judges that the people making decisions about including participants didn't know how allocation was being done, e.g. odd/even date of birth/hospital number)
3. RoB from confounding (additional item for NRCT and NRS; assess for each outcome)
  - Assumes a pre‐specified list of potential confounders defined in the protocol
  - Low(1) / 2 / 3 / 4 / high(5) / unclear RoB item
  - Judgment needs to factor in:
    ○ proportion of confounders (from pre‐specified list) that were considered
    ○ whether most important confounders (from pre‐specified list) were considered
    ○ resolution/precision with which confounders were measured
    ○ extent of imbalance between groups at baseline
    ○ care with which adjustment was done (typically a judgment about the statistical modeling carried out by authors)
  - Low RoB requires that all important confounders are balanced at baseline (not primarily/not only a statistical judgment OR measured ‘well’ and ‘carefully’ controlled for in the analysis.
  Assess against pre‐specified worksheet. Reviewers will make a RoB judgment about each factor first and then ‘eyeball’ these for the judgment RoB table.
4. RoB from lack of blinding (assess for each outcome, as per existing RoB tool)
  - Low(1) / 2 / 3 / 4 / high(5) / unclear RoB item
  - Judgment needs to factor in:
    ○ nature of outcome (subjective / objective; source of information)
    ○ who was / was not blinded and the risk that those who were not blinded could introduce performance or detection bias
    ○ see Ch.8
5. RoB from incomplete outcome data (assess for each outcome, as per existing RoB tool)
  - Low(1) / 2 / 3 / 4 / high(5) / unclear RoB item
  - Judgment needs to factor in:
    ○ reasons for missing data
    ○ whether amount of missing data balanced across groups, with similar reasons
    ○ whether censoring is less than or equal to 25% and taken into account
    ○ see Ch.8
6. RoB from selective reporting (assess for each outcome, NB different to existing Ch.8 recommendation)
  - Low(1) / 2 / 3 / 4 / high(5) /unclear RoB item
  - Judgment needs to factor in:
    ○ existing RoB guidance on selective outcome reporting (see Ch.8)
    ○ also, extent to which analyses (and potentially other choices) could have been manipulated to bias the findings reported, e.g. choice of method of model fitting, potential confounders considered / included
    ○ look for evidence that there was a protocol in advance of doing any analysis / obtaining the data (difficult unless explicitly reported); NRS very different from RCTs. RCTs must have a protocol in advance of starting to recruit (for REC/IRB/other regulatory approval); NRS need not (especially older studies)
    ○ Hence, separate yes/no items asking reviewers whether they think the researchers had a pre‐specified protocol and analysis plan.
7. RoB from other bias (assess for each outcome, NB different to existing Ch.8 recommendation)
  - Low(1) / 2 / 3 / 4 / high(5) /unclear RoB item
  - Judgment needs to factor in:
    ○ existing RoB guidance on other potential threats to validity (see Ch.8)
    ○ also, assess whether suitable cluster analysis is used (e.g. cluster summary statistics, robust standard errors, the use of the design effect to adjust standard errors, multilevel models and mixture models), if assignment of units to treatment is clustered

**Confounding worksheet**

**Table:** 

**Assessment of how researchers dealt with confounding**	** **	
Method for *identifying* relevant confounders described by researchers:	Yes	□
	no	□
If yes, describe the method used:		
Relevant confounders described:	yes	□
	no	□
List confounders described on next page		
Method used for controlling for confounding		
‘At design stage (e.g. matching, regression discontinuity, instrument variable):		
………………………………………		
………………………………………		
………………………………………		
At analysis stage (e.g. stratification, regression, difference‐indifference):		
………………………………………		
………………………………………		
………………………………………		
Describe confounders controlled for below		

**Confounders described by researchers**

Tick (yes[0]/no[1] judgment) if confounder considered by the researchers [Cons'd?]

Score (1[good precision] to 5[poor precision]) precision with which confounder measured

Score (1[balanced] to 5[major imbalance]) imbalance between groups

Score (1[very careful] to 5[not at all careful]) care with which adjustment for confounder was carried out

**Table:** 

**Confounder**	Considered	Precision	Imbalance	Adjustment
Gender	□	□	□	□
Age	□	□	□	□
Grade level	□	□	□	□
Socioeconomic status	□	□	□	□
Base line achievement	□	□	□	□
Local education spending	□	□	□	□
Unobservables^12^	□	Irrelevant	□	□
Other:	□	□	□	□

**User guide for unobservables**

Selection bias is understood as systematic baseline differences between groups and can therefore compromise comparability between groups. Baseline differences can be observable (e.g. age and gender) and unobservable (to the researcher; e.g. motivation and ‘ability’). There is no single non‐randomised study design that always solves the selection problem. Different designs solve the selection problem under different assumptions and require different types of data. Especially how different designs deal with selection on unobservables varies. The “right” method depends on the model generating participation, i.e. assumptions about the nature of the process by which participants are selected into a programme.

As there is no universal correct way to construct counterfactuals we will assess the extent to which the identifying assumptions (the assumption that makes it possible to identify the counterfactual) are explained and discussed (preferably the authors should make an effort to justify their choice of method). We will look for evidence that authors using e.g. (this is NOT an exhaustive list):

**Natural experiments:**

Discuss whether they face a truly random allocation of participants and that there is no change of behavior in anticipation of e.g. policy rules.

**Instrument variable (IV):**

Explain and discuss the assumption that the instrument variable does not affect outcomes other than through their effect on participation.

**Matching (including propensity scores):**

Explain and discuss the assumption that there is no selection on unobservables, only selection on observables.

**(Multivariate, multiple) Regression:**

Explain and discuss the assumption that there is no selection on unobservables, only selection on observables. Further discuss the extent to which they compare comparable people.

**Regression Discontinuity (RD):**

Explain and discuss the assumption that there is a (strict!) RD treatment rule. It must not be changeable by the agent in an effort to obtain or avoid treatment. Continuity in the expected impact at the discontinuity is required.

**Difference‐in‐difference (Treatment‐control‐before‐after):**

Explain and discuss the assumption that outcomes of participants and nonparticipants evolve over time in the same way.

## 12 Data and analyses

**Sensitivity analysis: Reading**

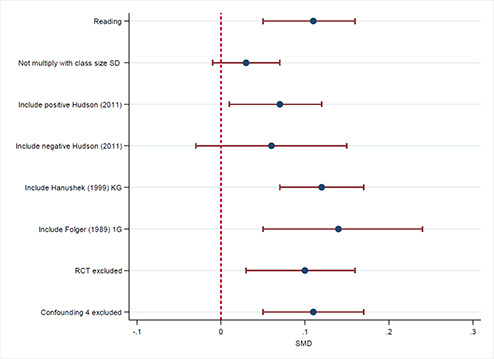

Sensitivity analysis: Mathematics

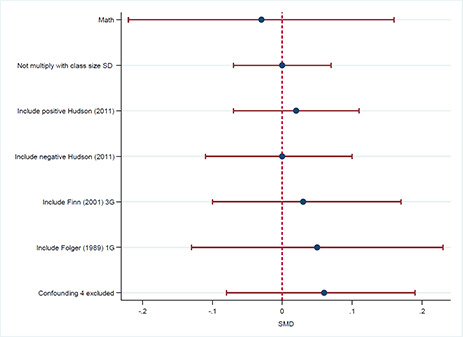

## Supporting information

Supplementary material

## Footnotes

1 The inverse chi‐square (Fisher) method (Hedges & Olkin, 1985)

2 The standardized regression coefficient measures the number of standard deviations of change in output that would be associated with a one standard deviation change in input.

3 The vote counting method did not necessarily lead to a different conclusion. It depends upon the inference procedure associated with the method (i.e., the category with the most “votes” represents the true state). The analysis in Hanushek (1989) shows that 24 (3) percent of the coefficients on expenditure were positive (negative) and significant and 46 (24) percent were positive (negative) and non‐significant, implying that the typical relation is positive. Note that none of the methods used in either Hanushek (1989) or Hedges et al. (1994) combines magnitude of effect size and significance.

4 This risk of bias model was introduced by Prof. Reeves at a workshop on risk of bias in non‐randomised studies at SFI Campbell, February 2011. The model is a further development of work carried out in the Cochrane Non‐Randomised Studies Method Group (NRSMG).

5 Student achievement data continued to be collected for students who had been in the STAR experiment (in grades K‐3) through grade 4 to 8.

6 Word et al. (1994) is the Technical report of the STAR experiment and Word et al. (1990) is the final summary report of the STAR experiment.

7 Avoiding overlap of data samples, 51 additional studies could have been used in the data synthesis.

8 Members of the review team at SFI Campbell were: the research assistants Julie Kaas Seerup, Julie Marie Nielsen, Louise Jean Kristiansen and Anne‐Marie Klint Jørgensen.

9 Which, as mentioned in section 1.4, did not have to be the one provided by Hanushek, it could have been more positive towards small classes.

10 The description of IV and their interpretation is based on Angrist & Pischke (2009) and Heckman & Urzúa (2010).

11 This risk of bias model was introduced by Prof. Reeves at a workshop on risk of bias in non‐randomised studies at SFI Campbell, February 2011. The model is a further development of work carried out in the Cochrane Non‐Randomised Studies Method Group (NRSMG).

12 See user guide for unobservables
