# Supplementary material for: Small class sizes for improving student achievement in primary and secondary schools: a systematic review
Source: Campbell Syst Rev. 2018 Oct 11;14(1):1–107. doi: 10.4073/csr.2018.10 (PMC8428040; doi:10.4073/csr.2018.10)
Supplement: Supplementary file 1 — Supplementary material [file CL2-14--s001.docx]

# Online supplements

Small Class Sizes for Improving Student Achievement in Primary and Secondary Schools: a systematic review

Trine Filges, Christoffer Scavenius Sonne-Schmidt, Anne-Marie Klint Jørgensen

# Table of contents

1 Characteristics of studies 3

1.1 Characteristics of included studies 3

1.2 Characteristics of excluded studies 47

2 Data appendices 49

2.1 Data extraction 49

2.2 Risk of bias 83

#

# Characteristics of studies

## Characteristics of included studies

| Study | Achilles, 1993a | Achilles, 1993b | Achilles, 1995 |
| --- | --- | --- | --- |
| **Participant characteristics (age, gender, grade level, socio economic status, ethnicity)** | Grade KG-3. Data from Project STAR. Participant characteristics not reported. | Data from Project STAR (grade K-3). Data from Lasting Benefits Study (LBS) (grade 4 & 5). A listing of specific data instruments is in Appendix A. | Students in 1. Grade through 3. grade, gender unspecified, ethnicity specified (white, minority - percentage of white: control 88,7 % - reduced 87,6 %), SES specified (percept of Chapter 1 (Ch.1), free/reduced lunch (FL), parents with less than high school education (<HS) - control: Ch.1 24,9 %, FL 36,8 %, <HS 25,9 % - reduced: Ch.1 16,7 %, FL 40,8 %, <HS 29,6 %) |
| **Type of school (private, boarding, public)** | Public School | Public School | Not reported |
| **Duration of class size reduction** | 4 years (K-3, data from Project STAR) | 4 years (K-3, data from Project STAR) | 1 year (from 1991-1992) |
| **Class size (divide into treated/comparison)** | Small classes: 13-17 (p. 7), Regular: 22-25 (p. 7), Regular-with-aide: 18-21 (p. 7). Some classes were 'out of range' (table 2, p. 8 and 16) | Small classes: 13-17. Regular: 22-26. Regular-with-aide: 22-26 | Small classes: average 15 students and regular classes: average 25 students |
| **Intensity (size of reduction)** | Not specified. | Not specified. | 10 |
| **Precision of class size measure (is it constant per subject/during the day)** | Constant | Constant. | Not reported |
| **Time period covered by analysis (divide into treatment/follow up)** | Project STAR: 1985-1989 | Project STAR: 1985-1989. LBS: 1990-1992 | 3 years: 1991-1994 (treatment in 1991-1992, follow up in 1993-1994) |

| Study | Akerhielm, 1995 | Angrist, 1999 | Angrist, 2014 |
| --- | --- | --- | --- |
| **Participant characteristics (age, gender, grade level, socio economic status, ethnicity)** | Grade: 8. Gender, ethnicity, SES: Not reported. | Grade: 3, 4 & 5. Gender: Not reported. SES: Not reported. Ethnicity: Not reported. | Italian Elementary schools. Grade 2 and grade 5. Fewer than 10 pct. of Italian primary and secondary students who attend private schools are omitted from the study. Student data include gender, citizenship and information on parents’ employment status and educational background (p. 7) "the empirical analysis is restricted to classes with more than the minimum number of students set by law (10 before 2010 and 15 before 2011). This selection rule eliminates classes in the least populated areas of the country, mostly mountainous areas and small islands. We also drop schools enrolling more than 160 students in a grade." |
| **Type of school (private, boarding, public)** | Public school | Public school | Unclear (both private and public) |
| **Duration of class size reduction** | Not reported | Not reported | Not reported |
| **Class size (divide into treated/comparison)** | Small: 0-20; medium: 16-30; large: 31+ | Class sizes varies from <22 to >37. | Focusses on class-level averages, p.7. Average class size about 20 in each grade, but slightly lower in the south. |
| **Intensity (size of reduction)** | Unclear | Varies | Not specified |
| **Precision of class size measure (is it constant per subject/during the day)** | Unclear; Class size varies pr. subject from 24-26 | Not reported | At the beginning of each school year. P.7 |
| **Time period covered by analysis (divide into treatment/follow up)** | 1 year (1988) | Test score results from June 1991 and June 1992. | 2009-2011 |

| Study | Annevelink, 2004 | Balestra, 2014 | Bingham, 1994 |
| --- | --- | --- | --- |
| **Participant characteristics (age, gender, grade level, socio economic status, ethnicity)** | Grade: 1. Gender: Not reported. Ethnicity: specified through language spoken at home (Dutch, non-Dutch, Friesian/dialect) No data on distribution. SES: specified through pupil weight. No data on distribution. | Grade: K-3. Gender, ethnicity, SES: Not reported. | Data from Project STAR (grade K-3). Focus on differences in achievement between white and minority students (researcher creates subsamples of white and minority students). Participant characteristics are not reported. Number of observations reported in appendix A, p. 45 |
| **Type of school (private, boarding, public)** | Not reported | Public school | Public School |
| **Duration of class size reduction** | 4 years (step 1: 1997, step 2, 3 and 4: 2000, 2001, 2002) (only step 1 relevant for study) | 4 years (Project STAR) | 4 years (K-3, data from Project STAR) |
| **Class size (divide into treated/comparison)** | Small class: average 15 students and regular classes: average 24 students | Small classes: 13-17. Regular classes: 22-26; Regular classes w. a teacher aide | Class sizes not reported in this article. Class size divided into small, regular and regular-with-aide. (p. 5-6) |
| **Intensity (size of reduction)** | One teacher to every 20 students (2002-2003) | 13-17 vs. 22-26 (13) | Not specified. |
| **Precision of class size measure (is it constant per subject/during the day)** | Not reported | Not reported | Constant |
| **Time period covered by analysis (divide into treatment/follow up)** | 3 years: 1997-2000 (treatment in 1997, follow up in 1999-2000) | Treatment: 4 years (1985-1989). Follow-up: 1 year (1998) | Project STAR: 1985-1989 |

| Study | Blatchford, 2002 | Blatchford, 2003a | Blatchford, 2003b |
| --- | --- | --- | --- |
| **Participant characteristics (age, gender, grade level, socio economic status, ethnicity)** | Age: 4-7 years old. Gender: Not reported. Ethnicity: Not reported. SES specified through free-lunch eligibility (no data on distribution). | Age: 5-7 years old. Gender: Not reported. Ethnicity: Not reported. SES: mentioned (no data on distribution and estimate unspecified). | P. 21: The schools are randomly selected (but were not compelled to take part), both situated in urban, suburban and rural areas (claims to be fairly representative), and contained pupils from families with low income, measured by free school meals, (17 pct., which was about the same as in England generally). At the start the study contained an equal number of boys and girls. 97 pct. Of the pupils had English as first language, 91 per cent were classified as being from white UK ethnic backgrounds. The children were followed from entry to reception year and then three years, and were aged 4-6/5-7 years. It then also states that students were either all through primary school: aged 4-11 years (74 pct.) or in infant school: aged 4-7 years (26 pct.). |
| **Type of school (private, boarding, public)** | Not reported | Not reported | Unclear (public) |
| **Duration of class size reduction** | Unclear | Not reported. | 3 years, p. 21 |
| **Class size (divide into treated/comparison)** | 30 students in average, no reference group | Large classes: 30 or above 30 students, large medium classes: 26-29 students, small medium classes: 20-25 students and small classes: below 20 students | This book focuses on a case study: Schools were selected for a case study within differing class size categories: large (30 and over), large medium (26–29), small medium (20–25) and small (under 20). Two classes in each class size band in each year were selected (eight classes were followed for three years, and visited each term, for years 1 and 2 there were three visits in the spring term) |
| **Intensity (size of reduction)** | Unspecified | 10 | Uses excising classes |
| **Precision of class size measure (is it constant per subject/during the day)** | Suggestions on math class reduction to 15-33 students and reading to 10-30 students (however, precision on measure is unspecified) | Not reported | Class size was measured "as on the school register, and also in terms of the number of children in the class at a given point in the term - called 'experienced' class size". (p. 23) |
| **Time period covered by analysis (divide into treatment/follow up)** | 2 years: 1996-1998 (data from 1996-1997 and 1997-1998, no follow up) | 2 years: 1996-1998 (data from 1996-1997 and 1997-1998, no follow up) | Enrolment /reception class (age 4-5 years): 1996/97. Year 1 (age 5-6 years): 1997/98. Year 2 (age 6-7 years): 1998/99. (p. 21). The children were given tests at the end of reception year, year one and year two. |

| Study | Bonesrønning, 2003 | Boozer, 1995 | Boozer, 2001a |
| --- | --- | --- | --- |
| **Participant characteristics (age, gender, grade level, socio economic status, ethnicity)** | From each region of Norway the county that revealed the largest variation in expenditure pr. student was chosen. Lower secondary school (8th-10th grade) - Students between 13 and 16 years old. Tests are performed for grade 9 and 10. 79 % of students are from intact families. 38 % of fathers (mothers) are classified as highly educated (p. 954-55). Approximately 7% of students receive special education services (p.956). Gender/age/ethnicity not reported. | Pupils: 8-12th grade (p. 4-5). Ethnicity (overall in sample): Blacks 14,1 %, Hispanics 11,2 %, White 70,5 %, Other 5,9 % (p. 11). Data from 'National Education Longitudinal Survey' (NELS). Data from ' The New Jersey Survey of Teachers': Conducted June 1994. Merged with data from 'Common Core of Data Surveys' (racial composition data) and administrative records from the New Jersey Department of Education (teacher data). (4) | Grade: 8 and 10. Gender: Not reported. Ethnicity: Not reported. SES specified through family income, parent's marital status (no data on distribution) |
| **Type of school (private, boarding, public)** | Unclear. | Public school | Public schools |
| **Duration of class size reduction** | Not reported. Variation in class size is generated from a maximum class size rule (max 30 students). | Cohort followed for 5 years (8th-12th grade): Survey data from 1988 through 1992 is used in the analysis (4) | Not reported. |
| **Class size (divide into treated/comparison)** | Class size varies between 9 and 51 students. Average size is 25.1 students (SD: 6.37). (p. 955) | Comparison of existing class sizes. Overall averages: Regular class average: 22.1; Special needs class average: 9.7; Remedial class average: 10; Gifted class average: 22.2; Other class average: 18.6. (11) | Regular class size mean 22 students, no reference group |
| **Intensity (size of reduction)** | Not applicable. Comparison of existing class sizes | Not applicable. Comparison of existing class sizes. | unspecified |
| **Precision of class size measure (is it constant per subject/during the day)** | Constant. Home-class size measure. (p. 954) | Variation in class size across academic subjects: math, science, reading, history (4) | Not reported |
| **Time period covered by analysis (divide into treatment/follow up)** | 1998 (tests at both the beginning and end of the school year). Class size data are reported from spring 1999. (p. 955) | 8th grade, 1988 (base sample). 10th grade, 1990 (follow up). 12th grade, 1992 (follow up). (p. 4) | 4 years: 1988-1992 (treatment in 1988, follow up in 1990 and 1992) |

| Study | Boozer, 2001b | Borland, 2005 | Bosworth, 2014 |
| --- | --- | --- | --- |
| **Participant characteristics (age, gender, grade level, socio economic status, ethnicity)** | Data from the Christchurch Health and Development Study. Summarizes highlights of six earlier reports. Ages 8 to 13, follow up at age 21 (measure completed education and unemployment). The CHDS data look strikingly similar to the Project STAR data. (p. 19) 59 children classified as Maori and 510 classified as Non-Maori. (p. 26) | Data from Kentucky Department of Education. Third grade students. Gender, race, age and innate ability collected and controlled for but not reported. (p. 74) | Grade: 4 and 5. Gender: 49 % female. Ethnicity: 59 % white, 29 % black, 6 % Hispanic and 6 % other. SES: specified through free lunch program, and parent education (=1 if parent has more than high school education) (free/reduced lunch 44 %, parent high education 0,45) |
| **Type of school (private, boarding, public)** | Both public and private schools (p. 28) | Not specified. | Not reported |
| **Duration of class size reduction** | 6 years | Unclear | Not reported. |
| **Class size (divide into treated/comparison)** | Classes separated into permanent categories of large, medium and small classes. Students who switch at least once between class size categories during the time frame of the sample are used as the comparison group. (p. 19) Average class size of small classes: 21.2, medium classes: 29.7, large classes: 33.2. Average drop in average class size from large to medium: 3.7 students, from medium to small: 8.5 students. (p. 21) | Average class sizes (appendix 2, p. 82): Mathematics: 20.98172 (SD: 3.169054). Spelling: 20.98192 (SD: 3.168612). Reading: 20.9822 (SD: 3.168482). Language: 20.98252 (SD: 3.168732). Science: 20.97134 (SD: 3.176206). Frequency distribution of class size found in appendix 3, p. 83 | Class size mean 23,02 students, range 15-30 |
| **Intensity (size of reduction)** | No class size reduction | Not applicable. Comparison of existing class sizes | Not specified |
| **Precision of class size measure (is it constant per subject/during the day)** | Measured annually. Constant for treated group: required that child stay in the same class type for all 6 years of the sample. (p. 19) | Class size measured annually per subject: Mathematics, Spelling, Reading, Language, Science (p. 82) | Not reported |
| **Time period covered by analysis (divide into treatment/follow up)** | 6 years. Follow up in 1998 (age 21). Treatment must then cover the time period 1984-90. | School year: 1989-1990 (p. 74) | 1 year: 2001-2002 (treatment in 2001-2002, no follow up) |

| Study | Bressoux, 2009 | Breton, 2012 | Burde, 1990 |
| --- | --- | --- | --- |
| **Participant characteristics (age, gender, grade level, socio economic status, ethnicity)** | Grade: 3. Gender: Not reported. Ethnicity: French vs. not-French (no data on distribution). SES specified through parents occupation, number of siblings (no data on distribution). | Data from TIMMS 2007. Fourth grade students in mathematics classes (p. 53). Participant characteristics (table 1, p. 53): Female: 0.50. Share of wealthy students in school (0-10% to >50%): 1.60. Computer at home (SES measure): 0.46. >10 books at home (SES measure): 0.66. Rural schools: 0.12. Ethnicity not reported | Population of sample 1: 400 'randomly selected' (identity not provided p. 55) fourth grade students in Michigan in the school year 1988-1889, who completed the MEAP reading and mathematics test (Michigan Educational Testing Program). Gender: 200 students were male, 200 students were female (p. 37). Sample 2 & 3 were derived from sample 1 (p.55). SES and ethnicity not reported. |
| **Type of school (private, boarding, public)** | Not reported | Both public and private school | Not reported. |
| **Duration of class size reduction** | Not reported. | Not reported. | Not reported. |
| **Class size (divide into treated/comparison)** | Class size mean 23.9 students overall; for untrained teachers 22.9. no reference group | Reported class size (incorrect): 44.1 (range: 6-80). Class size when tested (students present of the day of the test): 35.6 (range: 3-50). Reported class size (revised): 36.7 (range: 6-60). Number of students taking the test is used as the primary estimate of class size in the study. (Table 1, p. 53) | Sample 1 (p. 56): Mean of class size for males and females: 25.34 (SD: 5.04). Mean of class size for males only: 25.60 (SD: 4.98). Mean of class size for females only: 25.20 (SD: 5.10). Sample 2 (p. 57): Mean of class size for males and females: 25.27 (SD: 5.40). Mean of class size for males only: 26.45 (SD: 4.91). Mean of class size for females only: 24.61 (SD: 6.33). Sample 3 (p. 58): Class size 16-20 students: Mean is 19.38 (SD: 1.39) students. Class size 30-34 students: Mean is 31.82 (SD 1.21) students. |
| **Intensity (size of reduction)** | Unspecified | Not applicable. Comparison of existing class sizes | Not applicable. Comparison of existing class sizes |
| **Precision of class size measure (is it constant per subject/during the day)** | Not reported | Unclear. | Unclear |
| **Time period covered by analysis (divide into treatment/follow up)** | 1 year: 1991-1992 (treatment in 1991-1992, no follow up) | 2007. | Analysis on fall 1988 MEAP test (p.54). School year: 1988-1989 (p. 37) |

| Study | Carpenter, 2003 | Chargois, 2008 | Chetty, 2011 |
| --- | --- | --- | --- |
| **Participant characteristics (age, gender, grade level, socio economic status, ethnicity)** | Cohort (reception class) recruited in 1996. Pupils aged 4-7 years (reception year, year 1 and year 2). Participant characteristics collected but not reported. Results are only available on unfamiliar normalized scale. (p. 432) | Grade 5 students from 19 elementary campuses from one school district in southeast Texas. Community type: other central city (p. 79). Sixteen schools were Title 1 schools, where at least 40% of its student’s body on free-reduced price lunch (p. 81). Ethnicity: 62 % African American, 17 % Hispanics, 17 % White, Asian and Native American percentages below three per cent. Gender: 51 % female. School size: 23 % attended small schools (0-399), 40% attended medium schools (400-699), 35 % attended large schools (700+). (p. 85) | Data from STAR experiment. All schools were located in Tennessee, and the grades examined were Kindergarten to 3 grade. The children were 5-8 years old (p. 1598). The socioeconomic status of the sample was lower than the state of Tennessee and the U.S as a whole. |
| **Type of school (private, boarding, public)** | Not reported | Public School | Public |
| **Duration of class size reduction** | 3 years (reception class, year 1, year 2) (432) | Not reported. . | 4 years |
| **Class size (divide into treated/comparison)** | Descriptive information about class sizes are only reported in standard errors, where class size is centred at 30. (p. 440) | Class size divided into: 12-15, 16-19 and 20-21 in analysis (p. 103). | Large classes: 20-25 students and Small classes: 13-17 students. |
| **Intensity (size of reduction)** | Comparison of existing class sizes | Not applicable. Comparison of existing class sizes | From 22 on average to 15 on average |
| **Precision of class size measure (is it constant per subject/during the day)** | Unclear how class size is measured | Class sizes in math and reading are measured (p. 103), though class size is constant. | Constant |
| **Time period covered by analysis (divide into treatment/follow up)** | 1996-1999 | Students tested during 2007 (p. 77) | Children are followed from 1985 to 1989 (the STAR experiment). Then 95% of the participants are followed into adulthood, analysing their college attendance, earnings, and retirement savings and so on (about 20 years later, p. 1597). |

| Study | Clanet, 2010 | Costello, 1992 | Dee, 2011 |
| --- | --- | --- | --- |
| **Participant characteristics (age, gender, grade level, socio economic status, ethnicity)** | First year classes. Data is collected for a study by the French Ministry of Education. It states that psychosocial and demographic data were collected, but is not mentioned more specifically (p. 196) | Treatment and comparison together: Age: Not Reported. Gender: F: 56.8%. Grade: 1. SES: Unclear. Ethnicity: 73,9%, African-American and 26,1% Hispanic | NELS:88 data: National representative sample of 8th grade students (27) |
| **Type of school (private, boarding, public)** | Public school | Public school | Public school  (27) |
| **Duration of class size reduction** | Cohort followed for 1 year (196) | Test score results from the 1995 school year. It is unclear if that is the time period for the class size reduction as well. | Not specified |
| **Class size (divide into treated/comparison)** | Treatment: approximately 10 students per class. Control: 20-25 students per class (p. 196 + abstract) | Treatment: Small classes: 17; Comparison: large classes: 27 | Average class size in sample 24.5 (std. 5.9). (29) |
| **Intensity (size of reduction)** | 10-15 student reduction (abstract). | 17 vs. 27 (10) | Not applicable. Comparison of existing class sizes |
| **Precision of class size measure (is it constant per subject/during the day)** | Constant | Not reported | Variation in class size across academic subjects: math, science, English, history (29) |
| **Time period covered by analysis (divide into treatment/follow up)** | 2001-2002 | 1 year (1995) | Cohort followed for 13 years: 1988 - base year sample, 1990 - 1st follow up, 2000 - 4th follow up |

| Study | Dennis, 1986 | Dharmadasa, 1995 | Dieterle, 2013 |
| --- | --- | --- | --- |
| **Participant characteristics (age, gender, grade level, socio economic status, ethnicity)** | Second grade students from Metropolitan Nashville Public Schools, selected by the researcher (p. 82). The population was divided into an experimental school group, a control school group and a blind school group. The experimental school had approximately 50 pct. economically disadvantaged students. Community contains mostly single family residences; racial composition was 20 pct. black & 80 pct. white. The control group was drawn from a neighbourhood parallel to the experimental group. There were also a blind control group which was matched according to sex, race, economic status, birthdate within forty five days and pre reading (CAT 10) and reading (CAT 11) scores within four points. Free lunch status is compared between groups. | Fourth grade students (N=610), from 18 classrooms in the district of Kandy, Sri Lanka (p. 6). Participant characteristics not reported. | The class size reduction program was state wide. P.6. Grades were from kindergarten through G12. P.7. Data for teachers is available from first through sixth grade p.8. Teacher quality is researched based on data from grades: 4-6 p. 2. |
| **Type of school (private, boarding, public)** | Public School | Three types of schools (whether only public or not is unclear): Primary (grade 1-5), Kanista (grade 1-10), Maha Vidyala (grade 1-13) (p. 6) | Public school, p.8 |
| **Duration of class size reduction** | 2 years (p. 97) | Not reported. . Classes were selected primarily according to the three categories of class size. (p. 6) | Unclear - until class-size maximums were achieved |
| **Class size (divide into treated/comparison)** | Standard class size compared to classes with 15 students. (p.82) Control group consisted of classes with 25 students. | Three categories of class size: Small: 20-30 students, Medium: 31-40 students, Large: 41-50 students. (p.6-7) | P. 6-7: Separate class-size maximums were set for different grade levels: KG-G3: max 18 students. G4-G8: max 22 students. G9-G12: max 25 students. "The law allowed for a gradual phase-in of the mandated class sizes.” Average CS year 1: KG-G3: 23. G4-G8: 24. G9-G12: 24. Average CS year 8: KG-G3: 16. G4-G8: 19. G9-G12: 22. (Table 1 p. 7). There is differed between district CSR in 2003 and school CSR in 2006: the first three years the compliance of the law was based on a district average and for the next three years the compliance was based on a school-level average. p.7 |
| **Intensity (size of reduction)** | 10 | Not applicable. Comparison of existing class sizes | Unclear |
| **Precision of class size measure (is it constant per subject/during the day)** | Constant | Unclear. | Measured every year. |
| **Time period covered by analysis (divide into treatment/follow up)** | School year 1985/86. But is a continuation of research from a longitudinal study started in the school year 1984/85.( p. 2/3) 1984-1986 (p. 96-97) | 12 weeks (stated in abstract). | Data is available and compared from 2001-2002 school years until 2007-2008 school year. 7 years period. P. 7. Beginning of CSR program: 2003-2004 school years, p.6. Class-size averages are available from the beginning of the CSR program. P.8 |

.

| Study | Ding, 2005 | Ding, 2010 | Ding, 2011 |
| --- | --- | --- | --- |
| **Participant characteristics (age, gender, grade level, socio economic status, ethnicity)** | Data from Project STAR. Participant characteristics for sample: Free lunch (SES): 0,484, Ethnicity - White: 0,669; African American: 0,326, Gender - Female: 0,486, Inner city: 0,226, suburban: 0,223, rural: 0,461, urban: 0.09. (Table 1, appendix) | Data from STAR experiment, Tennessee. Children were from Kindergarten through first grade. Teachers were randomly assigned. Student characteristics are mentioned and calculated on but not stated (p.13-14), e.g. Free lunch status, gender, and race. | Data from Project STAR. Participant characteristics for sample: Free lunch (SES): 0,484, Ethnicity - White: 0,669; African American: 0,326, Gender - Female: 0,486, Inner city: 0,226, suburban: 0,223, rural: 0,461, urban: 0,09 (Table 1, appendix) |
| **Type of school (private, boarding, public)** | Public School | Unclear | Public School |
| **Duration of class size reduction** | Analysis focuses only on first year (Kindergarten) (p. 5). Overall duration of experiment: 4 years (K-3, data from Project STAR) | 4 years | Analysis focuses only on first year (Kindergarten) (p. 5). Overall duration of experiment: 4 years (K-3, data from Project STAR) |
| **Class size (divide into treated/comparison)** | Small classes: 13-17, Regular: 22-26, Regular-with-aide: 22-26. (p. 5). Mean class size in sample is 20,338 (SD: 3,981) (Table 1) | Small class: 13 to 17 students per teacher. Regular class: 22 to 25 students per teacher. Regular-with-aide class: 22 to 25 students with a full time teacher’s aide. Small classes are used for treatment and regular or regular-with-aide classes are used as control group. | Small classes: 13-17, Regular: 22-26, Regular-with-aide: 22-26. (p. 5). Mean class size in sample is 20,338 (SD: 3,981) (Table 1) |
| **Intensity (size of reduction)** | Not specified | 5-12 | Not specified |
| **Precision of class size measure (is it constant per subject/during the day)** | Constant | Constant | Constant |
| **Time period covered by analysis (divide into treatment/follow up)** | Data analysis on first year of experiment (p.5). Project STAR 1985-89 | 1985. 4 years. | Data analysis on first year of experiment (p.5). Project STAR 1985-89 |

| Study | Dobbelsteen, 2002 | Doulgas, 1989 | Ecalle, 2006 |
| --- | --- | --- | --- |
| **Participant characteristics (age, gender, grade level, socio economic status, ethnicity)** | Gender and weight factors accounting for SES: The weight factor ranges from 1 to 1.9: *1.9-pupils* are pupils with foreign-born parents with no or low income. *1.7-pupils* are pupils whose parents are transients. *1.4-pupils* live in a boarding school or a foster home. *1.25-pupils* are pupils of whom one parent has at most an education at VBO-level of education. All other pupils have a weight equal to one; Grade: 4, 6 and 8. Ethnicity: Unclear. | Re-analysis of project STAR data. Grade: K-3. | Grade: 1. Gender: Not reported. Ethnicity: French as first language vs. not as first language. SES: From high to low, 1-4. Data on distribution unclear, percentage unspecified. |
| **Type of school (private, boarding, public)** | Unclear | Public school | Not reported |
| **Duration of class size reduction** | Unclear | 4 years (STAR) | 1 year (from 2002-2003) |
| **Class size (divide into treated/comparison)** | Unclear | Small classes: 13-17 students, Regular classes: 22-25 students, Regular classes with full-time teacher's aide: 22-25 students | Small classes: 10-12 students and Regular classes: 20-25 students |
| **Intensity (size of reduction)** | Unclear | Not specified | 8-15 |
| **Precision of class size measure (is it constant per subject/during the day)** | Not reported | Constant | Not reported |
| **Time period covered by analysis (divide into treatment/follow up)** | 1994/1995 | 1985-1989 (STAR) | 1 year: 2002-2003 (treatment in 2002-2003, no follow up) |

| Study | Finn, 1989 | Finn, 1990a | Finn, 1990b |
| --- | --- | --- | --- |
| **Participant characteristics (age, gender, grade level, socio economic status, ethnicity)** | Data from project STAR (grade: K-3). Follow up in grade 4. Location: inner city, suburban, urban and rural. 23.7% minority students. 39.9% of students receiving free lunch. | Grade: 1. Gender: Not reported. Ethnicity specified as white vs. minorities: percentage of minorities: inner-city 96.2 % - urban 21.1 % - suburban 39.4 % - rural 7.2 %. SES: percentage receiving free lunch: inner-city 91.2 % - urban 46.6 % - suburban 33.2 % - rural 42.8 %. | Grade: K-2. Gender: Not reported. Ethnicity: control: majorities - percentage of minorities: inner-city 99.5 % - urban 11.8 % - suburban 31.5 % - rural 5.9 %. SES: percentage receiving free lunch: inner-city 89.7 % - urban 24 % - suburban 21.8 % - rural 33.6 %) |
| **Type of school (private, boarding, public)** | Public school | Public School | Public school |
| **Duration of class size reduction** | 4 years (STAR) | 4 years (from 1985-1989) | 4 years (from 1985-1989) |
| **Class size (divide into treated/comparison)** | Small classes: 13-17 students. Regular classes: 22-26 students and regular classes w. teacher aid | Small classes: 13-17 students. Regular classes: 22-26 students and regular classes w. teacher aid | Small classes: average 15 students, regular classes: average 22 students and regular classes w. teacher aid |
| **Intensity (size of reduction)** | Not specified | Not specified | 7 |
| **Precision of class size measure (is it constant per subject/during the day)** | Constant | Constant | Constant |
| **Time period covered by analysis (divide into treatment/follow up)** | 1985-1990 | 4 years: 1985-1989 (treatment in 1985-1989, no follow up) | 3 years (uncertain whether treatment is in 1985-1988 or 1986-1989) |

| Study | Finn, 1998 | Finn, 1999 | Finn, 2001 |
| --- | --- | --- | --- |
| **Participant characteristics (age, gender, grade level, socio economic status, ethnicity)** | Reviewing data and results mainly from Project STAR (Grade: K-3) and the Lasting Benefits Study (students tracked until 10th grade). | Grade: K-8.Gender: Not reported. Ethnicity specified as white vs. minority: no data on distribution. SES: Not reported. | Data from Project STAR. Kindergarten: 31,8 % minority students, 48,3 % receiving free lunch, Grade 1: 33,1 % minority students, 50,2 % receiving free lunch, Grade 2: 34,6 % minority students, 48,8 % receiving free lunch, Grade 3: 33,4 % minority students, 48,4 % receiving free lunch. Of the students identified as minority, 98.7 % were African American. Age and gender not reported (p. 149) |
| **Type of school (private, boarding, public)** | Public school | Public School | Public School |
| **Duration of class size reduction** | 4 years | 4 years (from 1985-1989) | 4 years (K-3, data from Project STAR) |
| **Class size (divide into treated/comparison)** | Small classes: 13-17 students Regular classes: 22-25 students Regular classes with full-time teacher's aide: 22-25 students | Small classes: 13-17 students. Regular classes: 22-26 students and regular classes w. teacher aid | Median class sizes (p. 149). Small classes: Kindergarten: 15 pupils - Grade 1: 15 pupils - Grade 2: 15 pupils - Grade 3: 16 pupils, Regular classes: Kindergarten: 22 pupils - Grade 1: 22 pupils - Grade 2: 23 pupils - Grade 3: 24 pupils, Regular-with-aide classes: Kindergarten: 23 pupils - Grade 1: 23 pupils - Grade 2: 23 pupils - Grade 3: 24 pupils |
| **Intensity (size of reduction)** | Not specified | Not specified | Not specified. |
| **Precision of class size measure (is it constant per subject/during the day)** | Constant | Constant | Constant |
| **Time period covered by analysis (divide into treatment/follow up)** | STAR: 1985-1989. LBS: 1990-1994 (though in the school year 1995/96 students were still being tracked) p.18 | 4 years: 1985-1989 (treatment in 1985-1989, no follow up) | Project STAR: 1985-1989 |

| Study | Finn, 2005 | Folger, 1989 | Galton, 2012 |
| --- | --- | --- | --- |
| **Participant characteristics (age, gender, grade level, socio economic status, ethnicity)** | STAR data, high school follow up. Grades KG-3 treatment. Percentage male 49.8, Percentage minority 31.6, Percentage free lunch 55.8 | Grade: K-3. Gender: Not reported. SES: specified as high vs. low. Ethnicity: Not reported. | Grade: Unclear; Gender, ethnicity, SES: Not reported. |
| **Type of school (private, boarding, public)** | Public School | Public School | Public School |
| **Duration of class size reduction** | 4 years (from 1985-1989) | 4 years (from 1985-1989) | Not reported |
| **Class size (divide into treated/comparison)** | Small classes: 13-17 students. Regular classes: 22-26 students and regular classes w. teacher aid | Small classes: 13-17 students and regular classes: 21-28 students | Small classes: < 25 pupils; normal/large classes: 38 pupils |
| **Intensity (size of reduction)** | Not specified | Not specified | 25 vs. 38 (13) |
| **Precision of class size measure (is it constant per subject/during the day)** | Constant | Constant | Not reported |
| **Time period covered by analysis (divide into treatment/follow up)** | 4 years: 1985-1989 (treatment in 1985-1989, follow up in high school 1997-2011) | 4 years: 1985-1989 (treatment in 1985-1989, no follow up) | Both treatment and follow up: 4 years (2004-2008) |

| Study | Gerritsen, 2017 | Gilman, 1988a | Gilman, 1988b |
| --- | --- | --- | --- |
| **Participant characteristics (age, gender, grade level, socio economic status, ethnicity)** | Data from longitudinal biannual PRIMA project (649). Pupils in grades 2, 4 6 and 8 are tested. 623 twin pairs, which have been assigned to different classrooms, are sampled: 448 same sex pairs (219 pairs of boys/229 pairs of girls). 173 opposite sex pairs identified. 2 twin pairs have missing information on gender. Distribution of twin pairs in grades: 235 pairs in grade 2, 175 pairs in grade 4, 132 pairs in grade 6 and 81 pairs in grade 8 (p. 650). Gender distribution in the total sample of twins is: 0.49 (SD: 0.50). Information on SES and age not found. | Grade: 1. Gender, Ethnicity and SES: Not reported. | Elementary students enrolled in first grade of three schools of the North Gibson School Corporation in Princeton, Indiana. No descriptive characteristics reported. |
| **Type of school (private, boarding, public)** | Public School | Not reported | Unclear |
| **Duration of class size reduction** | Not reported. | 4 years (from 1984-1988) | CSR during school year 1984/85. Students tested in March, April and May '85. |
| **Class size (divide into treated/comparison)** | Average class size for grade 2 (twin sample): 24,07, Average class size for grade 4, 6 and 8 (twin sample): 23,54, Average class size for the total twin sample: 23,74, Average Class size for total PRIMA sample: 24,28 (651) | Small class avg.: 18 students, large class average: 23,7 students | School year 1983/84: average of 23.8 students (range 20-26). School year 1984/85: maximum 18 students |
| **Intensity (size of reduction)** | Not applicable. Comparison of existing class sizes | 5 | Comparison of two different first grade years. |
| **Precision of class size measure (is it constant per subject/during the day)** | Not reported | Not reported | Constant/measured by enrolment in 1st grade |
| **Time period covered by analysis (divide into treatment/follow up)** | All six waves of PRIMA survey used, resulting in data from 1994 to 2005 (650) | 5 years: 1983-1988 (treatment in treated category from 1984-1988, control group from 1983-1984, no follow up) | 1983-1985 |

| Study | Haenn, 2002 | Hallinan, 1985 | Hanushek, 1999 |
| --- | --- | --- | --- |
| **Participant characteristics (age, gender, grade level, socio economic status, ethnicity)** | Kindergarten. Treatment schools are all inner city schools with large proportions of disadvantaged students. Control schools not mentioned | Grade: Not reported. Gender: Not reported. SES: Black students primarily from lower- to lower middleclass backgrounds, white and Asian/Chicano students from lower-middle to upper-middleclass backgrounds. Ethnicity: 55% black, 38 % white and 7 % Asian or Chicano. | Data from Project STAR. Students followed from Kindergarten through third grade. In the sample, 33 % of the experimental students were Black (p. 151) |
| **Type of school (private, boarding, public)** | Public school | Public school and private school | Public School |
| **Duration of class size reduction** | One year (for the relevant analysis) | Test score results from diverse standardized tests administered to the students in October and May. | 4 years (K-3, data from Project STAR) |
| **Class size (divide into treated/comparison)** | The average class size for the treated schools ranged from 14 to 21 for K-2 (we use only K). The average class size for the comparison schools ranged from 13.9 to 20-3 for K-2 grades. | 24 pupils pr. class to 37 students pr. class | Small: 13-17; Regular: 20-25; Regular-with-aide: 22-25 (p. 150) |
| **Intensity (size of reduction)** | Not reported | 24 vs. 37 (13) | Not specified. |
| **Precision of class size measure (is it constant per subject/during the day)** | Not reported | Not reported | Constant |
| **Time period covered by analysis (divide into treatment/follow up)** | Treatment at school level begins in 1994/1995 and data is probably from 2001 | 1 year | Project STAR: 1985-89 |

| Study | Harvey, 1994 | Hirschfeld,2016 | Hojo, 2011 |
| --- | --- | --- | --- |
| **Participant characteristics (age, gender, grade level, socio economic status, ethnicity)** | Grade: K-1. Gender: Kindergarten: 69 % male, Grade 1: 62 % male. Ethnicity: (data from 1985-86 - retained): 61 % white- non-retained: 59 % white. SES: K: 63,2 % receiving free lunch - Grade 1: 69,2 % receiving free lunch. | Participants attended a public middle school in Annapolis, Maryland. Students were 11-12 years old, and were enrolled in sixth grade. Gender distribution was 50% male and 50% female. Distribution of ethnicity not reported, but it is stated that: "The population consisted of a mix of African American, Hispanic, and White students." (p. 8). 40 % of the participants received free lunch/reduced price meals. 30 % were English Language Learners. 30 % received Special Education services (p. 9) | Grade: 8. Gender: 50 % males. Ethnicity and SES: Not reported/unclear. |
| **Type of school (private, boarding, public)** | Public school | Public School (8) | Public school |
| **Duration of class size reduction** | 2 years (students retained in kindergarten 1984-1985 and students retained in 1. grade 1985-1986) | Not reported. . | Not reported |
| **Class size (divide into treated/comparison)** | Small classes 13-17 students, regular classes 21-25 students and regular classes w. teacher aid | Average class sizes (p. 12): Language and Literature: 21,50 (SD: 3,13), Math: 23,05 (SD: 3,54) , Science: 27,98 (SD: 3,73), Individuals and Societies: 27,91 (SD: 2,96) | Unclear |
| **Intensity (size of reduction)** | 12 | Not applicable. Comparison of existing class sizes | Unclear |
| **Precision of class size measure (is it constant per subject/during the day)** | Not reported | Subject divided class sizes examined: Language and Literature, Math, Science, Individuals and Societies (p. 12) | Not reported |
| **Time period covered by analysis (divide into treatment/follow up)** | 5 years: 1984-1989 (treatment in 1984-1986, follow up 1989) | Survey conducted in April 2016 (Appendix B, p. 27) | 1 year (2007) |

| Study | Hojo, 2013 | Hudson, 2011 | Iacovou, 2002 |
| --- | --- | --- | --- |
| **Participant characteristics (age, gender, grade level, socio economic status, ethnicity)** | Grade: 4. Gender: F: 48%. Ethnicity: NR. SES: 3.8% | Data from National Education Longitudinal Study (NELS). Base year sample consists of 8th grade students (p. 16). Follow up in 10th and 12th grade (p. 17). Gender: 52 % females. Ethnicity: 5 % Asian, 9 % Black, 11 % Hispanic. Years of education for parents: Mean=14.05, SD=2.3. 16 % are from a single parent family. (p. 18) | Data from NCDS. All children born in the week of 3-9 March, 1958 (p. 262). Female: 0,489. In class: % of parents in social classes I & II: 23.63. In class: % of parents in social class V: 21.64. Fathers social class, class III (manual): 0,436. Ethnicity not reported (p. 288) |
| **Type of school (private, boarding, public)** | Public school | Both public and private schools (p. 16) | Public School (p. 264) |
| **Duration of class size reduction** | 1 year | Not reported. . | Not reported. . |
| **Class size (divide into treated/comparison)** | Unclear (max= more than 40.) | 10th grade, Reading: 22.61 (SD: 6.3). 10th grade, Math: 23.37 (SD: 7.1). 10th grade, Science: 23.89 (SD: 7.6). 12th grade, Math: 22.98 (SD: 6.9). 12th grade, Science: 23.96 (SD: 7.7). No measures of class sizes in 8th grade. (p. 18) | Mean class size in sample: 36,075 (SD: 5,433). Minimum is 20 students, and maximum is 45 students per class (p. 288) |
| **Intensity (size of reduction)** | Unclear | Not applicable. Comparison of existing class sizes | Comparison of existing class sizes |
| **Precision of class size measure (is it constant per subject/during the day)** | Not reported | Class size measured per subject: Reading, Mathematics and Science (p. 17-18) | Unclear. |
| **Time period covered by analysis (divide into treatment/follow up)** | Treatment: 1 year (2003). | 1988: Base year sample, 8th grade. 1990: 1st follow up, 10th grade. 1992: 2nd follow up, 12th grade. Continued in 1994, but no measures reported for this year. (p. 17) | Time period covered: 1958 (Birth). 1965: 1st follow up - wave 1/children aged 7. 1969: 2nd follow up - wave 2/children aged 11. 1973: 3rd follow up - wave 3/children aged 16 (p. 263) |

| Study | Iversen, 2013 | Jackson, 2013 | Jacobs, 1987 |
| --- | --- | --- | --- |
| **Participant characteristics (age, gender, grade level, socio economic status, ethnicity)** | Data from one cohort of fourth graders - 10 years old - in Norwegian elementary schools (306). Focuses on disadvantaged children. Most of schools at the first kink of 28 students are located in sparsely populated rural areas that are characterized by long traveling distances. (P. 307). The average educational level is between 4 and 5 for both mother and father on a 1-8 scale. The average parent has slightly more than 12 years of education. 74% of students live with both their biological parents, 26% of students live with only one biological parent or someone else (p.311). | Treatment/small classes: Grade: K and grade 1. Gender: 49 % female. SES: 49 % receiving free or reduced-priced lunch. Ethnicity: 32 % black students. | Grade: K-8. Gender: Not reported. Ethnicity specified as white, black, others: distribution unclear. SES specified through lunch eligibility: distribution unclear |
| **Type of school (private, boarding, public)** | Public School (306) | Public school | Public School |
| **Duration of class size reduction** | 4 years: 2000-2003 (p. 308) | 2 years | 2 years (from 1985-1987) |
| **Class size (divide into treated/comparison)** | Average class size 2003: 20.13 (SD 5.64) (p. 309). Small classes at the first kink are 14-17 students and the small class at the second kink are 19-21 students (p. 307). | Small classes: 13-17. Regular classes: 22-26. Regular classes w. teacher aide | Small classes: average 15 students, regular classes: average 25 students and regular classes w. teacher aid |
| **Intensity (size of reduction)** | In analysis, classes in the intervals of 11-18 and 19-27 students are used (p. 320) | Not specified | 10 |
| **Precision of class size measure (is it constant per subject/during the day)** | Constant | Constant | Constant |
| **Time period covered by analysis (divide into treatment/follow up)** | School year 2003/2004 | Treatment: 2 years (1985-1987) | 2 years: 1985-1987 (treatment in 1985-1987, no follow up) |

| Study | Jakubovski, 2006 | Konstantopoulos, 2008 | Konstantopoulos, 2009 |
| --- | --- | --- | --- |
| **Participant characteristics (age, gender, grade level, socio economic status, ethnicity)** | Grade 6; Gender, ethnicity, SES: Not reported. | Grade: K-3. Gender: not reported. Ethnicity specified (majority vs. minority, but no data on distribution. SES: specified through lunch eligibility (no data on distribution). | The students were followed for five years after the experiment (grades 4-8). The effects of small classes were examined in grade 3 on the achievement gap in subsequent grades 4-8; as well as the cumulative effects of small classes in grades kindergarten to 3 on the achievement gap in subsequent grades 4-8. Age, gender, SES, ethnicity: Not reported. |
| **Type of school (private, boarding, public)** | Public school | Public school | Public school |
| **Duration of class size reduction** | Not reported | 4 years (from 1985-1989) | From 0-4 years |
| **Class size (divide into treated/comparison)** | Unclear | Small classes: 13-17 students. Regular classes: 22-26 students and regular classes w. teacher aid | Small classes: 13-17 students. Larger classes: 22-26 and larger classes with full-time classroom aide. |
| **Intensity (size of reduction)** | Unclear | Not specified | Not specified |
| **Precision of class size measure (is it constant per subject/during the day)** | Not reported | Constant | Constant |
| **Time period covered by analysis (divide into treatment/follow up)** | 2 years (2002-2004) | 4 years: 1985-1989 (treatment in 1985-1989, no follow up) | Treatment: 4 years (Project STAR); Follow up: for 5 subsequent years after the experiment |

| Study | Konstantopoulos, 2011 | Konstantopoulos, 2014 | Konstantopoulos, 2016 |
| --- | --- | --- | --- |
| **Participant characteristics (age, gender, grade level, socio economic status, ethnicity)** | Age: NR; Grade: K-3. Gender, ethnicity, SES: Not reported. | 4th graders, both genders (female 50 %), ethnicity specified (Greece spoken at home or not - Greece spoken at home 90 %), SES specified (family size, items at home, parents education - parents with college education 20% - no data on other variables) | 4th and 8th grade data from TIMSS 2003/2007 from Cyprus. TIMSS 2003: 4th grade: Female 49.2 %; Age 9.9 years // 8th grade: Female 48.6 %; Age 13,775 years. TIMSS 2007: 8th grade: Female 49,9 %; Age 13,845 years (table 3, p. 99) |
| **Type of school (private, boarding, public)** | Public school | Public and private school | Not reported. |
| **Duration of class size reduction** | 4 years (Project STAR) | Not reported | Not reported. . |
| **Class size (divide into treated/comparison)** | Small classes: 13-17. Larger classes: 22-26 | Average class size: 18 students, no reference group | TIMSS 2003 - Fourth grade: 21,767 (SD: 5,716, min: 6/max: 32). TIMSS 2003 - Eight grade: 25,994 (SD: 3,238, min: 14/max: 32). TIMSS 2007 - Eight grade: 23,544 (SD: 2,715, min: 13/max: 30) (table 2, p. 93) |
| **Intensity (size of reduction)** | Not specified | Not reported | Not applicable. Comparison of existing class sizes |
| **Precision of class size measure (is it constant per subject/during the day)** | Constant | Not reported | Unclear. Class size reported by teachers in sampled classrooms (p. 93) |
| **Time period covered by analysis (divide into treatment/follow up)** | Treatment: 4 years | 1 year: 2001 (data from 2001, no follow up) | TIMSS survey round 2003 and 2007. |

| Study | Konstantopoulos, 2016 | Krueger, 1999 | Krueger, 2001a |
| --- | --- | --- | --- |
| **Participant characteristics (age, gender, grade level, socio economic status, ethnicity)** | Data from TIMSS 2011. Fourth grade mathematics achievement (509). 14 countries selected: Austria, Croatia, Czech Republic, Denmark, Germany, Hungary, Italy, Lithuania, Malta, Portugal, Romania, Slovak Republic, Slovenia, and Spain. (505). Descriptive statistics (gender, age, SES) for each of the 14 countries are found in Table 2, page 516-17. | Treatment Kindergarten: Age: 5.44; Gender: Not Reported. Grade: K-3; SES: 47% receiving free lunch. Ethnicity: 32% not white. Treatment grade 1: Age: 5.78; Gender: Not Reported. Grade: K-3. SES: 59% receiving free lunch. Ethnicity: 38% not white. Treatment grade 2: Age: 5.94. Gender: Not reported. Grade: K-3. SES: 66 % receiving free lunch. Ethnicity: 47% not white. Treatment grade 3: Age: 5.95. Gender: Not Reported. Grade: K-3. SES: 60 % receiving free lunch. Ethnicity: 34% not white. Comparison kindergarten: Age: 5.43. Gender: Not reported. Grade: K-3. SES: 48 % receiving free lunch. Ethnicity: 33% not white. Comparison grade 1: Age: 5.86. Gender: Not reported. Grade: K-3. SES: 62% receiving free lunch. Ethnicity: 44% not white. Comparison grade 2: Age: 6. Gender: Not Reported. Grade: K-3. SES: 63% receiving free lunch. Ethnicity: 46% not white. Comparison grade 3: Age: 5.92. Gender: Not Reported. Grade: K-3. SES: 64% receiving free lunch; Ethnicity: 43% not white. | Both treatment/comparison: Age: 5.4 at the beginning of K. Grade: kindergarten and grade 1. Gender: 50 % female. SES: 49 % receiving free or reduced-priced lunch. Ethnicity: 31 % black students. |
| **Type of school (private, boarding, public)** | Not reported. | Public school | Public school |
| **Duration of class size reduction** | Not reported. . | 4 years | 4 years (Project STAR) |
| **Class size (divide into treated/comparison)** | Average class size ranges from 19 (Austria) to 23 (Spain) across countries. Average class size overall is approximately 20 students per class. (518) | Small: 13-17; regular: 22-25; regular with full-time aide (22-25) | Small classes: 13-17. Regular classes: 22-26. Regular classes w. a teacher aide |
| **Intensity (size of reduction)** | Not applicable. Comparison of existing class sizes | Not specified | Not specified |
| **Precision of class size measure (is it constant per subject/during the day)** | Unclear. Class size reported by teachers in sampled classrooms (p. 508) | Constant | Constant |
| **Time period covered by analysis (divide into treatment/follow up)** | TIMSS: 2011 | Treatment: 1985-1989; no follow up | Treatment 4 years (1985-1989). Follow-up in 1998 (in high school or college). |

| Study | Krueger, 2001b | Krueger, 2002 | Lavy, 2001 |
| --- | --- | --- | --- |
| **Participant characteristics (age, gender, grade level, socio economic status, ethnicity)** | Grade: K-8 and to the age of ACT/SAT college entrance exams. Gender, SES and Ethnicity unclear. | Sample: 556 pupils from 16 schools in Stockholm (64) Pupils followed from the end of 5th grade and throughout 6th grade. Female: 0,50, Non-Swedish parents: 0,23, Family income (log): 12,60, socio-economic index: 0,0 (67) | Elementary schools. P. 6 in pdf. 4th and 5th grade. 1,039 Jewish public (secular and religious) schools. Data include a school-level index of students socioeconomic status measured as the per cent of disadvantaged students, and variables identifying ethnicity (Jewish, Arab) and religious affiliation (secular, religious). |
| **Type of school (private, boarding, public)** | Public school | Not specified | Public schools |
| **Duration of class size reduction** | 4 years (Project STAR) | Not reported. . |  |
| **Class size (divide into treated/comparison)** | Small classes: 13-17. Regular classes: 22-26. Regular classes w. a teacher aide | Comparison of existing class sizes. Average class size, fifth grade (math): 22.93. Average class size, sixth grade (math): 19,9 (p. 67) | Standard class: 21-40 students. Then they observe class with 9-21 students and class with less than 10 students. In table 1 class size is compared to test scores, with small classes to be less than or equal to 31 and large classes to be larger than 31 (measured by number of students) |
| **Intensity (size of reduction)** | Not specified | Not applicable. Comparison of existing class sizes | Not applicable. Comparison of existing class sizes |
| **Precision of class size measure (is it constant per subject/during the day)** | Constant | Class size measures are constructed from math classes. (p.67) | Unclear |
| **Time period covered by analysis (divide into treatment/follow up)** | Treatment 4 years (1985-1989). Follow-up in grade 8 and at the age of ACT/SAT college entrance exams | 1998 (spring/fall), 1999 (spring) (65) | 1991 |

| Study | Levin, 2001 | Li, 2015 | Li, 2017 |
| --- | --- | --- | --- |
| **Participant characteristics (age, gender, grade level, socio economic status, ethnicity)** | Grade: 4, 6 and 8. Gender and weight factors accounting for SES: The weight factor ranges from 1 to 1.9: 1.9-pupils are pupils with foreign-born parents with no or low income. 1.7-pupils are pupils whose parents are transients. 1.4-pupils live in a boarding school or a foster home. 1.25-pupils are pupils of whom one parent has at most an education at VBO-level of education. All other pupils have a weight equal to one. | Data from TIMSS 2011. Fourth grade data (p. 10). Descriptive statistics for countries (gender, age, SES) found in table 1.2, p. 21. | Fourth-grade sample from TIMSS 2011. 14 countries selected: Austria, Croatia, Czech Republic, Denmark, Germany, Hungary, Italy, Lithuania, Malta, Portugal, Romania, Slovak Republic, Slovenia, and Spain. Descriptive statistics (gender, age, SES) for each of the 14 countries are found in Table 4, page 305. |
| **Type of school (private, boarding, public)** | Public school | Not identified (p. 19) | Not reported. |
| **Duration of class size reduction** | Not reported | Not reported. . | Not reported. . |
| **Class size (divide into treated/comparison)** | Grade 4: 25-26, grade 6 & 8: 26-27 | Average class size for participating countries found in table 1.2, p. 21. Average class sizes for European countries are much smaller than for Asian countries. Austria has the smallest class size (19 students), while Spain has the largest in Europe (23 students). The largest class size average in Asia is found in Singapore (37 students). (p. 20) | Teacher reported class size (mean) for the 14 countries is reported in Table 3, page 304. |
| **Intensity (size of reduction)** | Unclear | Not applicable. Comparison of existing class sizes | Not applicable. Comparison of existing class sizes |
| **Precision of class size measure (is it constant per subject/during the day)** | Not reported | Unclear. Class size reported by teachers in sampled classrooms (p. 11) | Unclear. Class size reported by teachers in sampled classrooms (p. 297/304) |
| **Time period covered by analysis (divide into treatment/follow up)** | 1 year (1994/1995) | TIMSS survey round 2011 | 2011 (TIMSS) |

| Study | Lindahl, 2005 | Ma, 2006 | Maier, 1997 |
| --- | --- | --- | --- |
| **Participant characteristics (age, gender, grade level, socio economic status, ethnicity)** | Grade: 5 & 6. Gender: 50% female: SES. Unclear. Ethnicity: Non-Swedish parents = 23 %. | PRIMA dataset. Sample consist of pupils from grade 4, 6 and 8. Gender (female=1): 0.50, SES-status (min=0 / max=1): 0.53, Age and ethnicity not reported. (p. 491) | Kindergarten and 1. Grade. 49% girls, 49% white, 31% not eligible for subsidized lunch, 8% English as second language, 13% with exceptional education needs, 8% with individualized educational plan, 14% referred to M-team, 3% repeating grade |
| **Type of school (private, boarding, public)** | Public school | Both private and public school. Share of public school is 72 % (p. 491) | Public school |
| **Duration of class size reduction** | Test score results from tests administered once in 5. Grade and twice in 6. Grade. | Not reported. . | 1 year |
| **Class size (divide into treated/comparison)** | Class size math 5th grade: 23. Class size math 6th grade: 20. Class size regular 5. Grade: 25. Class size regular 6th grade: 23. | Class size varies between 5 and 39 students. Average Class size is 23.81 (SD: 6.46). 70 % of classes are between 15 and 35 students per class. (p. 491) | Treated: 15 students; control: not reported |
| **Intensity (size of reduction)** | 20 vs. 25 (5) | Not applicable. Comparison of existing class sizes | Not specified |
| **Precision of class size measure (is it constant per subject/during the day)** | Varies. Math class: 20-23 pupils’ pr. class. Regular class: 23-25 pupils | Unclear. It is stated that background data are gathered through parents and teachers (p.491) | Not reported |
| **Time period covered by analysis (divide into treatment/follow up)** | 1 year | School year 1994/1995 (p. 490) | 1996-1997 |

| Study | Maples, 2009 | McGiverin, 1989 | Mckee, 2010 |
| --- | --- | --- | --- |
| **Participant characteristics (age, gender, grade level, socio economic status, ethnicity)** | Data (End-of-Grade test) was retrieved from North Carolina Department of Public Instruction for the 2006/2007 school year (p. 57). Includes 33 middle schools in Sandhill Region, North Carolina. Grades 6 through 8 (p. 58). | Data from Indiana's Project Prime Time. Second grade pupils examined. Majority white and resides in areas of 1,500 or fewer residents. Majority of parents are wage/salary workers, local government employee or farmers. These parents have completed a median of 12.3 years of school. Average family income from 17.000 to 23.000 dollars. 6-10 % of households are below poverty level. Participant characteristics are comparable to state demographics (p. 50) | Data from Project STAR. Small Classes: 47.1 % receives free lunch (SD 0,499), 31.6 % are non-white (SD 0,465), mean of age is 5.47 years (SD 0.34). Regular Classes: 48.6 % receives free lunch (SD 0.5), 33.2 % are non-white (SD 0,471), mean of age is 5.46 years (SD 0.34). Gender not reported (table, 1, p. 28) |
| **Type of school (private, boarding, public)** | Public School (p. 57) | Public School | Public School |
| **Duration of class size reduction** | Not reported. | 2 years (p. 50) | 4 years (K-3, data from Project STAR) |
| **Class size (divide into treated/comparison)** | Small classes: 0-15 students per class. Medium classes: 16-24 students per class. Large classes: 25 or more student per class (p. 58) | Prime time classes who experienced class size reduction were compared to prime time classes with no class size reduction. Second grade year 1984-85: Average 26.6 students per class (no class size reduction). Second grade year 1985-86: Average 19,1 students per class (class size reduction since grade 1 - possibly a cumulative effect) (p. 50) | Small classes: 13-17, Regular: 22-25, Regular-with-aide: 22-25 (p. 13). Mean class size (small): 15,12 (SD: 1,50), Mean class size (regular): 22,53 (SD: 2,21) (table 1, p. 29) |
| **Intensity (size of reduction)** | Not applicable. Classes were grouped to analyse differences in end-of-grade scores. | Not specified. | Mean of 7 |
| **Precision of class size measure (is it constant per subject/during the day)** | Unclear/Not reported (p. 58). | Constant | Constant. |
| **Time period covered by analysis (divide into treatment/follow up)** | School year: 2006/2007 | School years: 1984-85 and 1985-86 (p. 50) | Project STAR: 1985-1989 |

| Study | McKee, 2015 | Merritt, 2011 | Milesi, 2006 |
| --- | --- | --- | --- |
| **Participant characteristics (age, gender, grade level, socio economic status, ethnicity)** | Data from Project STAR. Participant characteristics for small classes (kindergarten): Free lunch: 47,1 %; Non-white: 31,6 %; Age: 5,47, Participant characteristics for regular classes (kindergarten): Free lunch: 48,6 %, Non-white: 33,2%; Age: 5,46 (p. 1274) | Age: Not reported. Gender: 51.2% male. Grade: 3. SES: 26 % designated free/reduced lunch. Ethnicity: 48 % Hispanic, 10 % Caucasian, 22 % African American and 17 % Asia America. | Grade: Kindergarten. Gender: 50 % male. Ethnicity: 15 % black, 18 % Hispanic, 2 % Asian, 5 % other races. SES: family (composite) (small classes 0,03 above mean, large classes 0,03 below mean - range -4,75-3,69) |
| **Type of school (private, boarding, public)** | Public School | Public school | Public and private kindergartens |
| **Duration of class size reduction** | 4 years (K-3, data from Project STAR) | Not applicable | 1 year (from 1998-1999) |
| **Class size (divide into treated/comparison)** | Small classes (treatment): 13-17 students (mean: 15,1), Regular classes (control): 22-25 students (mean: 22,5) (p. 1274) | The study compares existing class sizes with achievement tests in the third grade. | Small classes: 17 or fewer students (lowest quantile), regular classes: 18-23 students, (reference), large classes: 24 or more students (highest quantile) |
| **Intensity (size of reduction)** | Mean is 7 | Unclear | At least 7 |
| **Precision of class size measure (is it constant per subject/during the day)** | Constant | Not applicable | Not reported |
| **Time period covered by analysis (divide into treatment/follow up)** | Project STAR: 1985-1989 | Treatment: 3 years | 1 year: 1998-1999 (treatment in 1998-1999, no follow up) |

| Study | Molnar, 1998 | Molnar, 1999a | Molnar, 1999b |
| --- | --- | --- | --- |
| **Participant characteristics (age, gender, grade level, socio economic status, ethnicity)** | Kindergarten to 2. Grade. 49% girls, 47% white, 22% not eligible for subsidized lunch, 7% English as second language, 13% with exceptional education needs, 8% with individualized educational plan, 14% referred to M-team, 3% repeating grade | Kindergarten to 3. Grade. 49% girls, 44% white, 36% not eligible for subsidized lunch, 8% English as second language, 13% with exceptional education needs, 13% with individualized educational plan, 13% referred to M-team, 2% repeating grade | Kindergarten to 1 grade. Only report participant characteristic for all grades for the school years 1996-1997 and 1997-1998 (see Maier et al., 1997 and Molnar et al., 1998) |
| **Type of school (private, boarding, public)** | Public school | Public school | Public school |
| **Duration of class size reduction** | 1-2 years | 1-3 years | 1-2 years |
| **Class size (divide into treated/comparison)** | Treated: 15 students; control: not reported | Treated: 15 students; control: not reported | Treated: 15 students; control: not reported |
| **Intensity (size of reduction)** | Not specified | Not specified | Not specified |
| **Precision of class size measure (is it constant per subject/during the day)** | Not reported | Not reported | Not reported |
| **Time period covered by analysis (divide into treatment/follow up)** | 1996-1998 | 1996-1999 | 1996-1998 |

| Study | Molnar, 2001 | Moshoeshoe, 2015 | Mosteller, 1995 |
| --- | --- | --- | --- |
| **Participant characteristics (age, gender, grade level, socio economic status, ethnicity)** | Kindergarten to 3. Grade. 48-49% girls, 44-49% white, 31-36% not eligible for subsidized lunch, 7-8% English as second language, 10-13% with exceptional education needs, individualized educational plan not reported, 10-14% referred to M-team, 1-3% repeating grade | Data from SACMEQ II collected in year 2000 (6). Grade 6 students are sampled, N=3155 (7). Participant characteristics (p. 9): Gender (female): 0.556 (SD: 0.497); Age in months: 169.63 (SD: 22.15); SES-index: -1.07e-09 (SD: 2.011); Speaks English at home: 0,707 (SD: 0,455); Once repeated a class: 0,608 (SD: 0,488); School location (urban): 0,351 (SD: 0,477). | Grade: K-3. Gender: Not reported. SES specified in poor, well-to-do: distribution unclear. Ethnicity specified as minority, majority, mixed: distribution unclear. |
| **Type of school (private, boarding, public)** | Public school | Unclear. | Public School |
| **Duration of class size reduction** | 1-4 years | Not reported. . | 4 years (from 1985-1989) |
| **Class size (divide into treated/comparison)** | Treated: 15 students; control: not reported | Average class size is 44,9 students per class (SD: 18,09) (p. 9) | Small classes: 13-17 students. Regular classes: 22-25 students and regular classes w. teacher aid |
| **Intensity (size of reduction)** | Not specified | Not applicable. Comparison of existing class sizes | Not reported |
| **Precision of class size measure (is it constant per subject/during the day)** | Not reported | Not specified. Class size reported by the teacher: total number of all students in a class, including those who were absent during the data collection (11) | Constant |
| **Time period covered by analysis (divide into treatment/follow up)** | The SAGE statute [s. 118.43] requires participating schools to (a) reduce class size to 15 in grades kindergarten and one in 1996-97, grades kindergarten to two in 1997-98, and grades kindergarten through three in 1998-99 to 2000-2001 | Survey (SACMEQ II) conducted in 2000 | 4 years: 1989-1993 (treatment in 1989-1993, no follow up) |

| Study | Munoz, 2001 | Murdoch, 1986 | Maasoumi, 2005 |
| --- | --- | --- | --- |
| **Participant characteristics (age, gender, grade level, socio economic status, ethnicity)** | Age: Treatment: 9/Comparison: 9. Gender: treatment: 45% female, comparison: 60% female. Grade: 3. SES: 59.9 % eligible for free or reduced-price lunch. Ethnicity: 34,8 % minority students | Students in grades 1-5 (N=1624, p. 30) attending the eight elementary schools in Madison School District 321, Rexburg, Idaho (p.23). 67 % of students attended city schools, and 33% attended rural schools (p. 31). Participant characteristics: "A large percentage of the population of Madison School District shares basic middle class values, belongs to the area's predominant religion, and sends a large majority of their graduating seniors to college" (p. 31). Data means and standard deviations were not available. | Grade: 8. Gender, ethnicity, SES: Not reported. |
| **Type of school (private, boarding, public)** | Unclear | Public School | Public and private schools |
| **Duration of class size reduction** | 1 year | Not reported. | Not reported |
| **Class size (divide into treated/comparison)** | Treatment (small classes): less than 19; comparison: more than 18 (‘usual’ size is 24, p. 25) | Class size varied from 16 to 36 students (p. 35) | Small classes: 19 or less, medium classes: 20-30 and large classes: more than 30. |
| **Intensity (size of reduction)** | Unclear | Not applicable. Comparison of existing class sizes | Less than 19 to more than 30 |
| **Precision of class size measure (is it constant per subject/during the day)** | Constant pr. subject | Unclear. Class size reported by "homeroom" teacher (p. 35) | Not reported |
| **Time period covered by analysis (divide into treatment/follow up)** | Treatment: year 1999-2000 | School year: 1984-1985 (p. 23) | 3 years (initial study 1988, first follow up 1990 and second follow up 1992). |

| Study | Nandrup, 2016 | NICHD, 2004 | Nye, 1992 |
| --- | --- | --- | --- |
| **Participant characteristics (age, gender, grade level, socio economic status, ethnicity)** | Danish registry data for all pupils in grade 2-8 (p. 85). Descriptive measures of participant characteristics not reported. | Grade: 1. Gender: control group: female 47.9 %. Ethnicity: control: white non-Hispanic - non-white 19.1 %. SES: Not reported. | Data from project STAR (grade: K-3) and LBS (grade 5). Children in STAR were from inner city, suburban, urban and rural schools. Other descriptive characteristics not reported. Race for LBS-students by class size (p. 10): Small: 31,5% minority, Regular: 37,9% minority, Regular/aide: 34,7% minority |
| **Type of school (private, boarding, public)** | Public School (p. 84-85) | Not reported | Public school |
| **Duration of class size reduction** | Not reported. . | Unclear | 4 years (project STAR) |
| **Class size (divide into treated/comparison)** | Class size averages found on p. 86: Reading sample: Grade 2: 21,20 (SD: 4,06), Grade 6: 21,37 (SD: 3,91), Grade 8: 21,87 (SD: 3,58), Math (physics/chemistry) sample: Grade 3: 21,19 (SD: 4,03), Grade 6: 21,36 (SD: 3,86), Grade 8 - physics/chemistry: 21,86 (SD: 3,57) | Various treatment/comparison depending on test (WJ-R: smaller classes of 21 students, larger classes of above 21 students (from page 658-660)) | Small classes: 13-17 students. Regular classes: 22-25 students. Regular classes with full-time teacher's aide: 22-25 students |
| **Intensity (size of reduction)** | Not applicable. Comparison of existing class sizes | Unspecified | Not specified |
| **Precision of class size measure (is it constant per subject/during the day)** | Constant. | Not reported | Constant |
| **Time period covered by analysis (divide into treatment/follow up)** | School years: 2009/10 - 2011/12 (p. 85) | 2 years: 1990-1992 (treatment in 1990-1991, follow up in 1991-1992) | STAR: 1985-1989. LBS: follow up in school year 1990/91 |

| Study | Nye, 1993 | Nye, 1994 | Nye, 2000 |
| --- | --- | --- | --- |
| **Participant characteristics (age, gender, grade level, socio economic status, ethnicity)** | Data from Project STAR (grade K-3). Data from the Lasting Benefits Study, LBS (grade 4 & 5). STAR: Students from both inner city, urban, suburb and rural. Other descriptive statistics stated in Table 1, p. 21. | Data from project STAR (grade K-3). Students from inner city, urban, suburb and rural. And data from the lasting benefits study (grade 4 & 5) | Grade: 1-3. Gender: Not reported. Ethnicity specified (white, minority - minority: kindergarten 33 % - 1th grade 33 % - 2th grade 34,7 % - 3th grade 33,6 %). SES specified through lunch eligibility (SES low, SES high - low SES: kindergarten 48 % - 1th grade 50 % - 2th grade 48,8 % - 3th grade 48,3 %) |
| **Type of school (private, boarding, public)** | Public School | Public School | Public school |
| **Duration of class size reduction** | 4 years (K-3, data from Project STAR) | 4 years (K-3, data from Project STAR) | 4 years (from 1985-1989) |
| **Class size (divide into treated/comparison)** | Small classes: 13-17. Regular: 22-26. Regular-with-aide: 22-26 (p. 4) | Small: 13-17; Regular: 20-25; Regular-with-aide: 22-25 (p. 10) | Small classes: 13-17 students. Regular classes: 22-26 students and regular classes w. teacher aid |
| **Intensity (size of reduction)** | Not specified. | Not specified | Not specified |
| **Precision of class size measure (is it constant per subject/during the day)** | Constant. | Constant | Constant |
| **Time period covered by analysis (divide into treatment/follow up)** | Project STAR: 1985-1989. LBS: 1990-1992 | Project STAR: 1985-1989. LBS: 1989-90 & 1990-91 | 1985-1989 (treatment in 1985-1989, no follow up) |

| Study | Nye, 2000 | Nye, 2001a | Nye, 2001b |
| --- | --- | --- | --- |
| **Participant characteristics (age, gender, grade level, socio economic status, ethnicity)** | Grade: K-3. Gender: Not reported. Ethnicity specified (white, minority) (no data on distribution). SES specified through lunch eligibility (no data on distribution). | Grade: 9. Gender: Not reported. Ethnicity specified as majority vs. minority: no data on distribution. SES specified through lunch eligibility (no data on distribution). | Grade. 1-3. Gender: Not reported. Ethnicity specified as white vs. minority - distribution not reported. SES: specified through lunch eligibility (SES low, SES high) (no data on overall distribution, data on individual distribution to each class (end of previous year (kindergarten to 1th grade, 1th grade to 2th grade etc.), start of year) |
| **Type of school (private, boarding, public)** | Public schools | Public School | Public school |
| **Duration of class size reduction** | 4 years (from 1985-1989) | 4 years (from 1985-1989) | 4 years (from 1985-1989) |
| **Class size (divide into treated/comparison)** | Small classes: 13-17 students, regular classes: 22-26 students, regular classes w. teacher aid. | Small classes: 13-17 students. Regular classes: 22-26 students and regular classes w. teacher aid | Small classes 13-17 students, regular classes 22-26 students, regular classes v/ aid |
| **Intensity (size of reduction)** | Not specified | Not specified | Not specified |
| **Precision of class size measure (is it constant per subject/during the day)** | Constant | Constant | Constant |
| **Time period covered by analysis (divide into treatment/follow up)** | 4 years: 1985-1989 (treatment in 1985-1989, no follow up) | 10 years: 1985-1995 (treatment in 1985-1989, follow up in 1995) | 4 years: 1985-1989 (treatment in 1985-1989, no follow up) |

| Study | Nye, 2002 | Otsu, 2015 | Pollard, 1995 |
| --- | --- | --- | --- |
| **Participant characteristics (age, gender, grade level, socio economic status, ethnicity)** | Grade: 1-3. Gender: Not reported. Ethnicity specified as majority vs. minority: no data on distribution. SES specified through lunch eligibility (no data on distribution) | Data from Angrist & Lavy (1999) used (p. 103). Focus is on 4th graders. | Grade: K-3., Gender: Not reported., SES and Ethnicity: Not reported |
| **Type of school (private, boarding, public)** | Public School | Unclear. | Not reported |
| **Duration of class size reduction** | 4 years (from 1985-1989) | Unclear. | 3 years in intervals (first 1990-1992, second 1996-1997) |
| **Class size (divide into treated/comparison)** | Small classes: 13-17 students. Regular classes: 22-26 students and regular classes w. teacher aid | Unclear. | Smallest classes 1-15 students, small classes 15-20 students, regular classes 21-25 students, large classes 26-40 students |
| **Intensity (size of reduction)** | Not specified | Varies | Target ratio on kindergarten through 3th grade to 15 students per teacher, 4th through 6th grade to 22 students per class, 7th through 12th grade to 25 students per class |
| **Precision of class size measure (is it constant per subject/during the day)** | Constant | Not reported | Not reported |
| **Time period covered by analysis (divide into treatment/follow up)** | 4 years: 1985-1989 (treatment in 1985-1989, no follow up) | Test score results from June 1991 and June 1992. | 7 years: 1990-1997 (treatment interval 1990-1997, no follow up) |

| Study | Pong, 2001 | Prais, 1996 | Sanogo, 1994 |
| --- | --- | --- | --- |
| **Participant characteristics (age, gender, grade level, socio economic status, ethnicity)** | Grade 8. (13 year olds). Gender: Not reported. SES specified on individual level for each country. Ethnicity not reported. | Data from Project STAR. Grade: K-3., Gender, SES and Ethnicity: Not reported | Data from Project STAR (grade K-3) and Indiana Prime Time (grade 1-3). Participant characteristics not reported. |
| **Type of school (private, boarding, public)** | Not reported | Public school | Public School |
| **Duration of class size reduction** | Comparison between countries | 4 years (from 1985-1989) | Project STAR: 4 years Prime Time: 3 years  (p. 10) |
| **Class size (divide into treated/comparison)** | Class size based on national level: Small classes (lowest quantile), regular classes (reference), large classes (highest quantile) - (Table 1, s. 260) | Small class average: 15 students. Regular class average: 24 students. Regular classes w. teacher aid | Project STAR: Small classes: 13-17; Regular classes: 22-25; Regular-with-aide classes: 22-25. Prime Time: Small class average: 19,1 students per class; Large class average: 29,9 students per class (p. 10) |
| **Intensity (size of reduction)** | Not reported | Average of 9 | Not specified for STAR. Prime time average of 10,8 students |
| **Precision of class size measure (is it constant per subject/during the day)** | Not reported | Constant | Constant |
| **Time period covered by analysis (divide into treatment/follow up)** | 1994-1995 (data from 1994-1995) | 5 years: 1985-1990 (treatment in 1985-1989, follow up in 1990) | Project STAR: 1985-1989. Indiana Prime Time: 1984-1987 |

| Study | Schanzenbach, 2007 | Shapson, 1980 | Shin, 2011 |
| --- | --- | --- | --- |
| **Participant characteristics (age, gender, grade level, socio economic status, ethnicity)** | Data from STAR experiment. All schools were located in Tennessee, and the grades examined were Kindergarten to 3 grade. The children were 5-8 years old. Students who were male, African American or on free or reduced-price lunch were more likely both to enter and to exit project STAR. p. 206. 33.1 pct. were minority students. 31.7 were black students. 24.4 pct. of children were below poverty level. 43.4 pct. of teachers had a master degree or higher. (p. 207 table 1). | Grade: 4 and 5. Gender: 70 % female. Ethnicity: Not reported. SES: specified (high, low) (low SES 52 %). | Age: Not reported. Gender: Not reported. Grade: K-3. SES: Percentage of students receiving free or reduced-price lunch: K: 48%, 1st: 61%, 2nd: 65%, 3rd: 65%. Ethnicity: percentage of black students: K: 33%, 1st: 38%, 2nd: 46%, 3rd: 40% |
| **Type of school (private, boarding, public)** | Unclear (public) | Not reported | Public |
| **Duration of class size reduction** | 4 years | Not reported. | 4 years (K-3 grade) |
| **Class size (divide into treated/comparison)** | Small class: 13 to 17 student’s pr. teacher. Regular class: 22 to 25 student’s pr. teacher. Regular-with-aide class: 22 to 25 students with a full time teacher aide. | Small classes: 16 students, regular classes: 23 students, large classes: 30 students, largest classes 37 students | Treatment: 11-20 students; Comparison: 15-30 students |
| **Intensity (size of reduction)** | Not specified | Varies | Varies |
| **Precision of class size measure (is it constant per subject/during the day)** | Constant | Not reported | Constant |
| **Time period covered by analysis (divide into treatment/follow up)** | 1985-1989 | 2 years: 1977-1979 (treatment in 1977-1979, no follow up) | Treatment: year 1985-1989 |

| Study | Shin, 2012 | Sohn, 2015 | Tienken, 2009 |
| --- | --- | --- | --- |
| **Participant characteristics (age, gender, grade level, socio economic status, ethnicity)** | Age: Not reported. Gender: 46-49% female. Grade: K-3. SES: Not reported. Ethnicity: 33-46% black students | Follow-up data from Project STAR. Measures math/reading score in 4th, 6th, and 8th grade, and whether or not the students graduate high school and take ACT/SAT test (table 1, p. 10). The article distinguishes between effective and ineffective schools (p. 6) Descriptive statistics for effective schools (N=2778): Regular Class: Girls 45,3 %, White 47,2 %, Free lunch 64,6 %. Small Class: Girls 50.9 %, White 56.6 %, Free lunch 55.6 %. Descriptive statistics for ineffective schools (N=5882): Regular class: Girls, 47.7%; White, 70.6 %; Free Lunch, 56.9 %. Small Class: Girls, 46,8 %; White, 72,9 %; Free Lunch, 56,8 % (Table 1, p. 10) | Grade: 6-8 |
| **Type of school (private, boarding, public)** | Not reported | Public School |  |
| **Duration of class size reduction** | 4 years (K-3 grade) | 4 years (K-3, data from Project STAR) | Cohort 1: regular class size for 3 years; Cohort 2 CSR for 3 years; Cohort 3: 1 year CSR and 2 years regular class size |
| **Class size (divide into treated/comparison)** | Treatment: 11-20; Comparison: 15-30 | Regular class' and 'regular class with aide' are aggregated (p. 6) in the article. Mean class sizes for effective schools (table 1, p. 10): Regular: 22,1, Small: 17,5, Mean class sizes for ineffective schools (table 1, p. 10): Regular: 22,2, Small: 17,9 | Treatment: 12-18; comparison: 23-26 |
| **Intensity (size of reduction)** | Varies | Size of reduction for effective schools: 4.6 students. Size of reduction for ineffective schools: 4,3 students (p. 9) | Varies |
| **Precision of class size measure (is it constant per subject/during the day)** | Constant | Constant. | Constant pr. subject |
| **Time period covered by analysis (divide into treatment/follow up)** | Treatment: year 1985-1989 | Project STAR: 1985-1989 (p. 3) | Treatment: 2001-2006 |

| Study | Tillitsky, 1988 | Uhrain, 2016 | Urquiola, 2006 |
| --- | --- | --- | --- |
| **Participant characteristics (age, gender, grade level, socio economic status, ethnicity)** | Students in grade 1-3 from schools in North Gibson School Corporation, Indiana. "The community is varied in socioeconomic background and parents of the students work at a variety of occupational levels." p. 37 | Secondary students from five of nine secondary schools in South Carolina district. Representative of population in county (48). Participant characteristics reported in figures/graphs for sampled schools (percentages are read from figure): Ethnicity (figure 1 p. 48 - percentages are read from figure): White 60-88 %; Black 7-32 %; Hispanic 2-12 %; Asian 1-5 %; Other 0-4 %. Eligibility for free lunch (figure 4, p. 50/51): 33-49 % | SIMECAL data, focus on 3rd grade pupils. Participant characteristics (table 1, p. 26). Total sample: Mean age 9.2 years. Spanish as first language: 75 %. Urban private: Mean age 8.7 years. Spanish as first language 93 %. Urban public: Mean age 9.1 years. Spanish as first language: 81 %. Rural public: Mean age 9.5 years. Spanish as first language: 60 %. "Spanish-only speakers display higher SES than native language users" (p. 7). Gender not reported. |
| **Type of school (private, boarding, public)** | Unclear | Public School | Urban Private. Urban Public. Rural Public. (Rural private schools are excluded from sample) (p. 4-5) |
| **Duration of class size reduction** | 3 years. The reduced prime time cohort was 1 year behind the large class cohort. | Not reported. | Not reported. . |
| **Class size (divide into treated/comparison)** | Large class cohort, by average: Grade 1, year 83/84: 23,1 pupils, Grade 2, year 84/85: 20,5 pupils, Grade 3, year 85/86: 24,0 pupils. Reduced class (PRIME TIME) cohort, by average: Grade 1, year 84/85: 19,9 pupils, Grade 2, year 85/86: 17,4 pupils, Grade 3, year 86/87: 18,0 pupils | Not reported. | 3rd grade class size averages: Total: 29.9 (SD: 9.9). Urban private: 37.4 (SD: 9.8). Urban public: 35.5 (SD: 6.4). Rural public: 23,3 (SD: 8,4) (Table 1, p. 26) |
| **Intensity (size of reduction)** | Not specified | Not applicable. Comparison of existing class sizes | Not applicable. Comparison of existing class sizes |
| **Precision of class size measure (is it constant per subject/during the day)** | Unclear/constant | Subject divided class sizes examined (p. 56). Different class types (40 in total) within the subjects of English (8 types), Math (9 types), Science (13 types) and Social Studies (10 types) are examined (56pp) | Unclear |
| **Time period covered by analysis (divide into treatment/follow up)** | 1983-1987 | School year 2012-2013 (51) | Unclear. SIMECAL was introduced in 1993. |

| Study | Watson, 2016 | Wenfan, 2005 | West, 2006 |
| --- | --- | --- | --- |
| **Participant characteristics (age, gender, grade level, socio economic status, ethnicity)** | Data from National Assessment Plan - Literacy and Numeracy (NAPLAN) (p. 511). Students enrolled in Catholic School in Sydney Archdiocese in the years 2008-2012. NAPLAN scores covers all years tested for year 3 to 9 (p. 514-15). Gender: Male 51 %/Female: 49 %. (p. 514). Language: English 47 % / LBOTE: 53 % (p. 514) | Age: Not reported. Gender: 49.3% female. Grade: Kindergarten. SES: 17.6% receiving free or reduced-price lunch. Ethnicity: Unclear | Data from TIMMS 1995. Students enrolled in the two adjacent grades, which contained the largest proportion of 13-year olds, which are the first two grades of secondary school. Each country drew a random sample of schools.  Participant characteristics (SES, gender, age) for each country found in Table A1. |
| **Type of school (private, boarding, public)** | Unclear. | Public and private kindergartens | Not specified. |
| **Duration of class size reduction** | Not reported. . | 1 year | Not reported. . |
| **Class size (divide into treated/comparison)** | Computed class sizes ranges between 12 and 34. Most schools reporting small class sizes (12-15) are excluded (p. 512). Specific class sizes averages not reported. | Small: 9-17; medium: 18-21; large: 22-30 | Class size (mean, SD) for each country found in Table 1. |
| **Intensity (size of reduction)** | Not applicable. Comparison of existing class sizes | Varies | Not applicable. Comparison of existing class sizes |
| **Precision of class size measure (is it constant per subject/during the day)** | Unclear. | Constant pr. subject | Both grade-average class size and class sizes for Mathematics and Science are reported (Table 1) |
| **Time period covered by analysis (divide into treatment/follow up)** | 2008 - 2012 (p. 514-15) | Treatment: 1998-1999 | 1995 |

| Study | Wiermann, 2005 | Word, 1990 | Wößmann, 2006 |
| --- | --- | --- | --- |
| **Participant characteristics (age, gender, grade level, socio economic status, ethnicity)** | Data from PISA 2000 and PISA-E (tension) 2000. Students in PISA 2000 are 15 years old. Students in Pisa-E 2000 are either 15 years old, or in 9th grade (p. 7). 52 % in sample are females. SES and ethnicity not reported. Type of school students are enrolled in (German system): 34 % attend grammar schools (Gymnasium), 25 % attend intermediate schools (Realschule), 18 % attend middle school (Haupt-/Realschule), 12 attend secondary general school (Hauptschule), 10 % attend comprehensive schools (Gesamtschule) (p. 8) | Project STAR data. Age: Not reported. Gender: Not reported. Grade: K-3. SES: Not reported. Ethnicity: Not reported | Age: 13 years old. Grade: 7 and 8. Gender: Not reported. Ethnicity specified through born in country (distribution on each country). SES specified through parent education (distribution on each country) (table 1, page 704, 705) |
| **Type of school (private, boarding, public)** | Not specified. | Public | Not reported |
| **Duration of class size reduction** | Not reported. | 4 years | Not reported |
| **Class size (divide into treated/comparison)** | Reading: 23.65 (SD: 4.7). Mathematics: 23.4 (SD: 4.88). Biology: 23.71 (SD: 4.19). Chemistry: 23.05 (SD: 4.92). Physics: 22,9448 (SD: 4,9023) (p. 8) | Small: 13-17; regular: 22-25; regular with full-time aide (22-25) | Average students per class specified on subject in each country varies |
| **Intensity (size of reduction)** | Not applicable. Comparison of existing class sizes | Not specified | Not specified |
| **Precision of class size measure (is it constant per subject/during the day)** | Class size measured per subject: Reading, Mathematics, Biology, Chemistry, Physics (p. 8) | Constant | Differs between subjects (mathematics, science) (number of students in each country, table 1, page 704, 705) |
| **Time period covered by analysis (divide into treatment/follow up)** | 2000 | Treatment: 1985-1989 | 1 year: 1994-1995 (treatment in 1994-1995, no follow up) |

| Study | Wößmann, 2003 | Wößmann, 2005a | Wößmann, 2005b |
| --- | --- | --- | --- |
| **Participant characteristics (age, gender, grade level, socio economic status, ethnicity)** | Data from TIMMS 1995. Students enrolled in the two adjacent grades, which contained the largest proportion of 13-year olds, which are the first two grades of secondary school. Each country drew a random sample of schools. Participant characteristics (SES, gender, age) for each country found in Table A2a. | Data from TIMMS 1995. Students enrolled in the two adjacent grades, which contained the largest proportion of 13-year olds, which are the first two grades of secondary school. Each country drew a random sample of schools (p. 333). Participant characteristics not reported. | Middle school students from 17 West European school systems (in 15 countries), 7th and 8th grade. P. 459. The average age of the tested students ranges from 13.1 years in Greece and Iceland to 14.3 years in Germany. P. 459 |
| **Type of school (private, boarding, public)** | Not specified. | Not specified. | Unclear |
| **Duration of class size reduction** | Not reported. | Not reported. | Not reported |
| **Class size (divide into treated/comparison)** | Class size (mean, SD) for each country found in table A2b. | Not reported in detail. "The smallest class sizes in the country sample are observed in Singapore with an average of about 33 students per class. In Korea and Thailand, average class sizes are as high as 50 students per class." (p. 334) | The lowest mean class size at around 20 students per class can be found in Denmark, the two Belgian systems and Switzerland. In Austria are the smallest classes in maths by far at 10.6 students on average. The largest average class sizes around 28 can be found in Greece and Spain. p.459-460 |
| **Intensity (size of reduction)** | Not applicable. Comparison of existing class sizes | Not applicable. Comparison of existing class sizes | Not reported |
| **Precision of class size measure (is it constant per subject/during the day)** | Both grade-average class size and class sizes for Mathematics and Science are reported (Table A2b) | Unclear. Within each school, generally one class was randomly chosen from each of the two grades and all of its students were tested. (p. 334). Class size measured in natural logarithm units (p. 341) | Not reported |
| **Time period covered by analysis (divide into treatment/follow up)** | 1995 | 1995 | Data is from TIMSS conducted in 1995 |

## Characteristics of excluded studies

| Study | Reason for exclusion |
| --- | --- |
| Agasisti et al., 2016 | Apparently the class size effect is only included at school level |
| Akabayashi & Nakamura, 2014 | No data at required level |
| Altinok & Kingdon , 2012 | No data at required level |
| Babcock & Betts, 2009 | Do not use class size as regressor but assume that transition from grade 3 to 4 captures variation in class size due to legislation |
| Baroody, 2017 | Class size included as covariate in an analysis on class format (departmentalized or self-contained) but coefficients not shown |
| Bernal, Mittag & Qureshi, 2016 | Do not use data at required level |
| Browning & Heinesen, 2007 | No data at required level |
| Cho, Glewwe & Whitler, 2012 | No data at required level |
| Choi, Moon & Ridder, 2017 | No data at required level |
| Chowa, Masa, Ramos & Ansong, 2015 | No data at required level |
| Corak & Lauzon, 2010 | No data at required level |
| Coupe, Olefir & Alonso, 2016 | No data at required level |
| Denny & Oppedisano, 2013 | No data on actual class size, it is estimated (see p. 10 in the Working Paper Denny & Oppedisano, 2010). |
| Dieterle, 2015 | Do have data at student level but analysis is on school level |
| Fabunmi, Brai-Abu & Adeyinka, 2007 | No data at required level |
| Ferrera, Cebada, Chaparro & Gonzalez, 2011 | Uses pupil-teacher-ratio (PTR). PISA data from Spain. Page 133 defines classroom size used, it is PTR and not actual class size |
| Fredriksson, Öckert & Oosterbeek, 2013 | Do not use data at required level (average class size in grades 4 to 6 in the district though only districts with one school). They do have actual class size |
| Fredriksson, Öckert & Oosterbeek, 2014 | Do not use data at required level (average class size in grades 4 to 6 in the district though only districts with one school). They do have actual class size |
| Funkhouser, 2009 | No data at the required level |
| Gary-Bobo & Mahjoub, 2013 | No relevant outcome |
| Grantham, 2000 | Before-after study design |
| Heinesen, 2009 | No data at required level (only number of students at school level) but restrict the sample to schools with no more than 20 students choosing French (the subject class under investigation) (and state that in only 6% of schools with grade enrolment of 20 are there more than one basic class) |
| Hoxby, 2000 | No data at the required level |
| Jaciw, 2016 | STAR data but only report results from other papers (Nye, Hedges, and Konstantopoulos’s (2000) table 10) |
| Jepsen & Rivkin, 2009 | No data at the required level |
| Jepsen & Rivkin, 2002 | No data at the required level |
| Jones, 2016 | Do not have actual class size for a large share of the students |
| Krassel & Heinesen, 2014 | No data at required level |
| Leuven & Løkken, 2017 | No data at required level |
| Leuven, Oosterbeek & Rønning, 2008 | No data at required level |
| Lubienski, Lubienski & Crane, 2008 | Do not use actual class size. Uses an estimate and the estimate is not at required level |
| Murdoch & Paul, 2002 | Participants not eligible (University students) |
| Musau & Migosi, 2013 | No data at required level |
| Sims, 2009 | No data at required level |
| Speas, 2002 | No data at required level |
| Speas, 2003 | No data at required level |
| Stecher & Bohrnstedt, 2000 | No data at required level. Summary of results from other reports |
| Tsai & Yang, 2015 | Do not analyse class size |

# Data appendices

## Data extraction

###

### Numeric data non-STAR studies

#### Studies used for analysis

| Author | Bressoux, 2009 | Ecalle, 2006 | Gerritsen, 2017 |
| --- | --- | --- | --- |
| **Type of outcome** | Continuous | Continuous | Continuous |
| **Outcome (there may be more than one, record them all)** | Reading and math | Reading and spelling | Math and reading |
| **Time Point (s) (record the exact time, there may be more than one, record them all)** | End of grade 3 | End of grade 1 (two time points March and June) | End of grade 2 |
| **Source (questionnaire, admin data, other(specify) or unclear)** | National tests | Novlex | Tests developed as part of the PRIMA project |
| **Valid Ns (only applicable for continuous outcome data). Mention treatment and comparison.** | 1680 students and 100 teachers/classes  In reading uses 1605/98 and math 1595/96 | T: 570 students, 100 classes; C: 622 students, 100 classes (what text says and first row in table 1) Second row in table 1 and table 2 (with results) says 622 T and 570 C? | 235 pairs of twins |
| **Method of estimation** | Linear regression with random class effect | None (as they only report significant results and CS NS) | Twin fixed-effect |
| **Statistics (risk ratio, odds ratio, standard error, 95 cf, DF, p-value, chi2)** | Coefficient and SE on class size, SD of tests and SD of class size | Means, SDs (separated by normal-aged/repeating a year) Normal-aged also available by early schooling, quarter of birth, first language and SES | Coefficient and SE on class size, SD of tests and SD of class size |
| **Page numbers and notes** | Table 1 and 4 p. 547 and 553 (novice teachers) | Table 2 and 4 (only normal aged students) | Table 1 and 3 p. 651 and 652 (2 grade twins) |
| **Level of aggregation** | Individual with class random effect | Not reported but probably individual as they (probably) do not have data for whole classes | Student. No clustering adjustment are necessary as they do not use whole classes |
| **Notes** | Coefficients times SD of 4 on CS and use SD of initial test scores (weighted for whole sample).Authors kindly provided number of students per class. | Lambert, E., & Chesnet, D. (2001). Novlex: une base de donne´es lexicales pour les e´le`ves de primaire [NovLex: a lexical database for French elementary-school readers]. L’Anne´e Psychologique, 101, 277–288. No clustering adjustment is necessary as they probably do not analyse whole classes | Coefficients times SD of 5 on CS, use SD on tests for the whole sample (PRIMA sample). |

| Author | Hudson, 2011 | Milesi, 2006 | Munoz, 2001 |
| --- | --- | --- | --- |
| **Type of outcome** | Continuous | Continuous | Continuous |
| **Outcome (there may be more than one, record them all)** | Math and Reading | Math and reading | Math, reading |
| **Time Point (s) (record the exact time, there may be more than one, record them all)** | Tenth grade | End of kindergarten | End of grade 3 |
| **Source (questionnaire, admin data, other(specify) or unclear)** | National tests | Item response theory (IRT) scaled scores | Scores in math and reading. Stanford Achievement Test (SAT) |
| **Valid Ns (only applicable for continuous outcome data). Mention treatment and comparison.** | Not reported (approximately 25,000 8th graders were surveyed but how many 10th graders is not reported) | Total (reading/math) 11567/12153 students and 2437/2556 classes. 442 teachers teach two classes | T: 47 in 4 schools; C: 57 students in same four schools. Number of classes not reported |
| **Method of estimation** | OLS and school random effects model (GLS) | Hierarchical linear model and teacher fixed effect | None |
| **Statistics (risk ratio, odds ratio, standard error, 95 cf, DF, p-value, chi2)** | Coefficient and t-statistic on class size, SD of tests and SD of class size. Use random effects model | Coefficient and SE: small vs large: regular vs large | Post-test mean (SD) and pre-test mean (SD) |
| **Page numbers and notes** | Table 1 and 3 page 17 and 23 and table 4 page 24 (use only the 12th grade math). | Table 1 and 6, p. 300 and 307, use model 2 | Table 2 and 5, p. 19 and 28 |
| **Level of aggregation** | Student. As they include school random effects no adjustment necessary | Two level model (student and class) | Individual. As not all in one class participate do not need correction for class clustering |
| **Notes** | Coefficients times SD of 6 and 7 on CS and use SD of final test scores. The coefficients on class size are positive (i.e. contrary to the expected, larger classes increase test scores) but in the text the researcher interprets these results as if class size reduction increases test scores. | Class size calculation, see p. 294 and note 6. Small classes less than 18, regular 18-23 and large classes more than 23. Use coefficient on small vs large, fall test score SD for all | Note the mean score of the treatment group decreases from pre to post in math (3.1 to 2.6) |

#### Studies not used for analysis/reason

| Author | Blatchford, 2003a | Blatchford, 2003b | Bosworth, 2014 |
| --- | --- | --- | --- |
| Notes | Collection of results from British Class Size Study. Cannot assess RoB as not enough information is provided. Only kindergarten ESs for reading reported, the rest reported as NS or a narrative description such as 'there was found to be an effect'. | No results reported other than graphs without CI. | Do not report SDs or number of observation (except class rooms but analysis is on individual level). Author was contacted February 2016 but did not provide the necessary data |

| Author | Clanet, 2010 | Dee, 2011 | Dieterle, 2013 |
| --- | --- | --- | --- |
| Notes | Only report the significance level and only report sign of the effects that are significant. Too little information provided to adequately assess risk of bias due to confounding | Subject specific test score, may be math, reading, science or history but not specified. First difference between subjects is outcome | Only have data at required level for two of three grades and do not provide useable separate results |

| Author | Maier, 1997 | Molnar, 1998 | Molnar, 1999a |
| --- | --- | --- | --- |
| Notes | A Regular classroom refers to a classroom with one teacher. Most regular classrooms have 15 or fewer students, but a few exceed 15. A 2-Teacher Team classroom is a class where two teachers work collaboratively to teach as many as 30 students. A Shared-Space classroom is a classroom that has been fitted with a temporary wall that creates two teaching spaces, each with one teacher and about 15 students. A Floating Teacher classroom is a room consisting of one teacher and about 30 students, except during reading, language arts, and mathematics instruction when another teacher joins the class to reduce the ratio to 15:1. Only analyse effect of type of classroom within SAGE schools. | See Maier, 1997 | See Maier, 1997 |

| Author | Molnar, 1999b | Molnar, 2001 | Murdoch, 1986 |
| --- | --- | --- | --- |
| Notes | See Maier, 1997 | See Maier, 1997 | Only report p values from a multivariate model (8 outcomes) with CS, age, gender and school, separated by grade |

| Author | Maasoumi, 2005 | NICHD, 2004 | Otsu, 2015 |
| --- | --- | --- | --- |
| Notes | Do not provide method/results we can use (first or second order stochastic dominance tests) | Not possible to extract SDs. Multiple regression (they report they use standard hierarchical multiple regression but no hierarchy is presented), report both linear and discontinuous piecewise linear regression. Report coefficients with only significance level (marked by stars) | Relevant results are presented graphically and no ES and SE can be extracted. |

| Author | Sanogo, 1994 | Shapson, 1980 | Wiermann, 2005 |
| --- | --- | --- | --- |
| Notes | Reproduction of STAR and Indiana PRIME Time results (Word et al. 1990 and Tillitsky, Gilman, Mohr, and Stone, 1988) | Not possible to extract ES. Unadjusted mean score (not separate for the two years) and residual mean score. They do not report outcomes for all groups for all years, so we cannot determine the effect of being randomised to one of the four arms. | Difference between math and physics test scores (the chemistry/biology and the reading/biology differences scores 5) |

#### Studies not used for analysis/too high risk of bias

| Author | Achilles, 1995 | Akerhielm, 1995 | Angrist, 1999 |
| --- | --- | --- | --- |
| Notes | Judged 5 on the confounding item | Judged 5 on the confounding item | Judged 5 on the confounding item |

| Author | Angrist, 2014 | Annevelink, 2004 | Blatchford, 2002 |
| --- | --- | --- | --- |
| Notes | Judged 5 on the confounding item | Judged 5 on the confounding item | Judged 5 on the selective reporting item |

| Author | Bonesrønning, 2003 | Boozer, 1995 | Boozer, 2001a |
| --- | --- | --- | --- |
| Notes | Judged 5 on the confounding item | Judged 5 on the confounding item | Judged 5 on the confounding item |

| Author | Boozer, 2001b | Borland, 2005 | Breton, 2012 |
| --- | --- | --- | --- |
| Notes | Judged 5 on the confounding item | Judged 5 on the confounding item | Judged 5 on the confounding item |

| Author | Burde, 1990 | Carpenter, 2003 | Chargois, 2008 |
| --- | --- | --- | --- |
| Notes | Judged 5 on the confounding item | Judged 5 on the selective reporting item | Judged 5 on the confounding item |

| Author | Costello, 1992 | Dennis, 1986 | Dharmadasa, 1995 |
| --- | --- | --- | --- |
| Notes | Judged 5 on the confounding item | Judged 5 on the other risk of bias item | Judged 5 on the confounding item |

| Author | Dobbelsteen, 2002 | Galton, 2012 | Gilman, 1988a |
| --- | --- | --- | --- |
| Notes | Judged 5 on the confounding item | Judged 5 on the confounding item | Judged 5 on the confounding item |

| Author | Gilman, 1988b | Haenn, 2002 | Hallinan, 1985 |
| --- | --- | --- | --- |
| Notes | Judged 5 on the confounding item | Judged 5 on the confounding item | Judged 5 on the confounding item |

‘

| Author | Hirschfeld, 2016 | Hojo, 2011 | Hojo, 2013 |
| --- | --- | --- | --- |
| Notes | Judged 5 on the confounding item | Judged 5 on the other bias item | Judged 5 on the confounding item |

| Author | Iacovou, 2002 | Iversen, 2013 | Jakubowski, 2006 |
| --- | --- | --- | --- |
| Notes | Judged 5 on the confounding item | Judged 5 on the confounding item | Judged 5 on the confounding item |

| Author | Konstantopoulos, 2014 | Konstantopoulos, 2016a | Konstantopoulos, 2016b |
| --- | --- | --- | --- |
| Notes | Judged 5 on the confounding item | Judged 5 on the confounding item | Judged 5 on the confounding item |

| Author | Krueger, 2002 | Lavy, 2001 | Levin, 2001 |
| --- | --- | --- | --- |
| Notes | Judged 5 on the confounding item | Judged 5 on the confounding item | Judged 5 on the confounding item |

| Author | Li, 2015 | Li, 2017 | Lindahl, 2005 |
| --- | --- | --- | --- |
| Notes | Judged 5 on the confounding item | Judged 5 on the confounding item | Judged 5 on the confounding item |

| Author | Ma, 2006 | Maples, 2009 | McGiverin, 1989 |
| --- | --- | --- | --- |
| Notes | Judged 5 on the confounding item | Judged 5 on the confounding item | Judged 5 on the confounding item |

| Author | Merritt, 2011 | Moshoeshoe, 2015 | Nandrup, 2016 |
| --- | --- | --- | --- |
| Notes | Judged 5 on the other bias item | Judged 5 on the confounding item | Judged 5 on the confounding item |

| Author | Pollard, 1995 | Pong, 2001 | Tienken, 2009 |
| --- | --- | --- | --- |
| Notes | Judged 5 on the confounding item | Judged 5 on the confounding item | Judged 5 on the confounding item |

| Author | Tillitsky, 1988 | Uhrain, 2016 | Urquiola, 2006 |
| --- | --- | --- | --- |
| Notes | Judged 5 on the confounding item | Judged 5 on the confounding item | Judged 5 on the other bias item |

| Author | Watson, 2016 | Wenfan, 2005 | West, 2006 |
| --- | --- | --- | --- |
| Notes | Judged 5 on the confounding item | Judged 5 on the confounding item | Judged 5 on the confounding item |

| Author | Wößmann, 2006 | Wößmann, 2003 | Wößmann, 2005a | Wößmann, 2005b |
| --- | --- | --- | --- | --- |
| Notes | Judged 5 on the confounding item | Judged 5 on the confounding item | Judged 5 on the confounding item | Judged 5 on the confounding item |

### Numeric data STAR studies

| Author | Achilles, 1993a | Achilles, 1993b | Balestra, 2014 |
| --- | --- | --- | --- |
| Type of outcome |  | Continuous | Continuous |
| Outcome (there may be more than one, record them all) |  | Math and reading | Math and reading |
| Time Point (s) (record the exact time, there may be more than one, record them all) |  | End of grade 4 and 5 | End of year kindergarten and 1 grade, and 4/8 grade |
| Source (questionnaire, admin data, other(specify) or unclear) |  | Scores in math and reading. The Tennessee Comprehensive Assessment Program (TCAP) | Scores in math and reading. Stanford Achievement Test (SAT) for KG and 1 grade and Technical reports and Database User's Guide report that the Tennessee Comprehensive Assessment Program (TCAP, Comprehensive Tests of Basic Skills (CTBS/McGraw Hill, 1989) and Basic Skills First (BSF) criterion-referenced tests) was used for the Lasting Benefits Study (grade 4-8) |
| Valid Ns (only applicable for continuous outcome data). Mention treatment and comparison. |  | Report that the sample contained 4243/4649 students in grade 4/5 and according to the Database User's Guide (Finn et al., 2007) there are 6339/2593 students in 4/5 grade with achievement data and at least one year in STAR. | Use 'whole sample', numbers reported are 5837/6449 for Kindergarten/1 grade but not separately reported for small class and regular with aide and quantiles. From the Technical report (Word, 1994): Kindergarten number of classes/students: small classes 127/1678, regular classes: 103/2049, regular with aide classes 98/2007; total 328/5734 and 79 schools; 1. grade number of classes: small classes 122, regular classes 111, regular with aide classes 98; total 331 classes and total 76 schools and only total number of students reported (6572). For 4/8 grade total numbers reported are 4043/5056 |
| Method of estimation |  | None | Unconditional quantile regression using scaled test scores (0-100) separate for math/reading for KG/1 grade and OLS (?) for 4/8 grade |
| Statistics (risk ratio, odds ratio, standard error, 95 cf, DF, p-value, chi2) |  | Effect sizes | NOTE separate regression for small vs regular and regular with aide vs regular. Coefficient (interpreted as percentage points higher score) with robust SE's |
| Page numbers and notes |  | Table 5 | Table 3, 4 (divided into subsamples of black, free lunch and boys) and 5 |
| Level of aggregation |  | Probably student | Student and school fixed effects |
| Notes | STAR. Reproduction of the results in Word et al. 1990 and further results on various subgroups (for example entering STAR in grade 1 or results on retained/not retained etc.) | LBS analysis. Grade 5 results judged 5 in the incomplete outcome data item. Separate results for S vs R and R vs RA | STAR and LBS reanalysis. Graduated from high school is also available |

| Author | Bingham, 1994 | Chetty, 2011 | Ding, 2005 |
| --- | --- | --- | --- |
| Notes | STAR reanalysis. No useful data provided (only means) | STAR no useful outcomes provided. Test score as the average math and reading percentile rank score attained in the student’s year of entry into the experiment is only relevant outcome reported for this review. | STAR reanalysis. None of the analyses can be used for this review. Analyses the effect of each class size in the range 12-28 relative to 22. Further report results from regressions where class size is interacted with several covariates. |

| Author | Ding, 2010 | Ding, 2011 | Doulgas, 1989 |
| --- | --- | --- | --- |
| Type of outcome | Continuous | Continuous |  |
| Outcome (there may be more than one, record them all) | Reading and math | Reading and math |  |
| Time Point (s) (record the exact time, there may be more than one, record them all) | End of grade KG, 1, 2 and 3 | End of KG |  |
| Source (questionnaire, admin data, other(specify) or unclear) | Scores in math and reading. Stanford Achievement Test (SAT) | Scores in math and reading. Stanford Achievement Test (SAT) |  |
| Valid Ns (only applicable for continuous outcome data). Mention treatment and comparison. | Use only those who started in KG. Small class 1900 and regular (with aid too) 4425 | Use only those who started in KG. Small class 1900 and regular (with aid too) 4425. Have reading test score on a total of 5849 students and math score on a total of 5871 students |  |
| Method of estimation | Structural equation model. Effects of number of years (and sequence) treated | Regression with small class interacted with covariates |  |
| Statistics (risk ratio, odds ratio, standard error, 95 cf, DF, p-value, chi2) | Coefficients and SEs (corrected) | Coefficients and SEs (corrected) |  |
| Page numbers and notes | Table 3 and 4 p. 38 and 39 | Table 3 and 4 |  |
| Level of aggregation | Student | Student |  |
| Notes | STAR reanalysis | STAR reanalysis. Uses KG data only. Do not separate R and RA | STAR re analysis; do not provide results we can use (percent of variance accounted for by class size, effect on highest and lowest achieving student in each class and separate regressions for small/regular classes). |

| Author | Finn, 1989 | Finn, 1990a | Finn, 1990b |
| --- | --- | --- | --- |
| Type of outcome | Continuous | Continuous |  |
| Outcome (there may be more than one, record them all) | Math and reading | Math and reading |  |
| Time Point (s) (record the exact time, there may be more than one, record them all) | End of grade 4 | End of grade 1 |  |
| Source (questionnaire, admin data, other(specify) or unclear) | Tennessee Comprehensive Assessment Program (TCAP, Comprehensive Tests of Basic Skills (CTBS/McGraw Hill, 1989) and Basic Skills First (BSF) criterion-referenced tests) | Scores in math and reading. Stanford Achievement Test (SAT) and percent passed on Basic Skills First (BSF) |  |
| Valid Ns (only applicable for continuous outcome data). Mention treatment and comparison. | 2207 students | 76 schools, 122 treated classes, 111 regular and 98 regular with aide. Total 6570 students |  |
| Method of estimation | Least squares with no covariates | None we can use |  |
| Statistics (risk ratio, odds ratio, standard error, 95 cf, DF, p-value, chi2) | Effect sizes | Means, SD (both at student and class level though not for the white/minority division), SMD for all three arms. Further both total and race separated |  |
| Page numbers and notes | Table 3 | Table 3, 4, 5 and 6 |  |
| Level of aggregation | Student | Students and teachers randomised and both individual and class is the level of analysis |  |
| Notes | Small classes in 3. Grade compared to regular classes in 3. grade | Results divided on location (inner-city, rural etc.) also provided. A growth analysis of students participating in the same classroom arrangement for both years and who had complete data (35%) performed but is given 5 on incomplete data | STAR reanalysis for those in same class arrangement for 3 years (K-2. grade). Judged 5 in the incomplete outcome data item |

| Author | Finn, 1998 | Finn, 1999 | Finn, 2001 |
| --- | --- | --- | --- |
| Type of outcome | Continuous |  | Continuous |
| Outcome (there may be more than one, record them all) | Math and reading |  | Math, reading and science (only grade 4, 6 and 8) |
| Time Point (s) (record the exact time, there may be more than one, record them all) | End of grade KG,1, 2 and 3 |  | End of KG and grade 1, 2, 3, 4, 6 and 8 |
| Source (questionnaire, admin data, other(specify) or unclear) | Scores in math and reading. Stanford Achievement Test (SAT) and percent passed on Basic Skills First (BSF) |  | Stanford Achievement Tests for KG-3. grade and Comprehensive Tests of Basic Skills (CTBS/McGraw Hill, 1989) and Basic Skills First (BSF) for grade 4, 6 and 8 |
| Valid Ns (only applicable for continuous outcome data). Mention treatment and comparison. | Numbers used not reported but from the Technical report (Word, 1994): Kindergarten number of classes/students: small classes 127/1678, regular classes: 103/2049, regular with aide classes 98/2007; total 328/5734 and 79 schools; 1. grade number of classes: small classes 122, regular classes 111, regular with aide classes 98; total 331 classes and total 76 schools and only total number of students reported (6572); 2. grade number of classes: small classes 109, regular classes 79, regular with aide classes 85; total 273 classes and total 62 schools and only total number of students reported (5328) (reduction due to removal of 67 teachers who received Project Star training); 3. grade number of classes: small classes 110, regular classes 68, regular with aide classes 85; total 263 classes and total 60 schools and only total number of students reported (4744) (two schools had incomplete test data and was removed) |  | Numbers used for analysis not reported, only sample sizes (these are different from the numbers reported in the Technical report (Word, 1994) (quite larger for 2. and 3. grade): Kindergarten number of classes: small classes 127, regular classes: 99, regular with aide classes 99; total number of students 6325 and 79 schools; 1. grade number of classes: small classes 123, regular classes 115, regular with aide classes 100; total 76 schools and total number of students 6829; 2. grade number of classes: small classes 133, regular classes 100, regular with aide classes 107; total 75 schools and total number of students 6840 (apparently do not remove the 67 teachers who received Project Star training); 3. grade number of classes: small classes 140, regular classes 90, regular with aide classes 108; total 75 schools and total number of students 6802 (two schools had incomplete test data but is apparently not removed). Total number of students (CTBS/BSF tests): 4. grade: 4015/4045; 6. grade: 6100/2737; 8. Grade: 5835/5217 |
| Method of estimation | None |  | None |
| Statistics (risk ratio, odds ratio, standard error, 95 cf, DF, p-value, chi2) | Effect sizes (S vs R + RA) |  | Effect sizes (probably S vs R) |
| Page numbers and notes | Table 1 |  | Table 2 and 4 |
| Level of aggregation | Student |  | Student |
| Notes | Results also provided separated by white/minority | Reporting of ES from other studies (using STAR), calculate Grade Equivalence ESs (not an outcome of this review) and behaviour ESs | STAR and LBS reanalysis. A three level HLM model (student, classes and location of school) with interaction terms also available |

| Author | Finn, 2005 | Folger, 1989 | Hanushek, 1999 |
| --- | --- | --- | --- |
| Type of outcome |  | Continuous | Continuous |
| Outcome (there may be more than one, record them all) |  | Math and reading | Reading and math |
| Time Point (s) (record the exact time, there may be more than one, record them all) |  | End of each grade (K, 1, 2 and 3) | End of grade KG, 1, 2 and 3 |
| Source (questionnaire, admin data, other(specify) or unclear) |  | Not reported specifically but probably total SAT | Scores in math and reading. Stanford Achievement Test (SAT) |
| Valid Ns (only applicable for continuous outcome data). Mention treatment and comparison. |  | Out-of-range classes excluded from original data (not reported what that is) Only total number of students reported: K: 5126, 1: 5541, 2: 5494 and 3: 5242 | Small classes read (math)/regular + regular with aide read (math): Kindergarten: 1739 (1762)/4049 (4108); 1 grade: 1823 (1867)/4572 (4731); 2 grade: 1794 (1789)/4283 (4276); 3 grade: 1915 (1938)/4085 (4139) |
| Method of estimation |  | None | z-score means |
| Statistics (risk ratio, odds ratio, standard error, 95 cf, DF, p-value, chi2) |  | SMDs by grade. | z-score means |
| Page numbers and notes |  | Figure 1 | Table 5 p. 156 |
| Level of aggregation |  | Class | Student |
| Notes | STAR data, analysis of high school graduation. Judged 5 in the other bias item | Also results for new students enrolled (each year) and high/low SES (not defined what that is) and total (during the 4 years) retention available |  |

| Author | Harvey, 1994 | Jackson, 2013 | Jacobs, 1987 |
| --- | --- | --- | --- |
| Type of outcome | Continuous | Continuous |  |
| Outcome (there may be more than one, record them all) | Math and reading | Math and reading composite score |  |
| Time Point (s) (record the exact time, there may be more than one, record them all) | For retained kindergartners: End of year kindergarten, 1, 2 and 3 grades; for retained 1. graders: End of year 1,2 and 3 grade | End of year kindergarten and 1 grade |  |
| Source (questionnaire, admin data, other(specify) or unclear) | Scores in math and reading. Stanford Achievement Test (SAT) | Scores in math and reading. Stanford Achievement Test (SAT) |  |
| Valid Ns (only applicable for continuous outcome data). Mention treatment and comparison. | Retained kindergartners (read/math): Small 59/61, regular 93/93 and regular with aide 76/77 (thereafter varies by time point); retained 1. Graders: Small 146/153, regular 472/505 and regular with aide 405/438 (thereafter varies by time point). | Use 'whole sample', numbers reported are 5812/6410 for Kindergarten/1 grade but not separately reported for small class and regular with aide and quantiles. From the Technical report (Word, 1994): Kindergarten number of classes/students: small classes 127/1678, regular classes: 103/2049, regular with aide classes 98/2007; total 328/5734 and 79 schools; 1. grade number of classes: small classes 122, regular classes 111, regular with aide classes 98; total 331 classes and total 76 schools and only total number of students reported (6572). |  |
| Method of estimation | ANOVA | OLS regression using standardised composite (reading/math) test scores (z-score with mean zero and SD 1 for each year) separate for KG/1 grade including covariates and school fixed effects (without also available) and quantile regression (results only shown in figures) |  |
| Statistics (risk ratio, odds ratio, standard error, 95 cf, DF, p-value, chi2) | Means, F-value and p-value from ANOVA | Coefficients and probably SE's |  |
| Page numbers and notes | Table 5 and 7 p. 16 and 18 | Table 1 |  |
| Level of aggregation | Unclear, but probably individual | Student and with (also without) school fixed effects |  |
| Notes | STAR data, only retainees used (reanalysis). Judged 5 in the other bias item | STAR reanalysis KG and 1 grade | No useful data: Results in table 3, 4 and 5 (for three different outcomes) have main effect for class type (not small separated out). Cross tabulation of the 3 outcomes in table 6, 7 and 8 but only raw totals and percent scoring low/middle/high and other tables subdivided on several covariates. Scores for small class size are given in fig. 20&38, but no std. dev. |

| Author | Konstantopoulos, 2008 | Konstantopoulos, 2009 | Konstantopoulos, 2011 |
| --- | --- | --- | --- |
| Type of outcome | Continuous | Continuous | Continuous |
| Outcome (there may be more than one, record them all) | Reading and math | Math, reading, science | Math and reading |
| Time Point (s) (record the exact time, there may be more than one, record them all) | End of year kindergarten, 1,2 and 3 grade | Grade 4, 5, 6, 7, 8 | End of grade K to 3 |
| Source (questionnaire, admin data, other(specify) or unclear) | Scores in math and reading. Stanford Achievement Test (SAT) | Stanford Achievement test is what they write but Technical reports and Database User's Guide report that the Tennessee Comprehensive Assessment Program (TCAP, Comprehensive Tests of Basic Skills (CTBS/McGraw Hill, 1989) and Basic Skills First (BSF) criterion-referenced tests) was used for the Lasting Benefits Study (grade 4-8) | Stanford Achievement test |
| Valid Ns (only applicable for continuous outcome data). Mention treatment and comparison. | Numbers used not reported but from the Technical report (Word, 1994): Kindergarten number of classes/students: small classes 127/1678, regular classes: 103/2049, regular with aide classes 98/2007; total 328/5734 and 79 schools; 1. grade number of classes: small classes 122, regular classes 111, regular with aide classes 98; total 331 classes and total 76 schools and only total number of students reported (6572); 2. grade number of classes: small classes 109, regular classes 79, regular with aide classes 85; total 273 classes and total 62 schools and only total number of students reported (5328) (reduction due to removal of 67 teachers who received Project Star training); 3. grade number of classes: small classes 110, regular classes 68, regular with aide classes 85; total 263 classes and total 60 schools and only total number of students reported (4744) (two schools had incomplete test data and was removed) | Only totals for each grade (4-8) is reported: 4305/2505/3450/3896/4411 for grade 4/5/6/7/8 | Use 'whole sample', numbers not reported but from the Technical report (Word, 1994): Kindergarten number of classes/students: small classes 127/1678, regular classes: 103/2049, regular with aide classes 98/2007; total 328/5734 and 79 schools; 1. grade number of classes: small classes 122, regular classes 111, regular with aide classes 98; total 331 classes and total 76 schools and only total number of students reported (6572); 2. grade number of classes: small classes 109, regular classes 79, regular with aide classes 85; total 273 classes and total 62 schools and only total number of students reported (5328) (reduction due to removal of 67 teachers who received Project Star training); 3. grade number of classes: small classes 110, regular classes 68, regular with aide classes 85; total 263 classes and total 60 schools and only total number of students reported (4744) (two schools had incomplete test data and was removed) |
| Method of estimation | Quantile regression with covariates (gender, ethnicity and SES). Whether achievement distribution used is taken over T/C or T+C not reported | Quantile regression on math, reading and science test scores separate for each grade (4-8). Comparing small class (in 3rd grade) effect for different quantiles. | Each school treated as an individual RCT - ES from linear regression (with small class and regular with aide in same model) - overall mean calculated by inverse variance weighted random effects model |
| Statistics (risk ratio, odds ratio, standard error, 95 cf, DF, p-value, chi2) | ESs with stars indicating significance level. |  | Weighted mean SMD (SE and variance (tau squared probably) and SE of variance) |
| Page numbers and notes | Table 5 | Table 1 | Table 3. In the working paper (Konstantopoulos, 2009) results differ slightly because individual gender, race and SES is controlled for. In addition a mixed effect meta regression that included several observed school characteristics as predictors is performed in the working paper, but results only reported as percentage of the variance explained. |
| Level of aggregation | Not reported but probably it is individual | Individual | Student and school |
| Notes | STAR reanalysis | Reanalyses STAR and LBS. ITT and IV analyses (same quantile regression effect of 3rd grade treatment in 4-8 grade separately), also available, and a dose analysis (do not use as it score 5 in other bias). Unclear what their achievement distribution is. | Reanalysis of STAR |

| Author | Krueger, 1999 | Krueger, 2001a | Krueger, 2001b |
| --- | --- | --- | --- |
| Type of outcome | Continuous | Dichotomous |  |
| Outcome (there may be more than one, record them all) | Average of Math, reading and word | College entrance exam (ACT and SAT) |  |
| Time Point (s) (record the exact time, there may be more than one, record them all) | End of year kindergarten, 1,2 and 3 grade | 1998 with treatment in 1985-1988 |  |
| Source (questionnaire, admin data, other(specify) or unclear) | Average percentile scores in math, reading and word (not shown separately). Stanford Achievement Test (SAT) | College entrance exam (ACT and SAT) |  |
| Valid Ns (only applicable for continuous outcome data). Mention treatment and comparison. | Use 'whole sample', numbers not reported but from the Technical report (Word, 1994): Kindergarten number of classes/students: small classes 127/1678, regular classes: 103/2049, regular with aide classes 98/2007; total 328/5734 and 79 schools; 1. grade number of classes: small classes 122, regular classes 111, regular with aide classes 98; total 331 classes and total 76 schools and only total number of students reported (6572); 2. grade number of classes: small classes 109, regular classes 79, regular with aide classes 85; total 273 classes and total 62 schools and only total number of students reported (5328) (reduction due to removal of 67 teachers who received Project Star training); 3. grade number of classes: small classes 110, regular classes 68, regular with aide classes 85; total 263 classes and total 60 schools and only total number of students reported (4744) (two schools had incomplete test data and was removed) | Use two samples: all STAR students 11294 and the subsample that were not behind grade 9117. |  |
| Method of estimation | OLS with (and without) school fixed effects. A 2SLS with random assignment as instrument and a model with imputed outcomes | Logit estimation with covariates and school/school-by-wave/none fixed effects |  |
| Statistics (risk ratio, odds ratio, standard error, 95 cf, DF, p-value, chi2) | Two analyses (separate models for treatment as received and for treatment as assigned) each comparing small to regular and regular with aide to regular. Analysis separate for each grade but average achievement including gender, minority and SES as well as teacher ethnicity, experience and education, with and without school fixed effects. Coefficient estimates and robust SE's. An analysis with actual treatment and imputed values for missing data (most recent test percentile). A 2SLS with random assignment as instrument model (and a model further divided by entry grade and current grade. A model of cumulative effects (total and separate by gender, SES, minority and location and one separate for the three tests) | Coefficients and SE's and marginal effects also provided. |  |
| Page numbers and notes | Table 5-11 | Table 4 (not behind grade), 5 (all) and 6 (separate ACT and SAT for those not behind grade). |  |
| Level of aggregation | Two level analysis (student and school) and three level (include class) | Student and school (and entry grade) fixed effects |  |
| Notes | Reanalysis of STAR | STAR follow up | Same as Krueger (2001a) with updated data, but report only weighted averages of percentages and do not report the numbers used for analysis, so no useable results |

| Author | Mckee, 2010 | McKee, 2015 | Mosteller, 1995 |
| --- | --- | --- | --- |
| Type of outcome | Continuous |  |  |
| Outcome (there may be more than one, record them all) | Math and Reading |  |  |
| Time Point (s) (record the exact time, there may be more than one, record them all) | End of KG |  |  |
| Source (questionnaire, admin data, other(specify) or unclear) | Stanford Achievement Tests |  |  |
| Valid Ns (only applicable for continuous outcome data). Mention treatment and comparison. | 1754 treated and 4089 control |  |  |
| Method of estimation | OLS with and without school FE |  |  |
| Statistics (risk ratio, odds ratio, standard error, 95 cf, DF, p-value, chi2) | Coefficient (SE) |  |  |
| Page numbers and notes | Table 2 |  |  |
| Level of aggregation | Student |  |  |
| Notes | STAR reanalysis. Only KG and merge R and RA. OLS w/wo school FE controlling for teachers with fewer than three years of experience and teachers with an advanced degree, and for the student's race-ethnicity, gender, age, special education status, whether or not they are repeating kindergarten, attendance record, and subsidized lunch eligibility. Specifications that do not include school fixed effects also include indicators for community type (suburban, rural, urban, and inner-city). | STAR reanalysis. Use only KG and pool R and RA classes and transform test scores to have zero mean and SD of one and include covariates | STAR Provides results from other articles: Finn, J.D., and Achilles, C.M. Answers and questions about class size: A state-wide experiment. American Educational Research Journal (1990) 27, 3:557–77, Table 5. And Word, E., Johnston, J., Bain, H.P., et al. Student/Teacher Achievement Ratio (STAR): Tennessee’s K-3 class size study, Nashville: Tennessee Department of Education, Figures 1 and 2. |

| Author | Nye, 1992 | Nye, 1993 | Nye, 1992/1994 |
| --- | --- | --- | --- |
| Type of outcome |  |  | Continuous |
| Outcome (there may be more than one, record them all) |  |  | Math, reading and science (only grade 4 and 5) |
| Time Point (s) (record the exact time, there may be more than one, record them all) |  |  | End of grade KG-5 |
| Source (questionnaire, admin data, other(specify) or unclear) |  |  | Scores in math and reading. Stanford Achievement Test (SAT) for grade KG-3 and percent passed on Basic Skills First (BSF) for grade 1-3 and the Tennessee Comprehensive Assessment Program (TCAP, Comprehensive Tests of Basic Skills (CTBS/McGraw Hill, 1989) and Basic Skills First (BSF) criterion-referenced tests) used for the Lasting Benefits Study (grade 4-5) |
| Valid Ns (only applicable for continuous outcome data). Mention treatment and comparison. |  |  | The numbers per grade used is 5734 in KG and 5905 in 1. Grade). Report that the sample contained 4320 students through grade 4 and 5 |
| Method of estimation |  |  | None |
| Statistics (risk ratio, odds ratio, standard error, 95 cf, DF, p-value, chi2) |  |  | Effect sizes (S vs R + RA) for grade KG-3 and S vs R and R vs RA for grade 4 and 5 |
| Page numbers and notes |  |  | Table 6 and 10 |
| Level of aggregation |  |  | Student |
| Notes | LBS technical report. Is judged 5 in the incomplete outcome data item | Same analyses and results as provided in Nye, 1992/1994 | Divided on white/minority also available for grade KG-3. BSF passing in grade 1-3 also available. Note the analysis for grade 4 and 5 are judged 5 in the other risk of bias item |

| Author | Nye, 2000a | Nye, 2000b | Nye, 2001a |
| --- | --- | --- | --- |
| Type of outcome | Continuous | Continuous |  |
| Outcome (there may be more than one, record them all) | Math and reading | Math and reading |  |
| Time Point (s) (record the exact time, there may be more than one, record them all) | End of year kindergarten, 1,2 and 3 grade | End of year kindergarten, 1,2 and 3 grade |  |
| Source (questionnaire, admin data, other(specify) or unclear) | Scores in math and reading. Stanford Achievement Test (SAT) | Scores in math and reading. Stanford Achievement Test (SAT) |  |
| Valid Ns (only applicable for continuous outcome data). Mention treatment and comparison. | Numbers used not reported but from the Technical report (Word, 1994): Kindergarten number of classes/students: small classes 127/1678, regular classes: 103/2049, regular with aide classes 98/2007; total 328/5734 and 79 schools; 1. grade number of classes: small classes 122, regular classes 111, regular with aide classes 98; total 331 classes and total 76 schools and only total number of students reported (6572); 2. grade number of classes: small classes 109, regular classes 79, regular with aide classes 85; total 273 classes and total 62 schools and only total number of students reported (5328) (reduction due to removal of 67 teachers who received Project Star training); 3. grade number of classes: small classes 110, regular classes 68, regular with aide classes 85; total 263 classes and total 60 schools and only total number of students reported (4744) (two schools had incomplete test data and was removed) | Numbers used not reported but from the Technical report (Word, 1994): Kindergarten number of classes/students: small classes 127/1678, regular classes: 103/2049, regular with aide classes 98/2007; total 328/5734 and 79 schools; 1. grade number of classes: small classes 122, regular classes 111, regular with aide classes 98; total 331 classes and total 76 schools and only total number of students reported (6572); 2. grade number of classes: small classes 109, regular classes 79, regular with aide classes 85; total 273 classes and total 62 schools and only total number of students reported (5328) (reduction due to removal of 67 teachers who received Project Star training); 3. grade number of classes: small classes 110, regular classes 68, regular with aide classes 85; total 263 classes and total 60 schools and only total number of students reported (4744) (two schools had incomplete test data and was removed) |  |
| Method of estimation | Hierarchical linear regression and 'raw' ES's also available | Hierarchical linear regression |  |
| Statistics (risk ratio, odds ratio, standard error, 95 cf, DF, p-value, chi2) | Analysis separate for each grade and reading and math including gender, SES and minority status, interaction of small class and gender, SES and minority respectively and (three way) interaction of small class, gender and minority and a similar analysis with three way interaction: small class, gender and SES. Coefficient estimates with stars. Cannot be used. Also available are effect sizes (d's) separated by white/minority and high/low SES and ES's by gender within race (white/minority) and SES (high/low) (no regression). | Three analyses (two separate models for treatment as received (a two level and a three level model) and a three level model for treatment as assigned) each comparing regular to small and (for the two level model only) regular with aide (although their writing up of the model does not include regular with aide as a variable and it only appears in the two level model not in any of the three level models as R and RA are assumed to be the same in order to be able to calculate variances for schools with only three classes)) Analysis separate for each grade and reading and math including gender and SES, interaction of small class and gender (although coefficients shown report they are for gender and minority interaction?), geographic location of school, teacher experience, school SES and school minority. Coefficient estimates with stars. |  |
| Page numbers and notes | Table 2-7 | Table 8-10 |  |
| Level of aggregation | Two level analysis (student and school) | Two level analysis (student and school) and three level (include class) |  |
| Notes | Table 2, 3, 5 and 6 provide effect sizes (d's) separated by white/minority and high/low SES and ES's by gender within race (white/minority) and SES (high/low) (no regression) (no regression). Interact treatment with several variables. A repeated measures analysis of the data is also available | ITT analysis and treatment as received analysis available. Interact treatment with gender (possibly, this is what the writing up of the model and the interpretation in the text says, the coefficient labels state it is a female and black interaction | STAR. Two analyses: 1) Students who participated at least 1 year and was part of the trial in 3. Grade 2) students participating all 4 years. Judged 5 in the incomplete outcome data item |

| Author | Nye, 2001b | Nye, 2002 | Prais, 1996 |
| --- | --- | --- | --- |
| Type of outcome | Continuous | Continuous | Continuous |
| Outcome (there may be more than one, record them all) | Reading and math | Reading and math | Math and reading |
| Time Point (s) (record the exact time, there may be more than one, record them all) | End of grade 1, 2 and 3 | End of grade 1, 2 and 3 | End of year kindergarten, 1,2 and 3 grade and value added (per year and 3 years average) |
| Source (questionnaire, admin data, other(specify) or unclear) | Scores in math and reading. Stanford Achievement Test (SAT) |  | Scores in math and reading. Stanford Achievement Test (SAT) |
| Valid Ns (only applicable for continuous outcome data). Mention treatment and comparison. | Small class in all grades (K-1): 1192; Small class in some (K only possible) or no grades: 3020 | Not reported but: from the Technical report (Word, 1994): Kindergarten number of classes/students: small classes 127/1678, regular classes: 103/2049, regular with aide classes 98/2007; total 328/5734 and 79 schools; 1. grade number of classes: small classes 122, regular classes 111, regular with aide classes 98; total 331 classes and total 76 schools and only total number of students reported (6572); 2. grade number of classes: small classes 109, regular classes 79, regular with aide classes 85; total 273 classes and total 62 schools and only total number of students reported (5328) (reduction due to removal of 67 teachers who received Project Star training); 3. grade number of classes: small classes 110, regular classes 68, regular with aide classes 85; total 263 classes and total 60 schools and only total number of students reported (4744) (two schools had incomplete test data and was removed) | Use 'whole sample', numbers not reported but from the Technical report (Word, 1994): Kindergarten number of classes/students: small classes 127/1678, regular classes: 103/2049, regular with aide classes 98/2007; total 328/5734 and 79 schools; 1. grade number of classes: small classes 122, regular classes 111, regular with aide classes 98; total 331 classes and total 76 schools and only total number of students reported (6572); 2. grade number of classes: small classes 109, regular classes 79, regular with aide classes 85; total 273 classes and total 62 schools and only total number of students reported (5328) (reduction due to removal of 67 teachers who received Project Star training); 3. grade number of classes: small classes 110, regular classes 68, regular with aide classes 85; total 263 classes and total 60 schools and only total number of students reported (4744) (two schools had incomplete test data and was removed) |
| Method of estimation | Hierarchical linear regression with random effects and a lagged dependent variable | Hierarchical linear regression and 'raw' ES's also available | None |
| Statistics (risk ratio, odds ratio, standard error, 95 cf, DF, p-value, chi2) | Analysis separate for each grade and reading and math including gender, SES, minority status and achievement in previous grade. Coefficient estimates with stars. | Analysis separate for each grade and reading and math including gender, SES, minority status, low achiever (below median within classes at end of kindergarten) and interaction of small class and low achiever. Coefficient estimates with stars. Cannot be used. Table 1 provides effect sizes (d's) separated by low/high achievers (relative within class at end of kindergarten) (no regression). | Reproduction of the Technical report (Word, 1994)s (math/reading average scores) table p. 47/47 and figure p. 54/53, figure p.65/64, figure p.78/77 and figure p. 92/93 and (own) calculation of yearly value added and 3 years average of value added |
| Page numbers and notes | Table 4 p. 343 | Table 1 and 2 p. 208 and 209 | Table 1 and 2 p. 402 and 407 |
| Level of aggregation | Two level model (student and school) | Two level model (student and school) | Students and teachers randomised and class is the level of analysis |
| Notes | STAR reanalysis grade 1-3, special sample. Grade 2 and 3 judged 5 in the incomplete outcome data item | Table 1 provides effect sizes (d's) separated by low/high achievers (relative within class at end of kindergarten) (no regression). Interact treatment with low achiever. The analysis is repeated by lowest quartile definition of low achiever. A repeated measures analysis of the data is also available | STAR reanalysis, value-added analysis |

| Author | Schanzenbach, 2007 | Shin, 2011 | Shin, 2012 |
| --- | --- | --- | --- |
| Type of outcome | Continuous |  |  |
| Outcome (there may be more than one, record them all) | Pooled math and reading test scores |  |  |
| Time Point (s) (record the exact time, there may be more than one, record them all) | End of grade KG, 1, 2 and 3. End of grade 4, 5,6 7 and 8 grade |  |  |
| Source (questionnaire, admin data, other(specify) or unclear) | Composite score in math and reading. Stanford Achievement Test (SAT) for grade KG-3 and probably was the Tennessee Comprehensive Assessment Program (TCAP, Comprehensive Tests of Basic Skills (CTBS/McGraw Hill, 1989) and Basic Skills First (BSF) criterion-referenced tests) used for the Lasting Benefits Study (grade 4-8) |  |  |
| Valid Ns (only applicable for continuous outcome data). Mention treatment and comparison. | Report number of teachers per year in Small/Regular classes: KG: 127/99, 1 grade: 123/115, 2 grade: 131/100, 3 grade: 138/89. Do not remove the teachers who received training in 2 grade. Do not report numbers used for grade 4-8 |  |  |
| Method of estimation | Regression with race, gender, free lunch covariates and school-by-entry wave fixed effects. Also separate estimates by the covariates and teacher experience. |  |  |
| Statistics (risk ratio, odds ratio, standard error, 95 cf, DF, p-value, chi2) | Coefficients and SEs clustered on class room. ITT analysis |  |  |
| Page numbers and notes | Table 4 and 5 p 214 and 217 |  |  |
| Level of aggregation | Student |  |  |
| Notes | STAR ITT reanalysis using composite math and reading. Also provide results for composite test score for 4, 5, 6, 7 and 8 grade. | Analyses new comers only (The sample data consist of 6,325, 2,314, 1,679, and 1,283 new students). Results cannot be used | Analyses new comers only (The sample data consist of 6,325, 2,314, 1,679, and 1,283 new students). Results cannot be used |

| Author | Sohn, 2015 | Word, 1990 | Word 1994 |
| --- | --- | --- | --- |
| Type of outcome |  | Continuous | Continuous |
| Outcome (there may be more than one, record them all) |  | Math and reading | Math and reading |
| Time Point (s) (record the exact time, there may be more than one, record them all) |  | End of year kindergarten, 1,2 and 3 grade | End of year kindergarten, 1,2 and 3 grade |
| Source (questionnaire, admin data, other(specify) or unclear) |  | Scores in math and reading. Stanford Achievement Test (SAT) | Scores in math and reading. Stanford Achievement Test (SAT) |
| Valid Ns (only applicable for continuous outcome data). Mention treatment and comparison. |  | Numbers not reported but from the Technical report (Word, 1994): Kindergarten number of classes/students: small classes 127/1678, regular classes: 103/2049, regular with aide classes 98/2007; total 328/5734 and 79 schools; 1. grade number of classes: small classes 122, regular classes 111, regular with aide classes 98; total 331 classes and total 76 schools and only total number of students reported (6572); 2. grade number of classes: small classes 109, regular classes 79, regular with aide classes 85; total 273 classes and total 62 schools and only total number of students reported (5328) (reduction due to removal of 67 teachers who received Project Star training); 3. grade number of classes: small classes 110, regular classes 68, regular with aide classes 85; total 263 classes and total 60 schools and only total number of students reported (4744) (two schools had incomplete test data and was removed) | Kindergarten number of classes/students: small classes 127/1678, regular classes: 103/2049, regular with aide classes 98/2007; total 328/5734 and 79 schools; 1. grade number of classes: small classes 122, regular classes 111, regular with aide classes 98; total 331 classes and total 76 schools and only total number of students reported (6572); 2. grade number of classes: small classes 109, regular classes 79, regular with aide classes 85; total 273 classes and total 62 schools and only total number of students reported (5328) (reduction due to removal of 67 teachers who received Project Star training); 3. grade number of classes: small classes 110, regular classes 68, regular with aide classes 85; total 263 classes and total 60 schools and only total number of students reported (4744) (two schools had incomplete test data and was removed) |
| Method of estimation |  | None (analysis-of-variance model results only reported as a summary of the analyses showing significance levels (.05, .01, .001, all levels are <=) | None (analysis-of-variance model results only reported as a summary of the analyses showing significance levels (.05, .01, .001, all levels are <=) |
| Statistics (risk ratio, odds ratio, standard error, 95 cf, DF, p-value, chi2) |  | ES for each grade and outcome and divided by SES and for grade 1-3 also for bottom quartile previous year available | ES for each grade and outcome and divided by SES and for grade 1-3 also for bottom quartile previous year available |
| Page numbers and notes |  | Table 7 p. 20 | Figure X-3 p. 188 |
| Level of aggregation |  | Students and teachers randomised and students is the level of analysis (in the model we use) | Students and teachers randomised and students is the level of analysis (in the model we use) |
| Notes | STAR and LBS reanalysis (CTBS data) 4., 6. and 8. Grade. Analyse number of years in small class and divide on 'effective' (i.e. significant difference) and ineffective schools (also show total). Results cannot be used | Small class 13-17 students, regular 21-28 students (the relevant results are based upon analyses conducted by Dr. John Folger, Vanderbilt University and reported in Folger, 1989, where it is reported that the size of regular classes used is 21-28) | Small class 13-17 students, regular 21-28 students (the relevant results are based upon analyses conducted by Dr. John Folger, Vanderbilt University and reported in Folger, 1989, where it is reported that the size of regular classes used is 21-28) |

## Risk of bias

### Risk of bias non-STAR studies

| Author | Achilles, 1995 | Akerhielm, 1995 | Angrist, 1999 |
| --- | --- | --- | --- |
| **Sequence generation (Judgement)** | High |  | High |
| **Allocation concealment (Judgement)** | High |  | High |
| **Blinding (Judgement)** | 4 |  | 4 |
| **Incomplete outcome data addressed (Judgement)** | Unclear |  | Unclear |
| **Incomplete outcome data addressed (Description, quote from paper or describe key information)** | Nothing reported except the number of schools are intact throughout the three years |  | Nothing reported |
| **Free of selective reporting (Judgement)** | 1 |  | 1 |
| **Free of selective reporting (Description, quote from paper or describe key information)** |  |  |  |
| **Free of other bias (Judgement)** | 1 |  | 4 |
| **Free of other bias (Description, quote from paper or describe key information)** |  |  | Depending on whether full sample, discontinuity (either 5 or 3) and whether and how covariates are included the results vary a lot |
| **A priori protocol (Judgement)** | No |  | Unclear |
| **A priori protocol (Description, quote from paper or describe key information)** | Only a priori plans for the pilot study (p. 25-26) |  |  |
| **A priori analysis plan (Judgement)** | No |  | Unclear |
| **A priori analysis plan (Description, quote from paper or describe key information)** | Only a priori plans for the pilot study (p. 25-26) |  |  |
| **Confounding (Judgement)** | 5 | 5 | 5 |
| **Confounding (Description, quote from paper or describe key information)** | The 'matching' on school level at the beginning of the third and last year of the treatment period essentially excludes 3 of the 10 control schools (they have 4 treatment schools). Method of matching not described. Distribution of classes among schools not reported. 4 T schools with 17 classes and 7 C schools with 26 classes. Total number of pupils/classes at school level not reported | Use average grade class size as instrument, but actual class size is part of this instrument so it is probably not exogenous. | Restrict the sample to schools with classes less than 45 students (meaning that some may have more than 40 so the rule apparently does not strictly bind for all, even though it seems like that looking at figure 1, they report that a few classes have more than 40 students). Only grade (of the prespecified confounders) are considered and separate analysis on grade performed. |
| **Method for identifying relevant confounders described by researchers. Yes/No - if Yes describe the method used.** | None |  | Yes, discussion p- 546 ff |
| **Relevant confounders described (See relevant sheet and list confounders and note if they were considered, precise, imbalanced or adjusted)** | School level ethnicity (percent white), SES as percent of Chapter one (not explained what that is), percent on free/reduced lunch, percent parents with less than high school and school last year average test score in reading and math, all considered and 'matched' at the beginning of the third (and last) year of the treatment period. Not considered: age, gender, baseline achievement and local education spending |  | None, except grade (separate models). In addition school SES and enrolment included, imbalances not shown but some discussion of possible direction of bias |
| **Method used for controlling for confounding (At design state)** | None |  | Include only Jewish public schools and exclude independent religious schools. IV using max class size (Maimonides rule) |
| **Method used for controlling for confounding (At analysis stage)** | Matching at school level in the third (and last) year of the treatment period |  | IV and several sample restrictions and model specifications |
| **Notes** | Burke County (California) | NELS data |  |

| Author | Angrist, 2014 | Annevelink, 2004 | Blatchford, 2003a |
| --- | --- | --- | --- |
| **Sequence generation (Judgement)** | High | High | High |
| **Allocation concealment (Judgement)** | High | High | High |
| **Blinding (Judgement)** | 4 | 4 | 4 |
| **Incomplete outcome data addressed (Judgement)** |  | 4 |  |
| **Incomplete outcome data addressed (Description, quote from paper or describe key information)** |  | Response rate at school level is 39% (implying the schools are not representative). Missing data at student level 27% (no analysis of differences). Do not report if the number of schools (46) and classes (73) are intact |  |
| **Free of selective reporting (Judgement)** |  | 1 |  |
| **Free of selective reporting (Description, quote from paper or describe key information)** |  |  |  |
| **Free of other bias (Judgement)** |  |  |  |
| **Free of other bias (Description, quote from paper or describe key information)** |  |  |  |
| **A priori protocol (Judgement)** |  | Unclear |  |
| **A priori protocol (Description, quote from paper or describe key information)** |  |  |  |
| **A priori analysis plan (Judgement)** |  | Unclear |  |
| **A priori analysis plan (Description, quote from paper or describe key information)** |  |  |  |
| **Confounding (Judgement)** | 5 | 5 |  |
| **Confounding (Description, quote from paper or describe key information)** | Uses Maimonides rule (although it is not at all strictly followed (see figure 2), especially for grade 5 (North and Centre region) and grade 2 (South region) it is not convincing, see figure 4 and 5) but also show that the estimated class size effect probably is due to score manipulation especially in the South region. Corrects for score manipulation by adding score manipulation instrumented by Maimonides rule to the, now multivariate, regression. The max class size rule is however a very weak instrument for score manipulation | Invite all relevant schools (118) (see p. 9, those that have 1 grade classes small or large relative to grade 2, 3 and 4 classes) to participate, although only among the 185 schools who participated in an evaluation of the implementation of the CS reduction initiative in 1999-2000 (where schools could choose between reducing CS, hire extra teachers in classes and provide extra support). Do not consider age and local education spending. Do not consider imbalances on confounders, do not even report the distribution of class size over schools (there could be only one deviation from the average or all deviations in the same school). |  |
| **Method for identifying relevant confounders described by researchers. Yes/No - if Yes describe the method used.** |  | Some discussion |  |
| **Relevant confounders described (See relevant sheet and list confounders and note if they were considered, precise, imbalanced or adjusted)** |  | Do not consider age and local education spending. Do not consider/show imbalances. Do not report average class sizes nor the distribution of class size over schools (there could be only one deviation from the average or all deviations in the same school) |  |
| **Method used for controlling for confounding (At design state)** |  | Invite all relevant schools (see p. 9, those that have 1 grade classes small or large relative to grade 2,3 and 4 classes) to participate, although only among the 185 schools who participated in an evaluation of the implementation of the CS reduction initiative in 1999-2000 |  |
| **Method used for controlling for confounding (At analysis stage)** |  | Multilevel analysis (using MLWin, nothing else reported) |  |
| **Notes** |  | 1 graders | Collection of results from British Class Size Study. Cannot assess RoB as not enough information is provided |

| Author | Blatchford, 2002 | Blatchford, 2003b | Bonesrønning, 2003 |
| --- | --- | --- | --- |
| **Sequence generation (Judgement)** | High | High | High |
| **Allocation concealment (Judgement)** | High | High | High |
| **Blinding (Judgement)** | 4 | 4 | 4 |
| **Incomplete outcome data addressed (Judgement)** | 4 |  | 5 |
| **Incomplete outcome data addressed (Description, quote from paper or describe key information)** | Missing data at student level 48% (no analysis of difference). Missing data at class or school level not reported. Do report that the number of classes (368) is not intact as a few very small and very large classes appeared to be anomalous and were excluded, number not reported. Do not report if the number of schools (220) is intact. | Initially number of local education authorities (LEAs) 9, schools 199, classes 330 and students 7142 | Attrition rate 40%. Starts with 2815 and 1684. 931 are used in analysis due to missing data (45% missing of the 1684). Total attrition and missing data level 67%. |
| **Free of selective reporting (Judgement)** | 5 |  | 5 |
| **Free of selective reporting (Description, quote from paper or describe key information)** | Only cohort 1 in year one is analysed. Why is data for cohort 1 year 2 and 3 and cohort 2 for all three years not analysed? In Blatcford et al., 2003 the cohorts are (best guess) analysed together and also the year 2 and 3 data. No significant effects found except in few specialised cases. |  | Do not show first stage regression results or discussing them. No sensitivity concerning discontinuity samples are performed |
| **Free of other bias (Judgement)** | 1 |  | 5 |
| **Free of other bias (Description, quote from paper or describe key information)** |  |  | Unclear what the effect measures. They have test scores for 4 time points starting fall 1998 and class size variable is from spring 1999. They do not report which test scores are used. |
| **A priori protocol (Judgement)** | Unclear |  | No |
| **A priori protocol (Description, quote from paper or describe key information)** |  |  |  |
| **A priori analysis plan (Judgement)** | Unclear |  | No |
| **A priori analysis plan (Description, quote from paper or describe key information)** |  |  |  |
| **Confounding (Judgement)** | 4 |  | 5 |
| **Confounding (Description, quote from paper or describe key information)** | Random selection of schools within the 8 participating local education authorities (LEAs) (?in Blatcford et al, 2003 it is reported that there is 9 LEAs, 199 schools (not 220 as in this paper), 330 classes (not 368 as in this paper) and 7142 students (not 9330 as in this paper)). Do not consider local education spending. Do not consider imbalances on confounders, do not even report the distribution of class size over schools (there could be only one deviation from the average or all deviations in the same school). |  | Include one school that has two classes, one of size 50 and the other at size 51, although the maximum class size in Norway is 30. This school is an experimental school where classes always have more than one teacher. Do not show imbalances nor discuss them. Do not report or mention anything concerning first stage regression. Unclear when student and family background data are from, could be post variables |
| **Method for identifying relevant confounders described by researchers. Yes/No - if Yes describe the method used.** | Some discussion |  | Some discussion |
| **Relevant confounders described (See relevant sheet and list confounders and note if they were considered, precise, imbalanced or adjusted)** | All controlled for except local education spending. More confounders added. Do not consider/show imbalances. Do not report average class sizes or the distribution of class size over schools (there could be only one deviation from the average or all deviations in the same school). Not even the number of classes and schools used in the analysis is reported, student missing data level 48% (according to the number reported used in analysis and the number reported as available in this paper). In Blatchford et al., 2003, the reported number of available schools is 10% lower, classes 10% lower and students 23% lower (numbers used in analysis not reported) |  | All except age considered but no imbalances shown or discussed |
| **Method used for controlling for confounding (At design state)** | None |  | IV using max class size (Maimonides rule) |
| **Method used for controlling for confounding (At analysis stage)** | Multilevel model with random coefficients. Class size is measured about an origin of 30. Uses a cubic regression spline with two knots (19 and 25) |  | IV and including covariates in regression |
| **Notes** | British Class Size Study (uses only cohort 1 and first year results only) | No results reported other than graphs without CI. Have not finished RoB |  |

| Author | Boozer, 2001a | Boozer, 1995 | Boozer, 2001b |
| --- | --- | --- | --- |
| **Sequence generation (Judgement)** |  | High | High |
| **Allocation concealment (Judgement)** |  | High | High |
| **Blinding (Judgement)** |  | 4 | 4 |
| **Incomplete outcome data addressed (Judgement)** |  |  | 3 |
| **Incomplete outcome data addressed (Description, quote from paper or describe key information)** |  |  | No information given other than they have a full sample of 873 individuals aged 8 to 13 years. Uses 569 in analysis probably due to missing data (level 35%) |
| **Free of selective reporting (Judgement)** |  | 4 | 1 |
| **Free of selective reporting (Description, quote from paper or describe key information)** |  | They pool the test subjects. They have 4 subjects: social science, math, reading, and science, but only appr. two observations per student: For each student, the teachers from two of the four test subjects were also surveyed.... teachers were asked about the student’s class size. By matching the class size with the test score for that particular subject, they create student-class records for each student in the sample. They have about two observations per student |  |
| **Free of other bias (Judgement)** |  | 4 | 4 |
| **Free of other bias (Description, quote from paper or describe key information)** |  | Each student counts twice in the regression, see the Selective reporting item | They use The Burt Word Reading Test: - a student reads from a list of 110 words and her score is the number of words read correctly. Generally, as the children age, they naturally tend to become better at this exercise, and so one of the most prominent features of the test scores over time is this growth in scores by age. Possibly they reach a ceiling at age 13 |
| **A priori protocol (Judgement)** |  |  | No |
| **A priori protocol (Description, quote from paper or describe key information)** |  |  |  |
| **A priori analysis plan (Judgement)** |  |  | No |
| **A priori analysis plan (Description, quote from paper or describe key information)** |  |  |  |
| **Confounding (Judgement)** | 5 | 5 | 5 |
| **Confounding (Description, quote from paper or describe key information)** | Uses state pupil/teacher ratio as instrument for actual class size. Pupil-teacher ratios are defined as the number of students in the school divided by the number of full-time teachers for an entire school and may have nothing to do with actual class size. This ratio often includes guidance counsellors, principals, and special education teachers in the count of teachers, thus the lower the ratio, the higher the non-teaching staff, regardless of actual class size. Moreover, even if the ratio only includes actual teachers, schools with the same pupil-teacher ratio may have significantly different class sizes depending on the average number of hours of teaching required. Apparently their sample includes remedial and special needs students (they do not report they exclude them) Unclear whether the state average pupil/teacher ratio is for the relevant grades only (probably not) | Uses state special education policy as instrument. Include the logarithm of the maximum class size (of special education classes), the logarithm of the maximum class size squared and dummies indicating that the state does not regulate the maximum class size for five out of seven special education categories, generating 15 instruments. Apparently their sample also includes remedial and special needs students but probably not only (they do not report anything) | Number of schools and classes and in general assignment to classes not mentioned or discussed. No imbalances shown or discussed (except gender imbalances). Do not control for pre-test, grade and local educational spending |
| **Method for identifying relevant confounders described by researchers. Yes/No - if Yes describe the method used.** |  |  | Endogeneity and the use of long run gain scores and quantile regression is discussed on p. 23 and 24 |
| **Relevant confounders described (See relevant sheet and list confounders and note if they were considered, precise, imbalanced or adjusted)** |  |  | Control for gender, mother and father ethnicity, and the change in family income. No imbalance shown. Do not consider grade, pre-test and local educational spending |
| **Method used for controlling for confounding (At design state)** |  |  | Not sure, on p. 7 they write: What is left after obliterating both the cohort level secular age effects and the individual level average differences is the growth in test scores for each individual. To the extent policies make a difference in these scores, this residual variation should represent a relatively clean lens with which to view such effects. |
| **Method used for controlling for confounding (At analysis stage)** |  |  | OLS regression and quantile regression using long run gain scores (from age 8 to 13) |
| **Notes** | NELS data |  | Christchurch Health and Development Study (CHDS) data. Children aged 8-13 |

| Author | Borland, 2005 | Bosworth, 2014 | Bressoux, 2009 |
| --- | --- | --- | --- |
| **Sequence generation (Judgement)** | High | High | High |
| **Allocation concealment (Judgement)** | High | High | High |
| **Blinding (Judgement)** | 4 | 4 | 4 |
| **Incomplete outcome data addressed (Judgement)** | Unclear | Unclear | 2 |
| **Incomplete outcome data addressed (Description, quote from paper or describe key information)** | Nothing is reported concerning missing data | Nothing reported | Missing data level for students is 15% and 7% for classes, after imputation is done (scores for at least one of 4 exercises in each test) |
| **Free of selective reporting (Judgement)** | 1 | 1 | 1 |
| **Free of selective reporting (Description, quote from paper or describe key information)** |  |  |  |
| **Free of other bias (Judgement)** | 1 | 1 | 1 |
| **Free of other bias (Description, quote from paper or describe key information)** |  |  |  |
| **A priori protocol (Judgement)** | No | Unclear | Unclear |
| **A priori protocol (Description, quote from paper or describe key information)** |  |  |  |
| **A priori analysis plan (Judgement)** | No | Unclear | Unclear |
| **A priori analysis plan (Description, quote from paper or describe key information)** |  |  |  |
| **Confounding (Judgement)** | 5 | 3 | 3 |
| **Confounding (Description, quote from paper or describe key information)** | Estimate a system of four endogenous variables, achievement, class size, teacher salary and education competition, but do not discuss at all the variables included and excluded in the different equations and the choices do not seem obvious (for example gender is included in class size and excluded in achievement, mean of class innate ability is included in class size and the standard deviation of the same variable is included in achievement along with individual innate ability). Use 3 grade only. | Have access to a rich data set which they unfortunately do not use on an individual basis but on a classroom basis in the analysis on an individual level. | Restrict sample to novice teacher classes so pre-test scores is not significant correlated with class size. All confounders + more controlled for but no imbalances concerning class size is shown or discussed (except pre-test) |
| **Method for identifying relevant confounders described by researchers. Yes/No - if Yes describe the method used.** | Some discussion at an overall level | Yes especially concerning the various ability measures they have access to (but unfortunately do not use on an individual basis) | Yes, especially on trained and experienced teachers, ending up with exclusion of experienced teachers from the analysis of class size. This implies that pre-test scores is no longer significant correlated with class size |
| **Relevant confounders described (See relevant sheet and list confounders and note if they were considered, precise, imbalanced or adjusted)** | Use grade 3 only. No imbalances shown or discussed | Individual gender, ethnicity, age and SES not considered; class composition regarding gender, ethnicity and SES (and several ability variables) controlled for. No imbalances shown or discussed (except about 300000 separate tests of whether classrooms appear to be the result of a random draw) | All considered + more. No imbalances concerning class size are shown or discussed (except that pre-test scores are not significantly correlated with class size), only the possible ways there can be selection bias |
| **Method used for controlling for confounding (At design state)** | Not explained | None | Exclude experienced teachers and argue (and test via initial achievement scores) that trained and untrained novice teachers are randomly assigned to classes (p. 549). One of the arguments is that the system of assignment of teachers to schools is centralised. The sample covers all novice teachers in the 12 'departments' selected for the survey (conducted by the French Ministry of Education) |
| **Method used for controlling for confounding (At analysis stage)** | System of four endogenous variables estimated | Regression with school, year and grade fixed effects | Regression with class random effect included |
| **Notes** |  | North Carolina Education Research Data Center (NCERDC) data for 4 and 5 grade |  |

| Author | Breton, 2012 | Burde, 1990 | Carpenter, 2003 |
| --- | --- | --- | --- |
| **Sequence generation (Judgement)** | High |  | High |
| **Allocation concealment (Judgement)** | High |  | High |
| **Blinding (Judgement)** | 4 |  | 4 |
| **Incomplete outcome data addressed (Judgement)** | 3 |  | 4 |
| **Incomplete outcome data addressed (Description, quote from paper or describe key information)** | Missing data level varies between 39-47% |  | Only report the numbers used (used complete data). From Blatchford et al. (2002) we know that missing data at student level is 48% (no analysis of difference). Missing data at class or school level not reported. From Blatchford et al. (2002) the initial number of classes (368) and schools (220) are reported. Here they report using 254 classes and 157 schools; missing data level is 31% for classes and 29% for schools. |
| **Free of selective reporting (Judgement)** | 1 |  | 5 |
| **Free of selective reporting (Description, quote from paper or describe key information)** |  |  | Only cohort 1 in year one is analysed. Why is data for cohort 1 year 2 and 3 and cohort 2 for all three years not analysed? In Blatcford et al., 2003 the cohorts are (best guess) analysed together and also the year 2 and 3 data. No significant effects found except in few specialised cases. |
| **Free of other bias (Judgement)** | 3 |  | 1 |
| **Free of other bias (Description, quote from paper or describe key information)** | Class size is measured with possible errors which are corrected for but the measure of class size used is an estimate |  |  |
| **A priori protocol (Judgement)** | Unclear |  | Unclear |
| **A priori protocol (Description, quote from paper or describe key information)** |  |  |  |
| **A priori analysis plan (Judgement)** | Unclear |  | Unclear |
| **A priori analysis plan (Description, quote from paper or describe key information)** |  |  |  |
| **Confounding (Judgement)** | 5 | 5 | 4 |
| **Confounding (Description, quote from paper or describe key information)** | Important confounders not considered. No imbalances shown or discussed. Unobservables taken care of by eliminating students from schools that group by ability | Select at random 400 students (50% females) from a total of 111,199 students who completed the fall 1988 fourth grade MEAP tests (Michigan Department of Education, 1989). From this sample every fourth male and every fourth female student was selected for inclusion with the sample analysed. Provide correlation coefficients separated by gender. No other confounders shown or discussed | Do not consider imbalances on confounders, do not even report the distribution of class size over schools (there could be only one deviation from the average or all deviations in the same school). |
| **Method for identifying relevant confounders described by researchers. Yes/No - if Yes describe the method used.** | No |  | Do not discuss anything, state that they retain parameters with estimates that were significant at the 5% level in Blatchford et al. (2002). |
| **Relevant confounders described (See relevant sheet and list confounders and note if they were considered, precise, imbalanced or adjusted)** | Age, baseline achievement and SES not considered. More is considered. No imbalances shown or discussed |  | All controlled for, except local education spending. More confounders added. Do not consider/show imbalances. Do not report average class sizes or the distribution of class size over schools (there could be only one deviation from the average or all deviations in the same school). |
| **Method used for controlling for confounding (At design state)** | None |  | None |
| **Method used for controlling for confounding (At analysis stage)** | Linear regression |  | Multilevel model with random coefficients. Class size is measured about an origin of 30. Uses a cubic regression spline with knot at 25 |
| **Notes** |  |  | British Class Size Study data |

| Author | Chargois, 2008 | Clanet, 2010 | Costello, 1992 |
| --- | --- | --- | --- |
| **Sequence generation (Judgement)** | High | High | High |
| **Allocation concealment (Judgement)** | High | High | High |
| **Blinding (Judgement)** | 4 | 4 | 4 |
| **Incomplete outcome data addressed (Judgement)** | Unclear | Unclear | Unclear |
| **Incomplete outcome data addressed (Description, quote from paper or describe key information)** | Nothing reported | Nothing reported | Nothing reported on how many they tested (randomly chose students in spring 1995 and used tests from spring 1996) |
| **Free of selective reporting (Judgement)** | 1 | 4 | 1 |
| **Free of selective reporting (Description, quote from paper or describe key information)** |  | Only report the significance level and only sign of the effects that are significant |  |
| **Free of other bias (Judgement)** | 1 | 1 | 1 |
| **Free of other bias (Description, quote from paper or describe key information)** |  |  |  |
| **A priori protocol (Judgement)** | No | No | Unclear |
| **A priori protocol (Description, quote from paper or describe key information)** |  |  |  |
| **A priori analysis plan (Judgement)** | No | No | Unclear |
| **A priori analysis plan (Description, quote from paper or describe key information)** |  |  |  |
| **Confounding (Judgement)** | 5 | Unclear | 5 |
| **Confounding (Description, quote from paper or describe key information)** | Do not consider any confounders | No confounders considered | Not controlled for anything except grade |
| **Method for identifying relevant confounders described by researchers. Yes/No - if Yes describe the method used.** | None | No | No |
| **Relevant confounders described (See relevant sheet and list confounders and note if they were considered, precise, imbalanced or adjusted)** | None | None | All participants in study were first graders. Otherwise no confounders tested or group equivalence documented |
| **Method used for controlling for confounding (At design state)** | None | The French Ministry of Education created 101 small size class rooms. Not reported who or how the 99 control classes were created | Randomly selected students from the two small classes and randomly selected students from the two large classes |
| **Method used for controlling for confounding (At analysis stage)** | None | None | None |
| **Notes** |  | No useful outcome data can be extracted |  |

| Author | Dee, 2011 | Dennis, 1986 | Dharmadasa, 1995 |
| --- | --- | --- | --- |
| **Sequence generation (Judgement)** | High |  | High |
| **Allocation concealment (Judgement)** | High |  | High |
| **Blinding (Judgement)** | 4 |  | 4 |
| **Incomplete outcome data addressed (Judgement)** | 3 |  | 2 |
| **Incomplete outcome data addressed (Description, quote from paper or describe key information)** | 19,396 students from public schools. Two teacher questionnaires are available for only 16,901 of these students (87%), thus the sample used is limited to 33,802 student-by-subject observations. Students missing are more likely to be minorities and low achieving (p. 28). Apparently there are missing data (probably test score) for some of these students. In the analysis (without teacher fixed effects) 29,724–31,140 observations are used (88%-92%) and in the analysis with student and teacher fixed effects 13,865–14,586 students are used (82%-86%) |  | Missing data level 12% (reading) and 10% (math) |
| **Free of selective reporting (Judgement)** | 1 |  | 1 |
| **Free of selective reporting (Description, quote from paper or describe key information)** |  |  |  |
| **Free of other bias (Judgement)** | 1 | 5 | 1 |
| **Free of other bias (Description, quote from paper or describe key information)** |  |  |  |
| **A priori protocol (Judgement)** | No |  | No |
| **A priori protocol (Description, quote from paper or describe key information)** |  |  |  |
| **A priori analysis plan (Judgement)** | No |  | No |
| **A priori analysis plan (Description, quote from paper or describe key information)** |  |  |  |
| **Confounding (Judgement)** | 4 |  | 5 |
| **Confounding (Description, quote from paper or describe key information)** | Only eight grade student are included. Student (and teacher) fixed effects so by construction all confounders are controlled for. In a footnote it is stated that gender-specific subject fixed effects are included but unclear what that is given student fixed effects are included |  | Do not consider any confounders |
| **Method for identifying relevant confounders described by researchers. Yes/No - if Yes describe the method used.** | Yes likely bias is discussed |  | None |
| **Relevant confounders described (See relevant sheet and list confounders and note if they were considered, precise, imbalanced or adjusted)** | Use only data from the 815 public schools in the data set. Student fixed effects and teacher fixed effects and class room observables (Teacher of opposite race/ethnicity, teacher of opposite gender, teacher certified by state in subject and % classmates with limited English proficiency). Estimates with student and teacher fixed effects without control for class room observables are also available. The teacher variables act as moderators (and thus blurs the magnitude of the effect) but the % classmates with limited English proficiency may be an important confounder to control for (although imbalance is not reported) |  | None |
| **Method used for controlling for confounding (At design state)** | Rely on contemporaneous within-student, within-teacher comparisons across two academic subjects. |  | None |
| **Method used for controlling for confounding (At analysis stage)** | Student and teacher fixed effects |  | None |
| **Notes** | NELS data. First difference between subjects, cannot be used | Cannot identify effect: Treated in one school and control in another school |  |

| Author | Dieterle, 2013 | Dobbelsteen, 2002 | Ecalle, 2006 |
| --- | --- | --- | --- |
| **Sequence generation (Judgement)** |  | High | Unclear (see notes) |
| **Allocation concealment (Judgement)** |  | High | Unclear |
| **Blinding (Judgement)** |  | 4 | 4 |
| **Incomplete outcome data addressed (Judgement)** |  | Unclear | Unclear |
| **Incomplete outcome data addressed (Description, quote from paper or describe key information)** |  | Nothing reported | Probably some missing data, but not precisely stated other than there were 100 classes in each condition and data were obtained from 570 children in the experimental group and 622 children in the control group and they state they randomly chose students from control classes (but 100 small classes with 10-12 students should give at least 1000 students from small classes). In addition, the number of students in T/C is interchanged: T: 570 students, C: 622 students (what text says and first row in table 1 reporting pre-tests). Second row in table 1 and table 2 (with results) says 622 T and 570 C and further pre-test subdivided on covariates are only shown for 496 T and 385 C which are also the numbers analysed in table 4 (with results but only for normal aged children) |
| **Free of selective reporting (Judgement)** |  | 1 | 4 |
| **Free of selective reporting (Description, quote from paper or describe key information)** |  |  | They state they perform two separate multivariate analyses of covariance (MANCOVA) on the two outcomes (It is most likely two ANCOVAs) but only report 5% significant effects (Class size NS). Results in table 4 are only for normal aged children (approximately 50 excluded in each condition) |
| **Free of other bias (Judgement)** |  | 5 | 4 |
| **Free of other bias (Description, quote from paper or describe key information)** |  | Instrument probably more correlated with outcome (additional resources to schools with many low SES students) than with class size (can use additional resources to add teachers to classrooms) | Not reported whether there are both small and normal classes within the same school. Number of schools not reported either. State there were 100 classes in each condition and data were obtained from 570 children in the experimental group and 622 children in the control group and they state they randomly chose students from control classes (but 100 small classes with 10-12 students should give at least 1000 students from small classes). In addition, the number of students in T/C is interchanged (see incomplete data) |
| **A priori protocol (Judgement)** |  | Unclear | Unclear |
| **A priori protocol (Description, quote from paper or describe key information)** |  |  |  |
| **A priori analysis plan (Judgement)** |  | Unclear | Unclear |
| **A priori analysis plan (Description, quote from paper or describe key information)** |  |  |  |
| **Confounding (Judgement)** |  | 5 | NR |
| **Confounding (Description, quote from paper or describe key information)** |  | Separate analysis for grade level. Included gender and SES in analysis. Do not consider age, baseline achievement and local education spending. Instrument based on total enrolment at school level (or rather a SES weighted enrolment rule where the amount of resources given to a school depends on the SES weighted number of students). Very small differences at the points of discontinuity (1 or 2 students in average class size). Partial correlation coefficient between instrument and class size is 0.38/0.47/0.43 for grades 4/6/8 |  |
| **Method for identifying relevant confounders described by researchers. Yes/No - if Yes describe the method used.** |  | Discussion p. 22-23 |  |
| **Relevant confounders described (See relevant sheet and list confounders and note if they were considered, precise, imbalanced or adjusted)** |  | Individual level: gender, weight factor accounting for SES. Class: teachers gender, class average of pupils gender and weight factor, dual teacher class. School: Average SES, total enrolment. No imbalances are reported and do not consider age, baseline achievement and local education spending. The discontinuities of the instrument are very small (1 or 2 students) |  |
| **Method used for controlling for confounding (At design state)** |  | IV |  |
| **Method used for controlling for confounding (At analysis stage)** |  | 2SLS Regression |  |
| **Notes** | Only have data at required level for two of three grades and do not provide useable separate results | PRIMA survey Dutch | 1 graders from low SES areas only. 100 T classes and 100 C classes were chosen from schools achieving very poor results in 3 grade tests. Teachers were randomly allocated (within schools? This is not stated explicit) but the method is not described. Students were randomly selected within the schools and randomly assigned to teachers. In addition children in normal classes were randomly chosen to be compared to those in classes of small size (but there should be at least 1000 students from small classes unless they were also chosen randomly for analysis). |

| Author | Galton, 2012 | Gerritsen, 2017 | Gilman, 1988a |
| --- | --- | --- | --- |
| **Sequence generation (Judgement)** | High | High | High |
| **Allocation concealment (Judgement)** | High | High | High |
| **Blinding (Judgement)** | 4 | 4 | 4 |
| **Incomplete outcome data addressed (Judgement)** | Unclear | Grade 2: 1 and Grade > 2: 4 | Unclear |
| **Incomplete outcome data addressed (Description, quote from paper or describe key information)** | No mentioning of attrition from treatment and comparison classes during the four years of study (there must have been some students leaving, or maybe not, it is in Hong Kong?) | Of the available twins in grade > 2, there are 44 percent without pre-tests. | Nothing reported |
| **Free of selective reporting (Judgement)** | 1 | 1 | 1 |
| **Free of selective reporting (Description, quote from paper or describe key information)** |  |  |  |
| **Free of other bias (Judgement)** | 1 | 1 | 2 |
| **Free of other bias (Description, quote from paper or describe key information)** |  |  | Locally constructed tests of basic skills in reading and math. For the purpose of this study the number of items were reduced compared to the original test. No explanation of why this was done |
| **A priori protocol (Judgement)** | Unclear | No | No |
| **A priori protocol (Description, quote from paper or describe key information)** |  |  | State hypotheses p. 7 but is a secondary analysis of the data |
| **A priori analysis plan (Judgement)** | Unclear | No | No |
| **A priori analysis plan (Description, quote from paper or describe key information)** |  |  |  |
| **Confounding (Judgement)** | 5 | Grade 2: 1; Grade > 2: 5 | 5 |
| **Confounding (Description, quote from paper or describe key information)** | Gender, classification of school as disadvantaged. Grade is controlled for by design. No imbalances shown or discussed, 'baseline achievements' are not taken at baseline, a post variable (motivation) is included, age, individual SES and local education spending not controlled for, analysis 3 and 4 compare cohorts in different calendar years, analysis 1 and 2 compare students in the same cohort (and same calendar year) but no description of how allocation was done, not even if T/C classes were in the same schools or not | All and more controlled for (except pre-test for the grade 2 sample). Students in Dutch primary education are reassigned to classes after grade 2. It might be expected that teachers, parents and students will have more information after grade 2 about themselves and other students which might lead to non-random selection into classes. Separate analyses for grade 2 and the whole sample. Do not control for grade when using the whole sample but controls for pre-test assuming the value of pre-test of twins in grade 2 are the same. Lose 27 percent of the total sample even with this assumption (44 percent of the grade > 2 sample). Sensitivity of the results for teacher experience on grade is shown and the results are very different depending on grade | Nothing considered or discussed |
| **Method for identifying relevant confounders described by researchers. Yes/No - if Yes describe the method used.** | No | Discussion | No |
| **Relevant confounders described (See relevant sheet and list confounders and note if they were considered, precise, imbalanced or adjusted)** | Gender, classification of school as disadvantaged. Grade is controlled for by design. Other covariates included (Principal characteristics), baseline achievement (varies between analyses when it is measured: A1 and A2: end of P2, A3: start of P1 and A4: end of P1) and students end of P4 motivation (this is a post variable and should not be included) Not included: age, individual SES and local education spending | Age, grade, school and family are identical. Gender, teacher gender, teacher experience, one full time or two half time teachers per class, mixed grades, class gender and class natives are controlled for. No imbalances are shown or discussed, only show that the variance in teacher characteristics within twin pairs are much larger than variation in class size | Nothing considered or discussed although only 1. Graders are analysed and C/T are (probably) from the same 3 schools. Control group is from 1983/84 and treated from 1984/85, 1985/86, 1986/87 and 1987/88 |
| **Method used for controlling for confounding (At design state)** | Separate analyses by grade 3 cohorts of students: Cohort 1 starts P1 in 04/05 in small classes, P2 in 05/06 some in small and some in large (unsure what is going on, the text says: In the same year (must be 2005 as they have just mentioned cohort 2 who starts in September 2005) Cohort 1 pupils who entered P2 normal classes at the commencement of the study were followed into P3, P3 in 06/07 some in small and some in normal, P4 in 07/08 all in normal; Cohort 2 starts P1 in 05/06 in small, P2 in 06/07 in small, P3 in 07/08 in normal; Cohort 3 starts in P1 in 06/07 in normal, P2 in 07/08 in normal | Assignment of twins to different class rooms | RD in time |
| **Method used for controlling for confounding (At analysis stage)** | 2 level HLM 4 analyses (note this is the description in section 3, not the same as the description on page 362)): Analysis 1: Cohort 1 students compared on end of P4 attainment T: small class in P1, P2 and P3 and C: those who were in normal classes in P3 in 06/07 (not sure about their status in P1 but in normal classes in P2 and probably also in P1), Analysis 2: cohort 1 students: end of P3 attainment, T: small class in P3 in 06/07 and C: normal classes in P3 in 05/06 (?something is wrong with the years if all are Cohort 1 students); Analysis 3: T: cohort 2 students in P1 in 05/06 and P2 in 06/07 (small class both years) and C: cohort 3 students in P1 in 06/07 and P2 in 07/08 (normal class both years) end of P2 attainment for both groups (at different calendar years); Analysis 4: T: cohort 1 students only those in small classes in P1, P2 and P3 and C: cohort 2 students in small classes in P1 and P2, end of pP3 attainment for both groups in different calendar years | Twin fixed-effect | None |
| **Notes** |  | PRIMA Dutch data | Indiana’s Project Prime Time |

| Author | Gilman, 1988b | Haenn, 2002 | Hallina, 1985 |
| --- | --- | --- | --- |
| **Sequence generation (Judgement)** |  | High | High |
| **Allocation concealment (Judgement)** |  | High | High |
| **Blinding (Judgement)** |  | 4 | 4 |
| **Incomplete outcome data addressed (Judgement)** |  | 1 | 4 |
| **Incomplete outcome data addressed (Description, quote from paper or describe key information)** |  | No missing data as schools are the unit of analysis | 10% did not participate (reduces to appr. 711), some were absent either at October or may test days and some transferred school, total missing data level 43%. Not reported separate for T/C |
| **Free of selective reporting (Judgement)** |  | 1 | Unclear |
| **Free of selective reporting (Description, quote from paper or describe key information)** |  |  |  |
| **Free of other bias (Judgement)** |  | 1 | Unclear |
| **Free of other bias (Description, quote from paper or describe key information)** |  |  |  |
| **A priori protocol (Judgement)** |  | Unclear | No |
| **A priori protocol (Description, quote from paper or describe key information)** |  |  |  |
| **A priori analysis plan (Judgement)** |  | Unclear | No |
| **A priori analysis plan (Description, quote from paper or describe key information)** |  |  |  |
| **Confounding (Judgement)** |  | 5 | 5 |
| **Confounding (Description, quote from paper or describe key information)** |  | Matching on protest scores. Otherwise no confounder identified or controlled for One to one 'matching' on pre-test (beginning of year test) but it only makes sense for kindergarten as grade 1-5 students are matched on a post test. For all students in the 22 control schools (in the relevant grade) an average of post scores is calculated for those students with exact the same pre-test score as one from the treatment schools and treated as one observation (the SDs reported are thus not correct) | Only pre-test and ethnicity is controlled for. No imbalances shown or discussed on any confounders, do not mention what grades are included, include both public and private schools |
| **Method for identifying relevant confounders described by researchers. Yes/No - if Yes describe the method used.** |  | No | None |
| **Relevant confounders described (See relevant sheet and list confounders and note if they were considered, precise, imbalanced or adjusted)** |  | Treatment schools are all inner city schools with large proportions of disadvantaged students. Control schools not mentioned. 'Match' on pre-test (beginning of year) | Do not consider age, local education spending (they include both public and private schools). Pre-test and ethnicity controlled for and separate analysis on classes with grouped/non grouped instruction. Also included gender and grade in analysis but as they showed no consistent effects (only significant in few of the 6 regressions (reading and math and all students and separate by grouping/not) they were left out. No imbalances shown or discussed, not even reported what grades are included |
| **Method used for controlling for confounding (At design state)** |  | Matching on pre-test score | Regression |
| **Method used for controlling for confounding (At analysis stage)** |  | One to one matching on beginning of year test, only makes sense for kindergarten | None |
| **Notes** | Indiana’s Project Prime Time but only for the treatment year 1985 | Match on beginning of year scores, only makes sense for kindergarten, for grade 1-5 beginning of year is post score for those who have spent more than a year in the same school (treatment at school level begins in 94/95 and data is probably from 2001). Two comparison schools as well (do not use as they are not 'matched') |  |

| Author | Hirschfeld, 2016 | Hojo, 2011 | Hojo, 2013 |
| --- | --- | --- | --- |
| **Sequence generation (Judgement)** |  | High | High |
| **Allocation concealment (Judgement)** |  | High | High |
| **Blinding (Judgement)** |  | 4 |  |
| **Incomplete outcome data addressed (Judgement)** |  | Unclear |  |
| **Incomplete outcome data addressed (Description, quote from paper or describe key information)** |  | Drop observations with missing data and exclude private schools. Do not report how many students were dropped due to missing data or attending a private school |  |
| **Free of selective reporting (Judgement)** |  | 1 |  |
| **Free of selective reporting (Description, quote from paper or describe key information)** |  |  |  |
| **Free of other bias (Judgement)** |  | 5 |  |
| **Free of other bias (Description, quote from paper or describe key information)** |  | As robustness check they conduct analyses for subgroups (table 5), among others the (only) relevant sample of discontinuities (of 5) and a threshold of 41. For math the coefficients varies between -0.381 to 2.943 and for science between -0.206 to -6.979 (only for these three: whole sample, discontinuity sample and threshold 41) |  |
| **A priori protocol (Judgement)** |  | No |  |
| **A priori protocol (Description, quote from paper or describe key information)** |  |  |  |
| **A priori analysis plan (Judgement)** |  | Unclear |  |
| **A priori analysis plan (Description, quote from paper or describe key information)** |  |  |  |
| **Confounding (Judgement)** | 5 | 4 | 5 |
| **Confounding (Description, quote from paper or describe key information)** | Correlation with only class size and no covariates at all | Uses Maimonides rule with a maximum number of 40 which can be slightly modified at the local government level (footnote 6) and approx. 10% of the classes have 41 students (figure 3). According to Hoxby (2000): between the discontinuities, predicted class size varies with actual enrolment, which is a function of the covariates. Therefore, predicted class size is not a valid instrument except when the rule triggers a change in the number of classes (their main result is based on the total sample. Further, identification arises only when the rule binds, so if one uses a rule that binds only in some schools, one learns about the effects of class size only for those schools | Uses Maimonides rule with a maximum number of 40 and in addition the same rule as a second instrument (as they estimate two effects in a piecewise linear model) coupled with an estimated threshold for the class size where the slope changes (probably where the effect of class size changes but it is not clearly explained) and a dummy indicating above/under this threshold. According to Hoxby (2000): between the discontinuities, predicted class size varies with actual enrolment, which is a function of the covariates. Therefore, predicted class size is not a valid instrument except when the rule triggers a change in the number of classes (they base their result on based on the total sample). Further, identification arises only when the rule binds, so if one uses a rule that binds only in some schools, one learns about the effects of class size only for those schools. The second instrument is not exogenous. The contribution of the paper is nonlinear effects of class size on outcomes, hence the piecewise liner function in t. We probably do not care about linear effects, but we can use eq. 2 and 8 in table 3, which estimate the linear effect of C on Y with Maimonides rule as IV. In eq 1 on the whole sample and in eq 8 on the sample of classes larger than 20. Unfortunately they do not report estimates on discontinuity samples (class size close to the jumps in fig 1), which would have less confounding than the whole sample. |
| **Method for identifying relevant confounders described by researchers. Yes/No - if Yes describe the method used.** |  | Some discussion |  |
| **Relevant confounders described (See relevant sheet and list confounders and note if they were considered, precise, imbalanced or adjusted)** |  | Do not consider baseline achievement and local education spending. Do not show or discuss imbalances. Do not consider whether the rule actually binds (which it probably does not especially in math, see figure 4). Only include public schools |  |
| **Method used for controlling for confounding (At design state)** |  | IV |  |
| **Method used for controlling for confounding (At analysis stage)** |  | Two stage least squares regression |  |
| **Notes** |  | TIMMS 2007 data for Japan | TIMMS 2003 data for Japan |

| Author | Hudson, 2011 | Iacovou, 2002 | Iversen, 2013 |
| --- | --- | --- | --- |
| **Sequence generation (Judgement)** | High | High | High |
| **Allocation concealment (Judgement)** | High | High | High |
| **Blinding (Judgement)** | 4 | 4 | 4 |
| **Incomplete outcome data addressed (Judgement)** | Unclear |  | Unclear |
| **Incomplete outcome data addressed (Description, quote from paper or describe key information)** | Nothing is reported |  | State (p.309) they have math test results for 55,322 students, the number reported in the tables of results are 53,500. Probable reasons for reductions are excluded schools (those who changed their position across the kinks through the three years, not reported how many) or missing data (nothing reported). Furthermore, at p. 319 some potential measurement errors are mentioned, but nothing further concerning what kind or for how many observations, only that they are discarded in the RD analysis. They are probably included in the IV analysis. Not all students participate in the test taking but only school average participation rates around the two first kinks are shown |
| **Free of selective reporting (Judgement)** | 1 |  | 5 |
| **Free of selective reporting (Description, quote from paper or describe key information)** |  |  | Do not report the number of schools excluded (those who changed their position across the kinks through the three years). Do not report how many students have moved school and thereby potentially class size. Mention one (or more, it is unclear) analysis at p. 314 but no results are shown and the coefficient mentioned in the text as being the coefficient of interest is not included in the equation shown (eq. 3). Do not show first stage regression results. Show enrolment distributions only for the post treatment year, not the treatment years. Have test results for reading but include only math. Sensitivity analysis concerning discontinuity samples only performed for sub groups (less educated parents and dissolved families) and only for the first kink (as no effects are found for the sub groups at the second kink) |
| **Free of other bias (Judgement)** | 5 for science score and 1 for reading and math |  | 5 |
| **Free of other bias (Description, quote from paper or describe key information)** | They use reading class size in the science score equation |  | Do not have the actual class size in the treatment years, only the post treatment year. Interpret treatment and results as if the duration of intervention is 3 years. Exclude schools that changed their position across the kinks through the three years but they end up using class size in grade 3 only and the students class size can still vary if the student has changed school. Not all students participate in the test taking but only the mean of school average participation rates around the two first kinks are shown |
| **A priori protocol (Judgement)** | No |  | No |
| **A priori protocol (Description, quote from paper or describe key information)** |  |  |  |
| **A priori analysis plan (Judgement)** | No |  | No |
| **A priori analysis plan (Description, quote from paper or describe key information)** |  |  |  |
| **Confounding (Judgement)** | 4 | 5 | 5 |
| **Confounding (Description, quote from paper or describe key information)** | All confounders controlled for and in addition: percent school white students, school size, percent minority students in class and a lot of teacher background variables (page 21). | Page 272: "Although it is quite likely that both the size and type of school are related to student outcomes, the interaction between these two variables may be used as an exogenous instrument for class size. The only assumptions that need be made are, first, that the interaction terms are related to class size (which they are, as we shall show) and, second, that they are not related to student attainment. This second assumption is equivalent to assuming that school size and school type both may affect student performance, but that their effects are independent: in other words, being in a school with 500 rather than 300 students has the same effect on performance regardless of whether the school is an infant or a combined school; and the effect of being in an infant or a combined school does not vary according to whether it is a large or a small school." Table 4 show that the two interaction terms they use (infantXsize and combinedXsize squared) (unclear why the second interaction term is used) have impacts of low magnitude although statistically significant: 0.009 (t value 2.997) and -0.004 (t value 7.633). We do not understand these arguments at all. In what direction should the behaviour change in order to fulfil the monotonicity assumption? What is complier/defier behaviour? | Only test of grade 4 is included, but student age, gender and prior achievement is not reported and SES only at school level. Do not properly show imbalances and do not have actual class size data for the treatment year(s). |
| **Method for identifying relevant confounders described by researchers. Yes/No - if Yes describe the method used.** | Discussion | Discussion and analysis of ability and class size | Yes, discussion |
| **Relevant confounders described (See relevant sheet and list confounders and note if they were considered, precise, imbalanced or adjusted)** | All and more confounders controlled for. No imbalances shown or discussed | Instrument used is the interaction between school size and school type (average class sizes in infant schools, taking children from age 5–7, are larger than in combined schools, taking children from age 5–11) | Show 'balancing of samples' in table 2 around the two first kinks (diff. of 5 on both sides) but only at school level and probably not for the treatment year (s) (year not reported); most likely it is for the post treatment year. Only test of grade 4 is included, but student age, gender and prior achievement is not reported and SES only at school level. Test participation rates only shown as the mean of school averages around the two first kinks. The IV results use all observations including those at the kinks. Further analyses using what is termed 'a classical RD' analysis is performed on sub groups only (less educated parents and dissolved families) and excludes observation that are not generated by the rule-triggered change in class size (diff of 5 students on both sides), observations that suffer from potential measurement errors (not reported what and how many) and observations right at the kink (probably enrolment rates 28 and 56) and performed separately for the first kink and the second kink. |
| **Method used for controlling for confounding (At design state)** | Regression and school random effects model | IV and exclude classes less than 20 students and more than 45 students | Include only fourth graders and exclude schools that have changed their position across the kinks through the three years. Seems unnecessary as they end up using class size in grade 3 only and the student’s class size can still vary if the student has changed school. IV using max class size (Maimonides rule) |
| **Method used for controlling for confounding (At analysis stage)** | OLS and GLS regression |  | IV and several sample restrictions and model specifications |
| **Notes** | NELS data. | Data from the National Child Development Study |  |

| Author | Jakubowski, 2006 | Konstantopoulos, 2016a | Konstantopoulos, 2016b |
| --- | --- | --- | --- |
| **Sequence generation (Judgement)** | High |  | High |
| **Allocation concealment (Judgement)** | High |  | High |
| **Blinding (Judgement)** | 4 |  | 4 |
| **Incomplete outcome data addressed (Judgement)** | 2 |  | Unclear |
| **Incomplete outcome data addressed (Description, quote from paper or describe key information)** | "From 8834 classes in public schools (class is there unit of analysis) we excluded 143 classes (1.6%) with 40 or more students, because they cannot be run legally, so we treated them as an error. We also decided to eliminate schools which have classes with 10 or less pupils for enrolment levels greater than 20 (27 cases) (0.3%). Visual inspection of eliminated data shows that omitted schools were either incorrectly coded or have classes for pupils with special needs. |  | Missing Class size data 0.5 percent for 4. Grade and 4-11 percent for 8. Grade. Enrolment data missing for 1 percent of grade 4 and 1-9 percent for grade 8. No other information on missing student or teacher data reported |
| **Free of selective reporting (Judgement)** | 1 |  | 4 |
| **Free of selective reporting (Description, quote from paper or describe key information)** |  |  | Results of sensitivity analyses not shown and only t-values for covariate balancing reported (and not shown for the sensitivity analysis either) |
| **Free of other bias (Judgement)** | 1 |  | 1 |
| **Free of other bias (Description, quote from paper or describe key information)** |  |  |  |
| **A priori protocol (Judgement)** | Unclear |  | No |
| **A priori protocol (Description, quote from paper or describe key information)** |  |  |  |
| **A priori analysis plan (Judgement)** | Unclear |  | No |
| **A priori analysis plan (Description, quote from paper or describe key information)** |  |  |  |
| **Confounding (Judgement)** | 5 | 5 | 5 |
| **Confounding (Description, quote from paper or describe key information)** | Two analyses with different instruments are performed. One using average grade class size, but actual class size is part of this instrument so it is probably not exogenous. The second is a constructed max class size rule (there is no such rule in Poland, the authors term it a theoretical class size function when in fact it is rather an empirical function). The relationship between the constructed max rule and actual class size (see figure 1) is not convincing | OLS regression and IV, using maximum class size rule for 14 countries (the IV class size is based on enrolment by the end of the school year and not the beginning which makes it potentially endogenous). Cannot identify private and public schools in the data. The researchers suggest: the IVs are weak in Denmark, Croatia, Italy and Malta (table 5). Both coefficients and t-statistics from first stage are reported in table 5 p 524. Note that coefficients are different from the ones reported in Li & Konstantopoulos (2017) for Denmark, Lithuania, Portugal and Romania even though the number of observations used is identical. The coefficients for the countries where the researchers suggest the instruments are strong vary between 0.38 to 0.62. Only the coefficient for Spain reaches 0.7 but for this country several of the covariates are not balanced (table 4). Figures of enrolment and class size shown for all countries. The authors suggest that the countries shown in figure 1/2/3 the schools followed the maximum rule very well/well/not well. We do not agree, none of the figures are convincing. No country specific information is given (this is a great concern as for example Nandrup (2016) shows that half the municipalities in Denmark do not abide to the rule), except for Hungary were it is noted that in certain cases the maximum can be raised by 30 percent. Several of the second stage coefficients are of the 'wrong' sign. They have tested covariates are balanced around cut-offs but only for a sample of +/- 5 around the cut offs which is not used for the main analysis (only the full sample is used for analysis) | IV using maximum rule and RD around cut off (5 and 3 but only 5 is shown). Report t-values of covariates around discontinuity and not coefficient values; sample size is low for the discontinuity sample thus the tests may lack power. In addition they do not have parent education (SES variable) for grade 4 students which is a covariate with a high t-value for 8. Grade in 2003 (but not in 2007, which is odd) and base line achievement for any grade (4 or 8)). The instrument is clearly not strong for grade 8 (correlation around 0.3-0.36). It correlates well with actual class size in grade 4 (0.8) but it is based on enrolment at time of testing and not by the start of the school year which makes it potentially endogenous. |
| **Method for identifying relevant confounders described by researchers. Yes/No - if Yes describe the method used.** | Yes, in the introduction and methods section |  |  |
| **Relevant confounders described (See relevant sheet and list confounders and note if they were considered, precise, imbalanced or adjusted)** | No |  |  |
| **Method used for controlling for confounding (At design state)** | IV and use public primary schools only (and other restrictions on schools included such as no schools for children with special needs) |  |  |
| **Method used for controlling for confounding (At analysis stage)** | IV (two different) 2SLS with school fixed effect |  |  |
| **Notes** |  | TIMMS data from 2011, 4. Grade. | TIMMS data from 2003 and 2007, 4. (only 2003) and 8. grade |

| Author | Konstantopoulos, 2014 | Krueger, 2002 | Lavy, 2001 |
| --- | --- | --- | --- |
| **Sequence generation (Judgement)** | High | High | High |
| **Allocation concealment (Judgement)** | High | High | High |
| **Blinding (Judgement)** | 4 | 4 | 4 |
| **Incomplete outcome data addressed (Judgement)** | Unclear | 4 | 1 |
| **Incomplete outcome data addressed (Description, quote from paper or describe key information)** | Nothing reported, not even size of the sample used is reported | Uses same data set as Lindahl (2001). Nothing reported here, but from Lindahl (2001): Nothing reported concerning number of students but of those taking at least one (of four) tests at all three time points 40% had no missing data. The author scaled each test part on each test occasion in percentile ranks, ignoring any missing test part score and took the average of these percentile rank scores on each occasion. | Number of classes used/available is 2047/2056 (4 grade) and 2016/2029 (5 grade) |
| **Free of selective reporting (Judgement)** | 1 | 1 | 4 |
| **Free of selective reporting (Description, quote from paper or describe key information)** |  |  | Only full sample results are shown |
| **Free of other bias (Judgement)** | 1 | 2 | 1 |
| **Free of other bias (Description, quote from paper or describe key information)** |  | See Lindahl 2001 |  |
| **A priori protocol (Judgement)** | Unclear | No | Unclear |
| **A priori protocol (Description, quote from paper or describe key information)** |  |  |  |
| **A priori analysis plan (Judgement)** | Unclear | No | Unclear |
| **A priori analysis plan (Description, quote from paper or describe key information)** |  |  |  |
| **Confounding (Judgement)** | 5 | 5 | 5 |
| **Confounding (Description, quote from paper or describe key information)** | Bad controls and Private schools are included in sample but not controlled for in the regression of class size on the instrument. | See Lindahl 2001 and Lindahl 2005 | As number of classes are the same as in Angrist and Lavy (1999) they probably restrict the sample to schools with classes less than 45 students (meaning that some may have more than 40 so the rule apparently does not strictly bind for all, even though it seems like that looking at figure 1 in Angrist and Lavy (1999), they report that a few classes have more than 40 students). Only grade (of the prespecified confounders) are considered and separate analysis on grade performed. |
| **Method for identifying relevant confounders described by researchers. Yes/No - if Yes describe the method used.** | Thorough discussion p. 5-9 | Some discussion | Yes, discussion in section 5 |
| **Relevant confounders described (See relevant sheet and list confounders and note if they were considered, precise, imbalanced or adjusted)** | All except baseline achievement and in addition some bad controls, some very bad controls and one very very bad control. No imbalances shown or discussed | Show correlations between class size and gender family SES (parents nationality, income, education). Include grade 6 only and analyse change scores in and include pre-test in the OLS model. Do not consider age and local education spending. In addition controls for teacher variables. | None, except grade (separate models). In addition school SES and enrolment included, imbalances not shown but some discussion of possible direction of bias. Dummy for religious school included and IV for instruction hours. |
| **Method used for controlling for confounding (At design state)** | Two stage selection of the sample: first schools and then classes within schools (only one class at each school, so effectively not a two stage sampling procedure) and IV | None | Include only Jewish students in public schools in both secular and religious schools, although independent religious schools are excluded. IV using max class size (Maimonides rule) |
| **Method used for controlling for confounding (At analysis stage)** | Two methods: multilevel regression not using the instrument and multilevel regression using the instrument | Regression, two approaches: a diff-in-diff (school year change (grade 6 spring minus fall) minus summer holiday change (grade 6 fall minus grade 5 spring) unconditional on pre-test (the 5 grade spring test) and the school year difference with summer change included in regression although instrumented with pre-test (the 5 grade spring test) | IV |
| **Notes** | PIRLS data from Greece | Only the analysis on Swedish data assessed. Also report STAR results although only graphical results are shown. Also report on a STAR follow up analysis (college entrance exam). 'Score-analysis' gets 5 in confounding (reasons not written here as the book reports on the same analyses as in Krueger and Whitmore (2001) |  |

| Author | Levin, 2001 | Li, 2015 | Li, 2017 |
| --- | --- | --- | --- |
| **Sequence generation (Judgement)** | High | High | High |
| **Allocation concealment (Judgement)** | High | High | High |
| **Blinding (Judgement)** | 4 | 4 |  |
| **Incomplete outcome data addressed (Judgement)** | 3 | Unclear |  |
| **Incomplete outcome data addressed (Description, quote from paper or describe key information)** | Missing data level appr. 22%, nothing else reported |  |  |
| **Free of selective reporting (Judgement)** | 1 | 5 |  |
| **Free of selective reporting (Description, quote from paper or describe key information)** |  | State that they tested if covariates were locally balanced across schools around cut-offs but results are not reported. Only mention on p. 19: "There was some evidence that showed associations between smaller class size and higher student SES level in Spain and Malta based on some regression analysis, which indicates parents with higher SES might manipulates the rules and raises some concern of the validity of the IV in these two countries." |  |
| **Free of other bias (Judgement)** | 1 | 5 |  |
| **Free of other bias (Description, quote from paper or describe key information)** |  | State that they tested if covariates were locally balanced across schools around cut-offs but results are not reported. Only mention on p. 19: "There was some evidence that showed associations between smaller class size and higher student SES level in Spain and Malta based on some regression analysis, which indicates parents with higher SES might manipulates the rules and raises some concern of the validity of the IV in these two countries." |  |
| **A priori protocol (Judgement)** | No | No |  |
| **A priori protocol (Description, quote from paper or describe key information)** |  |  |  |
| **A priori analysis plan (Judgement)** | No | No |  |
| **A priori analysis plan (Description, quote from paper or describe key information)** |  |  |  |
| **Confounding (Judgement)** | 5 | 5 | 5 |
| **Confounding (Description, quote from paper or describe key information)** | Separate analysis for grade level. Included gender and SES in analysis. Do not consider age, baseline achievement and local education spending. Instrument based on total enrolment at school level (or rather a SES weighted enrolment rule where the amount of resources given to a school depends on the SES weighted number of students). Very small differences at the points of discontinuity (1 or 2 students in average class size). In 13739043 it is reported: Partial correlation coefficient between instrument and class size is 0.38/0.47/0.43 for grades 4/6/8 | OLS regression and IV, using maximum class size rule for 18 countries (the IV class size is based on enrolment by the end of the school year and not the beginning which makes it potentially endogenous). Cannot identify private and public schools in the data. Purely based on the level of the t-statistic, the researcher suggests: the IVs might not be valid in Hong Kong, Malta and Spain; also, in Croatia, Denmark, Italy, Malta and Singapore, the IVs were weak, which made the IV estimates and inference unreliable. Both coefficients and t-statistics from first stage are reported in table 1.5 p 27. No country specific information is given, except for Hong Kong where it is noted that the maximum class size rules were only applicable to part of schools (which ones could not be identified). Several of the second stage coefficients are of the 'wrong' sign. State they have tested covariates are balanced around cut-offs but nothing is shown or mentioned (except for Spain and Malta, see the Reporting bias item) | OLS regression and IV, using maximum class size rule for 14 countries (the IV class size is based on enrolment by the end of the school year and not the beginning which makes it potentially endogenous). Cannot identify private and public schools in the data. The researchers suggest: the IVs are weak in Croatia, Italy and Malta (table 2). Both coefficients and F-statistics from first stage are reported in table 2 p 302. Note that coefficients are different from the ones reported in Li & Konstantopoulos (2016) for Denmark, Lithuania, Portugal and Romania even though the numbers of observations used are identical. The coefficients for the countries where the researchers suggest the instruments are strong vary between 0.30 to 0.62. Only the coefficient for Spain reaches 0.7 but for this country several of the covariates are not balanced (table 4 in Li & Konstantopoulos (2016)). Figures of enrolment and class size shown for all countries. The authors suggest that the countries shown in figure 1 the schools followed the maximum rule well (for 5 countries), not well (for 3 countries) and the remaining countries are not commented on. We do not agree that any of the countries seem to follow the rules well, none of the figures are convincing. No country specific information is given (this is a great concern as for example Nandrup (2016) shows that half the municipalities in Denmark do not abide to the rule). Several of the second stage coefficients are of the 'wrong' sign. No mentioning of whether covariates are balanced around cut-offs |
| **Method for identifying relevant confounders described by researchers. Yes/No - if Yes describe the method used.** | Some discussion |  |  |
| **Relevant confounders described (See relevant sheet and list confounders and note if they were considered, precise, imbalanced or adjusted)** | Individual level: gender, weight factor accounting for SES. Class: teachers gender, class average of pupils gender and weight factor, dual teacher class. School: Average SES, total enrolment. No imbalances are reported and do not consider age, baseline achievement and local education spending. The discontinuities of the instrument are very small (1 or 2 students) |  |  |
| **Method used for controlling for confounding (At design state)** | IV |  |  |
| **Method used for controlling for confounding (At analysis stage)** | Two stage least squares quantile regression |  |  |
| **Notes** | PRIMA survey Dutch as Dobbelsteen, 2002, but using quantile regression. Instrument probably more correlated with outcome (additional resources to schools with many low SES students) than with class size (can use additional resources to add teachers to classrooms) | TIMMS data from 2011, 4. Grade. | TIMMS data from 2011, 4. Grade. |

| Author | Lindahl, 2005 | Ma, 2006 | Maier 1997 |
| --- | --- | --- | --- |
| **Sequence generation (Judgement)** | High | High |  |
| **Allocation concealment (Judgement)** | High | High |  |
| **Blinding (Judgement)** | 4 | 4 |  |
| **Incomplete outcome data addressed (Judgement)** | 4 | Unclear |  |
| **Incomplete outcome data addressed (Description, quote from paper or describe key information)** | Nothing reported concerning number of students but of those taking at least one (of four) tests at all three time points 40% had no missing data. The author scaled each test part on each test occasion in percentile ranks, ignoring any missing test part score and took the average of these percentile rank scores on each occasion. | Nothing reported |  |
| **Free of selective reporting (Judgement)** | 1 | 1 |  |
| **Free of selective reporting (Description, quote from paper or describe key information)** |  |  |  |
| **Free of other bias (Judgement)** | 2 | 1 |  |
| **Free of other bias (Description, quote from paper or describe key information)** | The author (not the teachers) graded the tests at all three occasions |  |  |
| **A priori protocol (Judgement)** | Unclear | No |  |
| **A priori protocol (Description, quote from paper or describe key information)** |  |  |  |
| **A priori analysis plan (Judgement)** | Unclear | No |  |
| **A priori analysis plan (Description, quote from paper or describe key information)** |  |  |  |
| **Confounding (Judgement)** | 5 | 5 |  |
| **Confounding (Description, quote from paper or describe key information)** | Both models assume an unobserved ability variable has the same effect on learning in a school year and a summer holiday (of very different lengths). The diff-in-diff model assumes that baseline achievement does not affect learning (neither school year or summer holiday) and a fixed learning effect is removed by subtracting the school year learning (diff in test score percentiles) and the summer learning (diff in test score percentiles over the summer holiday period). It is not likely that the baseline achievement should not affect any of the learnings. The second model assumes baseline achievement does affect learning (and has the same effect on school learning and summer holiday learning) and use baseline achievement as an instrument for the summer learning included in the model of school year learning (may be a bad choice with only three time points, see Judson & Owen, 1999). It is not likely that baseline achievement should have the same effect on school year and summer holiday learning. They use only math classes where class size is different than regular classes (where teaching regular subjects take place). it is much more skewed with very small classes and more correlated with family background variables than regular class size | Do not perform separate analysis for grade level but pool grades. Unclear what is included in the analysis but probably gender, SES. Do not consider age, baseline achievement and local education spending (other than through the instrument). Instrument based on total enrolment at school level (or rather a SES weighted enrolment rule where the amount of resources given to a school depends on the SES weighted number of students). In Levin (2001) it is reported: Very small differences at the points of discontinuity (1 or 2 students in average class size). In Dobbelsteen, Levin & Oosterbeek (2002) it is reported: Partial correlation coefficient between instrument and class size is 0.38/0.47/0.43 for grades 4/6/8. But here they merge grades and do not report any 1. level results |  |
| **Method for identifying relevant confounders described by researchers. Yes/No - if Yes describe the method used.** | Yes | Some discussion |  |
| **Relevant confounders described (See relevant sheet and list confounders and note if they were considered, precise, imbalanced or adjusted)** | Age and local school education spending not considered. Base line achievement considered in one of the models, included as an instrument for change in achievement during the summer (holiday). Imbalances shown as correlation except baseline achievement, no imbalances shown or mentioned. | Individual level: gender, weight factor accounting for SES. Class: teachers gender, class average of pupils gender and weight factor, dual teacher class. School: Average SES, total enrolment. No imbalances are reported and do not consider age, baseline achievement and local education spending. The discontinuities of the instrument are very small (1 or 2 students) |  |
| **Method used for controlling for confounding (At design state)** | None | IV |  |
| **Method used for controlling for confounding (At analysis stage)** | Regression, two approaches: a diff-in-diff (school year change (grade 6 spring minus fall) minus summer holiday change (grade 6 fall minus grade 5 spring) unconditional on pre-test (the 5 grade spring test) and the school year difference with summer change included in regression although instrumented with pre-test (the 5 grade spring test) | Two stage least squares quantile regression |  |
| **Notes** |  | Dutch PRIMARY survey data. Instrument probably more correlated with outcome (additional resources to schools with many low SES students) than with class size (can use additional resources to add teachers to classrooms) | SAGE data: A Regular classroom refers to a classroom with one teacher. Most regular classrooms have 15 or fewer students, but a few exceed 15. A 2-Teacher Team classroom is a class where two teachers work collaboratively to teach as many as 30 students. A Shared-Space classroom is a classroom that has been fitted with a temporary wall that creates two teaching spaces, each with one teacher and about 15 students. A Floating Teacher classroom is a room consisting of one teacher and about 30 students, except during reading, language arts, and mathematics instruction when another teacher joins the class to reduce the ratio to 15:1. Only analyse effect of type of classroom within SAGE schools. Different results for different years |

| Author | Maples, 2009 | McGiverin, 1989 | Merritt, 2011 |
| --- | --- | --- | --- |
| **Sequence generation (Judgement)** | High | High | High |
| **Allocation concealment (Judgement)** | High | High | High |
| **Blinding (Judgement)** | 4 | 4 | 4 |
| **Incomplete outcome data addressed (Judgement)** | Unclear | Unclear | 1 |
| **Incomplete outcome data addressed (Description, quote from paper or describe key information)** |  | Nothing except total numbers analysed is reported | 4% missing data at some items only, and: Two outliers were identified and were removed. Missing data were handled using Full Information Maximum Likelihood approach in Mplus. |
| **Free of selective reporting (Judgement)** | 1 | 1 | 1 |
| **Free of selective reporting (Description, quote from paper or describe key information)** |  |  |  |
| **Free of other bias (Judgement)** | 1 | 1 | 5 |
| **Free of other bias (Description, quote from paper or describe key information)** |  |  | Mail correspondence with author Eileen Merritt: I think at this time, it would be best not to include the study in your review. This was a conference presentation, and results have changed quite a bit after reviewer feedback. It has not yet been published in a peer-reviewed journal, and would be better to be included at that time |
| **A priori protocol (Judgement)** | No | No | No |
| **A priori protocol (Description, quote from paper or describe key information)** |  |  | Data (on control group only) was taken from another RCT |
| **A priori analysis plan (Judgement)** | Yes | No | Unclear |
| **A priori analysis plan (Description, quote from paper or describe key information)** | State hypotheses |  | State a priori research questions |
| **Confounding (Judgement)** | 5 | 5 | 4 |
| **Confounding (Description, quote from paper or describe key information)** | Analyses are separated by grade and gender, otherwise nothing is considered | Analyse second graders for the school years of 1984-85 and 1985-86. PRIME Time implemented for 1. Graders in 1984-85. Analyse schools in this way where both PRIME Time was implemented and schools where it was not and pool the "effect sizes" in a meta-analysis. Some PRIME Time classes. Several studies (=schools) were rejected due to equal class sizes, or, in some instances, larger comparative class sizes for PRIME TIME groups. | No imbalances shown only correlations, CS correlated with class ability level, pre-test and teacher mathematical knowledge and there is a potential for large imbalances concerning the 30 students with special needs (no correlation shown) (the sizes of classes are 11-31). In addition they note themselves: participants in our study came from a large, well-funded suburban school district. In this district, administrators are able to adjust class sizes and personnel to classrooms where students need more help We can only be certain that grade and SES have no imbalances. We agree, that selection is an issue in this paper, but they do control for baseline-scores, teacher quality, and a random class-room intercept |
| **Method for identifying relevant confounders described by researchers. Yes/No - if Yes describe the method used.** |  |  | No, their research questions focused on variability in teacher quality and not class size |
| **Relevant confounders described (See relevant sheet and list confounders and note if they were considered, precise, imbalanced or adjusted)** |  |  | No imbalances shown (only correlations), they mention that: Students were 105 male; 108 Hispanic, 19 Caucasian, 43 African American, and 27 Asian American, and 169 English Language Learners. Also, 30 students received special education services, including 17 who were learning disabled and 9 with speech or language disorders. These 30 students is a potential serious problem if they are all in classes of same size. They have 205 students from 36 classes at 11 schools, no distribution is shown. Concerning teachers: Teachers had a mean of 11 years’ experience (median = 9 years, range 1 – 35 years). This wide range may also be much skewed in terms of class size. Other reported teacher characteristics not likely to matter: 32 Caucasian, 3 African American, 1 Asian and 1 Native American. Thirty-two teachers were fully licensed and 3 were provisionally licensed |
| **Method used for controlling for confounding (At design state)** |  |  | From 11 schools (see notes) all third grade children (n = 205) with designated free or reduced lunch status from 11 schools were included. |
| **Method used for controlling for confounding (At analysis stage)** |  |  | Hierarchical linear regression |
| **Notes** |  | Indiana PRIME Time |  |

| Author | Milesi, 2006 | Molnar, 1998 | Molnar, 1999a |
| --- | --- | --- | --- |
| **Sequence generation (Judgement)** | High | High | High |
| **Allocation concealment (Judgement)** | High | High | High |
| **Blinding (Judgement)** | 4 | 4 | 4 |
| **Incomplete outcome data addressed (Judgement)** | 3 |  |  |
| **Incomplete outcome data addressed (Description, quote from paper or describe key information)** | Of the complete data set 92.6% was retained as teacher and student records could be matched. In the retained data student achievement data was missing in 17% for reading and 12% for math. Further 14% of classes had no information on class size and an additional 9% had no information on the instructional activities (information used to create a variable used in the analysis). They performed mean imputation for four independent variables with a substantive amount of non-response and list wise deletion for the remaining. Percent of data used in reading analysis 64% and math analysis 67%. |  |  |
| **Free of selective reporting (Judgement)** | 1 |  |  |
| **Free of selective reporting (Description, quote from paper or describe key information)** |  |  |  |
| **Free of other bias (Judgement)** | 1 |  |  |
| **Free of other bias (Description, quote from paper or describe key information)** | Robustness check on specification of class size. Comparable results obtained for different categorization of class size (Appendix 1). Robustness check via teacher fixed effect model (Table 6). Additional HLM analyses are carried out (results not shown but described, p. 301) |  |  |
| **A priori protocol (Judgement)** | No |  |  |
| **A priori protocol (Description, quote from paper or describe key information)** |  |  |  |
| **A priori analysis plan (Judgement)** | Unclear |  |  |
| **A priori analysis plan (Description, quote from paper or describe key information)** |  |  |  |
| **Confounding (Judgement)** | 1 |  |  |
| **Confounding (Description, quote from paper or describe key information)** | All + more controlled for. Small imbalance on socioeconomic status and some imbalance on local education spending (half-day/full-day classes) but a model separated on half-day/full-day does not change results (although not shown, only described) |  |  |
| **Method for identifying relevant confounders described by researchers. Yes/No - if Yes describe the method used.** | Discussion p. 294-296 |  |  |
| **Relevant confounders described (See relevant sheet and list confounders and note if they were considered, precise, imbalanced or adjusted)** | All considered and controlled for + more. Some imbalance on socioeconomic status and local education spending (half-day/full-day classes) |  |  |
| **Method used for controlling for confounding (At design state)** | None | During 1997-98 the SAGE program was implemented in 30 schools located in 21 school districts in one state and of 14 Comparison schools located in 7 school districts. Students in 1997-98 were 7,161 active students in 117 kindergarten, 118 first-grade, and 113 second-grade classrooms (initial 8,843 students) |  |
| **Method used for controlling for confounding (At analysis stage)** | Hierarchical linear model and teacher fixed effect model |  |  |
| **Notes** | Early Childhood Longitudinal Study-Kindergarten Class of 1998-1999 | SAGE data, see Maier, 1997 | SAGE data, see Maier, 1997 |

| Author | Molnar, 1999b | Molnar, 2001 | Moshoeshoe, 2015 |
| --- | --- | --- | --- |
| **Sequence generation (Judgement)** | High | High | High |
| **Allocation concealment (Judgement)** | High | High | High |
| **Blinding (Judgement)** | 4 | 4 | 4 |
| **Incomplete outcome data addressed (Judgement)** |  |  |  |
| **Incomplete outcome data addressed (Description, quote from paper or describe key information)** |  |  |  |
| **Free of selective reporting (Judgement)** |  |  |  |
| **Free of selective reporting (Description, quote from paper or describe key information)** |  |  |  |
| **Free of other bias (Judgement)** |  |  |  |
| **Free of other bias (Description, quote from paper or describe key information)** |  |  |  |
| **A priori protocol (Judgement)** |  |  |  |
| **A priori protocol (Description, quote from paper or describe key information)** |  |  |  |
| **A priori analysis plan (Judgement)** |  |  |  |
| **A priori analysis plan (Description, quote from paper or describe key information)** |  |  |  |
| **Confounding (Judgement)** |  |  | 5 |
| **Confounding (Description, quote from paper or describe key information)** |  |  | Use average regional class size as instrument, but actual class size is part of this instrument so it is probably not exogenous. Also control for factors the researcher finds may threat the validity of the instrument. If there is only one class sampled in a district the size of this class is used to proxy the population class size of that region (p. 11), we do not understand this; if there is only one class it is not a proxy for the population class size, it IS the population class size. |
| **Method for identifying relevant confounders described by researchers. Yes/No - if Yes describe the method used.** |  |  |  |
| **Relevant confounders described (See relevant sheet and list confounders and note if they were considered, precise, imbalanced or adjusted)** |  |  |  |
| **Method used for controlling for confounding (At design state)** |  |  |  |
| **Method used for controlling for confounding (At analysis stage)** |  |  |  |
| **Notes** | SAGE data, see Maier, 1997 | SAGE data, see Maier, 1997 |  |

| Author | Munoz, 2001 | Murdoch, 1986 | Maasoumi, 2005 |
| --- | --- | --- | --- |
| **Sequence generation (Judgement)** | High | High | High |
| **Allocation concealment (Judgement)** | High | High | High |
| **Blinding (Judgement)** | 4 | 4 | 4 |
| **Incomplete outcome data addressed (Judgement)** | 1 |  |  |
| **Incomplete outcome data addressed (Description, quote from paper or describe key information)** | To be eligible students had to have pre and post measures so no incomplete data by construction |  |  |
| **Free of selective reporting (Judgement)** | 1 |  |  |
| **Free of selective reporting (Description, quote from paper or describe key information)** |  |  |  |
| **Free of other bias (Judgement)** | 3 |  |  |
| **Free of other bias (Description, quote from paper or describe key information)** | Not all students in the small classes are included. The reason is not reported, could be there was no match so not all treated students could be included but numbers without match or matching procedure is not reported. Could also be because there was no pre-test score on some of the students but nothing is reported |  |  |
| **A priori protocol (Judgement)** | Unclear |  |  |
| **A priori protocol (Description, quote from paper or describe key information)** |  |  |  |
| **A priori analysis plan (Judgement)** | Unclear |  |  |
| **A priori analysis plan (Description, quote from paper or describe key information)** |  |  |  |
| **Confounding (Judgement)** | 4 |  |  |
| **Confounding (Description, quote from paper or describe key information)** | In four schools (out of 34) students (not all) in small classes are matched with students in large classes at the same school. Matched on reading and math scores at beginning of year which may be a post score if they have been in same class size in grade K-2. Moderate imbalance in ethnicity and gender and teacher experience. Both T and C in same school which could act as a poor proxy for local education spending. |  |  |
| **Method for identifying relevant confounders described by researchers. Yes/No - if Yes describe the method used.** | Some during the literature review |  |  |
| **Relevant confounders described (See relevant sheet and list confounders and note if they were considered, precise, imbalanced or adjusted)** | All considered. Moderate imbalance in ethnicity and gender and teacher experience. Both T and C in same school which could act as a poor proxy for local education spending. Match on pre-test scores |  |  |
| **Method used for controlling for confounding (At design state)** | In their own words: "A quasi-experimental design, using aggregated matching procedure for both comparison and treatment group was conducted (N=102 students). Students were matched only from schools with (a) similar socio-economic characteristics, (b) participated in the assessment process at the beginning and at the end of the school year, and (c) having both conditions (regular and reduced class size)" p. 2. Note that only 4 of the possible 34 schools were eligible and again with their own words: "For all the schools participating in the CSR program (N= 1,798 students), the evaluator tested the impact of the program by randomly creating two matched groups: (a) more than 18 students (i.e., comparison group); and, (b) less than 19 students (i.e., treatment group)that participated in the assessment process at the beginning and at the end of the school year" p. 17. And further: "An aggregated matching procedure was used to guarantee that the two groups were equivalent. A total of eight participants were excluded from the analysis after ensuring that the groups were similar in the fundamental academic variables, namely reading and mathematics pre-test scores taken in the Fall of 1999 (i.e., from N = 110 to N = 102 students)." |  |  |
| **Method used for controlling for confounding (At analysis stage)** | None |  |  |
| **Notes** |  | Only report p values from a multivariate model (8 outcomes) with CS, age, gender and school, separated by grade | NELS data, no method/results we can use (first or second order stochastic dominance tests) |

| Author | Nandrup, 2016 | NICHD, 2004 | Otsu, 2015 |
| --- | --- | --- | --- |
| **Sequence generation (Judgement)** | High | High | High |
| **Allocation concealment (Judgement)** | High | High | High |
| **Blinding (Judgement)** | 4 | 4 | 4 |
| **Incomplete outcome data addressed (Judgement)** | Unclear | 4 |  |
| **Incomplete outcome data addressed (Description, quote from paper or describe key information)** |  | Of the original sample (of 8986 mothers who had just given birth was contacted) 1364 (15%) became the study participants (several reductions among others a conditionally (not stated further on what) random was chosen (56%), see p. 654). At follow up (children’s second year of school) the final sample for several reasons is reduced to 651 (48%). Of these missing data on 6% |  |
| **Free of selective reporting (Judgement)** | 5 | 3 |  |
| **Free of selective reporting (Description, quote from paper or describe key information)** | Several important results not shown | Only report significant results (table 4) |  |
| **Free of other bias (Judgement)** | 1 | 1 |  |
| **Free of other bias (Description, quote from paper or describe key information)** |  |  |  |
| **A priori protocol (Judgement)** | No | Unclear |  |
| **A priori protocol (Description, quote from paper or describe key information)** |  |  |  |
| **A priori analysis plan (Judgement)** | No | Unclear |  |
| **A priori analysis plan (Description, quote from paper or describe key information)** |  |  |  |
| **Confounding (Judgement)** | 5 | 4 |  |
| **Confounding (Description, quote from paper or describe key information)** | The actual class size shown in figure 1 never reaches the maximum of 28 (Table 1 reports average class size is 21 with SD of 4), yet the balancing of covariates tests are performed around a cut-off of 28. Only show p-values (for selected covariates) and not magnitude of coefficients. Almost all of their selected covariates have low p-values and even limiting to 4 pupils one of the selected covariates (Non-Western immigrant) is significant (p-value 0.029) and single mother has a p-value of 0.101. The impact (magnitude) of these covariates should be considered. There are more selected covariates (p-values only) reported in the JA and apparently also more included as the p-values differ from those reported in the WP. In the JA Father’s education, mother's age, father’s age, Non-Western immigrant and gestational age have low p-values. This is odd as separate OLS regressions have been performed for each variable and the main results reported in tables 3 and 4 in the JA and WP are exactly identical (with one exception out of 36 results). Nowhere is the first stage results shown or mentioned. Consider the fact that not all municipalities abide to the 28 rule and upon investigation (results of the investigation not shown) of this possibility approximately half the municipalities are left. Separate results for all (98) municipalities and those abiding (48) are shown | Age and local educational spending not considered nor are imbalances. Three bad controls included. |  |
| **Method for identifying relevant confounders described by researchers. Yes/No - if Yes describe the method used.** |  | Some discussion |  |
| **Relevant confounders described (See relevant sheet and list confounders and note if they were considered, precise, imbalanced or adjusted)** |  | All except age and local education spending. More is added of which three (Grade levels in class, Parent engagement and Problems preparing children (all in the class) for success) are assessed in grade 1 and may be considered as potential outcomes. Do not consider/show imbalances. |  |
| **Method used for controlling for confounding (At design state)** | IV maximum class size rule | None |  |
| **Method used for controlling for confounding (At analysis stage)** |  | Multiple regression (they report they use standard hierarchical multiple regression but no hierarchy is presented) |  |
| **Notes** |  | NICHD Study of Early Child Care database | Relevant results are presented graphically and no ES and SE can be extracted. (Uses selected data of Angrist and Lavy (1999); schools with either one or two classes in grade 4) |

| Author | Pollard, 1995 | Pong, 2001 | Sanogo, 1994 |
| --- | --- | --- | --- |
| **Sequence generation (Judgement)** | High | High | High |
| **Allocation concealment (Judgement)** | High | High | High |
| **Blinding (Judgement)** | 4 | 4 | 4 |
| **Incomplete outcome data addressed (Judgement)** | Unclear | 2 |  |
| **Incomplete outcome data addressed (Description, quote from paper or describe key information)** | The study includes students from all of Nevada's 17 school districts. Clark County School District is treated separately (see notes). In the rural and Washoe school districts in 1993 there were 5,326 second grade students included. Many districts did not test students in all of the grades being studied. If a school district did not test for one of the grades in a particular year, then the results exclude that district from the evaluation for that year. Otherwise nothing reported | Restricts the data to math teachers who reported class size and to students linked to teacher. No missing data at student level. One or two teachers in several countries excluded as they did not report age and gender (average number of classes per country is 173). Otherwise a few missing teacher and school variables, they applied the mean imputation method to fill in (whatever that means). Very small (<5) classes removed (primarily affects Iceland where approx. 10% of classes are removed) |  |
| **Free of selective reporting (Judgement)** | 1 | 1 |  |
| **Free of selective reporting (Description, quote from paper or describe key information)** |  |  |  |
| **Free of other bias (Judgement)** | 1 | 1 |  |
| **Free of other bias (Description, quote from paper or describe key information)** |  | Selected nine TIMSS participant countries on three criteria: world region, class size, and educational governance |  |
| **A priori protocol (Judgement)** | No | No |  |
| **A priori protocol (Description, quote from paper or describe key information)** |  |  |  |
| **A priori analysis plan (Judgement)** | No | Unclear |  |
| **A priori analysis plan (Description, quote from paper or describe key information)** |  |  |  |
| **Confounding (Judgement)** | 5 | 5 |  |
| **Confounding (Description, quote from paper or describe key information)** | Nothing considered. Unclear how the control group is selected, probably they are from schools were not enough teachers could be hired/enrolment higher than expected | Important confounders not considered and student imbalances not shown or discussed |  |
| **Method for identifying relevant confounders described by researchers. Yes/No - if Yes describe the method used.** | No | Some discussion p. 252-253 |  |
| **Relevant confounders described (See relevant sheet and list confounders and note if they were considered, precise, imbalanced or adjusted)** | No | All, except age, baseline achievement and local spending considered. In addition school and class level variables. Class size is used as a continuous variable so imbalances cannot be assessed (also model using lowest and highest quintiles and a middle group as reference, but class size in the groupings not reported) |  |
| **Method used for controlling for confounding (At design state)** | None | None |  |
| **Method used for controlling for confounding (At analysis stage)** | None, perform multiple regression with student characteristic included (do not report which variables) but only report percent explained by class size and student characteristics | Hierarchical linear model |  |
| **Notes** | Clark County School District was treated separately throughout the evaluation because they test students during the fall of a school year rather than in the spring. That district submitted a sample of 412 students from the third grade in 1993 rather than all of the approximately 10,000 targeted students. In 1994 Clark County did not test third grade students, but submitted the results from over 10,000 fourth grade students. In analysing the Clark County results, the fall tests for the third and fourth grades are compared with the spring tests for the second and third grades respectively. | TIMSS data. 8. graders | Reproduction of STAR and Indiana PRIME Time results (Word et al. 1990 and Tillitsky, Gilman, Mohr, and Stone, 1988). Do not report what types of classes are included in the PRIME Time results and the original paper (Tillitsky et al. 1988) is unavailable. |

| Author | Shapson, 1980 | Tienken, 2009 | Tillitsky, 1988 |
| --- | --- | --- | --- |
| **Sequence generation (Judgement)** | Unclear (see notes) | High | High |
| **Allocation concealment (Judgement)** | Unclear | High | High |
| **Blinding (Judgement)** | 4 | 4 | 4 |
| **Incomplete outcome data addressed (Judgement)** | Unclear | Unclear | Unclear |
| **Incomplete outcome data addressed (Description, quote from paper or describe key information)** | Nothing reported on either attrition nor missing data | No information about missing data. They included all available participants who met the inclusion criteria | Nothing reported other than total number of students analysed (not divided on T/C) and that only students receiving T or C for all three years are included |
| **Free of selective reporting (Judgement)** | 4 | 1 | 1 |
| **Free of selective reporting (Description, quote from paper or describe key information)** | They do not report outcomes for all groups for all years, so we cannot determine the effect of being randomized to one of the four arms. Report only pairwise test statistics for the significant differences (one out of 4 outcome measures is significant). |  |  |
| **Free of other bias (Judgement)** | 4 | 1 | 1 |
| **Free of other bias (Description, quote from paper or describe key information)** | For the second year the same teachers and students were randomised with the constraints that students should not be in the smallest and largest (of 4 sizes) classes for both years and teachers should not be assigned the two largest classes both years. Do not report data for the two years separately and students were not (necessary) in the same class size each year |  |  |
| **A priori protocol (Judgement)** | Unclear | Unclear | No |
| **A priori protocol (Description, quote from paper or describe key information)** |  |  |  |
| **A priori analysis plan (Judgement)** | Unclear | Unclear | No |
| **A priori analysis plan (Description, quote from paper or describe key information)** |  |  |  |
| **Confounding (Judgement)** |  | 5 | 5 |
| **Confounding (Description, quote from paper or describe key information)** |  | Only pre-test considered (t test showing no difference), nothing controlled for although same grade levels in same school but in different years. Cohort 1 attended 6-8 grade in 1999-2001, cohort 2 6-8 grade in 2001-2004 and cohort 3 in 2003-2006 | Nothing considered or discussed |
| **Method for identifying relevant confounders described by researchers. Yes/No - if Yes describe the method used.** |  | None | No |
| **Relevant confounders described (See relevant sheet and list confounders and note if they were considered, precise, imbalanced or adjusted)** |  | Only pre-test considered (t test showing no difference), nothing controlled for although same grade levels. | Nothing considered or discussed although analysis separated by grade (1-3). Control group is from 1983/84-1985/86 and treated from 1984/85-1986/87 |
| **Method used for controlling for confounding (At design state)** |  | None | RD in time |
| **Method used for controlling for confounding (At analysis stage)** |  | None | None |
| **Notes** | Both students and teachers were randomised (students were stratified on gender and academic performance), not described how and whether it was within schools. For the second year the randomisation was constrained for both teachers and students (see other bias). Do not report outcomes for all groups for all years, so we cannot determine the effect of being randomised to one of the four arms. |  |  |

| Author | Uhrain, 2016 | Urquiola, 2006 | Watson, 2016 |
| --- | --- | --- | --- |
| **Sequence generation (Judgement)** | High | High | High |
| **Allocation concealment (Judgement)** | High | High | High |
| **Blinding (Judgement)** | 4 | 4 | 4 |
| **Incomplete outcome data addressed (Judgement)** |  | Unclear |  |
| **Incomplete outcome data addressed (Description, quote from paper or describe key information)** |  | Nothing reported other than not all variables have valid data for the total sample size of 3,594 (Rural public) |  |
| **Free of selective reporting (Judgement)** |  | 1 |  |
| **Free of selective reporting (Description, quote from paper or describe key information)** |  |  |  |
| **Free of other bias (Judgement)** |  | 5 |  |
| **Free of other bias (Description, quote from paper or describe key information)** |  | Less than 1/4 of the teachers report that in their classrooms more than 50% of the students has textbooks. Half the teachers are in the lowest pay category or without category and possibly not certified and will often not have finished high school. Six percent of classrooms do not have a blackboard. No division on class size of these potential very important confounders are shown or discussed. |  |
| **A priori protocol (Judgement)** |  | No |  |
| **A priori protocol (Description, quote from paper or describe key information)** |  |  |  |
| **A priori analysis plan (Judgement)** |  | No |  |
| **A priori analysis plan (Description, quote from paper or describe key information)** |  |  |  |
| **Confounding (Judgement)** | 5 |  | 5 |
| **Confounding (Description, quote from paper or describe key information)** | Linear regression with only class size and no covariates at all |  | Controls for gender and language background only. Multivariate regression and canonical correlation analyses performed. No imbalances mentioned or shown |
| **Method for identifying relevant confounders described by researchers. Yes/No - if Yes describe the method used.** |  |  |  |
| **Relevant confounders described (See relevant sheet and list confounders and note if they were considered, precise, imbalanced or adjusted)** |  |  |  |
| **Method used for controlling for confounding (At design state)** |  |  |  |
| **Method used for controlling for confounding (At analysis stage)** |  |  |  |
| **Notes** |  |  |  |

| Author | Wenfan, 2005 | West, 2006 | Wierman, 2005 |
| --- | --- | --- | --- |
| **Sequence generation (Judgement)** | High | High | High |
| **Allocation concealment (Judgement)** | High | High | High |
| **Blinding (Judgement)** | 4 | 4 | 4 |
| **Incomplete outcome data addressed (Judgement)** | 3 |  | Unclear |
| **Incomplete outcome data addressed (Description, quote from paper or describe key information)** | No information about missing data. Of the population 29% is excluded as they move class/school/teacher or has limited English proficiency and cannot be assessed. |  | Report there is a small number of missing values (after restricting the data set on other variables) but do not report the numbers |
| **Free of selective reporting (Judgement)** | 5 |  | 1 |
| **Free of selective reporting (Description, quote from paper or describe key information)** | Report that they build up a series of regression models (no further information except they employ weights and takes the cluster sampling design into account). The results table (table 4) is not understandable, for each outcome there are 3 steps all including the same variables and no information as to what is the difference. No class size effects reported in the Math/General knowledge columns but in the text the coefficients in step 2/3 of the reading columns are reported as the effects on math/general knowledge |  |  |
| **Free of other bias (Judgement)** |  |  | 4 |
| **Free of other bias (Description, quote from paper or describe key information)** |  |  | Results were the sample is divided into states w/wo Central Exit Exams are very different in magnitude from each other and the results of the pooled sample |
| **A priori protocol (Judgement)** |  |  | No |
| **A priori protocol (Description, quote from paper or describe key information)** |  |  |  |
| **A priori analysis plan (Judgement)** |  |  | No |
| **A priori analysis plan (Description, quote from paper or describe key information)** |  |  |  |
| **Confounding (Judgement)** |  | 5 | The math/physics 4 and the chemistry/biology and the reading/biology 5 |
| **Confounding (Description, quote from paper or describe key information)** |  | Use average grade class size as instrument, but actual class size is part of this instrument so it is probably not exogenous. Also control for schools by estimating school fixed effects | All is controlled for, except baseline achievement which is considered by the way they handle the subject specific ability. It is assumed identical for math/physics, chemistry/biology and reading/biology in order to identify the relevant class size parameters. |
| **Method for identifying relevant confounders described by researchers. Yes/No - if Yes describe the method used.** |  |  | Some discussion |
| **Relevant confounders described (See relevant sheet and list confounders and note if they were considered, precise, imbalanced or adjusted)** |  |  | Students of age 15 and in grade 9 only. Use individual differences between subjects, this way SES, local education spending and general ability is controlled for. Gender is controlled for as the impact may be subject specific and likewise concerning school track. The subject specific ability is assumed identical for math/physics, chemistry/biology and reading/biology in order to identify the relevant class size parameters |
| **Method used for controlling for confounding (At design state)** |  |  | Restricting the data set and using first differences, i.e. using pairwise test score differences among five subjects |
| **Method used for controlling for confounding (At analysis stage)** |  |  | Linear Seemingly Unrelated Regression (SURE) |
| **Notes** | Early Childhood Longitudinal Study 1998-1999. We cannot use the results as it is unclear what the 3 steps are and the reporting of results in the text do not match the table headings | The aim is to identify sorting effects by decomposing the correlation between class size and achievement into the causal effect of class size on achievement and the bias introduced by sorting. In doing so they apply school fixed effects and use average CS at each grade in each school as instrument | First difference between subjects, cannot be used |

| Author | Wößmann, 2005a | Wößmann, 2005b | Wößmann, 2006 | Wößmann, 2003 |
| --- | --- | --- | --- | --- |
| **Sequence generation (Judgement)** | High | High | High | High |
| **Allocation concealment (Judgement)** | High | High | High | High |
| **Blinding (Judgement)** | 4 | 4 | 4 | 4 |
| **Incomplete outcome data addressed (Judgement)** |  |  |  |  |
| **Incomplete outcome data addressed (Description, quote from paper or describe key information)** |  |  |  |  |
| **Free of selective reporting (Judgement)** |  |  |  |  |
| **Free of selective reporting (Description, quote from paper or describe key information)** |  |  |  |  |
| **Free of other bias (Judgement)** |  |  |  |  |
| **Free of other bias (Description, quote from paper or describe key information)** |  |  |  |  |
| **A priori protocol (Judgement)** |  |  |  |  |
| **A priori protocol (Description, quote from paper or describe key information)** |  |  |  |  |
| **A priori analysis plan (Judgement)** |  |  |  |  |
| **A priori analysis plan (Description, quote from paper or describe key information)** |  |  |  |  |
| **Confounding (Judgement)** | 5 | 5 | 5 | 5 |
| **Confounding (Description, quote from paper or describe key information)** | Uses two strategies: the one also used in Wößmann and West (2006), the second exploiting discontinuities in class size induced by maximum class-size rules. Apply the rule to only 10 of 15 countries where there graphically seems to be few exceptions to the rule. However, the first stage results (using actual class size) are too week and continues using grade average class size. | Use average grade class size as instrument, but actual class size is part of this instrument so it is probably not exogenous. Also control for schools by estimating school fixed effects | Use average grade class size as instrument, but actual class size is part of this instrument so it is probably not exogenous. Also control for schools by estimating school fixed effects | Use average grade class size as instrument, but actual class size is part of this instrument so it is probably not exogenous. |
| **Method for identifying relevant confounders described by researchers. Yes/No - if Yes describe the method used.** | According to the author: In six countries (Denmark, Germany, Iceland, Norway, Spain and Sweden), the respective maximum class-size rules seem to be implemented without many exceptions, so that predicted and actual average class sizes coincide very nicely. In an additional four countries (France, Greece, Ireland and Switzerland), there seem to be more exceptions to the rule (or more data recording errors in the TIMSS data), but there is still a reasonably jigsaw like pattern to warrant an implementation of the quasi-experimental identification strategy. In the remaining European countries and the United States, there does not seem to be a properly enforced maximum class-size rule at the national level on which such an identification strategy could be based. (p. 476). Continue with only the 10 countries, however, the first stage results (using actual class size) are too week and continues using grade average class size (which is used as an instrument in the first strategy). |  |  |  |
| **Relevant confounders described (See relevant sheet and list confounders and note if they were considered, precise, imbalanced or adjusted)** |  |  |  |  |
| **Method used for controlling for confounding (At design state)** | Two strategies: Grade average used as instrument and Maximum class size rule | Grade average used as instrument and school fixed effects |  |  |
| **Method used for controlling for confounding (At analysis stage)** |  |  |  |  |
| **Notes** |  | TIMMS data. | TIMMS data. | TIMMS data. |

### Risk of bias STAR studies

| Author | Achilles, 1993a | Achilles, 1993b | Balestra, 2014 |
| --- | --- | --- | --- |
| **Sequence generation (Judgement)** | Unclear | Unclear | Unclear |
| **Sequence generation (Description, quote from paper or describe key information)** | Both children and teachers were randomly allocated within schools but the method is not described. From correspondence with the external statistician who conducted the original analyses, Jeremy Finn (January 29, 2015): "there were 5 Universities involved in different parts of the state and each was responsible for its own randomization. (...) Several used tables of random numbers." According to the Technical report (Word, 1994) 4 universities were involved (p. 23) | See Achilles, 1993a | See Achilles, 1993a |
| **Allocation concealment (Judgement)** | Low | Low | Low |
| **Allocation concealment (Description, quote from paper or describe key information)** | Non-sequential allocation | Non-sequential allocation | Non-sequential allocation |
| **Blinding (Judgement)** | 4 | 4 | 4 |
| **Incomplete outcome data addressed (Judgement)** |  | 4. Grade 4 and 5. grade 5 | Kindergarten 1 and 1. Grade 2. 4 and 8 grade 4 and 3 |
| **Incomplete outcome data addressed (Description, quote from paper or describe key information)** |  | Report that the sample contained 4243/4649 students in grade 4/5 and according to the Database User's Guide (Finn et al., 2007) there are 6339/2593 students in 4/5 grade with achievement data and at least one year in STAR. According to STAR Technical report (Word, 1994) there was 6804/75 in the 4 year (3. grade). Thus of the 3. grade participants, missing data + attrition (cannot separate due to no info!) level is 38%/63% (not considering the attrition from beginning of experiment) | Attrition and missing data in total, Students/classes/schools: kindergarten: 9%/0%/0%; 1. grade: 4%/2%/4%. Concerning LBS data: the Database User's Guide (Finn et al., 2007) reports 6339/6361 in 4/8 grade with achievement data and at least one year in STAR. In this analysis they report using 4043/5056 students for 4/8 grade analysis, thus missing data 36%/21%. |
| **Free of selective reporting (Judgement)** |  | 1 | 4 |
| **Free of selective reporting (Description, quote from paper or describe key information)** |  |  | Show STAR results for kindergarten and 1 grade only. Report that 2 and 3 grade show similar patterns. 'For ease of comprehension' they only show LBS results for 4 and 8 grade |
| **Free of other bias (Judgement)** | 4 | 5 | 4 and 5 for the LBS results |
| **Free of other bias (Description, quote from paper or describe key information)** | Each year between 18 per cent and 32 per cent of the classes were out-of range (too small or too large). At the beginning of 1. Grade approx. half of the students in regular and regular with aide classes interchanged classes (see Finn et al., 2007). At the beginning of 2. Grade (3. Grade) 6 (5) per cent of the students in regular and regular with aide classes interchanged classes. In addition each year students from small classes moved to regular or regular-with-aide classes and students from regular and regular with aide classes moved to small classes (6, 4 and 4 per cent at the beginning of 1., 2. and 3. Grade). In total 25 per cent of all students moved class type at some point. | See Achilles, 1993a. Unclear how the students (25 per cent) who move class type were categorised in the LBS follow up. | See Achilles, 1993a. Unclear how the students (25 per cent) who move class type were categorised in the LBS follow up. |
| **A priori protocol (Judgement)** | No | No | No |
| **A priori protocol (Description, quote from paper or describe key information)** |  |  |  |
| **A priori analysis plan (Judgement)** | Unclear | Unclear | Unclear |
| **A priori analysis plan (Description, quote from paper or describe key information)** |  |  |  |
| **Notes** | STAR. Reproduction of the results in Word et al. 1990 and further results on various subgroups (for example entering STAR in grade 1 or results on retained/not retained etc.) |  | They perform an attrition analysis, testing if attrition is different on a number of covariates. They have 85 tests in total and 4 (=5%) is significant at a 5% level (which is what you would expect even if no real difference exists) |

| Author | Bingham, 1994 | Chetty, 2011 | Ding, 2011 |
| --- | --- | --- | --- |
| **Sequence generation (Judgement)** | Unclear | Unclear | Unclear |
| **Sequence generation (Description, quote from paper or describe key information)** | See Achilles, 1993a | See Achilles, 1993a | See Achilles, 1993a |
| **Allocation concealment (Judgement)** | Low | Low | Low |
| **Allocation concealment (Description, quote from paper or describe key information)** | Non-sequential allocation | Non-sequential allocation | Non-sequential allocation |
| **Blinding (Judgement)** |  | 4 | 4 |
| **Incomplete outcome data addressed (Judgement)** |  | 2 | 1 |
| **Incomplete outcome data addressed (Description, quote from paper or describe key information)** |  | Test score observations 9,939. Missing data 14% | Attrition and missing data in total, Students/classes/schools: kindergarten: 9%/0%/0%; |
| **Free of selective reporting (Judgement)** |  | 1 | 1 |
| **Free of selective reporting (Description, quote from paper or describe key information)** |  |  |  |
| **Free of other bias (Judgement)** |  | 1 | 1 |
| **Free of other bias (Description, quote from paper or describe key information)** |  |  |  |
| **A priori protocol (Judgement)** |  | No | No |
| **A priori protocol (Description, quote from paper or describe key information)** |  |  |  |
| **A priori analysis plan (Judgement)** |  | No | No |
| **A priori analysis plan (Description, quote from paper or describe key information)** |  |  |  |
| **Notes** | STAR reanalysis. No useful data provided (only means) | Test score as the average math and reading percentile rank score attained in the student’s year of entry into the experiment is only relevant outcome which cannot be used for this review | STAR reanalysis. Uses KG data only |

| Author | Ding, 2005 | Ding, 2010 | Doulgas, 1989 |
| --- | --- | --- | --- |
| **Sequence generation (Judgement)** | Unclear | Unclear | Unclear |
| **Sequence generation (Description, quote from paper or describe key information)** | See Achilles, 1993a | See Achilles, 1993a | See Achilles, 1993a |
| **Allocation concealment (Judgement)** | Low | Low | Low |
| **Allocation concealment (Description, quote from paper or describe key information)** | Non-sequential allocation | Non-sequential allocation | Non-sequential allocation |
| **Blinding (Judgement)** |  | 4 | 4 |
| **Incomplete outcome data addressed (Judgement)** |  | Kindergarten 1 and 1. Grade 3 and 2. and 3. grade 4 | Unclear |
| **Incomplete outcome data addressed (Description, quote from paper or describe key information)** |  | Only include students starting in kindergarten. Attrition per grade: kindergarten: 9%; 1. grade: 28%; 2 grade: 44%; 3 grade: 51% | Report using 841 classes (total of small and regular) |
| **Free of selective reporting (Judgement)** |  | 1 | 1 |
| **Free of selective reporting (Description, quote from paper or describe key information)** |  |  |  |
| **Free of other bias (Judgement)** |  | 1 | 5 |
| **Free of other bias (Description, quote from paper or describe key information)** |  |  | See Achilles, 1993a. Unclear how the students (25 per cent) who move class type were categorised in the LBS follow up. |
| **A priori protocol (Judgement)** |  | No | No |
| **A priori protocol (Description, quote from paper or describe key information)** |  |  |  |
| **A priori analysis plan (Judgement)** |  | No | No |
| **A priori analysis plan (Description, quote from paper or describe key information)** |  |  |  |
| **Notes** | STAR reanalysis. None of the analyses can be used |  | STAR reanalysis. None of the analyses can be used |

| Author | Finn, 1998 | Finn, 1999 | Finn, 1990a |
| --- | --- | --- | --- |
| **Sequence generation (Judgement)** | Unclear | Unclear | Unclear |
| **Sequence generation (Description, quote from paper or describe key information)** | See Achilles, 1993a | See Achilles, 1993a | See Achilles, 1993a |
| **Allocation concealment (Judgement)** | Low | Low | Low |
| **Allocation concealment (Description, quote from paper or describe key information)** | Non-sequential allocation | Non-sequential allocation | Non-sequential allocation |
| **Blinding (Judgement)** | 4 |  | 4 |
| **Incomplete outcome data addressed (Judgement)** | Kindergarten 1; 1. Grade 2; 2. and 3. grade 3 |  | 2 for the cross sectional analysis and 5 for the growth analysis |
| **Incomplete outcome data addressed (Description, quote from paper or describe key information)** | Attrition and missing data in total (from the Technical report (Word, 1994)), Students/classes/schools: kindergarten: 9%/0%/0%; 1. grade: 4%/2%/4%; 2. grade: 22%/20%/17%; 3. grade: 30%/21%/20%. Reduction in 2. grade schools and classes due to removal of 67 teachers who received Project Star training (it is 67 teachers according to the Technical report (Word, 1994) page 73 (text and table IV-12 providing the numbers used for analysis) but on page 117 and 192 and according to Word (1990) and Folger & Breda (1989) it was 57 teachers in grade 2 from 13 randomly chosen schools and another 57 teachers in grade 3 from the same schools). Further in 3. Grade two schools had incomplete test data and were removed. Compared to the number of classes in 2. Grade however, only the number of regular classes is reduced (with 11). New students were enrolled each year (and randomised) |  | Attrition and missing data in total, Students/classes/schools: kindergarten: 9%/0%/0%; 1. grade: 4%/2%/4%. New students were enrolled each year (and randomised) For this particular sample (1. grade) 347 classes with 7100 students were available but due to coding errors 4.6% of classes and 7.5% of students could not be used for analysis (These numbers differ from those reported in the Technical report (Word, 1994) p. 59). A growth analysis of students participating in the same classroom arrangement for both years and who had complete data (35%) performed |
| **Free of selective reporting (Judgement)** | 1 |  | 1 |
| **Free of selective reporting (Description, quote from paper or describe key information)** |  |  |  |
| **Free of other bias (Judgement)** | 4 |  | 4 |
| **Free of other bias (Description, quote from paper or describe key information)** | See Achilles, 1993a. |  | See Achilles, 1993a. |
| **A priori protocol (Judgement)** | No |  | No |
| **A priori protocol (Description, quote from paper or describe key information)** |  |  |  |
| **A priori analysis plan (Judgement)** | No |  | No |
| **A priori analysis plan (Description, quote from paper or describe key information)** |  |  |  |
| **Notes** |  | Reporting of ES from other studies (using STAR), calculate Grade Equivalence ESs (not an outcome of this review) and behaviour ESs |  |

| Author | Finn, 1990b | Finn, 2005 | Finn, 1989 |
| --- | --- | --- | --- |
| **Sequence generation (Judgement)** | Unclear | Unclear | Unclear |
| **Sequence generation (Description, quote from paper or describe key information)** | See Achilles, 1993a | See Achilles, 1993a | See Achilles, 1993a |
| **Allocation concealment (Judgement)** | Low | Low | Low |
| **Allocation concealment (Description, quote from paper or describe key information)** | Non-sequential allocation | Non-sequential allocation | Non-sequential allocation |
| **Blinding (Judgement)** | 4 | 4 | 4 |
| **Incomplete outcome data addressed (Judgement)** | 5 | 4 | 4 |
| **Incomplete outcome data addressed (Description, quote from paper or describe key information)** | Attrition and missing data in total (from the Technical report (Word, 1994)), Students/classes/schools: kindergarten: 9%/0%/0%; 1. grade: 4%/2%/4%; 2. grade: 22%/20%/17%. Reduction in 2. Grade schools and classes due to removal of 67 teachers who received Project Star training. New students were enrolled each year (and randomised). This study excludes the 67 teachers who received training. Based on total numbers available (from Technical report (Word, 1994) p. 15) percent (students) not used (because they were not in the same classes for three years, K-grade 2) is 69% | Attrition and missing data in total for the original trial, Students/classes/schools: kindergarten: 9%/0%/0%; 1. grade: 4%/2%/4%; 2. grade: 22%/20%/17%; 3. grade: 30%/21%/20%. Reduction in 2. Grade schools and classes due to removal of 67 teachers who received Project Star training and further in 3. Grade two schools had incomplete test data and were removed. New students were enrolled each year (and randomised). The present study uses data for treated students in the experiment at least one year, with high school scripts and with achievement data in grade K-3 (1/3 had missing data on some achievement variables, adequate imputation (described p. 217) was carried out); T/C attrition (not considering attrition from original trial) 55%/59%. Used sample compared to full sample on gender, minority and free lunch, somewhat lower percentage minority in used sample | Concerning LBS data: the Database User's Guide (Finn et al., 2007) reports 6339 in 4 grades with achievement data and at least one year in STAR. In this analysis they report using 4230 students, thus missing data 37% |
| **Free of selective reporting (Judgement)** |  | 1 | 1 |
| **Free of selective reporting (Description, quote from paper or describe key information)** |  |  |  |
| **Free of other bias (Judgement)** |  | 5 | 5 |
| **Free of other bias (Description, quote from paper or describe key information)** |  | See Achilles, 1993a. Unclear how the students (25 per cent) who move class type were categorised in the LBS follow up.  Some of the students move out of their school during K.-3. Grade. That happens both in full size classes and small size classes. Treated students are divided into 4 categories: students who attended small classes for 1, 2, 3, or 4 years, respectively, and compared to the full sample of controls (of which some also have moved out of their school). Transience rates (one or more school move) are very different for control and the 4 treated groups: C: 52%, T1: 74.9%, T2: 70.5%, T3: 20.7% and T4: 0.0%. Besides these differences in transience rates this division of treated 'destroys' the randomisation. In addition they enter two high school characteristics as 'independent' variables (bad controls, may be outcome and no consideration of the degree to which students are in same school, the original class mates may have been spread all over the country/district) | See Achilles, 1993a. Unclear how the students (25 per cent) who move class type were categorised in the LBS follow up. |
| **A priori protocol (Judgement)** | No | No | No |
| **A priori protocol (Description, quote from paper or describe key information)** |  |  |  |
| **A priori analysis plan (Judgement)** | Unclear | Unclear | Unclear |
| **A priori analysis plan (Description, quote from paper or describe key information)** |  |  |  |
| **Notes** |  | Logistic multilevel regression, analysis of high school graduation |  |

| Author | Finn, 2001 | Folger, 1989 | Hanushek, 1999 |
| --- | --- | --- | --- |
| **Sequence generation (Judgement)** | Unclear | Unclear | Unclear |
| **Sequence generation (Description, quote from paper or describe key information)** | See Achilles, 1993a | See Achilles, 1993a | See Achilles, 1993a |
| **Allocation concealment (Judgement)** | Low | Low | Low |
| **Allocation concealment (Description, quote from paper or describe key information)** | Non-sequential allocation | Non-sequential allocation | Non-sequential allocation |
| **Blinding (Judgement)** | 4 | 4 | 4 |
| **Incomplete outcome data addressed (Judgement)** | Kindergarten-3 grade 2; 4. Grade 4; 6. Grade 2 and 5; 8. grade 2 and 3 | 3 and all 4 years student-analysis 5 | Kindergarten 1; 1 grade 2; 2 and 3 grade 3 |
| **Incomplete outcome data addressed (Description, quote from paper or describe key information)** | Attrition and missing data level varies around 13% for K-3 grade (they do not report the numbers used per grade, only the numbers per grade used varies between 5394 and 5910 students). Concerning LBS data: the Database User's Guide (Finn et al., 2007) reports 6339/6441/6361 in 4/6/8 grade with achievement data and at least one year in STAR. In this analysis they report using 4015/6100/5835 students for 4/6/8 grade analysis of CTBS, thus missing data 37%/5%/8%; for BSF: 36%/58%/18% | Attrition and missing data in total (from the Technical report (Word, 1994)), Students/classes/schools: kindergarten: 9%/0%/0%; 1. grade: 4%/2%/4%; 2. grade: 22%/20%/17%; 3. grade: 30%/21%/20%. Reduction in 2. Grade schools and classes due to removal of 67 teachers who received Project Star training and further in 3. Grade two schools had incomplete test data and were removed. New students were enrolled each year (and randomised). This study does not exclude the 67 teachers who received training but exclude out-of-range classes. Based on total numbers available (from Technical report (Word, 1994) p. 15) percent (students) not used are: K: 19%, 1: 19%, 2: 20% and 3: 23%. Analysis of students in project all 4 years uses approx. 30% of all students | No mentioning of attrition. Missing data level around 7-8% for kindergarten; 3-6% for grade 1; 11% for grade 2 and 3 |
| **Free of selective reporting (Judgement)** | 3 | 1 | 1 |
| **Free of selective reporting (Description, quote from paper or describe key information)** | Do not analyse grade 5 and 7 (with no explanation why) |  |  |
| **Free of other bias (Judgement)** | 4 and 5 for the LBS results | 4 | 4 |
| **Free of other bias (Description, quote from paper or describe key information)** | See Achilles, 1993a. Unclear how the students (25 per cent) who move class type were categorised in the LBS follow up. | See Achilles, 1993a. Exclude out-of range classes | See Achilles, 1993a. |
| **A priori protocol (Judgement)** | No | No | No |
| **A priori protocol (Description, quote from paper or describe key information)** |  |  |  |
| **A priori analysis plan (Judgement)** | Unclear | No | No |
| **A priori analysis plan (Description, quote from paper or describe key information)** |  |  |  |
| **Notes** | Age, grade level, performance at base-line, gender, socio-economic background local education spending are included |  |  |

| Author | Harvey, 1994 | Jackson, 2013 | Jacobs, 1987 |
| --- | --- | --- | --- |
| **Sequence generation (Judgement)** | High | Unclear | Unclear |
| **Sequence generation (Description, quote from paper or describe key information)** | Subgroup from a RCT analysed. Subgroup is retainees, i.e. selected on a potential outcome variable. | See Achilles, 1993a | See Achilles, 1993a |
| **Allocation concealment (Judgement)** | High | Low | Low |
| **Allocation concealment (Description, quote from paper or describe key information)** |  | Non-sequential allocation | Non-sequential allocation |
| **Blinding (Judgement)** | 4 | 4 |  |
| **Incomplete outcome data addressed (Judgement)** | Kindergarten/1. Grade unclear, 1. Grade/2. Grade 3 and 2. Grade/3. Grade 4 and 3. grade 5 | Kindergarten 1 and 1. Grade 2. |  |
| **Incomplete outcome data addressed (Description, quote from paper or describe key information)** | Kindergarten retainees: Kindergarten not reported. Number of students falls gradually by 35%, 48% and 67% from 1 to 3 grade. 1. Grade retainees: 1. grade not reported. Number of students falls gradually by 29% and 48% from 2 to 3 grade. | Attrition and missing data in total, Students/classes/schools: kindergarten: 9%/0%/0%; 1. grade: 4%/2%/4%. |  |
| **Free of selective reporting (Judgement)** |  | 3 |  |
| **Free of selective reporting (Description, quote from paper or describe key information)** |  | Conduct quantile regression but only report results in figures |  |
| **Free of other bias (Judgement)** | 5 | 2 |  |
| **Free of other bias (Description, quote from paper or describe key information)** | See Achilles, 1993a. No confounders related to class size considered, only related to overall retainees/non-retainees | See Achilles, 1993a. Argue that approximately 10% of the students switched class-types and they argue that movement between class-types may be correlated with student achievement. Therefore they base the analysis on students’ initial assignment and focus on early grades, in order to increase the correspondence between intent-to-treat and treatment-on-the-treated effects. They also argue that as previous studies have found that the treatment effects associated with the experiment are most strongly associated with the students’ environment during the first two years of school, the cleanest distributional analysis is one that uses data from kindergarten and first grade. |  |
| **A priori protocol (Judgement)** | No | No |  |
| **A priori protocol (Description, quote from paper or describe key information)** |  |  |  |
| **A priori analysis plan (Judgement)** | Unclear | Unclear |  |
| **A priori analysis plan (Description, quote from paper or describe key information)** |  |  |  |
| **Notes** |  |  | No useful data: Results in table 3, 4 and 5 (for three different outcomes) have main effect for class type (not small separated out). Cross tabulation of the 3 outcomes in table 6, 7 and 8 but only raw totals and percent scoring low/middle/high and other tables subdivided on several covariates |

| Author | Konstantopoulos, 2008 | Konstantopoulos, 2011 | Konstantopoulos, 2009 |
| --- | --- | --- | --- |
| **Sequence generation (Judgement)** | Unclear | Unclear | Unclear |
| **Sequence generation (Description, quote from paper or describe key information)** | See Achilles, 1993a | See Achilles, 1993a | See Achilles, 1993a |
| **Allocation concealment (Judgement)** | Low | Low | Low |
| **Allocation concealment (Description, quote from paper or describe key information)** | Non-sequential allocation | Non-sequential allocation | Non-sequential allocation |
| **Blinding (Judgement)** | 4 | 4 | 4 |
| **Incomplete outcome data addressed (Judgement)** | Kindergarten 1 and 1. Grade 2 and 2. and 3. grade 3 | Kindergarten 1 and 1. Grade 2 and 2. and 3. grade 3 | Unclear |
| **Incomplete outcome data addressed (Description, quote from paper or describe key information)** | Attrition and missing data in total, Students/classes/schools: kindergarten: 9%/0%/0%; 1. grade: 4%/2%/4%; 2. grade: 22%/20%/17%; 3. grade: 30%/21%/20%. Reduction in 2. Grade schools and classes due to removal of 67 teachers who received Project Star training and further in 3. Grade two schools had incomplete test data and were removed. New students were enrolled each year (and randomised) | Attrition and missing data in total (from STAR), Students/classes/schools: kindergarten: 9%/0%/0%; 1. grade: 4%/2%/4%; 2. grade: 22%/20%/17%; 3. grade: 30%/21%/20%. Reduction in 2. Grade schools and classes due to removal of 67 teachers who received Project Star training and further in 3. Grade two schools had incomplete test data and were removed. New students were enrolled each year (and randomised). Do not report number of students used, only number of schools. Analyse students as randomised (ITT for those with no missing data, do not impute) | Attrition and missing data in total (from STAR), Students/classes/schools: kindergarten: 9%/0%/0%; 1. grade: 4%/2%/4%; 2. grade: 22%/20%/17%; 3. grade: 30%/21%/20%. Reduction in 2. Grade schools and classes due to removal of 67 teachers who received Project Star training and further in 3. Grade two schools had incomplete test data and were removed. New students were enrolled each year (and randomised). First analysis use FU data to 3. grade treatment (as do second analysis but in a different way), number of students with missing data at FU 4, 5, 6, 7 and 8 grade apparently varies a lot and nothing is reported concerning number of students available each year. From technical report for 5. Grade (Nye et al., 1992) there were 4230/4649 students in 4/5 grade who had participated in STAR at least for 3. Grade, whereas the Database User's Guide (Finn et al., 2007) reports 6339/2593/6441/4942/6361 in 4/5/6/7/8 grade with achievement data. According to STAR Technical report (Word, 1994) there was 6804/75 in the 4 year (3. grade). Thus of the 3. grade participants, missing data + attrition (cannot separate due to no info!) level is: 37/63/49/43/35% in grade 4/5/6/7/8 (not considering the attrition from beginning of experiment) |
| **Free of selective reporting (Judgement)** | 1 | 4 | 1 |
| **Free of selective reporting (Description, quote from paper or describe key information)** |  | Within school effects are found using regression controlled for gender, race, and SES effects. Do not report these results. Do not fully report the results from the mixed effect model (only mentioned in WP). The journal article do not mention the mixed effect model |  |
| **Free of other bias (Judgement)** | 4 | 4 | 5 |
| **Free of other bias (Description, quote from paper or describe key information)** | See Achilles, 1993a. | See Achilles, 1993a. ITT analysis. Within school effects using STAR data. Many schools did not have more than one class in one or all of the three conditions implying that within school effects cannot be separated from class effect (teacher and other) in a model where small classes and regular with aide classes are compared to regular classes (approximately 43% of schools had only one small class and 81% had only one small and/or one regular class)) Although the average mean from a random effect meta-analysis is reported it is a weighted average of ESs where a large part of them (81%) do not identify a class size effect even though the teachers were also randomised within schools. Using a random effect model it is assumed that each school's ES represents an estimate of its own population effect. | See Achilles, 1993a. Unclear how the students (25 per cent) who move class type were categorised in the LBS follow up Their second analysis is about cumulative effects (actually it is dose: 4 vs 0, 4 vs 1, 4 vs 2 and 4 vs 3). Do not report the numbers with 0,1,2,3 and 4 years of treatment but from other analyses of this kind it is known that the number with 4 years of treatment is very small). Both analyses uses quantile regression but it is not reported in what year(s) the distribution is taken from. It is probably the 3. grade distribution (but may be the last years distribution) where students had spent 0-4 years in treatment |
| **A priori protocol (Judgement)** | No | No | No |
| **A priori protocol (Description, quote from paper or describe key information)** |  |  |  |
| **A priori analysis plan (Judgement)** | No | No | No |
| **A priori analysis plan (Description, quote from paper or describe key information)** |  |  |  |
| **Notes** |  |  | Stanford Achievement test is what they write but Technical reports and Database User's Guide report that the Tennessee Comprehensive Assessment Program (TCAP, Comprehensive Tests of Basic Skills (CTBS/McGraw Hill, 1989) and Basic Skills First (BSF) criterion-referenced tests) was used for the Lasting Benefits Study (grade 4-8) |

| Author | Krueger, 2001a | Krueger, 2001b | Krueger, 1999 |
| --- | --- | --- | --- |
| **Sequence generation (Judgement)** | Unclear | Unclear | Unclear |
| **Sequence generation (Description, quote from paper or describe key information)** | See Achilles, 1993a | See Achilles, 1993a | See Achilles, 1993a |
| **Allocation concealment (Judgement)** | Low | Low | Low |
| **Allocation concealment (Description, quote from paper or describe key information)** | Non-sequential allocation | Non-sequential allocation | Non-sequential allocation |
| **Blinding (Judgement)** | 4 | 4 | 4 |
| **Incomplete outcome data addressed (Judgement)** | 1 | Unclear | Kindergarten 1 and 1. Grade 2 and 2. and 3. grade 3 |
| **Incomplete outcome data addressed (Description, quote from paper or describe key information)** | Use two samples: all STAR students 11600 and the subsample that were not behind grade 9397. Missing data levels: 3% and 3% | Updated sample (of the one used in Krueger & Whitmore, 2001) so an additional 10.7 percent of previously unmatched students were matched, bringing the rate of unmatched students down from 19% to 17%. Do not report the numbers used for analysis of taking exam (whole sample or subsample of those matched) and do not report level of missing data | Attrition and missing data in total, Students/classes/schools: kindergarten: 9%/0%/0%; 1. grade: 4%/2%/4%; 2. grade: 22%/20%/17%; 3. grade: 30%/21%/20%. Reduction in 2. Grade schools and classes due to removal of 67 teachers who received Project Star training and further in 3. Grade two schools had incomplete test data and were removed. They do not report the actual numbers they use for analysis, only the total numbers available which is quite different from the numbers with useful data (see numeric outcome). Several models estimated, among others a model using imputed outcomes (most recent test percentile) |
| **Free of selective reporting (Judgement)** | 1 | 1 | 1 |
| **Free of selective reporting (Description, quote from paper or describe key information)** |  |  |  |
| **Free of other bias (Judgement)** | 5 | 5 | 4 and 3 for the ITT model |
| **Free of other bias (Description, quote from paper or describe key information)** | See Achilles, 1993a. Unclear how the students (25 per cent) who move class type were categorised in the LBS follow up. Analysis of scores on the two entrance exams. Attempt to correct for selection *and* treatment bias (p. 22-23) | See Achilles, 1993a. Unclear how the students (25 per cent) who move class type were categorised in the LBS follow up. Analysis of scores on the two entrance exams. Attempt to correct for selection *and* treatment bias as in Krueger & Whitmore, 2001 | See Achilles, 1993a.. In this paper they also report results of an ITT model, a model using assignment as instrument and a model with imputed values |
| **A priori protocol (Judgement)** | No | No | No |
| **A priori protocol (Description, quote from paper or describe key information)** |  |  |  |
| **A priori analysis plan (Judgement)** | Unclear | Unclear | Unclear |
| **A priori analysis plan (Description, quote from paper or describe key information)** |  |  |  |
| **Notes** |  |  |  |

| Author | McKee, 2015 | Mckee, 2010 | Mosteller, 1995 |
| --- | --- | --- | --- |
| **Sequence generation (Judgement)** | Unclear | Unclear |  |
| **Sequence generation (Description, quote from paper or describe key information)** | See Achilles, 1993a | See Achilles, 1993a | See Achilles, 1993a |
| **Allocation concealment (Judgement)** | Low | Low | Low |
| **Allocation concealment (Description, quote from paper or describe key information)** | Non-sequential allocation | Non-sequential allocation | Non-sequential allocation |
| **Blinding (Judgement)** | 4 | 4 | 4 |
| **Incomplete outcome data addressed (Judgement)** | 1 | 1 |  |
| **Incomplete outcome data addressed (Description, quote from paper or describe key information)** | Attrition and missing data in total, Students/classes/schools: kindergarten: 9%/0%/0%; (from Technical report (Word, 1994)) | Attrition and missing data in total, Students/classes/schools: kindergarten: 9%/0%/0%; (from Technical report (Word, 1994)) |  |
| **Free of selective reporting (Judgement)** | 1 | 1 |  |
| **Free of selective reporting (Description, quote from paper or describe key information)** |  |  |  |
| **Free of other bias (Judgement)** | 1 | 1 |  |
| **Free of other bias (Description, quote from paper or describe key information)** |  |  |  |
| **A priori protocol (Judgement)** | No | No |  |
| **A priori protocol (Description, quote from paper or describe key information)** |  |  |  |
| **A priori analysis plan (Judgement)** | No | No |  |
| **A priori analysis plan (Description, quote from paper or describe key information)** |  |  |  |
| **Notes** | STAR reanalysis. Use only KG and pool R and RA classes and transform test scores to have zero mean and SD of one and include covariates | STAR reanalysis. Use only KG and merge R and RA. OLS w/wo school FE controlling for teachers with fewer than three years of experience and teachers with an advanced degree, and for the student's race-ethnicity, gender, age, special education status, whether or not they are repeating kindergarten, attendance record, and subsidized lunch eligibility. Specifications that do not include school fixed effects also include indicators for community type (suburban, rural, urban, and inner-city). | STAR Provides results from other articles: Finn, J.D., and Achilles, C.M. Answers and questions about class size: A state-wide experiment. American Educational Research Journal (1990) 27,3:557–77, Table 5. and Word, E., Johnston, J., Bain, H.P., et al. Student/Teacher Achievement Ratio (STAR): Tennessee’s K-3 class size study, Nashville: Tennessee Department of Education, Figures 1 and 2. |

| Author | Nye, 1992 | Nye, 1993 | Nye, 1992/1994 |
| --- | --- | --- | --- |
| **Sequence generation (Judgement)** | Unclear | Unclear | Unclear |
| **Sequence generation (Description, quote from paper or describe key information)** | See Achilles, 1993a | See Achilles, 1993a | See Achilles, 1993a |
| **Allocation concealment (Judgement)** | Low | Low | Low |
| **Allocation concealment (Description, quote from paper or describe key information)** | Non-sequential allocation | Non-sequential allocation | Non-sequential allocation |
| **Blinding (Judgement)** | 4 | 4 | 4 |
| **Incomplete outcome data addressed (Judgement)** | 5 | Kindergarten and 1 grade 2; 2 and 3 grade 3; 4. Grade 4 and 5. grade 5 | Kindergarten and 1 grade 2; 2 and 3 grade 3; 4. Grade 4 and 5. grade 5 |
| **Incomplete outcome data addressed (Description, quote from paper or describe key information)** | Report that the sample contained 4649 students and according to the Database User's Guide (Finn et al., 2007) there are 2593 students in 5 grade with achievement data and at least one year in STAR. According to STAR Technical report (Word, 1994) there was 6804/75 in the 4 year (3. grade). Thus of the 3. grade participants, missing data + attrition (cannot separate due to no info!) level is 63% (not considering the attrition from beginning of experiment) | Attrition and missing data level varies around 13% for KG and 1 grade (they do not report the numbers used in grade 2 and 3 grade (but report the trained teachers are not included), only the numbers per grade used is 5734 in KG and 5905 in 1. grade). Report that the sample contained 4320 students through grade 4 and 5 and according to the Database User's Guide (Finn et al., 2007) there are 6339/2593 students in 4/5 grade with achievement data and at least one year in STAR. According to STAR Technical report (Word, 1994) there was 6804/75 in the 4 year (3. grade). Thus of the 3. grade participants, missing data + attrition (cannot separate due to no info!) level is 38%/63% (not considering the attrition from beginning of experiment) | Attrition and missing data level varies around 13% for KG and 1 grade (they do not report the numbers used in grade 2 and 3 grade (but report the trained teachers are not included), only the numbers per grade used is 5734 in KG and 5905 in 1. grade). Report that the sample contained 4230/4649 students in grade 4/5 and according to the Database User's Guide (Finn et al., 2007) there are 6339/2593 students in 4/5 grade with achievement data and at least one year in STAR. According to STAR Technical report (Word, 1994) there was 6804/75 in the 4 year (3. grade). Thus of the 3. grade participants, missing data + attrition (cannot separate due to no info!) level is 38%/63% (not considering the attrition from beginning of experiment) |
| **Free of selective reporting (Judgement)** | 1 | 1 | 1 |
| **Free of selective reporting (Description, quote from paper or describe key information)** |  |  |  |
| **Free of other bias (Judgement)** | 5 | 4 and 5 for the LBS results | 4 and 5 for the LBS results |
| **Free of other bias (Description, quote from paper or describe key information)** | See Achilles, 1993a. Unclear how the students (25 per cent) who move class type were categorised in the LBS follow up. | See Achilles, 1993a. Unclear how the students (25 per cent) who move class type were categorised in the LBS follow up. | See Achilles, 1993a. Unclear how the students (25 per cent) who move class type were categorised in the LBS follow up. |
| **A priori protocol (Judgement)** | Unclear | Unclear | Unclear |
| **A priori protocol (Description, quote from paper or describe key information)** |  |  |  |
| **A priori analysis plan (Judgement)** | Unclear | Unclear | Unclear |
| **A priori analysis plan (Description, quote from paper or describe key information)** |  |  |  |
| **Notes** |  | Exactly same analysis and results as in Nye, 1992/1994 |  |

| Author | Nye, 2000b | Nye, 2000a | Nye, 2002 |
| --- | --- | --- | --- |
| **Sequence generation (Judgement)** | Unclear | Unclear | Unclear |
| **Sequence generation (Description, quote from paper or describe key information)** | See Achilles, 1993a | See Achilles, 1993a | See Achilles, 1993a |
| **Allocation concealment (Judgement)** | Low | Low | Low |
| **Allocation concealment (Description, quote from paper or describe key information)** | Non-sequential allocation | Non-sequential allocation | Non-sequential allocation |
| **Blinding (Judgement)** | 4 | 4 | 4 |
| **Incomplete outcome data addressed (Judgement)** | Kindergarten 1 and 1. Grade 2 and 2. and 3. grade 3 | Kindergarten 1 and 1. Grade 2 and 2. and 3. grade 3 | Unclear |
| **Incomplete outcome data addressed (Description, quote from paper or describe key information)** | Attrition and missing data in total, Students/classes/schools: kindergarten: 9%/0%/0%; 1. grade: 4%/2%/4%; 2. grade: 22%/20%/17%; 3. grade: 30%/21%/20%. Reduction in 2. Grade schools and classes due to removal of 67 teachers who received Project Star training and further in 3. Grade two schools had incomplete test data and were removed. They do not report the actual numbers they use for analysis, only the total numbers available which is quite different from the numbers with useful data (see numeric outcome) | Attrition and missing data in total, Students/classes/schools: kindergarten: 9%/0%/0%; 1. grade: 4%/2%/4%; 2. grade: 22%/20%/17%; 3. grade: 30%/21%/20%. Reduction in 2. Grade schools and classes due to removal of 67 teachers who received Project Star training and further in 3. Grade two schools had incomplete test data and were removed. They do not report the actual numbers they use for analysis, only the total numbers available which is quite different from the numbers with useful data (see numeric outcome) | Nothing reported |
| **Free of selective reporting (Judgement)** | 2 | 2 | 2 |
| **Free of selective reporting (Description, quote from paper or describe key information)** | Estimated several models (two level model and three level model for the actual assignment and a three level model for ITT). Do not explain why they do not estimate a two level model for the ITT analysis | Estimated several models using various combinations of gender, SES, minority status and combinations of these variables. Report results for only one of them and state that results were similar. Considering school level they estimate models with random effects and state that the results with all (seven) coefficients random and those presented were not qualitatively different (present only results for one model where only significant variance components not constrained to be 0) | Estimated several models using various combinations of gender, SES, minority status and combinations of these variables. Report results for only one of them and state that results were similar. Considering school level they estimate models with random effects and state that the results with all (seven) coefficients random and those presented were not qualitatively different (present only results for one model where only significant variance components not constrained to be 0) |
| **Free of other bias (Judgement)** | 4 and 3 for the ITT model | 4 | 4 |
| **Free of other bias (Description, quote from paper or describe key information)** | See Achilles 1993a. In this paper they report results of an ITT model but the writing up of the models, the text and the coefficient labels do not match. | See Achilles 1993a. | See Achilles 1993a. |
| **A priori protocol (Judgement)** | No | No | No |
| **A priori protocol (Description, quote from paper or describe key information)** |  |  |  |
| **A priori analysis plan (Judgement)** | No | No | No |
| **A priori analysis plan (Description, quote from paper or describe key information)** |  |  |  |
| **Notes** | Small class interaction terms included | Small class interaction terms included | Small class interaction terms included |

| Author | Nye, 2001a | Nye, 2001b | Prais, 1996 |
| --- | --- | --- | --- |
| **Sequence generation (Judgement)** | Unclear | Unclear | Unclear |
| **Sequence generation (Description, quote from paper or describe key information)** | See Achilles, 1993a | See Achilles, 1993a | See Achilles, 1993a |
| **Allocation concealment (Judgement)** | Low | Low | Low |
| **Allocation concealment (Description, quote from paper or describe key information)** | Non-sequential allocation | Non-sequential allocation | Non-sequential allocation |
| **Blinding (Judgement)** | 4 | 4 | 4 |
| **Incomplete outcome data addressed (Judgement)** | 5 | 1. Grade 4 and 2. Grade 5 and 3. grade 5 | Kindergarten 1 and 1. Grade 2 and 2. and 3. grade 3 |
| **Incomplete outcome data addressed (Description, quote from paper or describe key information)** | Attrition and missing data in total for the original trial, Students/classes/schools: kindergarten: 9%/0%/0%; 1. grade: 4%/2%/4%; 2. grade: 22%/20%/17%; 3. grade: 30%/21%/20%. Reduction in 2. Grade schools and classes due to removal of 67 teachers who received Project Star training and further in 3. Grade two schools had incomplete test data and were removed. New students were enrolled each year (and randomised). Analysis 1) Students who participated at least 1 year and was part of the trial in 3. grade: T/C attrition (not considering attrition from original trial) 61%/58% 2) students participating all 4 years: T/C attrition (not considering attrition from original trial) 63%/59% | Attrition and missing data in total for the original trial, Students/classes/schools: kindergarten: 9%/0%/0%; 1. grade: 4%/2%/4%; 2. grade: 22%/20%/17%; 3. grade: 30%/21%/20%. Reduction in 2. Grade schools and classes due to removal of 67 teachers who received Project Star training and further in 3. Grade two schools had incomplete test data and were removed. New students were enrolled each year (and randomised). Analysis 1) Students who participated in grade K and 1 and had data for both years and further only treated who were treated both years: T/Total (not control here because unsure if they include as controls those who had small class in some years) attrition (as % of the number used in the original analysis, i.e. the nominator is excl. the 'original' attrition and missing data) 26%/36% 2) Students who participated in grade 1 and 2 and had data for both years and further only treated who were treated all three years: T/Total (not control here because unsure if they include as controls those who had small class in some years) attrition (as % of the number used in the original analysis, i.e. incl. the 'original' attrition and missing data) 33%/13% 3) Students who participated in grade 2 and 3 and had data for both years and further only treated who were treated all four years: T/Total (not control here because unsure if they include as controls those who had small class in some years) attrition (as % of the number used in the original analysis, i.e. incl. the 'original' attrition and missing data) 46%/1% | Attrition and missing data in total, Students/classes/schools: kindergarten: 9%/0%/0%; 1. grade: 4%/2%/4%; 2. grade: 22%/20%/17%; 3. grade: 30%/21%/20%. Reduction in 2. Grade schools and classes due to removal of 67 teachers who received Project Star training and further in 3. Grade two schools had incomplete test data and were removed. New students were enrolled each year (and randomised) |
| **Free of selective reporting (Judgement)** |  | 1 | 1 |
| **Free of selective reporting (Description, quote from paper or describe key information)** |  |  |  |
| **Free of other bias (Judgement)** | 5 | 4 | 4 |
| **Free of other bias (Description, quote from paper or describe key information)** | See Achilles, 1993a. Unclear how the students (25 per cent) who move class type were categorised in the LBS follow up. | See Achilles 1993a. In addition it is unclear whether some of the students in the control group they use have spent some years in a small class (the control group is characterised by: small class in some or no grades, see table 1). In the analysis for each grade they include only treated who were in small class for that grade and all previous grades. Unclear whether the control group is required to have been in the experiment for all previous grades but probably not, the total sample size increases from grade 1 to 3 whereas the treated group considerably decreases | See Achilles 1993a. |
| **A priori protocol (Judgement)** | No | No | No |
| **A priori protocol (Description, quote from paper or describe key information)** |  |  |  |
| **A priori analysis plan (Judgement)** | No | No | No |
| **A priori analysis plan (Description, quote from paper or describe key information)** |  |  |  |
| **Notes** |  |  | The paper argues (p 404) that the movement of students from small classes in grade 1 is sufficient to eliminate the math gain in small class over average class size |

| Author | Schanzenbach, 2007 | Shin, 2012 | Shin, 2011 |
| --- | --- | --- | --- |
| **Sequence generation (Judgement)** | Unclear | Unclear | Unclear |
| **Sequence generation (Description, quote from paper or describe key information)** | See Achilles, 1993a | See Achilles, 1993a | See Achilles, 1993a |
| **Allocation concealment (Judgement)** | Low | Low | Low |
| **Allocation concealment (Description, quote from paper or describe key information)** | Non-sequential allocation | Non-sequential allocation | Non-sequential allocation |
| **Blinding (Judgement)** | 4 | 4 | 4 |
| **Incomplete outcome data addressed (Judgement)** | Kindergarten 1 and 1. and 2. Grade 2 and 3. Grade 3. Grade 4-8 Unclear | 1 | 1 |
| **Incomplete outcome data addressed (Description, quote from paper or describe key information)** | Attrition and missing data in total (from the Technical report (Word, 1994)), Students/classes/schools: kindergarten: 9%/0%/0%; 1. grade: 4%/2%/4%; 2. grade: 22%/20%/17%; 3. grade: 30%/21%/20%. Reduction in 2. Grade schools and classes due to removal of 67 teachers who received Project Star training and further in 3. Grade two schools had incomplete test data and were removed. New students were enrolled each year (and randomised). This study does not exclude the 67 teachers who received training. Numbers used for grade 4-8 not reported. From technical report for 5. Grade (Nye et al., 1992) there were 4230/4649 students in 4/5 grade who had participated in STAR at least for 3. Grade, whereas the Database User's Guide (Finn et al., 2007) reports 6339/2593/6441/4942/6361 in 4/5/6/7/8 grade with achievement data. According to STAR Technical report (Word, 1994) there was 6804/75 in the 4 year (3. grade). Thus of the 3. grade participants, missing data + attrition (cannot separate due to no info!) level is: 37/63/49/43/35% in grade 4/5/6/7/8 (not considering the attrition from beginning of experiment) | Attrition and missing data in total, Students/classes/schools: kindergarten: 9%/0%/0%; 1. grade: 4%/2%/4%; 2. grade: 22%/20%/17%; 3. grade: 30%/21%/20%. Reduction in 2. Grade schools and classes due to removal of 67 teachers who received Project Star training and further in 3. Grade two schools had incomplete test data and were removed. New students were enrolled each year (and randomised). Use only new comers each year and impute missing data | Attrition and missing data in total, Students/classes/schools: kindergarten: 9%/0%/0%; 1. grade: 4%/2%/4%; 2. grade: 22%/20%/17%; 3. grade: 30%/21%/20%. Reduction in 2. Grade schools and classes due to removal of 67 teachers who received Project Star training and further in 3. Grade two schools had incomplete test data and were removed. New students were enrolled each year (and randomised). Use only new comers each year and impute missing data |
| **Free of selective reporting (Judgement)** | 1 | 1 | 1 |
| **Free of selective reporting (Description, quote from paper or describe key information)** |  |  |  |
| **Free of other bias (Judgement)** | 3 | 3 | 3 |
| **Free of other bias (Description, quote from paper or describe key information)** | See Achilles, 1993a. Estimate the effect of being assigned to a small class | Estimate the effect of being assigned to a small (using an IV approach) class using new comers only | Estimate the effect of being assigned to a small (using an IV approach) class using new comers only |
| **A priori protocol (Judgement)** | No | No | No |
| **A priori protocol (Description, quote from paper or describe key information)** |  |  |  |
| **A priori analysis plan (Judgement)** | No | No | No |
| **A priori analysis plan (Description, quote from paper or describe key information)** |  |  |  |
| **Notes** | ITT reanalysis using composite math and reading. Composite score cannot be used in data synthesis. Also provide results for composite test score for 4, 5, 6, 7 and 8 grade. | They investigate whether there is school-level confounding, by comparing a model with school-level fixed-effects to a model without fixed-effects (comparison of 3L ITT and 2L ITT in table 2 and 3). Analyses new comers only (The sample data consist of 6,325, 2,314, 1,679, and 1,283 new students) | They investigate whether there is school-level confounding, by comparing a model with school-level fixed-effects to a model without fixed-effects (comparison of 3L ITT and 2L ITT in table 4 and 5). Analyses new comers only (The sample data consist of 6,325, 2,314, 1,679, and 1,283 new students) |

| Author | Sohn,2015 | Word, 1990 | Word 1994 |
| --- | --- | --- | --- |
| **Sequence generation (Judgement)** | Unclear | Unclear | Unclear |
| **Sequence generation (Description, quote from paper or describe key information)** | See Achilles, 1993a | See Achilles, 1993a | See Achilles, 1993a |
| **Allocation concealment (Judgement)** | Low | Low | Low |
| **Allocation concealment (Description, quote from paper or describe key information)** | Non-sequential allocation | Non-sequential allocation | Non-sequential allocation |
| **Blinding (Judgement)** | 4 | 3 | 3 |
| **Incomplete outcome data addressed (Judgement)** | 4. Grade 4; 6. Grade 3; 8. grade 3 | Kindergarten 1 and 1. Grade 2 and 2. and 3. grade 3 and 5 for longitudinal | Kindergarten 1 and 1. Grade 2 and 2. and 3. grade 3 and 5 for longitudinal |
| **Incomplete outcome data addressed (Description, quote from paper or describe key information)** | Concerning LBS data: the Database User's Guide (Finn et al., 2007) reports 6339/6441/6361 in 4/6/8 grade with achievement data and at least one year in STAR. In this analysis they report using 3974/5942/5733 students for 4/6/8 grade analysis of CTBS, thus missing data 37%/8%/10%; | Attrition and missing data in total, Students/classes/schools: kindergarten: 9%/0%/0%; 1. grade: 4%/2%/4%; 2. grade: 22%/20%/17%; 3. grade: 30%/21%/20%. Reduction in 2. Grade schools and classes due to removal of 67 teachers who received Project Star training and further in 3. Grade two schools had incomplete test data and were removed. New students were enrolled each year (and randomised). The longitudinal analysis excludes new comers for the K-3 and K-1 analysis. Three longitudinal analyses where students had to be in the same class for the relevant years: K-3, K-1 and 1-3. Approx. 6800 students available each year, using this as basis the level of data used for longitudinal analysis is: 27% for K-3, 36% for K-1 and 38% for 1-3 | Attrition and missing data in total, Students/classes/schools: kindergarten: 9%/0%/0%; 1. grade: 4%/2%/4%; 2. grade: 22%/20%/17%; 3. grade: 30%/21%/20%. Reduction in 2. Grade schools and classes due to removal of 67 teachers who received Project Star training and further in 3. Grade two schools had incomplete test data and were removed. New students were enrolled each year (and randomised). The longitudinal analysis excludes new comers for the K-3 and K-1 analysis. Three longitudinal analyses where students had to be in the same class for the relevant years: K-3, K-1 and 1-3. Approx. 6800 students available each year, using this as basis the level of data used for longitudinal analysis is: 27% for K-3, 36% for K-1 and 38% for 1-3 |
| **Free of selective reporting (Judgement)** | 3 | 1 | 1 |
| **Free of selective reporting (Description, quote from paper or describe key information)** | Do not analyse grade 5 and 7 (with no explanation why) |  |  |
| **Free of other bias (Judgement)** | 5 | 4 | 4 |
| **Free of other bias (Description, quote from paper or describe key information)** | See Achilles 1993a. Unclear how the students (25 per cent) who move class type were categorised in the LBS follow up. | See Achilles 1993a. | See Achilles 1993a. |
| **A priori protocol (Judgement)** | No | Yes | Yes |
| **A priori protocol (Description, quote from paper or describe key information)** |  | Yes, for the original project | Yes, for the original project |
| **A priori analysis plan (Judgement)** | No | Yes | Yes |
| **A priori analysis plan (Description, quote from paper or describe key information)** |  | Yes, for the original analyses which are reproduced here | Yes, for the original analyses which are reproduced here |
| **Notes** | Analyse number of years in small class and divide on 'effective' (i.e. significant difference) and ineffective schools (also show total). Results cannot be used | Final summary of original results. Provide effect sizes for KG-3 grade from an analysis conducted by Folger (also provided in Folger & Breda, 1989). | Technical report of the STAR experiment. Only significance levels reported (analysis-of-variance model results can not be used as they are only reported as a summary of the analyses showing significance levels (.05, .01, .001, all levels are <=). Provide effect sizes for KG-3 grade from an analysis conducted by Folger (also provided in Folger & Breda, 1989). |
